# Knockdown of Cytochrome P450 Genes *Gh_D07G1197* and *Gh_A13G2057* on Chromosomes D07 and A13 Reveals Their Putative Role in Enhancing Drought and Salt Stress Tolerance in *Gossypium hirsutum*

**DOI:** 10.3390/genes10030226

**Published:** 2019-03-18

**Authors:** Richard Odongo Magwanga, Pu Lu, Joy Nyangasi Kirungu, Qi Dong, Xiaoyan Cai, Zhongli Zhou, Xingxing Wang, Yuqing Hou, Yanchao Xu, Renhai Peng, Stephen Gaya Agong, Kunbo Wang, Liu Fang

**Affiliations:** 1 State Key Laboratory of Cotton Biology/Institute of Cotton Research, Chinese Academy of Agricultural Science (ICR, CAAS), Anyang, Henan 455000, China; magwangarichard@yahoo.com (R.O.M.); lupu1992@cricaas.com.cn (P.L.); joynk@cricaas.com.cn (J.N.K.); dongqi@cricaas.com.cn (Q.D.); caixy@cricaas.com.cn (X.C.); zhouzl@cricaas.com.cn (Z.Z.); wangxx@cricaas.com.cn (X.W.); houyq@cricaas.com.cn (Y.H.); xuyanchao2016@163.com (Y.X.); 2 School of Biological and Physical sciences (SBPS), Main campus, Jaramogi Oginga Odinga University of Science and Technology (JOOUST), P.O Box 210-40601, Bondo 210-40601, Kenya; sgagong@jooust.ac.ke; 3 Research Base in Anyang Institute of Technology, State Key Laboratory of Cotton Biology/Anyang Institute of technology, State key laboratory of cotton R.P, Anyang, Henan 455000, China; aydxprh@163.com

**Keywords:** cytochrome P450 genes, virus-induced-gene-silencing, oxidant and antioxidant enzymes, stress-responsive genes, drought and salt stress

## Abstract

We identified 672, 374, and 379 *CYPs* proteins encoded by the *CYPs* genes in *Gossypium hirsutum, Gossypium raimondii*, and *Gossypium arboreum*, respectively. The genes were found to be distributed in all 26 chromosomes of the tetraploid cotton, with chrA05, chrA12, and their homeolog chromosomes harboring the highest number of genes. The physiochemical properties of the proteins encoded by the *CYP450* genes varied in terms of their protein lengths, molecular weight, isoelectric points (pI), and even grand hydropathy values (GRAVY). However, over 99% of the cotton proteins had GRAVY values below 0, which indicated that the majority of the proteins encoded by the *CYP450* genes were hydrophilic in nature, a common property of proteins encoded by stress-responsive genes. Moreover, through the RNA interference (RNAi) technique, the expression levels of *Gh_D07G1197* and *Gh_A13G2057* were suppressed, and the silenced plants showed a higher concentration of hydrogen peroxide (H_2_O_2_) with a significant reduction in the concentration levels of glutathione (GSH), ascorbate peroxidase (APX), and proline compared to the wild types under drought and salt stress conditions. Furthermore, the stress-responsive genes 1-Pyrroline–5-Carboxylate Synthetase (*GhP5CS*), superoxide dismutase (*GhSOD*), and myeloblastosis (*GhMYB*) were downregulated in VIGS plants, but showed upregulation in the leaf tissues of the wild types under drought and salt stress conditions. In addition, *CYP450*-silenced cotton plants exhibited a high level of oxidative injury due to high levels of oxidant enzymes, in addition to negative effects on CMS, ELWL, RLWC, and chlorophyll content The results provide the basic foundation for future exploration of the proteins encoded by the *CYP450* genes in order to understand the physiological and biochemical mechanisms in enhancing drought and salt stress tolerance in plants.

## 1. Introduction

Cytochrome P450 (CYPs) is a diverse gene superfamily, often referred to as an ancient enzymatic system, with a wide distribution in plants and animals [1]. Moreover, their number has exploded in plants [2]. The distinctive feature of the P450 enzymes is their light absorption spectrum; they absorb light within 450 nm by the use of heme cofactor [3]. Once the enzymes are activated, they catalyze a series of oxidative reactions such as lipid and steroidal hormone synthesis, which in turn leads to activation or inactivation of intracellular and extracellular chemicals [4]. The gene members of the cytochrome super family are coded with a CYP prefix, followed by a numeral for the family, a letter for the subfamily, and another numeral for the individual gene [5]. They are classified based on amino acid sequence similarity; for instance, any two P450 proteins with a sequence identity of <40% are classified as members of the same family, while those with identity of 55% are classified as members of the same subfamily [6]. The P450 enzymes are believed to have evolved due to evolutionary adaptations as a result of metabolic and environmental changes in both plants and animals [3].

In plants, the *CYP450*s are of two types, A-type and non-A-type [7]. *PgCYP736B*, which belongs to the A-type, originated from a single ancestor and has been found to play important roles in the synthesis of secondary metabolites that enhance plants’ adaptation to biotic and abiotic stress conditions [8]. Several other cytochrome P450 families in various plant species play an important role in enhancing drought and salt tolerance [9]. Moreover, high numbers of *CYP450* genes have been identified in a number of plants; for instance, a total of 1415 cytochrome P450 sequences have been identified in *Arabidopsis thaliana*, *Vitis vinifera*, *Carica papaya*, *Physcomitrella patens*, *Oryza sativa*, and *Populus trichocarpa* [10]. The plants have a relatively large number of *CYP450* genes and their expansion in land plants has generated the largest family of enzymes in plant metabolism [7]. Several studies have shown that the *CYP450* genes have a significant role in enhancing plants’ adaptation to various abiotic stress factors; for instance, overexpression of *CYP709B3* genes enhanced salt stress tolerance in transgenic *Arabidopsis* plants [11]. Moreover, overexpression of *PgCYP736B* gene conferred enhanced resistance to salt stress via decreased production of hydrogen peroxide (H_2_O_2_) accumulation, increased carotenoid levels, and abscisic acid biosynthesis gene expression [8].

Drought and salinity stress are the main abiotic stress factors, with highly negative effects on plant growth and development [12]. Due to ever-changing environmental conditions and a high rate of pollution, plants have evolved various survival strategies to cope with water deficit and salinity stress [13]. The evolutionary strategy adopted by plants, due to their sessile life, is through physiological and or molecular processes of modulating the concentration levels of the various phytohormones and induction of the stress response genes [14,15]. Among the most important plant secondary metabolites are the brassinosteroids (BR), known to be involved in a series of plant physiological pathways, in addition to plant growth and development [16]. The CYPs monooxygenases have been found to play a critical role in the BR biosynthetic pathways [17], as the various enzymes involved in catalyzing the conversion of the intermediate products to BR encode CYPs monooxygenases, such as dwarf4 (DWF4), steroid 5-α-reductase DET2 (DET2), CYP85A2, CYP85A1, and carboxypeptidase (CPD) [18]. Several studies have demonstrated that the *CYP450* genes have a functional role in enhancing tolerance to environmental stress; for instance, the CYP82 members have been reported to be highly upregulated under abiotic stress in *Nicotiana tabacum* [19], *Pisum sativum* L [20], *Glycine max* [21], and *A. thaliana* [22]. Moreover, genome-wide identification of the *CYP450* genes in *A. thaliana* revealed the highest number of proteins encoded by the *CYP450* genes, compared to any other known plant transcriptome factors [23]. Thus their abundance could be integral in promoting a plant’s survival under stressful conditions. In addition, further studies have shown that the CYPs constitute about 1% of the protein coding sequences and are one of the largest enzyme families in plants [24]. In monocotyledonous plants such as rice, a hydrophytic plant, known to be highly water-dependent, has been found to harbor about 450 *CYPs* genes [10]. To date several stress-responsive genes have been identified in plants, such as the late embryogenesis abundant (*LEA*) genes [25] and *MYBs* [26], among others, but not in the high numbers detected for the *CYPs*. Therefore, this shows the diverse evolution of the plant *CYP450* genes, which could have occurred hand in hand with a whole-genome duplication event in a number of plants. Even though the *CYP450* genes are abundant and have diverse roles in plants, no research has ever been done to characterize and evaluate their roles in cotton. Moreover, despite rapid advances in recent years, the function of a large proportion of the plant P450 enzymes remains either unknown or only superficially understood [27].

Cotton is the primary source of raw materials to the textile industry, but its production has continued to decline due to the effects of various environmental stresses [28]. Due to intensive selection and inbreeding, the genetic diversity of cultivated cotton has been significantly eroded, and improving the quality and adaptability of cotton plants through conventional methods has become almost impossible. The completion of genome sequencing of three cotton varieties, the tetraploid, *Gossypium hirsutum* [29], diploid of D genome, *Gossypium raimondii* [30], and diploid cotton of A genome, *Gossypium arboreum* [31], has provided a perfect platform for the exploration of the various cotton transcription factors, aimed at elucidating their possible roles in plants under abiotic and abiotic stress conditions. In this research work, we carried out genome-wide identification of the *CYP450* genes in the three cotton species, determining their protein characteristics and their expressions under drought and salt stress conditions. Moreover, we evaluated their role in cotton through virus-induced gene silencing (VIGS), in upland cotton, *G. hirsutum*, CRI12, the main cotton produced in major cotton-growing regions in China. The findings of this research work will be vital for cotton breeders to utilize these diverse genes in unraveling the perennial problem of salinity and water deficit conditions, which has become a major problem not only in cotton production but also in food crops.

## 2. Materials and Methods

### 2.1. Identification and Physiochemical Analysis of the Proteins Encoded by the Cotton Cytochrome P450 Genes

The conserved *CYP450* protein domain was obtained from Hidden Markov Model (HMM) PF00067. The *CYP450* proteins’ HMM profile was used as a query to carry out a HMMER search (http://hmmer.janelia.org/) against the *G. hirsutum* genome database (http://mascotton.njau.edu.cn), *G. arboreum* obtained from the cotton genome project (http://cgp.genomics.org.cn), and *G. raimondii* obtained from phytozome (https://phytozome.jgi.doe.gov/pz/portal.html), using a stringent condition with an E value of less than 0.01. All the sequences were further analyzed through an online tool, Pfam Scan (https://www.ebi.ac.uk/Tools/pfa/pfamscan/) and SMART search. (http://smart.embl-heidelberg.de/smart/). Additionally, physiochemical properties, such as molecular weight (MW), isoelectric point (pI), grand average of hydropathy (GRAVY) values, and charge of the deduced polypeptides, were calculated by ExPasy (http://web.expasy.org/ compute_pi/). Moreover, we determined the cis-regulatory elements by analyzing the genomic sequence regions of 1 kb up- and downstream of the 5′-untranslated region (UTR) through an online tool, the PLACE database (http://www.dna. affrc.go.jp/PLACE/).

### 2.2. Chromosome Mapping and Subcellular Sublocalization Prediction of the Proteins Encoded by the G. hirsutum CYP450 Genes

The subcellular localization prediction for all the upland cotton *CYP450* proteins was carried out through an online tool, Wolfpsort (https://wolfpsort.hgc.jp/). The results were further validated by two other types of online software, the TargetP1.1 (http://www.cbs.dtu.dk/services/TargetP/) server and Protein Prowler Subcellular Localization Predictor version 1.2 (http://bioinf.scmb.uq.edu.au/pprowler_webapp_1-2/). Finally, chromosomal mapping of all the cotton *CYP450* genes was done based on their respective positions using Mapchart 2.2 software.

### 2.3. RNA Sequencing Analysis of the Cotton Cytochrome (CYP450) Genes Profiled under Drought and Salt Stress Conditions in Various Tissues

The RNA expression data for the putative cotton *CYP450* genes profiled under abiotic stress conditions were retrieved from a cotton functional genome database (https://cottonfgd.org). The RNA sequencing data used were from the root, stem, and leaf tissues of upland cotton, *Gossypium hirsutum*, sampled at 0 h, 1 h, 3 h, 6 h, and 12 h under osmotic and salt stress conditions, in a hydroponic setup. Osmotic and salt stress were imposed by the use of PEG-6000 and sodium chloride (NaCl) solution, respectively. The read per kilobase per million mapped reads (RPKM) values were log10 transformed and used for the construction of the heat map using MeV heatmap generation software.

### 2.4. Plant Materials and Abiotic Stress Treatment

In order to investigate the expression levels of the upland cotton, *G. hirsutum CYP450* genes under drought and salt stress conditions, an upland cotton *G. hirsutum* accession number CRI-12 (G09091801–2) was used. The CRI-12 cotton germplasm was developed by Cotton Research Institute of Chinese Academy of Agricultural Sciences (CRI-CAAS), in Anyang, Henan Province, China. CRI-12 is the dominant cotton cultivar grown in China due to its high productivity and relatively superior fiber quality [32], but it is highly susceptible to various environmental stress factors. Healthy seeds were delinted before pre-germination was conducted. The germinated seedlings were then transferred to a hydroponic setup, with Hoagland nutrient solution in a well-controlled room, with temperature set at 28 °C day/25 °C night, 14 h photoperiod, and 60–70% relative humidity [33]. At the three-leaf stage, the seedlings were exposed to drought and salt stress simultaneously. For drought stress, 17% PEG-6000 [34], was added, while for the salt stress condition, 250 mM of NaCl solution was added to the Hoagland nutrient solution [35]. In both drought and salt stress treatments, a hydroponic setup was employed in order to determine the expression levels of the various cotton *CYP450* genes at 0 h, 1 h, 3 h, 6 h, and 12 h through RT-qPCR analysis; moreover, the hydroponic setup is preferred for determining the effective gene expression under various abiotic stress treatments during short time intervals compared to the use of soil medium [36]. The samples were frozen in liquid nitrogen upon collection, and stored at −80 °C, awaiting RNA extraction. In each treatment three biological replicates were carried out.

### 2.5. RNA Isolation and RT-qPCR Analysis

Total RNA was extracted from the samples using a modified CTAB method [37]. The RNA quality was evaluated on a 1.2% formaldehyde agarose gel and Nano drop machine (Thermo Fisher, Waltham, MA, USA), with optical density (OD) ratios set at 260 nm/280 nm. Dnase-1 was used to remove any form of DNA contamination. To examine the expression level of the selected genes by real time quantitative polymerase chain reaction (RT-qPCR). The RNAs were transcribed to cDNA using TranScript-All-in-One First-Strand cDNA Synthesis SuperMix (TransGen Biotech, Beijing, China) for qPCR, in accordance with the manufacturer’s instructions. The genes specific primers were designed by Primer Premier 5 [38] and the *GhActin* gene was used as the reference gene for internal control. The Fast Start Universal SYBRgreen Master (Rox) (Roche, Mannheim, Germany) was used to carry out RT-qPCR as outlined in the manufacturer’s protocol. The RT-qPCR reaction mixture was prepared in a volume of 20 mL, with 10 mL of SYBR green master mix, cDNA template (2 mL), ddH_2_O (6 mL), and 2 mL of each of the primer for a final concentration of 10 mM. The PCR thermal cycling conditions were set as follows; 95 °C for 10 min, 40 cycles of 95 °C for 5 s, 60 °C for 30 s, and 72 °C for 30 s. Data were collected during the extension step: 95 °C for 15 s, 60 °C for 1 min, 95 °C for 30 s, and 60 °C for 15 s. Three biological replicates and three technical replicates were performed per cDNA sample. The details of all the primers used for the RT-qPCR are listed in Table S1. The relative expression levels of the genes used in the RT-qPCR validation were analyzed as per the 2^−ΔCT^ method [39].

### 2.6. Preparation of Inocula and Inoculation of Plants

Virus-induced gene silencing (VIGS) was performed using tobacco rattle virus (TRV) according to the methods described in a previous study [40]. The production of a TRV-based VIGS vector suitable for *Agrobacterium*-mediated inoculation and the production of a recombinant pTRV vector for the silencing of *Gh_A13G2057* and *Gh_D07G1197* with 351 bp and 416 bp, respectively, in cotton was carried out as described by Gao et al. [41]. A gene-specific fragment from the *CYP450* genes were amplified by PCR using the following primers; for *Gh_A13G2057* the restriction enzyme used was; *SacI* 5′C**GAGCTC**ACACCCACAAACTCCCCTA3′ and *XhoI* 5′C**CTCGAG**TCTGTACCAATCGACCCAA3′; and for *Gh_D07G1197, SacI* 5′C**GAGCTC**TGGATG GATTTACAAGG GAT3′ and *XhoI* 5′C**CTCGAG**GGAGTAGATGGATGCTTTCG3′. Binary plasmids harboring pTRV1, and either empty pTRV2 or the recombinant pTRV2 containing 35S:: *Gh_A13G2057* and 35S::*Gh_A13G2057* were electroporated into *Agrobacterium tumefaciens* strain LBA4404 and selected on plates containing kanamycin (50 μg mL^−1^) and rifampicin (50 μg mL^−1^). Preparation of the culture for inoculation was done as described Gao et al. [41]. In order to confirm the success of the gene silencing, we carried out gel electrophoresis of the RNA extracted from the leaf tissues of phytoene desaturase (PDS); positive control), wild type (non-VIG plants), TRV:00 (control) and TRV:*CYP450*s (VIGS plants) using the two genes’ specific primer, *Gh_A13G2057* forward sequence “CCACAAACTCCCCTACCTTCA” and the reverse sequence CATAGCCACCC AATTTCGCAT; *Gh_D07G1197* forward sequence GCAAGGGGAAGCCTGATTTT and reverse sequence “GTCGGTGCCAGCAGTGAATA” the *TRV1* primer sequence, forward sequence ‘TTAC AGGTTATTTGGGCTAG and reverse sequence CCGGGTTCAATTCCTTATC and the primer sequence for *TRV2*, forward sequence TGTTTGAGGGAAAAGTAGAGAACGT and the reverse sequence TTACCGATCAATCAAGATCAGTCGA.

### 2.7. Abiotic Stress Treatment of the VIG Plants, Wild Type and Infused with TRV:00 (Empty Vector)

The cotton seeds were germinated in moist sand at the emergence of the radicle; the germinated seeds were then transferred into small pots with vermiculite and humus at a ratio of 1:1. In each of the two genes, a total of 45 individual plants were used, in addition to the positive control and the wild type, so a total of 135 plants were set up per treatment. The high number was due to the various parameters evaluated: CMS, ELWL, RLWC, and RT-qPCR analysis of the stress-responsive and *CYP450* genes’ expression under salt and drought stress conditions. Approximately 3 mL were administered to the cotton cotyledons before the emergence of the first true leaf. The seedling cotyledons that were infiltrated with pTRV1 and pTRV2 were used as negative controls. Each inoculation was carried out three times; six seedlings were infiltrated for each construct, three replicates. When the VIGS phenotype was visible, and at the three-leaf stage, the WT, positive control, and VIG plants were subjected to salt and drought stress; drought stress was imposed by total withdrawal of watering, while salt stress was carried out by watering with 250 mM of sodium chloride solution [25].

### 2.8. Physiological and Biochemical Evaluations of the VIGS Plants and Wild-Type Cotton Seedlings under Drought and Salt Stress Conditions

The VIGS plants and the wild type were evaluated under drought and salt stress conditions at the three-leaf stage after infusion. For drought stress no watering was done, while for salt stress, 250 mM of NaCl solution was applied, and the application of salt solution was staggered in three levels with 50 mL, 150 mM, and 250 mM for a period of eight days, while drought stress was imposed by withholding water and allowing the plants to be progressively exposed to osmotic stress/dry conditions [42]. Physiological traits measured were saturated leaf weight (SLW), excised leaf water loss (ELWL), and cell membrane stability (CMS), determined through ion leakage concentrations. For SLW, a fresh leaf weight was taken; thereafter, they were submerged in distilled water overnight, after which its turgid weight measured. A leaf sample was taken from each plant. In determining ELWL, the leaf samples were immediately weighed for their fresh weight (FW); the leaf samples were then left on laboratory bench at room temperature overnight. After 24 h the weights of the wilted leaf samples were then recorded. The leaf samples were then oven-dried at 80 °C to obtain their dry weights (DW). ELWL was then calculated by adopting the formula described by Clarke and McCaig [43]. For the CMS, leaf discs weighing 0.5 g were taken from each cotton treatment. Leaf discs were then washed with distilled water, followed with deionized water, before being placed in sterilized test tubes. In each test tube, 9 mL of deionized water was added, and left at room temperature for 24 h. After 24 h, the test tubes were shaken before measuring the electrical conductivity (EC) of the water by a conductivity meter. Upon taking the measurements (T_1_), the leaves were autoclaved at 70 °C for 20 min. The samples were then cooled to room temperature before total EC values were taken (T_2_). The CMS was calculated using the formula described by Fokar [44]. CMS, ELWL, and SLW traits have been used in screening for drought tolerance in a number of field plants [45]. Furthermore, levels of oxidants and antioxidant enzymes such as glutathione (GSH), ascorbate peroxidase (APX), proline, and hydrogen peroxide (H_2_O_2_) were quantified according to the method described by Bartosz [46]. All the parameters were determined after eight days of post-stress treatment in three biological replications. Finally, we examined the root architecture of the VIGS plants compared to that of the wild types (WT) under drought and salt stress conditions.

### 2.9. Stress-Responsive Gene Profiling in VIGS Plants and Wild Types under Drought and Salt Stress Conditions

In order to investigate the knockdown effect of the two genes in upland cotton, three stress-responsive genes were used to evaluate their expression levels in the leaves of the VIGS plants, wild type, and the positive controlled plants under salt and drought stress conditions. The leaf samples were obtained after eight days of stress exposure. The RNA was extracted from the samples using a modified CTAB method [37], and the RNA quality assessment was done by agarose gel electrophoresis using standard protocols and concentrations determined by the Nano drop method described in Section 2.5. The RNAs were transcribed to cDNA using TranScript-All-in-One First-Strand cDNA Synthesis SuperMix (TransGen Biotech, Beijing, China) for qPCR, in accordance with the manufacturer’s instructions. Gene-specific primers were designed for three stress-responsive genes, cotton pyrroline-5-carboxylate synthase (*GhP5CS*) F “TTGAAATAGTGGACGACGTGGC” and the R “CTCAGCGCCTAGACCAAATCG”; cotton superoxide dismutase (*GhSOD*) F “CATCTCTCACGCACTCTGTC” and R “CCTTAGCCATTTC TGTCTGTG” and lastly the cotton myeloblastosis (*GhMYB*) F “TGGGAGTAGAGGAGGAGAAGC” and R “TTGAGGTGCCTGTGGATTG”. *GhActin* gene was used as the reference gene for internal control. The Fast Start Universal SYBRgreen Master (Rox) (Roche, Mannheim, Germany) was used to carry out RT-qPCR as outlined in the manufacturer’s protocol. The RT-qPCR reaction mixture was prepared in a volume of 20 mL, with 10 mL of SYBR green master mix, cDNA template (2 mL), ddH_2_O (6 mL), and 2 mL of each of the primer for a final concentration of 10 mM. The PCR thermal cycling conditions were set as follows: 95 °C for 10 min, 40 cycles of 95 °C for 5 s, 60 °C for 30 s, and 72 °C for 30 s. Data were collected during the extension step: 95 °C for 15 s, 60 °C for 1 min, 95 °C for 30 s, and 60 °C for 15 s. Three biological replicates and three technical replicates were performed per cDNA sample.

## 3. Results

### 3.1. Identification and Physiochemical Properties of the Cotton Cytochrome P450 (CYPs) Genes

The cotton *CYP450* genes were identified by the use of the PFAM number, PF00067, downloaded from the Pfam database. The candidate gene sequences were further evaluated through Pfam scan and SMART search; all sequences with no CYP functional domain were eliminated from further analysis. As a result of the analysis, 672, 373, and 379 *CYPs* genes were identified in *G. hirsutum*, *G. raimondii*, and *G. arboreum*, respectively. The number of proteins encoded by the cotton *CYP540* genes were in agreement with previous findings in which a higher proportion of the *CYP450* genes have been found to be abundant compared to other genes; for instance, in the model legume *Medicago truncatula*, a total of 151 putative *CYP450* genes were identified, including 135 novel sequences [47]. Moreover, 174 *CYP450* genes have been identified in mulberry through bioinformatics analyses [23]. The polypeptide lengths of the *CYPs* genes in the three cotton species varied; for instance, in *G. hirsutum*, the polypeptide lengths ranged from 51 aa to 2144 aa, molecular weights ranged from 5.95 kDa to 245.579 kDa, the isoelectric points (pI) was predicted to range from 4.153 to 10.971, and the GRAVY ranged from −0.926 to 0.376 (Table S2). In the two diploid cotton varieties, *G. arboreum* of the A genome proteins encoded by the *CYP450* genes exhibited varied physiochemical properties, with protein lengths ranging from 97 aa to 1002 aa, the GRAVY value ranged from −0.812 to 0.309, pI values ranged from 4.663 to 11.302, and the molecular weight ranged from 11.022 kDa to 114.353 kDa (Table S3). Lastly, the *CYP450* proteins from *G. raimondii* of the D genome exhibited similar properties, with the GRAVY value predicted to range from −0.406 to 0.188; pI values ranged from 5.126 to 10.495 and protein lengths ranged from 153 to 985 amino acids (aa) (Table S4). The results were in agreement with previous findings in which a rice *CYP450*-like gene, *Os08g01480*, encoded a protein type of the CYP450 domain was found to have 67–390 aa, which falls within the polypeptide range of 55 aa to 2144 aa identified for the *CYP450* domains in cotton [48]. Analysis of the various physiochemical properties of the proteins encoded by the *CYP450* genes revealed that the proteins were both hydrophobic and hydrophilic in nature, though over 99% of the proteins in the three cotton species had GRAVY values below 0, an indication that the majority of the proteins were hydrophilic in nature. Hydrophilicity is a dominant attribute among the various proteins encoded by a number of stress-responsive genes such as the *LEA* genes, the proteins encoded by the *LEA* genes have been found to be very versatile, adaptive, hydrophilic proteins, generally referred to as molecular shields for their anti-stress properties, attributable to partial or complete structural randomness [49].

### 3.2. Subcellular Localization Analysis of the Cotton Cytochrome P450

In the determination of the possible subcellular localizations of the *CYP450* proteins encoded by the *CYP450* genes in cotton, we applied an online tool, WOLFSPORT. We found that the majority of the proteins encoded by the cotton *CYP450* genes were localized within the nucleus, with 675 proteins encoded by the *CPY450* genes being predicted to be located within the nucleus, accounting for over 47% of the *CYP450* proteins encoded by the *CPY450* genes obtained for the three cotton species. The endoplasmic reticulum (ER) was found to be the cellular organelle with the second-highest number of proteins predicted to be embedded within it, with 389 (27.31%) of the proteins encoded by the *CYP450* genes in the three cotton species. However, other cellular structures were also predicted to harbor proteins, such as the plasma membrane with 180 (12.64%), the cytoplasm with 87 (6.11%), the mitochondria with 66 (4.63%), chloroplasts with eight (0.56%), vacuoles with 12 (0.84%), and extracellular structures with only seven proteins, accounting for less than 1% of the proteins encoded by *CYP450* genes in cotton. The results of the subcellular localization prediction of the cotton *CYP450* proteins were in agreement with previous findings that revealed that the endoplasmic reticulum membrane is the primary subcellular target for eukaryotic P450 and CPR enzymes, although some of the P450s are present in animal mitochondria [50], whereas in plants, through green fluorescent protein (GFP) fusion, cinnamate-4-hydroxylase (C4H), a well-characterized plant P450 enzyme, was found to be exclusively embedded in the ER [51]. However, in recent years, P450-mediated reactions have been discovered to occur in the chloroplast [52]. Moreover, a CYP86B1, cytochrome P450 protein required for long-chain omega-hydroxyacid and α is located within the endoplasmic reticulum [53]. The proportions of the various CYP proteins among the three cotton genomes showed a balanced distribution; for instance, the number of nucleus CYP proteins in the tetraploid cotton, *G. hirsutum*, was 321, while for the two diploid cotton types, 177 CYP proteins were detected in the nucleus for *G. arboreum* (At subgenome) and *G. raimondii* (Dt subgenome). Similar observations were made in the other subcellular structures, such as the ER, plasma membrane, cytoplasm, mitochondria, vacuoles, chloroplasts, and extracellular structures. The results of subcellular localization prediction were further confirmed by Pprowler and TargetP, which showed that higher proportions of the *CYP450* proteins were involved in secretory pathways, while the second-largest category was largely similar, in that others designate other cellular structures apart from the mitochondria (mitochondrial targeting peptide mTP) and chloroplasts (chloroplast transit peptide cTP) (Figure S1 and Tables S2–S4).

### 3.3. Cis-Regulatory Element Analysis of the Cotton Cytochrome P450

The cis-regulatory elements were analyzed in order to determine any possible link between the various proteins encoded by the *CPY450* genes and any of the known cis-regulatory elements known to be responsible for enhancing abiotic stress tolerance in plants. In the analysis of the cis-regulatory elements, 29 stress-responsive elements were detected; among them were ABREATCONSENSUS with a signal sequence of YACGTGGC, which plays a role in ABA signaling/enhance abiotic stress tolerance [54]; ABREATRD22 (RYACGTGGYR) ABA-responsive element/dehydration responsiveness; and other several MYB types of cis-regulatory elements such as MYB1AT (WAACCA), MYB2CONSENSUSAT (CANNTG), MYBATRD22 (CTAACCA), and MYBCORE (CNGTTR) (Table S5). The results obtained were consistent with previous findings in which similar cis-regulatory elements have been detected for other proteins encoded by stress-responsive genes such as *LEA* [25]. Moreover, the *CYP707A* gene has recently been discovered to be an ABA 8′-hydroxylase, an enzyme that degrades ABA in order to break seed dormancy [55]. Moreover, overexpression of the *CYP707A* gene in *Arabidopsis* improved tolerance to dehydration [56]. Thus, the detection of the various cis-regulatory elements is an indication of the vital role played by the cotton *CYP450* genes in enhancing drought and salt stress tolerance in cotton.

### 3.4. Chromosomal Mapping of the Upland G. hirsutum Cytochrome CYP450 Genes

Gene distribution and the physical architecture of the cell are known to be significantly correlated [57]. Therefore, we sought to determine the mapping and distribution of the cotton *CYP450* genes across their genomes. The genes were distributed in all 26 chromosomes of the upland tetraploid cotton, though in an asymmetrical pattern. Interestingly, in each of the chromosomes, their corresponding homeolog chromosome pair contained almost equal numbers of genes. The analysis of the *CYP450* gene-clustering pattern in *Zea mays* showed that evolutionarily related *CYP450* genes belonging to the same sub-family were clustered on the same chromosome [58]; this could explain why homeolog chromosomes harbored almost equal numbers of *CYP450* genes. The highest gene loci were detected in chrA05 and its homeolog ChrD05, with 40 and 41 genes, respectively; the chromosome with the second-highest number of gene loci was chrA12 and its homeolog chrD12, with 38 and 34 genes, respectively. In comparing the gene distribution between the two subgenomes, the *CYP450* genes’ distribution across the tetraploid cotton genome occurred in a symmetrical pattern, with 314 and 331 *CYP450* genes found to be distributed in the At and Dt subgenomes, respectively. However, the number of *CYP450* genes detected for the two diploid cotton species was much higher than the number of genes mapped in either of the two subgenomes, *G. raimondii* and *G. arboreum*—373 and 379 *CYP450* genes, respectively. The low number of *CYP450* genes in the At and Dt subgenomes could be explained by either gene loss or poor annotation, with 27 genes not mapped and thus located within the scaffold region (Figure S2, Tables S3 and S4).

### 3.5. RNA Sequencing Analysis and RT-qPCR Validation of the Upland Cotton Cytochrome P450 Genes under Salt and Drought Stress Conditions

**Figure 1:**
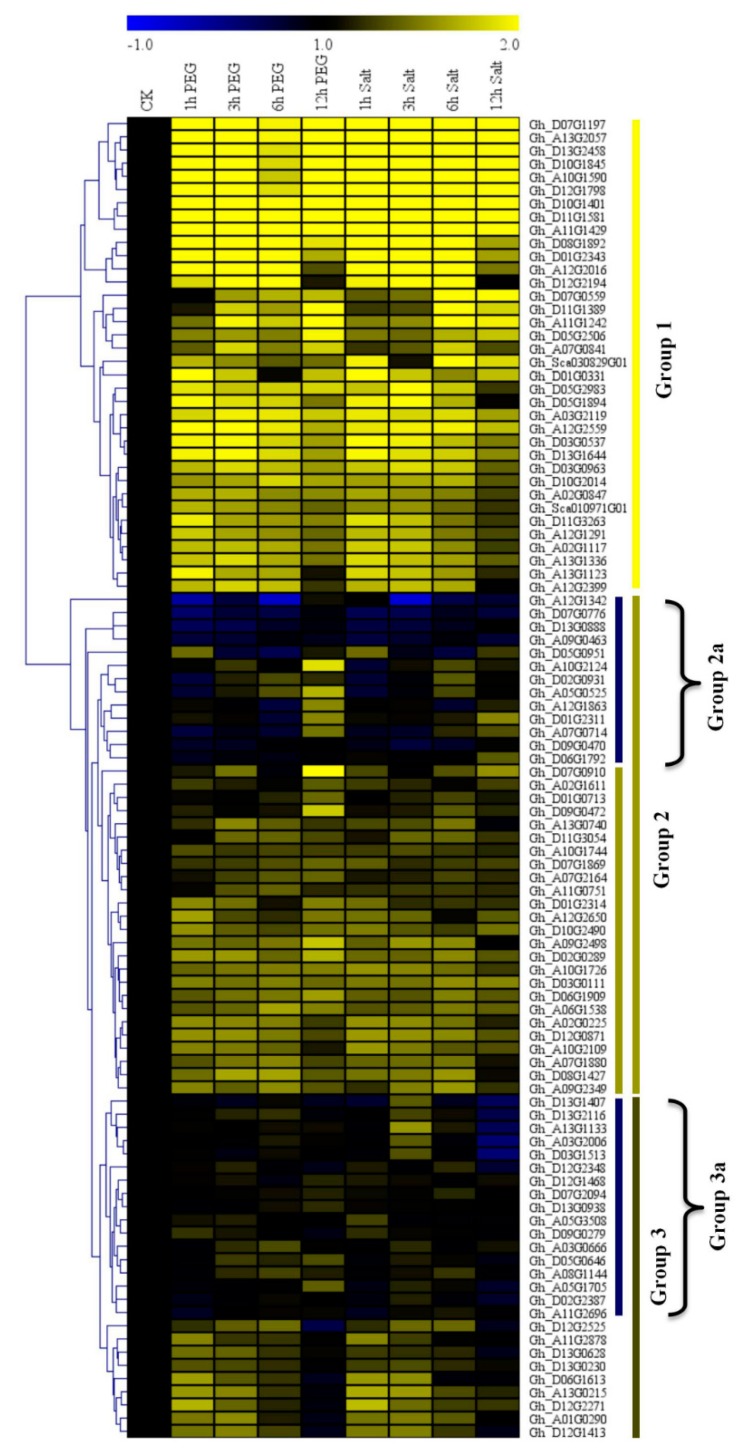
RNA sequencing analysis for the top 100 upland cotton *Cytochrome P450* genes profiled under drought and salt stress conditions. The heat map was visualized using the Mev.exe program (shown by log 10 values) in the control, and in treated samples after 0 h, 1 h, 3 h, 6 h, and 12 h of drought and salt treatment. Yellow—upregulated, blue—downregulated, black—no expression. PEG: Polyethylene glycol.

**Figure 2:**
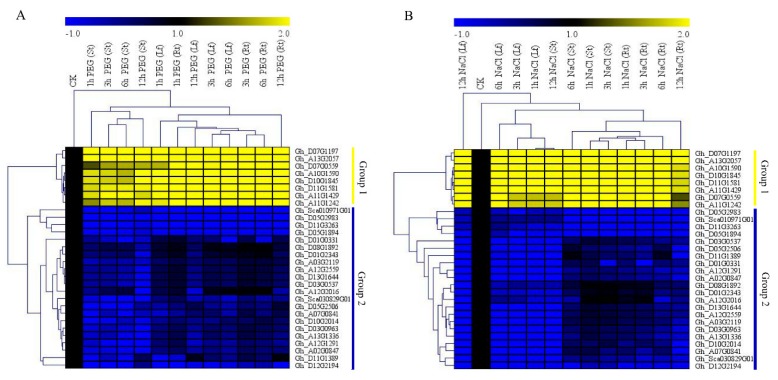
RT-qPCR analysis of the 30 highly upregulated cotton cytochrome P450 genes as obtained from the RNA sequence data. The heat map was visualized using the Mev.exe program (shown by log 2 values) in control, and in treated samples after 0 h, 1 h, 3 h, 6 h, and 12 h of drought and salt treatment. (**A**) Osmotic stress treatment by 17% PEG-6000; (**B**) salt stress treatment by applying 250 mM of NaCl solution. Yellow—upregulated, blue—downregulated, black—no expression.

We obtained RNA profile data from the cotton functional data base (https://cottonfgd.org/search/). The RNAs were profiled under salt and drought conditions at 0 h, 1 h, 3 h, 6 h, and 12 h post-stress treatment. Based on the raw data and their transformed log 10 values, the top 100 genes were further analyzed and a heatmap constructed. The genes were classified into three categories, with group 1 exhibiting higher expression levels under drought and salt stress conditions, while the group 2 members were largely differentially expressed with the majority of the genes exhibiting downregulation or showed no expression; however, the sub members of group 2, group 2a, were significantly downregulated, but some showed upregulation at specific time points. For instance, after 12 h of drought stress imposed by 6000-PEG, the majority of the genes exhibited upregulation. Group 3 members showed partial up- and downregulation, though some were not expressed as specific time points (Figure 1). The two genes used in further analysis of the cotton *CYPs* genes were highly upregulated under drought and salt stress conditions. The high expression levels of the two genes showed that they could play a role in plants under drought stress conditions. We later carried out RT-qPCR validation of the highly expressed genes as per the RNA sequencing data; 30 genes were used for the RT-qPCR expression analysis using three tissues, the leaf, stem, and root tissues of *G. hirsutum*. The gene expressions were stratified into two groups, group 1 with eight genes, *Gh_D07G1197, Gh_A13G2057, Gh_D07G0559, Gh_A10G1590, Gh_A10G1845, Gh_D11G1581, Gh_A11G1429*, and *Gh_D11G1242*, were highly upregulated, just as in the RNA sequencing profiling; among them were *Gh_A13G2057* and *Gh_D07G1197*. The group 2 members were mainly downregulated and, in some genes, showed no expression, as evident in the black patches within the heatmap (Figure 2).

### 3.6. Expression Analysis of the Two Genes Gh_D07G1197 and Gh_A13G2057 in the Tissues of VIGS Seedlings and Wild-Type Cotton Seedlings

**Figure 3:**
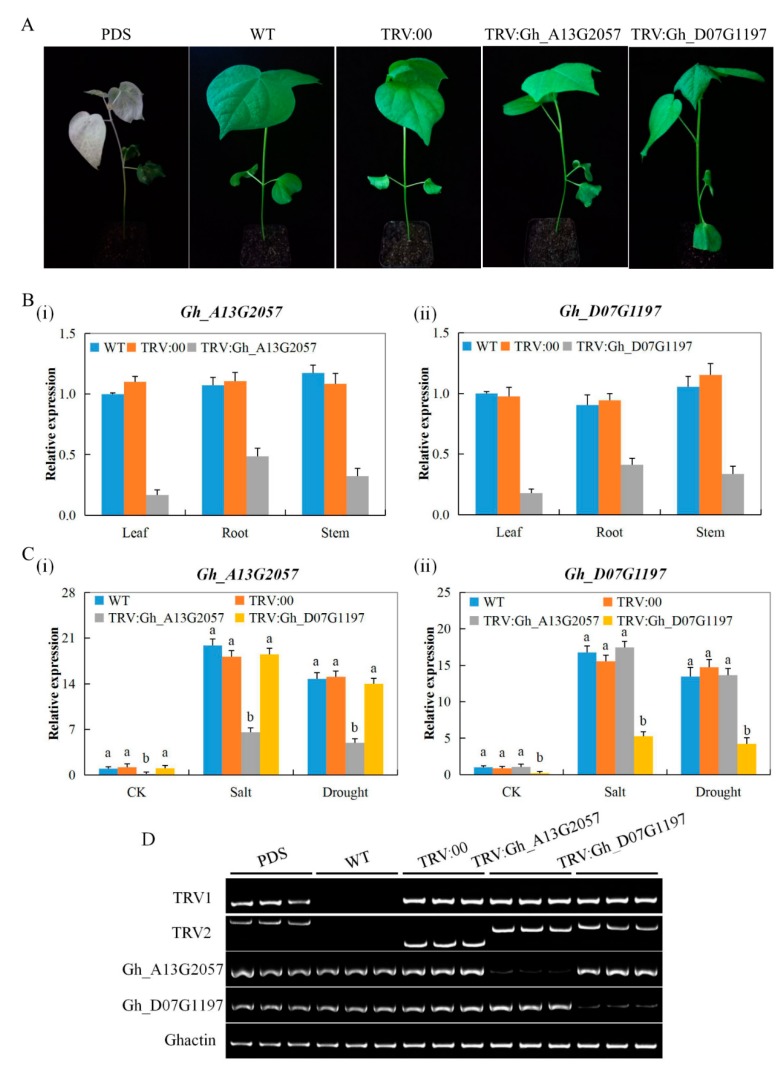
Phenotype observed in the silenced plants with the TRV2:00 empty vector, wild-type plants and *CYP450*-silenced plants at 12 days post-inoculation. (**A**) No obvious symptoms in the leaves of the TRV:00-infected plant (positive control); in each experiment, six plants were used and there were three replicates. (**B**) RT-qPCR analysis of the changes in the expression level of the two *CYP450* genes in the leaf, stem, and root tissues of upland cotton, *G. hirsutum*, plants treated with VIGS before stress exposure. (**C**) RT-qPCR analysis of the changes in the expression level of the two *CYP450* genes in the leaf tissues of *G. hirsutum* cotton plants, wild type, TRV:00 and the VIGS plants under drought and salt stress conditions. (**D**) Gel electrophoresis in determining the effectiveness of gene silencing. TRV2:00 represents the positive control infused with the TRV2 empty vector; TRV2:Gh_*Gh_A13G2057* and TRV2:Gh_*Gh_D07G1197* represent the *CYP450*-silenced plants, PDS: phytoene desaturase; WT: wild type (non-VIG plants). Error bars represent the standard deviation of three biological replicates. Letters a/b indicate statistically significant differences (two-tailed, *p* < 0.01). CK: untreated, Salt: 250 mM NaCl treatment, Drought: 17% PEG-6000 treatment.

In order to evaluate the success of the infusion, phytoene desaturase (PDS) was used, which caused the leaf to lose its chlorophyll and therefore have albino-like symptoms (Figure 3A). In addition, the two genes were profiled in the roots, stem, and leaves in order to determine the tissue in which the gene caused significant downregulation in expression. The genes were found to be significantly downregulated in the leaves compared to the root and stem; however, the genes showed significant upregulation in the tissues of non-silenced plants (wild types) (Figure 3B). In addition, we investigated the knockdown effect of the two *CYP450* genes by profiling the expression levels of the silenced genes in the leaf tissues obtained from the *Gh_A13G2015* and *Gh_D07G1197*-VIGS plants. We observed that the expression levels of the two genes were independent of each other. For instance, the knockdown of *Gh_A13G2015* had no effect on the expression level of *Gh_D07G1197* in the leaf tissues of *Gh_A13G2015*-silenced plants, and the reverse was true for the other gene, even though the two genes were members of the same subfamily (Figure 3C). Finally, we examined the VIGS and non-silenced plants’ leaf tissues through gel electrophoresis, and found that the level of the silenced genes was significantly downregulated; the bands formed very thin lines on the gels compared to those on the non-silenced plants (Figure 3D). The results showed that the two genes were effectively silenced. RNAi has proven to be very efficient in interfering with gene expression in various plant systems and has been a useful tool to identify gene function by effecting degradation of the targeted transcript [59].

### 3.7. Evaluation of Performance of the VIGS Plants and Wild Cotton, G. hirsutum, under Drought and Salt Stress Condition

**Figure 4:**
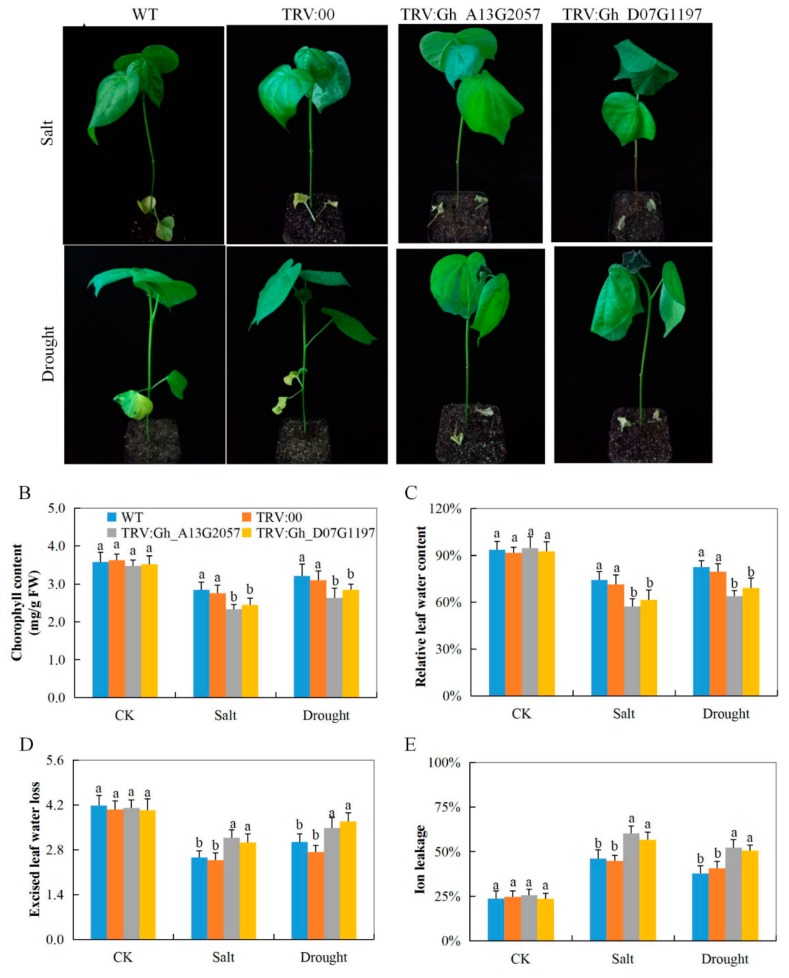
Physiological trait measurements in *Gh_A13G2057 (CYP450)* and *Gh_D07G1197 (CYP450)*-VIGS cotton plants under salt and drought stress conditions. (**A**) Representative images of VIGS (TRV: *CYP450s*, silenced; TRV: 00, control) and the non-VIGS (WT) plants, after eight days of drought and salt stress treatment. Quantitative determination of (**B**) chlorophyll content, (**C**) relative water content (RLWC), (**D**) excised leaf water loss (ELWL), (**E**) cell membrane stability (CMS) as a measure of ion leakage concentration in leaves of the WT, control and *CYP450s*-silenced VIGS. All measurements were done after eight days of stress exposure. In (**B**–**E**), each experiment was repeated three times. Error bars of the physiological trait measurements represent the standard deviation of three biological replicates. Different letters indicate significant differences between wild-type and *CYP450*s-VIGS plants (two-tailed; *p* < 0.01). CK: untreated, Salt: 250 mM NaCl treatment and Drought: 17% PEG-6000 treatment.

The silenced plants showed signs of wilting and dropping leaves after eight days of stress exposure, compared to the wild-type and controlled plants under similar conditions (Figure 4A). The dropping showed that the plant cells lost their turgor pressure. Analysis of various physiological traits, such as the excised leaf water loss (ELWL), saturated leaf weight (SLW), or cell membrane stability (CMS), all registered negative effects among the two VIGS cotton seedlings, whereas, in the control and the wild type, no significant differences were observed; the wild and controlled plants showed signs of positively regulating the effects of drought and salt stress (Figure 4B–E). The results showed that the wild types were more stress-tolerant compared to the VIGS cotton seedlings. In addition, we sought to investigate the biochemical component by assaying the various oxidant and antioxidant enzyme concentration levels. The concentration levels of the antioxidant enzymes, glutathione (GSH), APX, and proline, in the leaves of the VIG plants were significantly reduced compared to their individual concentration levels in the leaves of the wild type (Figure S3A). The low levels of the antioxidant enzymes showed that the VIGS plants had lost the capacity to reduce the effect of oxidative stress caused by high release of reactive oxygen species (ROS). Moreover, the concentrations of the oxidant hydrogen peroxide (H_2_O_2_) were significantly elevated in the leaves of the VIGS plant, but were significantly reduced in the leaves of the wild types under drought and salt stress conditions (Figure S3B–D).

### 3.8. Stress-Responsive Gene Profiling on the Tissues of VIGS Plants and the Wild Types under Drought and Salt Stress Conditions

**Figure 5:**
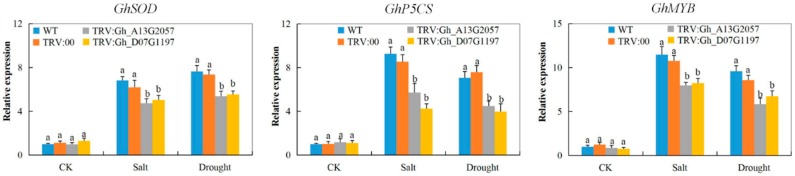
Stress-responsive genes (*GhSOD*, *GhP5CS*, and *GhMYB*) expression profiling in *CYP450s*-VIGS plants. *GhActin* was used as the reference gene. The letters a/b indicate statistically significant differences (two-tailed, *p* < 0.01). Error bars of the gene expression levels represent the standard deviation of three biological replicates. CK: untreated, Salt: 250 mM NaCl treatment, Drought: 17% PEG-6000 treatment.

Analysis of various stress-responsive genes indicated the possibility of the silenced gene’s involvement in enhancing stress tolerance. Three genes were used, cotton Delta-1-pyrroline-5-carboxylate synthetase (*GhP5CS*), cotton myeloblastosis (*GhMYB*), and cotton superoxide dismutase (*GhSOD*). All three genes showed higher expression levels in the leaves of the wild cotton, but were significantly downregulated in the leaves of the VIGS cotton plants under drought and salt stress conditions (Figure 5). The glutamate pathway is the key pathway of proline biosynthesis, in which P5CS enzymes plays an important role; the P5CS enzymes catalyze the activation of glutamate by phosphorylation and also the reduction of the labile intermediate, γ glutamyl phosphate, into glutamate semialdehyde (GSA) [60]. The *P5CS* gene has been found to play an integral role in enhancing abiotic stress in various plants such as wheat [61], and thus the downregulation of *P5CS* gene in the tissues of the VIGS plants showed that the knockdown of the two *CYP450* genes affected the proline biosynthesis pathways, lowering the ability of the VIGS plants to tolerate the effects caused by drought and salt stress.

## 4. Discussion

In this study, we identified a huge number of *CYP450* genes, higher than the major classes of the genes such as *MYBs*, with 672, 373, and 379 genes in *G. hirsutum*, *G. raimondii*, and *G. arboreum*, respectively. The high number of these genes in cotton corroborates previous studies that showed that the proteins encoded by *CYP450* are among the largest gene families in the plant genome [62]. The genes were found to be distributed in all 26 chromosomes and, more significantly, the GRAVY values showed that over 99% of the proteins encoded by the cotton *CYPs* genes were hydrophilic in nature. A number of hydrophilic proteins have been highly correlated to abiotic stress tolerance; for instance, the LEA proteins have been found to protect plants through antioxidants, and as membrane and protein stabilizers during water stress [63]. Moreover, the LEA proteins, together with other hydrophilic proteins, have been found to act as space fillers to prevent cellular collapse in low water conditions [64]. In addition, the hydrophilic OSI-SAP1 protein from rice, encoded by the zinc-finger genes, has been found to be induced when plants are exposed to various stresses [65]. Similarly, several hydrophilic proteins have been found to confer abiotic stress tolerance, such as globulin 1, globulin 2, etc. [66]. Thus, the discovery that a high number of proteins encoded by the cotton *CYPs* genes are hydrophilic is an indication of the vital role they play in enhancing plants’ abiotic stress tolerance.

Some of the cis-regulatory elements known to target the top-ranked plant stress-responsive genes include abscisic acid-responsive element (ABRE) [54], dehydration-responsive element (DRE) [67], and C-repeat [68], among others. In the analysis of the cis-regulatory elements in the promoter regions of the proteins encoded by cotton *CYPs* genes, several cis-regulatory elements were detected. For instance, ABREATCONSENSUS, with a signal sequence of YACGTGGC, which functions in stress-responsive abscisic acid signaling/enhance abiotic stress tolerance, ABREATRD22 (RYACGTGGYR) with a role in ABA-responsive element/dehydration responsiveness, and several other MYB types of cis-regulatory elements such as MYB1AT (WAACCA), MYB2CONSENSUSAT (CANNTG), MYBATRD22 (CTAACCA), and MYBCORE (CNGTTR). These cis-regulatory elements have previously been found to be associated with various stress-responsive genes such as the NAM, ATAF, and CUC (*NAC*) genes [69]. The detection of these regulatory elements points to the putative role of the proteins encoded by cotton *CYP* genes in enhancing abiotic stress tolerance.

Through the RNA sequencing data and RT-qPCR validation, two genes were found to maintain a similar expression pattern under drought and salinity conditions, so we carried out further functional analysis of them through VIGS. The VIGS plants (the plants with the two genes silenced) and the wild type (not silenced) showed variation in terms of their morphological, physiological, and transcription factor induction ability under abiotic stress conditions. Since morphological parameters such as plant height, root length, leaf size, leaf weight, stem length, stem diameter, and leaf number are highly influenced by the environment [70], no measurements were carried out. We concentrated on the physiological parameters and biochemical assays of the oxidants and antioxidant enzymes. In the evaluation of CMS, ELWL, and SLW, the VIGS plants recorded a sharp reduction. There was a significantly higher ion leakage, higher percentage of water loss from the excised leaf, and relatively low weight of the saturated leaf of the VIGS plants compared to their wild types under drought and salt stress conditions. Reduction in SLW, increased ion leakage, and percentage water loss from the excised leaf are indications that the VIGS plants suffered intensive oxidative stress, and thus lost the ability to tolerate the effects of drought and salinity stress [71]. Moreover, oxidant and antioxidant enzyme assays showed that the VIGS plants registered higher levels of oxidant enzymes and a significant reduction of the antioxidant enzymes under salt and drought stress conditions compared to their wild types. The MDA is a measure of lipid peroxidation or degradation levels [72]; the levels of MDA and H_2_O_2_ indicate free oxygen radical reactions occurring in the stressed tissue [73]. Thus, higher concentration levels in the leaves of the VIGS plant showed that the plants suffered more oxidative stress compared to the wild types, which further revealed the vital role played by the proteins encoded by the *CYP450* genes in enhancing drought and salt stress tolerance in cotton.

Finally, we examined the expression levels of three known stress-responsive genes; 1-Pyrroline–5-Carboxylate Synthetase (*P5CS*), superoxide dismutase (*SOD*), and myeloblastosis (*MYB*) on the leaf tissues of the VIGS plants and wild type, under drought and salt stress conditions. All three genes were downregulated in the VIGS plants, but were significantly upregulated in the leaf tissues of the wild types under drought and salt stress conditions. The *P5CS* gene is known to play an integral role in the proline biosynthesis pathway because it encodes for a bi-functional enzyme that catalyzes the rate-limiting reaction in proline biosynthesis in living organisms [74]. Proline functions as a plant osmoprotectant, having been found to accumulate to significantly high levels without disrupting plant metabolic processes during abiotic stress exposure [75]. The downregulation of the *GhP5Cs* genes could explain why H_2_O_2_ was detected in significantly higher concentrations in the leaves of the VIGS plants under salt and drought stress conditions compared to the wild type. The proline biosynthesis pathways were completely stopped since H_2_O_2_ and proline content concentration in the plant tissues occurs in a reciprocal manner: when the proline content is high, the H_2_O_2_ level is significantly reduced, and vice versa [76]. Similarly, the *GhSOD* and *GhMYB* genes were also found to be downregulated in the leaves of the VIGS plants under salt and drought stress conditions. The delicate equilibrium between ROS production and release shifts when plants are exposed to any form of stress. For instance, salt and drought stress leads to water potential imbalance, turgor decrease, hyperosmotic stress, and oxidative stress [77]. Oxidative stress is caused by increased production and accumulation of ROS [78]. The downregulation of the *GhSOD* gene in the VIGS plants impaired the capacity of the proteins encoded by the *GhSOD* genes to scavenge the excess ROS, leading to its high accumulation, thus resulting in oxidative stress, as evident in the high levels of H_2_O_2_, in addition to MDA, in the leaf tissues of the VIGS plants under salt and drought stress conditions compared to their wild types. The proteins encoded by the *SOD* genes are important because they protect the plant cells from the toxic effects of ROS by scavenging superoxide radicals and detoxifying the ROS into non-toxic compounds such as oxygen and water [79].

In plants, transcription factors such as MYB, NAC, and MAPK are ranked top in the context of drought and salinity, indicating their important roles in a plant combating drought and salinity stress [25]. The proteins encoded by the *MYB* genes have been found to play diverse roles in plants under abiotic stress [80]. However, the *GhMYB* gene was found to be downregulated in the tissues of the *Gh_A13G2057*- and *Gh_D07G1197*-silenced plant leaf tissues, an indication that the VIGS plants had lost their ability to tolerate the effects of drought and salt stress.

## 5. Conclusions

In this research work, we identified 672, 373, and 379 *CYPs* genes in *G. hirsutum*, *G. raimondii*, and *G. arboreum*, respectively. The various cotton *CYP450* proteins encoded by the *CYP450* genes exhibited varied physiochemical properties. For instance, the GRAVY values ranged from −0.926 to 0.376; low GRAVY values are associated with hydrophilic proteins, which are mainly encoded by the stress-responsive genes such as the *LEA* genes [25]. Higher percentages of the proteins encoded by the *CYP450* genes were predicted to be located within the ER. The ER provides the channel for the eukaryotic protein secretory pathway. Moreover, the secretory proteins are moved into the ER and then into the protein-folding cycles to fold and assemble themselves; no folded proteins are retained, which makes the ER an important organelle for protecting proteins against aggregation [81]. The VIGS plants’ ability to tolerate the effects of salt and drought stress was significantly reduced, as evident in the higher levels of various oxidant enzymes measured. Moreover, all the stress-responsive genes were downregulated in the leaf tissues of the VIGS plants, but showed significant upregulation in the leaves of the wild types under drought and salt stress conditions. These findings provide a foundation for future exploration of these genes in developing a more resilient cotton genotype with improved performance under various environmental stress factors, including drought and salt stress.

## Supplementary Materials

The following are available online at https://www.mdpi.com/2073-4425/10/3/226/s1: Figure S1: Subcellular prediction of the cotton CYPS proteins encoded by the *CYP450* genes, as determined through the wolfsport subcellular prediction tool; Figure S2: Chromosome mapping of the upland cotton, *G. hirsutum* CYPs. The genes red box were the silenced genes in the determination of the role of the CYPP450 genes in cotton under salt and drought stress conditions; Figure S3: Analysis of oxidant and antioxidant enzymes and in the leaves of the *CYP450s*–VIGS cotton plants under salt and drought stress conditions. Quantitative determination of (A) H_2_O_2_, (B) GSH, (C) Proline activity and (D) APX activity in wild type, control (TRV:00) and TRV:*CYP450s* VIGS plants after eight days salt or drought treatment. In (A–D), each experiment was repeated three times. Bar indicates standard error (SE). Different letters indicate significant differences between wild-type and VIGS plants (ANOVA; *p* < 0.05). CK: normal conditions. Table S1: list of primers used in the expression analysis of the upland cotton cytochrome P450 genes under drought and salt stress conditions; Table S2: Details of the tetraploid cotton, *G. hirsutum* cytochrome P450 proteins, their physiochemical properties and gene annotation, as analyzed through a phylogenetic tree; Table S3: Details of the diploid cotton, *G. arboreum* cytochrome P450 proteins, their physiochemical properties and gene annotation, as analyzed through a phylogenetic tree; Table S4: Details of the diploid cotton, *G. raimondii* cytochrome P450 proteins, their physiochemical properties, and gene annotation as analyzed through a phylogenetic tree; Table S5: Cis-regulatory elements with a profound role in abiotic stress responses found within the promoter regions of the various cotton cytochromes (*CYP450*) genes. The cis-regulatory elements were analyzed in the 1 kb up- and downstream regions of translation start site using the PLACE database.
