# Supplementary material for: Knockdown of Cytochrome P450 Genes Gh_D07G1197 and Gh_A13G2057 on Chromosomes D07 and A13 Reveals Their Putative Role in Enhancing Drought and Salt Stress Tolerance in Gossypium hirsutum
Source: Genes (Basel). 2019 Mar 18;10(3):226. doi: 10.3390/genes10030226 (PMC6471685; doi:10.3390/genes10030226)
Supplement: Supplementary file 1 [file genes-10-00226-s001.zip › Supplementary files/Table S1.docx]

Supplementary Table S1: list of primers used in the expression analysis of the upland cotton cytochrome P450 genes under drought and salt stress conditions

| GENE ID | Forward | Reverse |
| --- | --- | --- |
| Gh_A02G0847 | TTACCAATCACTGACCACG | CAACAGCAGAAGGAGAAGA |
| Gh_A03G2119 | AGACAAAGAACTTGCCGAG | GAACCGAAGAAAGCCCTAC |
| Gh_A07G0841 | ATATTAGGGTGGCGGAGGG | GATGGAGGTGACCGATGAG |
| Gh_A10G1590 | TGATTTTATCCCCATTTTG | TGTTGTCAGTTCTTTTTGT |
| Gh_A11G1242 | CTTGGAAGTTGCCTGGTAA | GACATTTTTTTGGGGTGGA |
| Gh_A11G1429 | CAATGTTCCCCCAAGTTTCC | TTTTTTCTCCACGATTTCCA |
| Gh_A12G1291 | GTCCTGGTGTGGTTTTTGA | ACTATCCCCCCATTTTGAG |
| Gh_A12G2016 | AGAACTTTTGGGTCTACCT | ATCTTATCAATAATTGCGT |
| Gh_A12G2559 | GAGGAGAAAGATGTTGAAAA | AGTAAAAAGTAGGTTGGTGG |
| Gh_A13G1336 | TGAAAAAACTCAAGTACAC | CCAAACAGAGATAAAAATA |
| Gh_A13G2057 | CCAACAACCCTGCTAACTG | CTTCTTCTCCCCACGCCAA |
| Gh_D01G0331 | AGAGAAATGGAAGCAAAGA | CACACCAGTAAGACCGAAG |
| Gh_D01G2343 | ATTTCTCCCACTATCACTT | GCATCTTCTCTGTTCTTTA |
| Gh_D03G0537 | GCTGGCACTGATACTACCTC | AACCCCTTTAATTTCACGAA |
| Gh_D03G0963 | AGTCCCTTCTTGCTCTTTGC | GATTGGGTTTTGTTTTGGTG |
| Gh_D05G1894 | GATGTGGATGTTAGGGGTCA | GAAGGCGTGCAAAAAATTAG |
| Gh_D05G2506 | GCTGTCGTCGAGGAAGGCA | CCGGCAGGAAATCACCCAT |
| Gh_D05G2983 | AACCTTCACAAGATCCAAA | ACAGCTCCTCCTACCACTA |
| Gh_D07G0559 | TTCCTCGTCATCAGCCCCT | ACCTCCGCCACCGTATTTT |
| Gh_D07G1197 | CCTCCTTCGGGATTTGGTA | AAGTGTTCGTTTGGGGTTA |
| Gh_D08G1892 | CTTCTCCAACTCAGCCACC | CCTACAACCAAATCCAATA |
| Gh_D10G1845 | GAATCGAAGGTAGAAGCC | AAATAATCCCTGGACAGC |
| Gh_D10G2014 | CGAGACACCACATCATCAG | ATCGTTTCGAGTTCAGTTA |
| Gh_D11G1389 | TCGTTCCCTGTTGTCGTGG | TAGTGTATTCTCCTGCCGC |
| Gh_D11G1581 | AACTTGATATTCACTCTTGG | ACATACTCGTTTCACTTCGT |
| Gh_D11G3263 | ACTCTTGATGTTATCGGTC | AATAGGGTAAAATGTCCGT |
| Gh_D12G2194 | AATCCAAGGAGGGGAAGCCG | AGGACGAAGGAAGGGAAGGT |
| Gh_D13G1644 | GGCATAGTTGAGGCTGTT | CTTCCTTTGTCGTGGTGA |
| Gh_Sca010971G01 | GTAGAGCAAAAGGCAGAA | CAGTGAAAACGTCAAGGA |
| Gh_Sca030829G01 | TTTCTACCCTTTTCAACAG | AAATAATTAACAGGACCTC |
| GhActin | ATCCTCCGTCTTGACCTTG | TGTCCGTCAGGCAACTCAT |
| [Gh_A13G2057](https://cottonfgd.org/profiles/gene/Gh_A13G2057/) | CCACAAACTCCCCTACCTTCA | CATAGCCACCCAATTTCGCAT |
| [Gh_D07G1197](https://cottonfgd.org/profiles/gene/Gh_D07G1197/) | GCAAGGGGAAGCCTGATTTT | GTCGGTGCCAGCAGTGAATA |
| pTRV1 | TTACAGGTTATTTGGGCTAG | CCGGGTTCAATTCCTTATC |
| pTRV2 | TGTTTGAGGGAAAAGTAGAGAACGT | TTACCGATCAATCAAGATCAGTCGA |
| GhP5CS | TTGAAATAGTGGACGAC GTGGC | CTCAGCGCCTAGACCAAATCG |
| GhSOD | CATCTCTCACGCACTCTGTC | CCTTAGCCATTTCTGTCTGTG |
| GhMYB | TGGGAGTAGAGGAGG AGAAGC | TTGAGGTGCCTGTGGATTG |
