# Supplementary material for: Knockdown of Cytochrome P450 Genes Gh_D07G1197 and Gh_A13G2057 on Chromosomes D07 and A13 Reveals Their Putative Role in Enhancing Drought and Salt Stress Tolerance in Gossypium hirsutum
Source: Genes (Basel). 2019 Mar 18;10(3):226. doi: 10.3390/genes10030226 (PMC6471685; doi:10.3390/genes10030226)
Supplement: Supplementary file 1 [file genes-10-00226-s001.zip › Supplementary files/Table S2.docx]

Supplementary Table S2: Details of the tetraploid cotton, *G. hirsutum* cytochrome P450 proteins, their physiochemical properties and gene annotation as analysed through phylogenetic tree

| **Gene ID** | **Gene Name** | **Gene annotation** | **Strand** | **Length (bp)** | **Protein Length (aa)** | **Molecular Weight (kDa)** | **Charge** | **pI** | **GRAVY** | **Transcript Length (bp)** | **CDS Length (bp)** | **CDS GC Content (%)** | **Exon Number** | **Mean Exon Length (bp)** | **Mean Intron Length (bp)** | **wolfsport subcellular prediction** |
| --- | --- | --- | --- | --- | --- | --- | --- | --- | --- | --- | --- | --- | --- | --- | --- | --- |
| Gh_D12G2036 | CYP76B6 | *Gh_CYP76B6_1* | - | 2,830 | 501 | 57.465 | 13 | 8.551 | -0.183 | 1,506 | 1,506 | 45.3 | 2 | 753 | 1,324.00 | plas |
| Gh_A12G1864 | CYP76B6 | *Gh_CYP76B6_2* | - | 3,044 | 509 | 58.35 | 12 | 8.545 | -0.192 | 1,530 | 1,530 | 45.2 | 2 | 765 | 1,514.00 | plas |
| Gh_D12G2034 | CYP76B10 | *Gh_CYP76B10_3* | - | 1,016 | 314 | 36.024 | 2 | 7.147 | -0.322 | 945 | 945 | 43.1 | 2 | 472.5 | 71 | E.R |
| Gh_D12G2035 | CYP76C1 | *Gh_CYP76C1_4* | - | 402 | 133 | 14.69 | 5.5 | 9.403 | 0.251 | 402 | 402 | 51.2 | 1 | 402 | No intron | chlo |
| Gh_A12G1863 | CYP76B6 | *Gh_CYP76B6_5* | - | 1,571 | 499 | 56.712 | 11.5 | 8.855 | -0.113 | 1,500 | 1,500 | 44.1 | 2 | 750 | 71 | nucl |
| Gh_D08G1892 | CYP76B6 | *Gh_CYP76B6_6* | + | 1,576 | 498 | 56.975 | 12 | 8.844 | -0.068 | 1,497 | 1,497 | 42.2 | 2 | 748.5 | 79 | nucl |
| Gh_A08G2395 | CYP76B6 | *Gh_CYP76B6_7* | + | 1,576 | 498 | 56.928 | 13.5 | 9.112 | -0.089 | 1,497 | 1,497 | 41.9 | 2 | 748.5 | 79 | nucl |
| Gh_D08G1898 | CYP76B6 | *Gh_CYP76B6_8* | + | 1,605 | 498 | 57.272 | 17 | 9.529 | -0.246 | 1,497 | 1,497 | 41.3 | 3 | 499 | 54 | cyto |
| Gh_A08G1588 | CYP76B6 | *Gh_CYP76B6_9* | + | 1,804 | 360 | 40.644 | 12 | 8.923 | -0.064 | 1,083 | 1,083 | 43.4 | 2 | 541.5 | 721 | nucl |
| Gh_D08G1894 | CYP76B6 | *Gh_CYP76B6_10* | + | 1,506 | 446 | 51.116 | 11.5 | 8.268 | -0.128 | 1,341 | 1,341 | 41.9 | 3 | 447 | 82.5 | nucl |
| Gh_A08G2396 | CYP76B10 | *Gh_CYP76B10_11* | + | 1,324 | 415 | 47.451 | 1.5 | 6.743 | -0.137 | 1,248 | 1,248 | 40.5 | 2 | 624 | 76 | nucl |
| Gh_D08G1895 | CYP76B6 | *Gh_CYP76B6_12* | + | 1,135 | 329 | 37.235 | 4 | 7.738 | -0.138 | 990 | 990 | 40.8 | 2 | 495 | 145 | nucl |
| Gh_A08G1585 | CYP76B6 | *Gh_CYP76B6_13* | + | 1,333 | 414 | 47.152 | -2.5 | 6.201 | -0.177 | 1,245 | 1,245 | 39.9 | 2 | 622.5 | 88 | nucl |
| Gh_A08G2394 | CYP76B6 | *Gh_CYP76B6_14* | + | 606 | 201 | 22.59 | -3 | 5.61 | -0.023 | 606 | 606 | 40.8 | 1 | 606 | No intron | nucl |
| Gh_D08G1893 | CYP76B6 | *Gh_CYP76B6_15* | + | 1,341 | 415 | 47.412 | -1 | 6.4 | -0.139 | 1,248 | 1,248 | 40.4 | 2 | 624 | 93 | E.R |
| Gh_D08G1896 | CYP76B6 | *Gh_CYP76B6_16* | + | 859 | 232 | 26.396 | 3.5 | 7.326 | -0.142 | 699 | 699 | 40.3 | 2 | 349.5 | 160 | Extr |
| Gh_A09G1731 | CYP76B6 | *Gh_CYP76B6_17* | - | 522 | 173 | 19.492 | 13 | 10.483 | -0.045 | 522 | 522 | 45.6 | 1 | 522 | No intron | nucl |
| Gh_A09G1730 | CYP76B10 | *Gh_CYP76B10_18* | - | 1,017 | 156 | 18.051 | -0.5 | 6.272 | -0.399 | 471 | 471 | 35.9 | 2 | 235.5 | 546 | nucl |
| Gh_D09G1843 | CYP76B6 | *Gh_CYP76B6_19* | - | 1,578 | 348 | 39.273 | 9.5 | 9.416 | -0.177 | 1,047 | 1,047 | 41.5 | 2 | 523.5 | 531 | nucl |
| Gh_A09G1729 | CYP76B6 | *Gh_CYP76B6_20* | - | 1,889 | 485 | 54.572 | 9.5 | 9.245 | -0.095 | 1,458 | 1,458 | 42.9 | 2 | 729 | 431 | nucl |
| Gh_D09G1842 | CYP76B6 | *Gh_CYP76B6_21* | - | 11,513 | 500 | 56.18 | 8 | 8.367 | -0.055 | 1,503 | 1,503 | 42.6 | 2 | 751.5 | 10,010.00 | nucl |
| Gh_A09G1734 | CYP76B6 | *Gh_CYP76B6_22* | - | 872 | 226 | 25.352 | -4 | 5.138 | -0.061 | 681 | 681 | 41.1 | 2 | 340.5 | 191 | mito |
| Gh_A09G1733 | CYP76B10 | *Gh_CYP76B10_23* | - | 968 | 258 | 28.829 | 0 | 6.514 | -0.015 | 777 | 777 | 40.9 | 2 | 388.5 | 191 | nucl |
| Gh_D09G2441 | CYP76A2 | *Gh_CYP76A2_24* | - | 462 | 153 | 17.613 | 18 | 10.797 | -0.272 | 462 | 462 | 45.9 | 1 | 462 | No intron | vacu |
| Gh_D11G3255 | CYP76A2 | *Gh_CYP76A2_25* | - | 1,946 | 513 | 58.417 | 18 | 9.02 | -0.167 | 1,542 | 1,542 | 44.9 | 2 | 771 | 404 | nucl |
| Gh_A11G2871 | CYP76A2 | *Gh_CYP76A2_26* | - | 1,967 | 513 | 58.403 | 17.5 | 9.144 | -0.188 | 1,542 | 1,542 | 45.7 | 2 | 771 | 425 | nucl |
| Gh_D09G2440 | CYP76A2 | *Gh_CYP76A2_27* | - | 1,242 | 353 | 40.153 | -7 | 5.105 | -0.21 | 1,062 | 1,062 | 45 | 2 | 531 | 180 | nucl |
| Gh_D04G1016 | CYP76A2 | *Gh_CYP76A2_28* | - | 1,615 | 512 | 58.374 | 11.5 | 8.926 | -0.128 | 1,539 | 1,539 | 44.7 | 2 | 769.5 | 76 | E.R |
| Gh_A04G0558 | CYP76A2 | *Gh_CYP76A2_29* | - | 53,208 | 485 | 55.258 | 12.5 | 9.236 | -0.247 | 1,458 | 1,458 | 45.7 | 3 | 486 | 25,875.00 | plas |
| Gh_A05G0557 | CYP75A1 | *Gh_CYP75A1_30* | - | 1,736 | 527 | 59.542 | 14 | 9.313 | -0.078 | 1,584 | 1,584 | 47 | 3 | 528 | 76 | nucl |
| Gh_D05G0686 | CYP75A1 | *Gh_CYP75A1_31* | - | 1,736 | 527 | 59.529 | 15 | 9.339 | -0.069 | 1,584 | 1,584 | 47.3 | 3 | 528 | 76 | nucl |
| Gh_A08G1561 | CYP75B2 | *Gh_CYP75B2_32* | + | 1,224 | 407 | 46.427 | 8.5 | 8.436 | -0.181 | 1,224 | 1,224 | 41.9 | 1 | 1,224.00 | No intron | E.R |
| Gh_D08G1867 | CYP75A1 | *Gh_CYP75A1_33* | + | 1,628 | 502 | 56.85 | 15 | 9.362 | -0.067 | 1,509 | 1,509 | 43.8 | 2 | 754.5 | 119 | plas |
| Gh_A08G1560 | CYP73A12 | *Gh_CYP73A12_34* | + | 363 | 120 | 13.077 | 7.5 | 10.302 | 0.504 | 363 | 363 | 45.5 | 1 | 363 | No intron | Extr |
| Gh_D03G1513 | CYP93A2 | *Gh_CYP93A2_35* | + | 1,703 | 536 | 60.137 | 4.5 | 7.552 | -0.103 | 1,611 | 1,611 | 43.7 | 2 | 805.5 | 92 | nucl |
| Gh_A03G2006 | CYP75B1 | *Gh_CYP75B1_36* | + | 1,703 | 536 | 60.133 | 4 | 7.524 | -0.118 | 1,611 | 1,611 | 43.8 | 2 | 805.5 | 92 | nucl |
| Gh_A03G1994 | CYP736A12 | *Gh_CYP736A12_37* | - | 7,912 | 365 | 40.839 | -0.5 | 6.384 | -0.059 | 1,098 | 1,098 | 42.2 | 3 | 366 | 3,407.00 | nucl |
| Gh_D03G1505 | CYP75A2 | *Gh_CYP75A2_38* | + | 2,764 | 532 | 60.112 | 6.5 | 7.837 | -0.108 | 1,599 | 1,599 | 42 | 2 | 799.5 | 1,165.00 | nucl |
| Gh_D03G1504 | CYP75A2 | *Gh_CYP75A2_39* | + | 2,969 | 532 | 60.026 | 10 | 8.542 | -0.09 | 1,599 | 1,599 | 42.2 | 2 | 799.5 | 1,370.00 | nucl |
| Gh_D03G1507 | CYP75A2 | *Gh_CYP75A2_40* | + | 2,990 | 532 | 60.13 | 10 | 8.543 | -0.074 | 1,599 | 1,599 | 42.3 | 2 | 799.5 | 1,391.00 | nucl |
| Gh_D03G1510 | CYP75A2 | *Gh_CYP75A2_41* | - | 1,664 | 527 | 59.583 | 5 | 7.654 | -0.084 | 1,584 | 1,584 | 42.1 | 2 | 792 | 80 | nucl |
| Gh_D03G1506 | CYP76B1 | *Gh_CYP76B1_42* | - | 2,208 | 325 | 36.55 | -2.5 | 5.459 | 0.093 | 978 | 978 | 42.6 | 2 | 489 | 1,230.00 | nucl |
| Gh_D03G1512 | CYP71Z7 | *Gh_CYP71Z7_43* | - | 546 | 181 | 20.031 | 3.5 | 8.934 | 0.445 | 546 | 546 | 45.4 | 1 | 546 | No intron | nucl |
| Gh_Sca056974G01 | CYP71D6 | *Gh_CYP71D6_44* | - | 402 | 133 | 14.596 | 0.5 | 6.79 | 0.376 | 402 | 402 | 45.5 | 1 | 402 | No intron | nucl |
| Gh_D03G1509 | CYP75B2 | *Gh_CYP75B2_45* | + | 839 | 252 | 28.765 | 2.5 | 7.216 | -0.264 | 759 | 759 | 42 | 2 | 379.5 | 80 | nucl |
| Gh_D03G1508 | CYP76AH1 | *Gh_CYP76AH1_46* | + | 783 | 260 | 28.795 | 4 | 8.77 | 0.183 | 783 | 783 | 46 | 1 | 783 | No intron | nucl |
| Gh_A03G1995 | CYP75A2 | *Gh_CYP75A2_47* | - | 1,526 | 481 | 54.225 | 5.5 | 7.919 | -0.095 | 1,446 | 1,446 | 43.2 | 2 | 723 | 80 | nucl |
| Gh_A03G1993 | CYP75A2 | *Gh_CYP75A2_48* | - | 912 | 303 | 33.956 | 3.5 | 8.428 | 0.071 | 912 | 912 | 44.2 | 1 | 912 | No intron | nucl |
| Gh_D12G2194 | CYP93A2 | *Gh_CYP93A2_49* | + | 3,457 | 502 | 56.183 | 0.5 | 6.61 | -0.045 | 1,509 | 1,509 | 46.2 | 2 | 754.5 | 1,948.00 | E.R |
| Gh_A12G2016 | CYP93A1 | *Gh_CYP93A1_50* | + | 1,988 | 502 | 56.344 | 5 | 7.704 | -0.045 | 1,509 | 1,509 | 46.3 | 2 | 754.5 | 479 | E.R |
| Gh_D10G2213 | CYP93A2 | *Gh_CYP93A2_51* | + | 2,877 | 533 | 60.082 | 13 | 9.311 | -0.021 | 1,602 | 1,602 | 46.8 | 2 | 801 | 1,275.00 | E.R |
| Gh_A10G2357 | CYP93A2 | *Gh_CYP93A2_52* | - | 2,913 | 529 | 59.866 | 17.5 | 9.832 | -0.065 | 1,590 | 1,590 | 46.4 | 2 | 795 | 1,323.00 | E.R |
| Gh_D12G2525 | CYP93A2 | *Gh_CYP93A2_53* | + | 3,121 | 524 | 59.525 | 15 | 9.685 | -0.043 | 1,575 | 1,575 | 45 | 2 | 787.5 | 1,546.00 | nucl |
| Gh_A12G2399 | CYP76B6 | *Gh_CYP76B6_54* | + | 3,437 | 540 | 61.903 | 28 | 10.087 | -0.164 | 1,623 | 1,623 | 45 | 3 | 541 | 907 | nucl |
| Gh_A12G2396 | CYP76AH1 | *Gh_CYP76AH1_55* | + | 3,093 | 530 | 60.495 | 12 | 8.991 | -0.027 | 1,593 | 1,593 | 44.5 | 2 | 796.5 | 1,500.00 | nucl |
| Gh_A12G2398 | CYP76C3 | *Gh_CYP76C3_56* | + | 1,691 | 242 | 27.688 | -1.5 | 6.042 | 0 | 729 | 729 | 42.9 | 2 | 364.5 | 962 | nucl |
| Gh_D12G2523 | CYP76C3 | *Gh_CYP76C3_57* | + | 2,734 | 531 | 60.533 | 9.5 | 8.957 | -0.023 | 1,596 | 1,596 | 45.2 | 2 | 798 | 1,138.00 | E.R |
| Gh_A12G2397 | CYP76C3 | *Gh_CYP76C3_58* | + | 2,747 | 531 | 60.48 | 13 | 9.48 | -0.06 | 1,596 | 1,596 | 45.1 | 2 | 798 | 1,151.00 | nucl |
| Gh_D12G2524 | CYP76C3 | *Gh_CYP76C3_59* | + | 2,548 | 531 | 60.569 | 9 | 8.702 | -0.063 | 1,596 | 1,596 | 45.2 | 2 | 798 | 952 | nucl |
| Gh_D12G2522 | CYP76C3 | *Gh_CYP76C3_60* | + | 1,951 | 338 | 39.053 | -4.5 | 5.32 | -0.111 | 1,017 | 1,017 | 42.2 | 2 | 508.5 | 934 | nucl |
| Gh_A11G0069 | CYP736A12 | *Gh_CYP736A12_61* | - | 1,352 | 425 | 47.963 | 5 | 7.619 | -0.065 | 1,278 | 1,278 | 43.6 | 2 | 639 | 74 | nucl |
| Gh_A11G0067 | CYP76C3 | *Gh_CYP76C3_62* | - | 12,997 | 785 | 89.615 | 11 | 8.195 | -0.07 | 2,358 | 2,358 | 43.5 | 6 | 393 | 2,127.80 | E.R |
| Gh_A04G0165 | CYP75B1 | *Gh_CYP75B1_63* | + | 1,675 | 531 | 60.056 | 12.5 | 8.796 | -0.041 | 1,596 | 1,596 | 45.1 | 2 | 798 | 79 | E.R |
| Gh_A04G0163 | CYP93A1 | *Gh_CYP93A1_64* | + | 39,830 | 388 | 43.775 | 11 | 8.927 | 0.007 | 1,167 | 1,167 | 45.2 | 3 | 389 | 19,331.50 | E.R |
| Gh_D05G3536 | CYP76B6 | *Gh_CYP76B6_65* | - | 1,647 | 522 | 59.134 | 4 | 7.238 | -0.032 | 1,569 | 1,569 | 43.8 | 2 | 784.5 | 78 | E.R |
| Gh_D05G3537 | CYP76C3 | *Gh_CYP76C3_66* | - | 1,177 | 365 | 41.656 | -1.5 | 6.194 | -0.175 | 1,098 | 1,098 | 43.5 | 2 | 549 | 79 | nucl |
| Gh_D05G3540 | CYP76C3 | *Gh_CYP76C3_67* | - | 1,177 | 365 | 41.617 | 0.5 | 6.638 | -0.177 | 1,098 | 1,098 | 42.7 | 2 | 549 | 79 | nucl |
| Gh_D05G3736 | CYP76A2 | *Gh_CYP76A2_68* | + | 1,648 | 440 | 49.7 | 5 | 7.525 | -0.025 | 1,323 | 1,323 | 44.6 | 3 | 441 | 162.5 | E.R |
| Gh_A11G0068 | CYP80B3 | *Gh_CYP80B3_69* | - | 456 | 151 | 17.443 | 4.5 | 8.7 | -0.325 | 456 | 456 | 43.9 | 1 | 456 | No intron | vacu |
| Gh_A07G1098 | CYP75A3 | *Gh_CYP75A3_70* | + | 447 | 148 | 16.491 | 11.5 | 10.683 | 0.092 | 447 | 447 | 48.8 | 1 | 447 | No intron | nucl |
| Gh_D07G1197 | CYP75A3 | *Gh_CYP75A3_71* | + | 1,662 | 510 | 57.201 | 13.5 | 9.356 | -0.108 | 1,533 | 1,533 | 49.4 | 2 | 766.5 | 129 | plas |
| Gh_D12G1798 | CYP75B2 | *Gh_CYP75B2_72* | + | 4,029 | 510 | 56.804 | 14 | 9.489 | -0.096 | 1,533 | 1,533 | 52 | 3 | 511 | 1,248.00 | plas |
| Gh_A12G2650 | CYP75B2 | *Gh_CYP75B2_73* | + | 4,375 | 510 | 56.784 | 13 | 9.176 | -0.091 | 1,533 | 1,533 | 51.5 | 3 | 511 | 1,421.00 | plas |
| Gh_A01G1660 | CYP93A1 | *Gh_CYP93A1_74* | + | 2,085 | 515 | 58.55 | 7.5 | 7.905 | -0.182 | 1,548 | 1,548 | 43.2 | 2 | 774 | 537 | mito |
| Gh_Sca130916G01 | CYP71B29 | *Gh_CYP71B29_75* | + | 219 | 73 | 8.356 | 0.5 | 7.258 | -0.632 | 219 | 219 | 46.1 | 1 | 219 | No intron | nucl |
| Gh_D01G1909 | CYP93A1 | *Gh_CYP93A1_76* | + | 2,078 | 514 | 58.407 | 6.5 | 7.751 | -0.147 | 1,545 | 1,545 | 43.1 | 2 | 772.5 | 533 | nucl |
| Gh_A09G1178 | CYP93A1 | *Gh_CYP93A1_77* | + | 1,653 | 508 | 58.007 | 14 | 8.916 | -0.163 | 1,527 | 1,527 | 44.5 | 2 | 763.5 | 126 | nucl |
| Gh_D09G1185 | CYP93E1 | *Gh_CYP93E1_78* | + | 1,653 | 508 | 58.157 | 15 | 9.043 | -0.198 | 1,527 | 1,527 | 44.4 | 2 | 763.5 | 126 | nucl |
| Gh_A09G1179 | CYP93A1 | *Gh_CYP93A1_79* | + | 29,939 | 453 | 51.112 | 15.5 | 9.457 | -0.21 | 1,362 | 1,362 | 43.6 | 3 | 454 | 14,288.50 | nucl |
| Gh_D09G1186 | CYP93A1 | *Gh_CYP93A1_80* | + | 1,660 | 512 | 58.254 | 15.5 | 9.119 | -0.264 | 1,539 | 1,539 | 42.9 | 2 | 769.5 | 121 | E.R |
| Gh_A09G1774 | CYP93B1 | *Gh_CYP93B1_81* | - | 435 | 144 | 15.812 | 2.5 | 8.325 | -0.012 | 435 | 435 | 49 | 1 | 435 | No intron | nucl |
| Gh_D09G1898 | CYP93B1 | *Gh_CYP93B1_82* | - | 1,551 | 316 | 35.648 | 8 | 8.155 | -0.164 | 951 | 951 | 47.8 | 4 | 237.8 | 200 | E.R |
| Gh_A09G1775 | CYP93B1 | *Gh_CYP93B1_83* | - | 1,050 | 317 | 36.649 | 10.5 | 9.058 | -0.004 | 954 | 954 | 44.1 | 2 | 477 | 96 | E.R |
| Gh_D13G1277 | CYP93B1 | *Gh_CYP93B1_84* | - | 2,277 | 519 | 58.968 | 7 | 7.443 | -0.071 | 1,560 | 1,560 | 45.1 | 2 | 780 | 717 | E.R |
| Gh_A13G1025 | CYP93B1 | *Gh_CYP93B1_85* | - | 1,123 | 326 | 37.468 | 7.5 | 8.048 | 0.012 | 981 | 981 | 43.6 | 2 | 490.5 | 142 | E.R |
| Gh_A13G1007 | CYP93B1 | *Gh_CYP93B1_86* | + | 2,303 | 532 | 60.464 | 7.5 | 7.417 | -0.078 | 1,599 | 1,599 | 44.2 | 2 | 799.5 | 704 | E.R |
| Gh_A01G1658 | CYP93A3 | *Gh_CYP93A3_87* | - | 2,163 | 505 | 57.498 | 6 | 7.355 | -0.127 | 1,518 | 1,518 | 41.8 | 2 | 759 | 645 | E.R |
| Gh_D01G1907 | CYP93A3 | *Gh_CYP93A3_88* | - | 2,153 | 505 | 57.605 | 3 | 6.873 | -0.138 | 1,518 | 1,518 | 41.4 | 2 | 759 | 635 | nucl |
| Gh_Sca154986G01 | CYP93A2 | *Gh_CYP93A2_89* | + | 198 | 66 | 7.881 | -0.5 | 5.881 | -0.926 | 198 | 198 | 32.8 | 1 | 198 | No intron | nucl |
| Gh_A01G1659 | CYP93A1 | *Gh_CYP93A1_90* | - | 1,624 | 515 | 58.534 | 6 | 7.683 | -0.257 | 1,548 | 1,548 | 42.4 | 2 | 774 | 76 | E.R |
| Gh_D01G1908 | CYP93A1 | *Gh_CYP93A1_91* | - | 1,624 | 515 | 58.539 | 7.5 | 7.89 | -0.262 | 1,548 | 1,548 | 42.2 | 2 | 774 | 76 | nucl |
| Gh_A09G1181 | CYP93A1 | *Gh_CYP93A1_92* | + | 1,719 | 517 | 58.773 | 8.5 | 8.21 | -0.184 | 1,554 | 1,554 | 42.5 | 2 | 777 | 165 | E.R |
| Gh_D09G1187 | CYP93A1 | *Gh_CYP93A1_93* | + | 1,735 | 517 | 58.855 | 6.5 | 7.796 | -0.192 | 1,554 | 1,554 | 42.7 | 2 | 777 | 181 | E.R |
| Gh_D09G1968 | CYP736A12 | *Gh_CYP736A12_94* | - | 1,573 | 499 | 56.962 | 7.5 | 7.594 | -0.112 | 1,500 | 1,500 | 44.7 | 2 | 750 | 73 | nucl |
| Gh_A09G1848 | CYP736A12 | *Gh_CYP736A12_95* | - | 1,573 | 499 | 56.876 | 8.5 | 7.77 | -0.09 | 1,500 | 1,500 | 44.6 | 2 | 750 | 73 | nucl |
| Gh_D09G1969 | CYP736A12 | *Gh_CYP736A12_96* | - | 1,573 | 499 | 56.854 | 6 | 7.382 | -0.148 | 1,500 | 1,500 | 45.3 | 2 | 750 | 73 | nucl |
| Gh_A09G1849 | CYP736A12 | *Gh_CYP736A12_97* | - | 1,573 | 499 | 56.843 | 5 | 7.196 | -0.162 | 1,500 | 1,500 | 45.5 | 2 | 750 | 73 | nucl |
| Gh_A05G2516 | CYP736A12 | *Gh_CYP736A12_98* | - | 1,128 | 340 | 38.546 | -8.5 | 4.974 | -0.063 | 1,023 | 1,023 | 39.7 | 2 | 511.5 | 105 | nucl |
| Gh_A05G2517 | CYP736A12 | *Gh_CYP736A12_99* | - | 345 | 114 | 12.399 | 10.5 | 10.639 | 0.421 | 345 | 345 | 44.3 | 1 | 345 | No intron | chlo |
| Gh_A05G2518 | CYP736A12 | *Gh_CYP736A12_100* | - | 3,137 | 502 | 56.576 | 10.5 | 8.525 | -0.008 | 1,509 | 1,509 | 39.8 | 2 | 754.5 | 1,628.00 | nucl |
| Gh_D05G2795 | CYP736A12 | *Gh_CYP736A12_101* | - | 1,611 | 502 | 56.655 | 13.5 | 9.16 | -0.014 | 1,509 | 1,509 | 39.6 | 2 | 754.5 | 102 | nucl |
| Gh_D09G0471 | CYP736A12 | *Gh_CYP736A12_102* | + | 61,351 | 320 | 36.561 | -6.5 | 5.157 | -0.2 | 963 | 963 | 40.6 | 3 | 321 | 30,194.00 | nucl |
| Gh_A09G0463 | CYP736A12 | *Gh_CYP736A12_103* | + | 1,587 | 493 | 55.824 | 11 | 8.663 | -0.096 | 1,482 | 1,482 | 39.7 | 2 | 741 | 105 | nucl |
| Gh_A09G2498 | CYP736A12 | *Gh_CYP736A12_104* | - | 1,599 | 497 | 55.728 | 9.5 | 8.357 | 0.01 | 1,494 | 1,494 | 42.4 | 2 | 747 | 105 | nucl |
| Gh_D09G0472 | CYP736A12 | *Gh_CYP736A12_105* | + | 67,703 | 1,103 | 125.23 | 40 | 9.634 | -0.135 | 3,312 | 3,312 | 40.2 | 5 | 662.4 | 16,097.80 | nucl |
| Gh_A09G0164 | CYP736A12 | *Gh_CYP736A12_106* | - | 3,282 | 480 | 54.796 | 10.5 | 8.589 | -0.142 | 1,443 | 1,443 | 42.2 | 3 | 481 | 919.5 | nucl |
| Gh_D09G0470 | CYP736A12 | *Gh_CYP736A12_107* | + | 1,637 | 513 | 58.028 | 13.5 | 8.56 | -0.063 | 1,542 | 1,542 | 42.8 | 2 | 771 | 95 | E.R |
| Gh_A01G0481 | CYP736A12 | *Gh_CYP736A12_108* | - | 1,051 | 264 | 29.451 | 13.5 | 9.903 | 0.158 | 795 | 795 | 46 | 2 | 397.5 | 256 | plas |
| Gh_D01G0496 | CYP736A12 | *Gh_CYP736A12_109* | - | 52,781 | 519 | 58.778 | 9.5 | 8.338 | 0.016 | 1,560 | 1,560 | 45.9 | 2 | 780 | 51,221.00 | plas |
| Gh_D01G0495 | CYP736A12 | *Gh_CYP736A12_110* | - | 1,826 | 519 | 58.709 | 8 | 7.908 | -0.058 | 1,560 | 1,560 | 46.4 | 2 | 780 | 266 | plas |
| Gh_Sca006093G01 | CYP736A12 | *Gh_CYP736A12_111* | + | 1,233 | 243 | 28.031 | -4 | 5.362 | -0.218 | 732 | 732 | 43.3 | 3 | 244 | 250.5 | E.R |
| Gh_A01G0480 | CYP736A12 | *Gh_CYP736A12_112* | - | 315 | 104 | 12.043 | 3.5 | 8.784 | -0.313 | 315 | 315 | 45.4 | 1 | 315 | No intron | nucl |
| Gh_A13G0217 | CYP736A12 | *Gh_CYP736A12_113* | - | 2,656 | 507 | 57.742 | 13.5 | 8.535 | -0.111 | 1,524 | 1,524 | 44.5 | 2 | 762 | 1,132.00 | plas |
| Gh_D13G0230 | CYP736A12 | *Gh_CYP736A12_114* | - | 3,973 | 438 | 49.925 | 3.5 | 6.998 | -0.105 | 1,317 | 1,317 | 44.8 | 2 | 658.5 | 2,656.00 | E.R |
| Gh_A13G2079 | CYP736A12 | *Gh_CYP736A12_115* | - | 480 | 159 | 18.383 | 1.5 | 7.058 | -0.242 | 480 | 480 | 42.1 | 1 | 480 | No intron | nucl |
| Gh_A13G0215 | CYP736A12 | *Gh_CYP736A12_116* | - | 3,866 | 506 | 57.518 | 13.5 | 8.702 | -0.112 | 1,521 | 1,521 | 44.8 | 2 | 760.5 | 2,345.00 | plas |
| Gh_A13G2080 | CYP736A12 | *Gh_CYP736A12_117* | - | 3,785 | 329 | 37.372 | 14.5 | 9.404 | -0.057 | 990 | 990 | 44.8 | 3 | 330 | 1,397.50 | plas |
| Gh_Sca060631G01 | CYP84A1 | *Gh_CYP84A1_118* | - | 523 | 63 | 6.928 | -4 | 4.153 | -0.1 | 189 | 189 | 44.4 | 2 | 94.5 | 334 | mito |
| Gh_D13G0298 | CYP84A1 | *Gh_CYP84A1_119* | - | 2,484 | 544 | 61.104 | 0.5 | 6.579 | -0.147 | 1,635 | 1,635 | 49.1 | 2 | 817.5 | 849 | nucl |
| Gh_A13G0279 | CYP84A1 | *Gh_CYP84A1_120* | - | 2,484 | 544 | 61.206 | 2.5 | 6.843 | -0.162 | 1,635 | 1,635 | 48.6 | 2 | 817.5 | 849 | plas |
| Gh_A11G1648 | CYP84A1 | *Gh_CYP84A1_121* | - | 1,779 | 515 | 58.451 | 0 | 6.524 | -0.177 | 1,548 | 1,548 | 49.9 | 2 | 774 | 231 | E.R |
| Gh_Sca170850G01 | CYP84A1 | *Gh_CYP84A1_122* | + | 189 | 63 | 7.143 | 0 | 6.576 | -0.483 | 189 | 189 | 54 | 1 | 189 | No intron | mito |
| Gh_D11G1805 | CYP84A1 | *Gh_CYP84A1_123* | - | 1,778 | 515 | 58.498 | -2 | 6.21 | -0.178 | 1,548 | 1,548 | 50.3 | 2 | 774 | 230 | E.R |
| Gh_A10G0875 | CYP84A1 | *Gh_CYP84A1_124* | + | 1,527 | 508 | 58.027 | 9 | 8.453 | -0.177 | 1,527 | 1,527 | 48.3 | 1 | 1,527.00 | No intron | nucl |
| Gh_D10G0878 | CYP84A1 | *Gh_CYP84A1_125* | - | 1,527 | 508 | 57.706 | 7.5 | 8.264 | -0.169 | 1,527 | 1,527 | 48.3 | 1 | 1,527.00 | No intron | nucl |
| Gh_Sca004990G01 | CYP84A1 | *Gh_CYP84A1_126* | - | 2,032 | 517 | 58.407 | 3 | 7.052 | -0.133 | 1,554 | 1,554 | 48.3 | 2 | 777 | 478 | nucl |
| Gh_A11G3018 | CYP84A1 | *Gh_CYP84A1_127* | + | 2,046 | 517 | 58.458 | 2 | 6.854 | -0.135 | 1,554 | 1,554 | 47.9 | 2 | 777 | 492 | E.R |
| Gh_D01G0713 | CYP71D8 | *Gh_CYP71D8_128* | - | 1,633 | 502 | 56.947 | 8 | 8.26 | -0.049 | 1,509 | 1,509 | 42.3 | 2 | 754.5 | 124 | nucl |
| Gh_A01G0696 | CYP71D11 | *Gh_CYP71D11_129* | - | 1,633 | 502 | 57.115 | 10 | 8.443 | -0.048 | 1,509 | 1,509 | 41.6 | 2 | 754.5 | 124 | nucl |
| Gh_A13G1031 | CYP71D10 | *Gh_CYP71D10_130* | + | 1,603 | 508 | 57.645 | 8 | 7.825 | -0.11 | 1,527 | 1,527 | 42.6 | 2 | 763.5 | 76 | nucl |
| Gh_D01G0717 | CYP71D55 | *Gh_CYP71D55_131* | - | 1,608 | 495 | 56.554 | 8.5 | 7.922 | -0.188 | 1,488 | 1,488 | 42.1 | 3 | 496 | 60 | nucl |
| Gh_A01G0699 | CYP71D55 | *Gh_CYP71D55_132* | - | 14,032 | 481 | 55.135 | 4 | 6.966 | -0.175 | 1,446 | 1,446 | 41.8 | 3 | 482 | 6,293.00 | E.R |
| Gh_A01G0697 | CYP71D10 | *Gh_CYP71D10_133* | - | 1,813 | 530 | 60.377 | 5 | 7.021 | -0.122 | 1,593 | 1,593 | 40.1 | 2 | 796.5 | 220 | nucl |
| Gh_D01G0712 | CYP71D55 | *Gh_CYP71D55_134* | - | 1,747 | 508 | 57.963 | 3.5 | 6.826 | -0.139 | 1,527 | 1,527 | 41.5 | 2 | 763.5 | 220 | E.R |
| Gh_A01G0694 | CYP71D55 | *Gh_CYP71D55_135* | - | 1,744 | 507 | 57.884 | 6 | 7.148 | -0.141 | 1,524 | 1,524 | 41.3 | 2 | 762 | 220 | E.R |
| Gh_D01G0714 | CYP71D10 | *Gh_CYP71D10_136* | - | 61,052 | 324 | 36.844 | 6.5 | 7.725 | -0.205 | 975 | 975 | 43.9 | 3 | 325 | 30,038.50 | nucl |
| Gh_A01G0698 | CYP71D10 | *Gh_CYP71D10_137* | - | 1,052 | 242 | 27.576 | -5.5 | 5.187 | -0.284 | 729 | 729 | 40.7 | 2 | 364.5 | 323 | nucl |
| Gh_D01G0716 | CYP71AV8 | *Gh_CYP71AV8_138* | + | 576 | 191 | 21.901 | 14.5 | 10.551 | 0.214 | 576 | 576 | 39.6 | 1 | 576 | No intron | chlo |
| Gh_D01G0715 | CYP71D10 | *Gh_CYP71D10_139* | + | 1,307 | 299 | 34.164 | -2 | 6.111 | -0.208 | 900 | 900 | 40 | 2 | 450 | 407 | nucl |
| Gh_D01G0710 | CYP71D8 | *Gh_CYP71D8_140* | - | 25,046 | 517 | 58.241 | 10 | 8.453 | -0.11 | 1,554 | 1,554 | 41.1 | 3 | 518 | 11,746.00 | nucl |
| Gh_A01G0690 | CYP71D8 | *Gh_CYP71D8_141* | - | 1,608 | 511 | 57.515 | 8 | 8.076 | -0.12 | 1,536 | 1,536 | 41.1 | 2 | 768 | 72 | nucl |
| Gh_A02G0050 | CYP71D10 | *Gh_CYP71D10_142* | + | 1,405 | 413 | 46.48 | -7 | 5.367 | -0.144 | 1,242 | 1,242 | 41.5 | 3 | 414 | 81.5 | nucl |
| Gh_D02G0063 | CYP71D10 | *Gh_CYP71D10_143* | + | 1,576 | 498 | 56.297 | 10 | 8.346 | -0.072 | 1,497 | 1,497 | 41.8 | 2 | 748.5 | 79 | E.R |
| Gh_D13G1649 | CYP71D55 | *Gh_CYP71D55_144* | - | 1,195 | 365 | 41.174 | 10.5 | 9.424 | -0.208 | 1,098 | 1,098 | 40 | 2 | 549 | 97 | nucl |
| Gh_A13G1341 | CYP71D9 | *Gh_CYP71D9_145* | - | 1,405 | 306 | 34.719 | -3.5 | 5.691 | -0.192 | 921 | 921 | 38.1 | 4 | 230.3 | 161.3 | nucl |
| Gh_D13G1650 | CYP71D10 | *Gh_CYP71D10_146* | + | 1,301 | 386 | 43.552 | 4 | 7.887 | -0.235 | 1,161 | 1,161 | 40.4 | 2 | 580.5 | 140 | nucl |
| Gh_D07G2094 | CYP71D10 | *Gh_CYP71D10_147* | + | 1,598 | 505 | 56.847 | 2 | 6.854 | -0.111 | 1,518 | 1,518 | 40.3 | 2 | 759 | 80 | mito |
| Gh_A07G1880 | CYP71D10 | *Gh_CYP71D10_148* | + | 1,610 | 509 | 57.383 | 6.5 | 7.638 | -0.071 | 1,530 | 1,530 | 40.7 | 2 | 765 | 80 | mito |
| Gh_D13G1407 | CYP71D10 | *Gh_CYP71D10_149* | + | 1,634 | 508 | 57.896 | 13 | 8.962 | -0.297 | 1,527 | 1,527 | 46.6 | 2 | 763.5 | 107 | plas |
| Gh_A13G1133 | CYP71D10 | *Gh_CYP71D10_150* | + | 1,631 | 508 | 57.944 | 14.5 | 9.121 | -0.312 | 1,527 | 1,527 | 46.3 | 2 | 763.5 | 104 | plas |
| Gh_A13G1123 | CYP71D10 | *Gh_CYP71D10_151* | + | 2,736 | 542 | 61.614 | 12.5 | 8.695 | 0.058 | 1,629 | 1,629 | 40.5 | 3 | 543 | 553.5 | nucl |
| Gh_D13G1394 | CYP71D10 | *Gh_CYP71D10_152* | + | 537 | 178 | 20.29 | 1.5 | 7.03 | -0.129 | 537 | 537 | 39.3 | 1 | 537 | No intron | nucl |
| Gh_D13G1405 | CYP71D10 | *Gh_CYP71D10_153* | - | 1,617 | 500 | 56.444 | 9.5 | 8.294 | -0.006 | 1,503 | 1,503 | 40.7 | 2 | 751.5 | 114 | nucl |
| Gh_A04G0401 | CYP71D12 | *Gh_CYP71D12_154* | - | 1,619 | 273 | 30.461 | 8 | 9.577 | -0.122 | 822 | 822 | 42.8 | 5 | 164.4 | 199.3 | nucl |
| Gh_A04G0400 | CYP71D10 | *Gh_CYP71D10_155* | - | 1,619 | 508 | 56.871 | 17 | 9.647 | -0.052 | 1,527 | 1,527 | 42 | 2 | 763.5 | 92 | nucl |
| Gh_D05G3234 | CYP71D10 | *Gh_CYP71D10_156* | + | 1,619 | 508 | 56.758 | 12.5 | 8.944 | -0.031 | 1,527 | 1,527 | 42 | 2 | 763.5 | 92 | E.R |
| Gh_D05G3228 | CYP71D10 | *Gh_CYP71D10_157* | + | 1,619 | 508 | 56.763 | 13 | 9.125 | -0.022 | 1,527 | 1,527 | 41.9 | 2 | 763.5 | 92 | E.R |
| Gh_D05G3230 | CYP71D10 | *Gh_CYP71D10_158* | + | 1,585 | 464 | 51.856 | 6 | 7.626 | -0.07 | 1,395 | 1,395 | 41.8 | 3 | 465 | 95 | E.R |
| Gh_D05G3235 | CYP71D9 | *Gh_CYP71D9_159* | + | 3,453 | 473 | 53.439 | 7 | 7.443 | -0.063 | 1,422 | 1,422 | 43.2 | 6 | 237 | 406.2 | nucl |
| Gh_Sca010971G01 | CYP71D10 | *Gh_CYP71D10_160* | + | 1,616 | 507 | 57.003 | 3.5 | 6.996 | -0.067 | 1,524 | 1,524 | 42.5 | 2 | 762 | 92 | nucl |
| Gh_A04G0398 | CYP71D10 | *Gh_CYP71D10_161* | - | 1,070 | 325 | 36.979 | 1.5 | 6.882 | -0.201 | 978 | 978 | 40.1 | 2 | 489 | 92 | nucl |
| Gh_A04G0399 | CYP71D10 | *Gh_CYP71D10_162* | - | 1,616 | 507 | 57.224 | 4.5 | 7.328 | -0.058 | 1,524 | 1,524 | 41.7 | 2 | 762 | 92 | nucl |
| Gh_A10G2124 | CYP71A1 | *Gh_CYP71A1_163* | + | 1,841 | 502 | 57.113 | 5 | 7.02 | -0.114 | 1,509 | 1,509 | 44.5 | 2 | 754.5 | 332 | vacu |
| Gh_D10G2445 | CYP71A9 | *Gh_CYP71A9_164* | + | 2,731 | 504 | 57.542 | 5.5 | 7.127 | -0.156 | 1,515 | 1,515 | 45 | 2 | 757.5 | 1,216.00 | vacu |
| Gh_A03G0667 | CYP71A1 | *Gh_CYP71A1_165* | + | 906 | 276 | 31.665 | -11 | 4.74 | -0.142 | 831 | 831 | 43.9 | 2 | 415.5 | 75 | nucl |
| Gh_D03G0963 | CYP71A1 | *Gh_CYP71A1_166* | + | 1,602 | 508 | 57.877 | 4.5 | 7.066 | -0.078 | 1,527 | 1,527 | 44.9 | 2 | 763.5 | 75 | E.R |
| Gh_A03G0666 | CYP71BL2 | *Gh_CYP71BL2_167* | + | 654 | 217 | 24.593 | 17 | 10.393 | 0.007 | 654 | 654 | 46.3 | 1 | 654 | No intron | Extr |
| Gh_A07G0491 | CYP71A9 | *Gh_CYP71A9_168* | - | 35,503 | 334 | 37.499 | 8.5 | 9.195 | -0.151 | 1,005 | 1,005 | 43.5 | 3 | 335 | 17,249.00 | nucl |
| Gh_D07G0559 | CYP71A9 | *Gh_CYP71A9_169* | - | 2,953 | 484 | 54.859 | 8 | 8.033 | -0.224 | 1,455 | 1,455 | 43.1 | 2 | 727.5 | 1,498.00 | E.R |
| Gh_A07G0489 | CYP71A9 | *Gh_CYP71A9_170* | - | 2,690 | 426 | 48.483 | 4 | 7.306 | -0.261 | 1,281 | 1,281 | 41.8 | 2 | 640.5 | 1,409.00 | E.R |
| Gh_A02G1507 | CYP83B1 | *Gh_CYP83B1_171* | + | 1,585 | 497 | 57.176 | 7 | 7.597 | -0.113 | 1,494 | 1,494 | 39.8 | 2 | 747 | 91 | cyto |
| Gh_D10G2258 | CYP83B1 | *Gh_CYP83B1_172* | + | 1,456 | 412 | 47.796 | 4 | 7.273 | -0.199 | 1,239 | 1,239 | 39.5 | 3 | 413 | 108.5 | E.R |
| Gh_D03G0206 | CYP83B1 | *Gh_CYP83B1_173* | - | 19,676 | 497 | 57.48 | 4 | 7.119 | -0.115 | 1,494 | 1,494 | 39.7 | 2 | 747 | 18,182.00 | mito |
| Gh_A02G1508 | CYP83B1 | *Gh_CYP83B1_174* | + | 6,595 | 508 | 58.764 | 10 | 8.033 | -0.158 | 1,527 | 1,527 | 40 | 3 | 509 | 2,534.00 | mito |
| Gh_A02G1509 | CYP83B1 | *Gh_CYP83B1_175* | + | 1,328 | 411 | 47.353 | 5 | 7.496 | -0.136 | 1,236 | 1,236 | 39.1 | 2 | 618 | 92 | nucl |
| Gh_D05G1794 | CYP71A1 | *Gh_CYP71A1_176* | + | 465 | 154 | 17.47 | 3.5 | 8.464 | -0.011 | 465 | 465 | 38.5 | 1 | 465 | No intron | nucl |
| Gh_A10G1727 | CYP83B1 | *Gh_CYP83B1_177* | + | 5,376 | 565 | 65.01 | 6 | 7.34 | -0.133 | 1,698 | 1,698 | 39.5 | 3 | 566 | 1,839.00 | E.R |
| Gh_D10G2555 | CYP83B1 | *Gh_CYP83B1_178* | - | 1,624 | 510 | 58.824 | 8.5 | 7.768 | -0.083 | 1,533 | 1,533 | 40 | 2 | 766.5 | 91 | E.R |
| Gh_A10G1724 | CYP83B1 | *Gh_CYP83B1_179* | - | 1,601 | 503 | 57.628 | 5.5 | 7.667 | -0.039 | 1,512 | 1,512 | 39 | 2 | 756 | 89 | E.R |
| Gh_D10G2001 | CYP83B1 | *Gh_CYP83B1_180* | - | 1,586 | 498 | 57.452 | 7 | 7.956 | -0.105 | 1,497 | 1,497 | 39.5 | 2 | 748.5 | 89 | E.R |
| Gh_A10G1725 | CYP71B34 | *Gh_CYP71B34_181* | - | 1,620 | 503 | 57.588 | 10.5 | 8.521 | -0.018 | 1,512 | 1,512 | 40.6 | 2 | 756 | 108 | E.R |
| Gh_D10G2003 | CYP71B36 | *Gh_CYP71B36_182* | - | 1,017 | 269 | 30.9 | 8.5 | 8.987 | -0.037 | 810 | 810 | 41.1 | 3 | 270 | 103.5 | nucl |
| Gh_A10G1726 | CYP83B1 | *Gh_CYP83B1_183* | + | 9,966 | 997 | 113.911 | 17 | 7.924 | 0.028 | 2,994 | 2,994 | 41.1 | 4 | 748.5 | 2,324.00 | nucl |
| Gh_D10G2556 | CYP83B1 | *Gh_CYP83B1_184* | - | 1,058 | 325 | 37.163 | -3 | 5.5 | -0.075 | 978 | 978 | 40.4 | 2 | 489 | 80 | nucl |
| Gh_A10G1723 | CYP83B1 | *Gh_CYP83B1_185* | - | 1,598 | 503 | 57.552 | 10.5 | 8.389 | -0.016 | 1,512 | 1,512 | 41.4 | 2 | 756 | 86 | E.R |
| Gh_A10G1722 | CYP83B1 | *Gh_CYP83B1_186* | - | 1,602 | 504 | 57.402 | 10.5 | 8.401 | 0.014 | 1,515 | 1,515 | 40.9 | 2 | 757.5 | 87 | E.R |
| Gh_D10G2000 | CYP83B1 | *Gh_CYP83B1_187* | - | 1,605 | 505 | 57.611 | 7.5 | 8.007 | -0.007 | 1,518 | 1,518 | 40.9 | 2 | 759 | 87 | cyto |
| Gh_D11G2164 | CYP71E7 | *Gh_CYP71E7_188* | + | 856 | 252 | 29.073 | -1 | 6.156 | -0.308 | 759 | 759 | 42.3 | 2 | 379.5 | 97 | nucl |
| Gh_D02G1591 | CYP71B36 | *Gh_CYP71B36_189* | - | 1,339 | 415 | 47.371 | -3 | 5.924 | -0.156 | 1,248 | 1,248 | 40.8 | 2 | 624 | 91 | nucl |
| Gh_A12G1342 | CYP71B36 | *Gh_CYP71B36_190* | + | 5,613 | 514 | 58.426 | 12 | 8.282 | -0.051 | 1,545 | 1,545 | 46 | 2 | 772.5 | 4,068.00 | nucl |
| Gh_D12G1468 | CYP71B36 | *Gh_CYP71B36_191* | + | 12,730 | 515 | 58.446 | 12 | 8.283 | -0.044 | 1,548 | 1,548 | 46.1 | 2 | 774 | 11,182.00 | nucl |
| Gh_A12G1682 | CYP71A25 | *Gh_CYP71A25_192* | + | 1,692 | 497 | 56.533 | 7.5 | 7.674 | -0.138 | 1,494 | 1,494 | 44.4 | 2 | 747 | 198 | plas |
| Gh_A12G1729 | CYP71A26 | *Gh_CYP71A26_193* | - | 1,499 | 387 | 44.182 | 16 | 9.858 | -0.139 | 1,164 | 1,164 | 43.5 | 3 | 388 | 167.5 | E.R |
| Gh_D12G1841 | CYP71A26 | *Gh_CYP71A26_194* | + | 1,766 | 542 | 61.642 | 9 | 7.928 | -0.13 | 1,629 | 1,629 | 44.1 | 2 | 814.5 | 137 | E.R |
| Gh_D03G0961 | CYP71A1 | *Gh_CYP71A1_195* | - | 7,626 | 956 | 107.31 | -13.5 | 5.805 | -0.124 | 2,871 | 2,871 | 43.4 | 6 | 478.5 | 951 | E.R |
| Gh_A03G0664 | CYP71A1 | *Gh_CYP71A1_196* | - | 1,703 | 466 | 53.267 | 1.5 | 6.732 | -0.053 | 1,401 | 1,401 | 41.6 | 3 | 467 | 151 | E.R |
| Gh_A10G1109 | CYP71A1 | *Gh_CYP71A1_197* | + | 1,628 | 502 | 57.332 | 13 | 8.681 | -0.075 | 1,509 | 1,509 | 43 | 2 | 754.5 | 119 | plas |
| Gh_D10G1398 | CYP71A1 | *Gh_CYP71A1_198* | - | 1,630 | 508 | 58.033 | 12.5 | 8.537 | -0.024 | 1,527 | 1,527 | 43.2 | 2 | 763.5 | 103 | E.R |
| Gh_D07G0561 | CYP71A1 | *Gh_CYP71A1_199* | + | 2,585 | 525 | 60.551 | 6 | 7.78 | -0.083 | 1,578 | 1,578 | 38.9 | 2 | 789 | 1,007.00 | plas |
| Gh_D12G1840 | CYP71A1 | *Gh_CYP71A1_200* | - | 1,363 | 420 | 48.655 | -2.5 | 5.66 | -0.244 | 1,263 | 1,263 | 41 | 2 | 631.5 | 100 | nucl |
| Gh_A12G1681 | CYP71A1 | *Gh_CYP71A1_201* | - | 1,716 | 539 | 62.019 | 8.5 | 8.61 | -0.181 | 1,620 | 1,620 | 42.2 | 2 | 810 | 96 | E.R |
| Gh_A12G1679 | CYP71A1 | *Gh_CYP71A1_202* | - | 1,334 | 259 | 29.745 | -2.5 | 5.714 | -0.238 | 780 | 780 | 42.9 | 2 | 390 | 554 | nucl |
| Gh_D10G2055 | CYP71A1 | *Gh_CYP71A1_203* | + | 1,631 | 512 | 58.671 | 5 | 7.301 | -0.043 | 1,539 | 1,539 | 41.5 | 2 | 769.5 | 92 | E.R |
| Gh_D10G2544 | CYP71A1 | *Gh_CYP71A1_204* | - | 1,631 | 512 | 58.676 | 4.5 | 7.14 | -0.025 | 1,539 | 1,539 | 41.4 | 2 | 769.5 | 92 | E.R |
| Gh_D11G0381 | CYP71A1 | *Gh_CYP71A1_205* | - | 1,202 | 335 | 38.408 | -2.5 | 5.764 | -0.16 | 1,008 | 1,008 | 41.6 | 3 | 336 | 97 | nucl |
| Gh_A10G1781 | CYP71A1 | *Gh_CYP71A1_206* | - | 8,902 | 453 | 51.962 | 1 | 6.651 | -0.144 | 1,362 | 1,362 | 41.6 | 3 | 454 | 3,770.00 | E.R |
| Gh_D10G2058 | CYP71A1 | *Gh_CYP71A1_207* | - | 1,622 | 507 | 58.051 | 9 | 8.343 | -0.083 | 1,524 | 1,524 | 41.8 | 2 | 762 | 98 | E.R |
| Gh_D10G2057 | CYP71A1 | *Gh_CYP71A1_208* | + | 1,614 | 507 | 57.805 | 3 | 6.952 | -0.037 | 1,524 | 1,524 | 41.8 | 2 | 762 | 90 | E.R |
| Gh_D10G2056 | CYP71A1 | *Gh_CYP71A1_209* | + | 1,609 | 507 | 58.032 | 1 | 6.651 | -0.068 | 1,524 | 1,524 | 41.3 | 2 | 762 | 85 | E.R |
| Gh_Sca005458G01 | CYP71A1 | *Gh_CYP71A1_210* | + | 1,620 | 510 | 58.297 | 11 | 8.377 | -0.006 | 1,533 | 1,533 | 40.8 | 2 | 766.5 | 87 | E.R |
| Gh_D13G2116 | CYP71A1 | *Gh_CYP71A1_211* | + | 24,028 | 349 | 39.798 | 2 | 6.849 | -0.155 | 1,050 | 1,050 | 40.8 | 2 | 525 | 22,978.00 | nucl |
| Gh_D06G0790 | CYP75B1 | *Gh_CYP75B1_212* | - | 2,090 | 517 | 59.735 | 13.5 | 9.298 | -0.294 | 1,554 | 1,554 | 45.4 | 2 | 777 | 536 | nucl |
| Gh_A06G0689 | CYP75B1 | *Gh_CYP75B1_213* | - | 2,221 | 517 | 59.596 | 11 | 8.902 | -0.294 | 1,554 | 1,554 | 44.9 | 2 | 777 | 667 | mito |
| Gh_D11G1389 | CYP71A1 | *Gh_CYP71A1_214* | + | 2,478 | 511 | 58.269 | 9.5 | 8.613 | -0.23 | 1,536 | 1,536 | 45.5 | 2 | 768 | 942 | E.R |
| Gh_A11G1242 | CYP71A1 | *Gh_CYP71A1_215* | + | 2,504 | 511 | 58.367 | 8 | 8.284 | -0.21 | 1,536 | 1,536 | 45.4 | 2 | 768 | 968 | plas |
| Gh_A10G0501 | CYP75A7 | *Gh_CYP75A7_216* | - | 2,081 | 501 | 57.004 | 17 | 9.53 | -0.12 | 1,506 | 1,506 | 44.6 | 2 | 753 | 575 | E.R |
| Gh_D10G2598 | CYP71A1 | *Gh_CYP71A1_217* | + | 2,367 | 510 | 58.378 | 14.5 | 9.398 | -0.236 | 1,533 | 1,533 | 45.3 | 2 | 766.5 | 834 | E.R |
| Gh_A10G0500 | CYP71A1 | *Gh_CYP71A1_218* | - | 2,116 | 510 | 58.261 | 6 | 7.985 | -0.243 | 1,533 | 1,533 | 46.9 | 2 | 766.5 | 583 | nucl |
| Gh_D10G2599 | CYP71A1 | *Gh_CYP71A1_219* | + | 2,103 | 510 | 58.16 | 5 | 7.647 | -0.218 | 1,533 | 1,533 | 47.2 | 2 | 766.5 | 570 | nucl |
| Gh_D12G1132 | CYP703A2 | *Gh_CYP703A2_220* | - | 2,026 | 524 | 59.923 | 11 | 7.92 | -0.232 | 1,575 | 1,575 | 48.4 | 2 | 787.5 | 451 | nucl |
| Gh_A12G1014 | CYP703A2 | *Gh_CYP703A2_221* | - | 2,086 | 555 | 63.664 | 8 | 7.226 | -0.184 | 1,668 | 1,668 | 46.5 | 2 | 834 | 418 | E.R |
| Gh_D10G1865 | CYP79A2 | *Gh_CYP79A2_222* | - | 1,874 | 555 | 62.723 | 11.5 | 8.648 | -0.179 | 1,668 | 1,668 | 46.5 | 2 | 834 | 206 | E.R |
| Gh_D05G2655 | CYP79A2 | *Gh_CYP79A2_223* | - | 1,756 | 532 | 59.829 | 14 | 8.996 | -0.173 | 1,599 | 1,599 | 44.8 | 3 | 533 | 78.5 | nucl |
| Gh_A05G0953 | CYP79D4 | *Gh_CYP79D4_224* | + | 2,247 | 550 | 61.355 | 5.5 | 7.327 | -0.013 | 1,653 | 1,653 | 49.1 | 2 | 826.5 | 594 | plas |
| Gh_D05G1038 | CYP79D4 | *Gh_CYP79D4_225* | + | 2,249 | 550 | 61.738 | 6.5 | 7.536 | -0.023 | 1,653 | 1,653 | 48.8 | 2 | 826.5 | 596 | plas |
| Gh_D07G1947 | CYP79D4 | *Gh_CYP79D4_226* | + | 1,817 | 548 | 62.002 | 1.5 | 6.654 | -0.23 | 1,647 | 1,647 | 44.2 | 2 | 823.5 | 170 | mito |
| Gh_A07G1744 | CYP79D4 | *Gh_CYP79D4_227* | + | 1,817 | 545 | 61.584 | 8 | 7.41 | -0.207 | 1,638 | 1,638 | 44.4 | 2 | 819 | 179 | nucl |
| Gh_D07G1948 | CYP79D4 | *Gh_CYP79D4_228* | + | 1,823 | 547 | 62.155 | 4.5 | 6.973 | -0.227 | 1,644 | 1,644 | 44.2 | 2 | 822 | 179 | nucl |
| Gh_D11G1440 | CYP82C2 | *Gh_CYP82C2_229* | - | 1,712 | 528 | 59.756 | 21 | 9.598 | -0.088 | 1,587 | 1,587 | 46.5 | 2 | 793.5 | 125 | E.R |
| Gh_D07G1584 | CYP82C4 | *Gh_CYP82C4_230* | - | 1,917 | 530 | 59.615 | 0.5 | 6.562 | -0.047 | 1,593 | 1,593 | 45.4 | 2 | 796.5 | 324 | E.R |
| Gh_A07G1481 | CYP82C4 | *Gh_CYP82C4_231* | - | 1,911 | 530 | 59.594 | 1 | 6.617 | -0.058 | 1,593 | 1,593 | 45.9 | 2 | 796.5 | 318 | plas |
| Gh_D07G0911 | CYP82G1 | *Gh_CYP82G1_232* | + | 2,109 | 495 | 56.097 | 14.5 | 8.748 | -0.189 | 1,488 | 1,488 | 46.2 | 3 | 496 | 310.5 | nucl |
| Gh_D07G0912 | CYP82G1 | *Gh_CYP82G1_233* | + | 2,776 | 439 | 49.95 | 7.5 | 7.8 | -0.314 | 1,320 | 1,320 | 45.5 | 2 | 660 | 1,456.00 | E.R |
| Gh_A07G0841 | CYP82G1 | *Gh_CYP82G1_234* | + | 82,998 | 966 | 109.868 | 26 | 8.702 | -0.25 | 2,901 | 2,901 | 46.2 | 4 | 725.3 | 26,699.00 | nucl |
| Gh_D07G0910 | CYP82G1 | *Gh_CYP82G1_235* | + | 3,356 | 515 | 58.534 | 11 | 8.159 | -0.184 | 1,548 | 1,548 | 46.5 | 2 | 774 | 1,808.00 | E.R |
| Gh_D12G0394 | CYP82C4 | *Gh_CYP82C4_236* | - | 1,640 | 520 | 58.452 | 13 | 8.555 | -0.021 | 1,563 | 1,563 | 45.3 | 2 | 781.5 | 77 | nucl |
| Gh_A12G0265 | CYP82C4 | *Gh_CYP82C4_237* | + | 1,639 | 511 | 56.872 | 15 | 8.806 | 0.019 | 1,536 | 1,536 | 45.8 | 3 | 512 | 51.5 | nucl |
| Gh_D04G1715 | CYP82C4 | *Gh_CYP82C4_238* | - | 1,723 | 549 | 61.99 | 18 | 8.917 | -0.021 | 1,650 | 1,650 | 43.6 | 2 | 825 | 73 | nucl |
| Gh_A04G1107 | CYP82C4 | *Gh_CYP82C4_239* | - | 1,723 | 549 | 62.357 | 14.5 | 8.47 | -0.066 | 1,650 | 1,650 | 43.3 | 2 | 825 | 73 | nucl |
| Gh_A04G1108 | CYP82C4 | *Gh_CYP82C4_240* | - | 1,477 | 465 | 52.797 | 8.5 | 8.141 | -0.088 | 1,398 | 1,398 | 45.3 | 2 | 699 | 79 | nucl |
| Gh_D04G1721 | CYP82D47 | *Gh_CYP82D47_241* | - | 951 | 250 | 28.518 | -2.5 | 6.034 | -0.126 | 753 | 753 | 43 | 2 | 376.5 | 198 | nucl |
| Gh_D04G1725 | CYP82D47 | *Gh_CYP82D47_242* | + | 951 | 250 | 28.615 | -3.5 | 5.808 | -0.096 | 753 | 753 | 43.3 | 2 | 376.5 | 198 | nucl |
| Gh_D04G1717 | CYP82H23 | *Gh_CYP82H23_243* | + | 924 | 307 | 35.345 | 20.5 | 10.392 | -0.181 | 924 | 924 | 42.3 | 1 | 924 | No intron | nucl |
| Gh_D04G1716 | CYP82C4 | *Gh_CYP82C4_244* | - | 1,672 | 530 | 59.919 | 14.5 | 8.736 | -0.058 | 1,593 | 1,593 | 44.4 | 2 | 796.5 | 79 | nucl |
| Gh_D05G1897 | CYP82C4 | *Gh_CYP82C4_245* | - | 1,825 | 525 | 59.067 | 10 | 8.28 | -0.081 | 1,578 | 1,578 | 46.5 | 2 | 789 | 247 | E.R |
| Gh_A05G1710 | CYP82C4 | *Gh_CYP82C4_246* | - | 1,828 | 526 | 59.087 | 6 | 7.396 | -0.079 | 1,581 | 1,581 | 46.4 | 2 | 790.5 | 247 | E.R |
| Gh_A11G1748 | CYP82C4 | *Gh_CYP82C4_247* | - | 43,897 | 352 | 39.682 | 4.5 | 7.525 | -0.072 | 1,059 | 1,059 | 43.2 | 2 | 529.5 | 42,838.00 | E.R |
| Gh_D11G1904 | CYP82C4 | *Gh_CYP82C4_248* | - | 1,728 | 525 | 59.346 | 9 | 7.747 | -0.105 | 1,578 | 1,578 | 44.7 | 2 | 789 | 150 | E.R |
| Gh_A11G1747 | CYP82C3 | *Gh_CYP82C3_249* | - | 1,178 | 198 | 22.034 | 6 | 8.755 | -0.209 | 597 | 597 | 48.7 | 3 | 199 | 290.5 | nucl |
| Gh_A11G1745 | CYP82C3 | *Gh_CYP82C3_250* | - | 1,029 | 234 | 26.536 | -6 | 4.974 | -0.242 | 705 | 705 | 46.1 | 2 | 352.5 | 324 | nucl |
| Gh_A11G1746 | CYP82A3 | *Gh_CYP82A3_251* | - | 1,163 | 338 | 38.018 | 10 | 8.322 | -0.02 | 1,017 | 1,017 | 43.2 | 2 | 508.5 | 146 | E.R |
| Gh_D05G2486 | CYP82A3 | *Gh_CYP82A3_252* | - | 1,885 | 526 | 59.879 | 6 | 7.261 | -0.214 | 1,581 | 1,581 | 47.4 | 2 | 790.5 | 304 | plas |
| Gh_A05G2226 | CYP82A3 | *Gh_CYP82A3_253* | - | 7,283 | 537 | 60.985 | 11.5 | 8.524 | -0.221 | 1,614 | 1,614 | 46.4 | 3 | 538 | 2,834.50 | plas |
| Gh_A01G0960 | CYP82C4 | *Gh_CYP82C4_254* | + | 1,645 | 520 | 59.431 | 10 | 8.091 | -0.21 | 1,563 | 1,563 | 42.9 | 2 | 781.5 | 82 | plas |
| Gh_D01G1008 | CYP82C4 | *Gh_CYP82C4_255* | + | 1,654 | 520 | 59.161 | 11 | 8.222 | -0.191 | 1,563 | 1,563 | 43.4 | 2 | 781.5 | 91 | plas |
| Gh_A05G1704 | CYP82A2 | *Gh_CYP82A2_256* | - | 767 | 233 | 26.375 | 22 | 10.971 | -0.082 | 702 | 702 | 46.2 | 2 | 351 | 65 | nucl |
| Gh_D05G1892 | CYP82A4 | *Gh_CYP82A4_257* | - | 1,464 | 412 | 46.611 | 9 | 8.239 | -0.301 | 1,239 | 1,239 | 46.7 | 3 | 413 | 112.5 | plas |
| Gh_A05G1703 | CYP82C4 | *Gh_CYP82C4_258* | - | 669 | 222 | 24.897 | -0.5 | 6.416 | 0.018 | 669 | 669 | 46 | 1 | 669 | No intron | E.R |
| Gh_A06G0121 | CYP82A4 | *Gh_CYP82A4_259* | - | 1,441 | 338 | 38.401 | -3.5 | 6.049 | -0.18 | 1,017 | 1,017 | 40.9 | 2 | 508.5 | 424 | nucl |
| Gh_D06G0103 | CYP82A4 | *Gh_CYP82A4_260* | - | 1,160 | 298 | 34.024 | 3.5 | 7.128 | -0.209 | 897 | 897 | 40.9 | 2 | 448.5 | 263 | nucl |
| Gh_Sca053338G01 | CYP82A3 | *Gh_CYP82A3_261* | - | 657 | 218 | 24.414 | -1.5 | 6.227 | 0.016 | 657 | 657 | 40.9 | 1 | 657 | No intron | chlo |
| Gh_A06G0119 | CYP82A4 | *Gh_CYP82A4_262* | - | 1,723 | 525 | 59.6 | 17 | 9.345 | -0.152 | 1,578 | 1,578 | 41.9 | 2 | 789 | 145 | nucl |
| Gh_D06G0099 | CYP82A3 | *Gh_CYP82A3_263* | - | 5,392 | 523 | 59.664 | 23 | 9.633 | -0.186 | 1,572 | 1,572 | 41.5 | 3 | 524 | 1,910.00 | E.R |
| Gh_D06G0101 | CYP82A3 | *Gh_CYP82A3_264* | - | 1,721 | 525 | 59.866 | 15 | 8.899 | -0.126 | 1,578 | 1,578 | 41.5 | 2 | 789 | 143 | E.R |
| Gh_Sca005367G02 | CYP82D47 | *Gh_CYP82D47_265* | - | 440 | 98 | 11.164 | 0.5 | 6.784 | -0.487 | 297 | 297 | 42.4 | 2 | 148.5 | 143 | nucl |
| Gh_D06G0104 | CYP82A3 | *Gh_CYP82A3_266* | - | 510 | 169 | 18.796 | 10 | 10.318 | -0.004 | 510 | 510 | 43.5 | 1 | 510 | No intron | E.R |
| Gh_A06G0122 | CYP82A3 | *Gh_CYP82A3_267* | - | 510 | 169 | 18.802 | 12 | 10.598 | 0.03 | 510 | 510 | 43.5 | 1 | 510 | No intron | E.R |
| Gh_D06G0105 | CYP82A4 | *Gh_CYP82A4_268* | - | 1,704 | 483 | 54.696 | 9 | 8.226 | -0.091 | 1,452 | 1,452 | 41.5 | 3 | 484 | 126 | E.R |
| Gh_D06G0102 | CYP82A3 | *Gh_CYP82A3_269* | - | 1,732 | 525 | 59.418 | 12.5 | 8.998 | -0.172 | 1,578 | 1,578 | 42 | 2 | 789 | 154 | E.R |
| Gh_D06G0098 | CYP82C3 | *Gh_CYP82C3_270* | - | 6,943 | 472 | 53.556 | 2.5 | 6.8 | -0.188 | 1,419 | 1,419 | 42.6 | 4 | 354.8 | 1,841.30 | nucl |
| Gh_A06G0118 | CYP82A3 | *Gh_CYP82A3_271* | - | 2,371 | 526 | 59.83 | 7 | 7.521 | -0.214 | 1,581 | 1,581 | 41.7 | 2 | 790.5 | 790 | E.R |
| Gh_A06G0117 | CYP82A3 | *Gh_CYP82A3_272* | - | 2,110 | 525 | 60.05 | 16 | 9.462 | -0.195 | 1,578 | 1,578 | 40.8 | 2 | 789 | 532 | plas |
| Gh_D06G0097 | CYP82C3 | *Gh_CYP82C3_273* | - | 997 | 158 | 17.775 | -7.5 | 4.531 | -0.037 | 477 | 477 | 38.4 | 2 | 238.5 | 520 | Extr |
| Gh_D06G0096 | CYP82A1 | *Gh_CYP82A1_274* | - | 1,734 | 440 | 50.019 | 9 | 8.154 | -0.146 | 1,323 | 1,323 | 41.6 | 2 | 661.5 | 411 | plas |
| Gh_A06G0116 | CYP82A1 | *Gh_CYP82A1_275* | - | 1,999 | 521 | 59.639 | 9.5 | 7.855 | -0.125 | 1,566 | 1,566 | 40.5 | 3 | 522 | 216.5 | plas |
| Gh_Sca005367G01 | CYP82A3 | *Gh_CYP82A3_276* | - | 3,494 | 572 | 65.32 | 13 | 8.541 | -0.152 | 1,719 | 1,719 | 41.8 | 4 | 429.8 | 591.7 | E.R |
| Gh_D06G0100 | CYP82A3 | *Gh_CYP82A3_277* | - | 1,708 | 531 | 60.532 | 8 | 8.035 | -0.123 | 1,596 | 1,596 | 41.2 | 2 | 798 | 112 | nucl |
| Gh_A06G0120 | CYP82A3 | *Gh_CYP82A3_278* | - | 1,682 | 519 | 58.867 | 10.5 | 8.82 | -0.138 | 1,560 | 1,560 | 41 | 3 | 520 | 61 | nucl |
| Gh_D05G1896 | CYP82A3 | *Gh_CYP82A3_279* | - | 1,736 | 526 | 58.759 | 8.5 | 8.301 | -0.032 | 1,581 | 1,581 | 47.7 | 2 | 790.5 | 155 | E.R |
| Gh_A05G1709 | CYP82A3 | *Gh_CYP82A3_280* | - | 1,736 | 526 | 58.808 | 10.5 | 8.541 | -0.02 | 1,581 | 1,581 | 47.6 | 2 | 790.5 | 155 | E.R |
| Gh_A06G0123 | CYP82A3 | *Gh_CYP82A3_281* | - | 1,656 | 520 | 58.729 | 11 | 9.123 | -0.138 | 1,563 | 1,563 | 43.5 | 2 | 781.5 | 93 | nucl |
| Gh_D06G2297 | CYP82A3 | *Gh_CYP82A3_282* | - | 1,659 | 521 | 58.827 | 11 | 9.137 | -0.134 | 1,566 | 1,566 | 43.5 | 2 | 783 | 93 | nucl |
| Gh_Sca032195G01 | CYP82A3 | *Gh_CYP82A3_283* | + | 939 | 313 | 35.484 | 9 | 9.581 | -0.154 | 939 | 939 | 44.1 | 1 | 939 | No intron | nucl |
| Gh_D05G1895 | CYP82A3 | *Gh_CYP82A3_284* | + | 1,658 | 496 | 55.295 | 14 | 9.288 | -0.156 | 1,491 | 1,491 | 44.4 | 3 | 497 | 83.5 | E.R |
| Gh_A05G1708 | CYP82A3 | *Gh_CYP82A3_285* | + | 1,677 | 523 | 58.671 | 15 | 9.282 | -0.186 | 1,572 | 1,572 | 43.6 | 2 | 786 | 105 | E.R |
| Gh_A05G2685 | CYP82A3 | *Gh_CYP82A3_286* | - | 1,664 | 522 | 58.355 | 10.5 | 8.4 | -0.216 | 1,569 | 1,569 | 44 | 2 | 784.5 | 95 | cyto |
| Gh_D05G2983 | CYP82A3 | *Gh_CYP82A3_287* | - | 1,663 | 522 | 58.277 | 13 | 9.132 | -0.216 | 1,569 | 1,569 | 44.7 | 2 | 784.5 | 94 | cyto |
| Gh_D05G1894 | CYP82A3 | *Gh_CYP82A3_288* | - | 1,658 | 522 | 58.49 | 14 | 9.158 | -0.211 | 1,569 | 1,569 | 45.4 | 2 | 784.5 | 89 | E.R |
| Gh_A05G1707 | CYP82A3 | *Gh_CYP82A3_289* | - | 726 | 241 | 27.663 | 5.5 | 9.32 | -0.327 | 726 | 726 | 43.9 | 1 | 726 | No intron | E.R |
| Gh_D05G1893 | CYP82A3 | *Gh_CYP82A3_290* | - | 1,658 | 522 | 58.572 | 12 | 9.104 | -0.218 | 1,569 | 1,569 | 45.1 | 2 | 784.5 | 89 | plas |
| Gh_A05G1705 | CYP82A3 | *Gh_CYP82A3_291* | - | 1,658 | 522 | 58.604 | 13 | 9.235 | -0.21 | 1,569 | 1,569 | 44.4 | 2 | 784.5 | 89 | plas |
| Gh_A11G1996 | CYP81D11 | *Gh_CYP81D11_292* | + | 736 | 173 | 19.309 | -8 | 4.582 | -0.193 | 522 | 522 | 45.4 | 3 | 174 | 107 | nucl |
| Gh_Sca011991G01 | CYP81D11 | *Gh_CYP81D11_293* | - | 736 | 173 | 19.309 | -8 | 4.582 | -0.193 | 522 | 522 | 45.4 | 3 | 174 | 107 | nucl |
| Gh_D11G1986 | CYP81E8 | *Gh_CYP81E8_294* | - | 1,583 | 500 | 56.54 | 3.5 | 6.923 | -0.164 | 1,503 | 1,503 | 45.6 | 2 | 751.5 | 80 | nucl |
| Gh_A11G1997 | CYP81E8 | *Gh_CYP81E8_295* | + | 1,585 | 500 | 56.763 | 7 | 7.657 | -0.187 | 1,503 | 1,503 | 45.4 | 2 | 751.5 | 82 | E.R |
| Gh_D12G0534 | CYP81D1 | *Gh_CYP81D1_296* | - | 1,580 | 500 | 56.948 | 12 | 8.533 | -0.236 | 1,503 | 1,503 | 44.7 | 2 | 751.5 | 77 | plas |
| Gh_A12G0520 | CYP81D1 | *Gh_CYP81D1_297* | - | 1,583 | 500 | 56.987 | 12.5 | 8.535 | -0.222 | 1,503 | 1,503 | 44.9 | 2 | 751.5 | 80 | plas |
| Gh_D07G0776 | CYP81D1 | *Gh_CYP81D1_298* | + | 31,147 | 954 | 108.212 | 25 | 9.173 | -0.319 | 2,865 | 2,865 | 44.6 | 5 | 573 | 7,070.50 | plas |
| Gh_A07G0714 | CYP81D1 | *Gh_CYP81D1_299* | + | 110,621 | 1,088 | 123.329 | 18 | 7.761 | -0.306 | 3,267 | 3,267 | 44 | 6 | 544.5 | 21,470.80 | plas |
| Gh_D10G2494 | CYP81E8 | *Gh_CYP81E8_300* | + | 825 | 274 | 31.344 | 13.5 | 9.82 | -0.169 | 825 | 825 | 43.8 | 1 | 825 | No intron | E.R |
| Gh_D10G2495 | CYP81E8 | *Gh_CYP81E8_301* | + | 2,518 | 497 | 56.862 | 26 | 9.913 | -0.148 | 1,494 | 1,494 | 43.5 | 2 | 747 | 1,024.00 | cyto |
| Gh_D10G2493 | CYP81E8 | *Gh_CYP81E8_302* | + | 2,522 | 497 | 57.032 | 29.5 | 10.053 | -0.14 | 1,494 | 1,494 | 42 | 2 | 747 | 1,028.00 | plas |
| Gh_D12G0687 | CYP81E8 | *Gh_CYP81E8_303* | - | 2,262 | 504 | 57.428 | 5.5 | 7.384 | -0.13 | 1,515 | 1,515 | 44 | 2 | 757.5 | 747 | plas |
| Gh_A12G0685 | CYP81D1 | *Gh_CYP81D1_304* | - | 2,123 | 372 | 42.345 | 4.5 | 7.897 | -0.192 | 1,119 | 1,119 | 43 | 3 | 373 | 502 | mito |
| Gh_D03G0235 | CYP81E8 | *Gh_CYP81E8_305* | - | 3,286 | 444 | 50.35 | 17.5 | 9.55 | -0.328 | 1,335 | 1,335 | 45.3 | 2 | 667.5 | 1,951.00 | nucl |
| Gh_A02G1479 | CYP81E8 | *Gh_CYP81E8_306* | + | 3,432 | 507 | 57.103 | 11 | 8.16 | -0.133 | 1,524 | 1,524 | 44 | 2 | 762 | 1,908.00 | E.R |
| Gh_A06G0198 | CYP81E8 | *Gh_CYP81E8_307* | - | 1,385 | 434 | 50.504 | 3.5 | 7.237 | -0.192 | 1,305 | 1,305 | 43.8 | 2 | 652.5 | 80 | nucl |
| Gh_A02G0844 | CYP81E7 | *Gh_CYP81E7_308* | - | 1,618 | 508 | 58.018 | 6 | 7.783 | -0.162 | 1,527 | 1,527 | 45.6 | 2 | 763.5 | 91 | plas |
| Gh_D02G0893 | CYP81E7 | *Gh_CYP81E7_309* | - | 1,618 | 508 | 57.978 | 9 | 8.312 | -0.172 | 1,527 | 1,527 | 45.4 | 2 | 763.5 | 91 | plas |
| Gh_D05G1993 | CYP81D1 | *Gh_CYP81D1_310* | + | 2,256 | 379 | 44.485 | 2 | 6.941 | -0.295 | 1,140 | 1,140 | 44.7 | 4 | 285 | 372 | nucl |
| Gh_A05G1798 | CYP81E8 | *Gh_CYP81E8_311* | + | 1,704 | 542 | 61.706 | 12.5 | 8.68 | -0.185 | 1,629 | 1,629 | 45.2 | 2 | 814.5 | 75 | E.R |
| Gh_D05G2506 | CYP81E8 | *Gh_CYP81E8_312* | - | 1,594 | 501 | 57.63 | 16 | 9.13 | -0.219 | 1,506 | 1,506 | 46.3 | 2 | 753 | 88 | E.R |
| Gh_A05G2246 | CYP81E8 | *Gh_CYP81E8_313* | - | 17,877 | 501 | 57.977 | 17 | 9.114 | -0.195 | 1,506 | 1,506 | 44.6 | 2 | 753 | 16,371.00 | nucl |
| Gh_D05G1988 | CYP81E1 | *Gh_CYP81E1_314* | - | 2,483 | 518 | 58.272 | 3.5 | 7.089 | -0.112 | 1,557 | 1,557 | 43.9 | 2 | 778.5 | 926 | E.R |
| Gh_A05G1792 | CYP81E1 | *Gh_CYP81E1_315* | - | 2,459 | 518 | 58.118 | 1.5 | 6.737 | -0.082 | 1,557 | 1,557 | 44.1 | 2 | 778.5 | 902 | E.R |
| Gh_A05G1793 | CYP81D1 | *Gh_CYP81D1_316* | - | 336 | 111 | 12.169 | -4 | 4.663 | -0.177 | 336 | 336 | 49.1 | 1 | 336 | No intron | nucl |
| Gh_A02G0847 | CYP81E1 | *Gh_CYP81E1_317* | + | 2,179 | 533 | 61.228 | 9 | 7.981 | -0.014 | 1,602 | 1,602 | 42.8 | 2 | 801 | 577 | E.R |
| Gh_D02G0896 | CYP81E8 | *Gh_CYP81E8_318* | + | 2,173 | 536 | 61.591 | 12 | 8.353 | -0.02 | 1,611 | 1,611 | 43.6 | 3 | 537 | 281 | plas |
| Gh_A13G2072 | CYP98A2 | *Gh_CYP98A2_319* | + | 3,970 | 510 | 57.883 | 9 | 7.987 | -0.178 | 1,533 | 1,533 | 49.2 | 3 | 511 | 1,218.50 | plas |
| Gh_Sca194955G01 | CYP98A2 | *Gh_CYP98A2_320* | + | 153 | 51 | 5.95 | -1 | 5.635 | -0.724 | 153 | 153 | 53.6 | 1 | 153 | No intron | mito |
| Gh_A01G1681 | CYP98A2 | *Gh_CYP98A2_321* | + | 1,818 | 508 | 57.88 | 12 | 8.366 | -0.25 | 1,527 | 1,527 | 46.4 | 3 | 509 | 145.5 | E.R |
| Gh_D01G1930 | CYP98A2 | *Gh_CYP98A2_322* | + | 1,814 | 508 | 58.119 | 11.5 | 8.358 | -0.215 | 1,527 | 1,527 | 46.2 | 3 | 509 | 143.5 | E.R |
| Gh_D03G0436 | CYP78A7 | *Gh_CYP78A7_323* | - | 1,712 | 537 | 60.552 | 13 | 9.125 | -0.06 | 1,614 | 1,614 | 49.1 | 2 | 807 | 98 | plas |
| Gh_A02G1295 | CYP78A7 | *Gh_CYP78A7_324* | - | 1,712 | 537 | 60.683 | 11.5 | 8.763 | -0.051 | 1,614 | 1,614 | 48.8 | 2 | 807 | 98 | plas |
| Gh_A06G0999 | CYP78A7 | *Gh_CYP78A7_325* | - | 1,714 | 544 | 60.584 | 7 | 8.019 | 0.05 | 1,635 | 1,635 | 46 | 2 | 817.5 | 79 | nucl |
| Gh_D06G1191 | CYP78A7 | *Gh_CYP78A7_326* | - | 1,714 | 544 | 60.364 | 8.5 | 8.222 | 0.042 | 1,635 | 1,635 | 47.2 | 2 | 817.5 | 79 | nucl |
| Gh_A02G0970 | CYP78A6 | *Gh_CYP78A6_327* | - | 2,292 | 530 | 59.67 | 16.5 | 9.089 | 0.038 | 1,593 | 1,593 | 48.1 | 2 | 796.5 | 699 | E.R |
| Gh_D03G0782 | CYP78A3 | *Gh_CYP78A3_328* | + | 2,397 | 553 | 62.056 | 22.5 | 9.57 | NA | 1,662 | 1,662 | 48.6 | 2 | 831 | 735 | nucl |
| Gh_Sca116758G01 | CYP78A9 | *Gh_CYP78A9_329* | + | 330 | 109 | 12.266 | 1 | 7.42 | 0 | 330 | 330 | 53.3 | 1 | 330 | No intron | nucl |
| Gh_A08G0963 | CYP78A3 | *Gh_CYP78A3_330* | + | 1,779 | 532 | 60.015 | 16.5 | 8.856 | -0.005 | 1,599 | 1,599 | 47 | 2 | 799.5 | 180 | nucl |
| Gh_D08G1176 | CYP78A3 | *Gh_CYP78A3_331* | + | 1,782 | 533 | 60.146 | 15 | 8.656 | -0.012 | 1,602 | 1,602 | 47.4 | 2 | 801 | 180 | nucl |
| Gh_A12G2169 | CYP78A3 | *Gh_CYP78A3_332* | - | 1,834 | 530 | 59.821 | 11.5 | 8.478 | 0.009 | 1,593 | 1,593 | 47.1 | 2 | 796.5 | 241 | E.R |
| Gh_D12G2348 | CYP78A3 | *Gh_CYP78A3_333* | - | 1,835 | 530 | 59.809 | 14.5 | 8.807 | 0.001 | 1,593 | 1,593 | 47.1 | 2 | 796.5 | 242 | E.R |
| Gh_A11G0905 | CYP78A3 | *Gh_CYP78A3_334* | + | 1,835 | 540 | 60.659 | 13 | 8.554 | -0.024 | 1,623 | 1,623 | 47.6 | 2 | 811.5 | 212 | nucl |
| Gh_D11G1051 | CYP78A3 | *Gh_CYP78A3_335* | + | 1,828 | 540 | 60.786 | 16 | 8.863 | 0.008 | 1,623 | 1,623 | 47.5 | 2 | 811.5 | 205 | mito |
| Gh_D12G1148 | CYP78A6 | *Gh_CYP78A6_336* | + | 1,682 | 533 | 60.038 | 16.5 | 9.089 | 0.003 | 1,602 | 1,602 | 45.3 | 2 | 801 | 80 | nucl |
| Gh_A12G1029 | CYP78A3 | *Gh_CYP78A3_337* | + | 1,681 | 533 | 59.992 | 13.5 | 8.757 | -0.017 | 1,602 | 1,602 | 45.1 | 2 | 801 | 79 | nucl |
| Gh_D08G1378 | CYP78A6 | *Gh_CYP78A6_338* | - | 1,135 | 326 | 37.049 | 20 | 10.505 | -0.067 | 981 | 981 | 48 | 2 | 490.5 | 154 | cyto |
| Gh_A08G1098 | CYP78A6 | *Gh_CYP78A6_339* | - | 1,773 | 537 | 60.985 | 14.5 | 9.161 | -0.054 | 1,614 | 1,614 | 48 | 2 | 807 | 159 | cyto |
| Gh_Sca140089G01 | CYP78A9 | *Gh_CYP78A9_340* | + | 318 | 106 | 11.827 | 0 | 6.517 | -0.146 | 318 | 318 | 50.9 | 1 | 318 | No intron | nucl |
| Gh_A03G0918 | CYP78A5 | *Gh_CYP78A5_341* | + | 5,855 | 461 | 52.297 | 8.5 | 7.933 | 0.008 | 1,386 | 1,386 | 45.2 | 2 | 693 | 4,469.00 | nucl |
| Gh_D02G1300 | CYP78A5 | *Gh_CYP78A5_342* | + | 1,658 | 518 | 58.594 | 6.5 | 7.288 | 0.027 | 1,557 | 1,557 | 45.7 | 2 | 778.5 | 101 | nucl |
| Gh_D12G1591 | CYP78A5 | *Gh_CYP78A5_343* | - | 1,656 | 518 | 57.96 | 4.5 | 7.062 | 0.089 | 1,557 | 1,557 | 47.2 | 2 | 778.5 | 99 | nucl |
| Gh_A12G1471 | CYP78A5 | *Gh_CYP78A5_344* | - | 1,659 | 519 | 57.981 | 1.5 | 6.679 | 0.106 | 1,560 | 1,560 | 47.4 | 2 | 780 | 99 | nucl |
| Gh_D13G0865 | CYP78A5 | *Gh_CYP78A5_345* | + | 1,640 | 518 | 58.273 | 6 | 7.273 | 0.03 | 1,557 | 1,557 | 46.7 | 2 | 778.5 | 83 | E.R |
| Gh_A13G0740 | CYP78A5 | *Gh_CYP78A5_346* | + | 1,641 | 518 | 58.122 | 11 | 8.382 | 0.047 | 1,557 | 1,557 | 46.8 | 2 | 778.5 | 84 | E.R |
| Gh_A04G1209 | CYP78A5 | *Gh_CYP78A5_347* | + | 3,416 | 559 | 63.496 | 21 | 9.896 | 0.076 | 1,680 | 1,680 | 45.3 | 3 | 560 | 868 | nucl |
| Gh_D01G1515 | CYP78A5 | *Gh_CYP78A5_348* | - | 3,022 | 524 | 59.272 | 19 | 9.645 | 0.051 | 1,575 | 1,575 | 45 | 2 | 787.5 | 1,447.00 | E.R |
| Gh_A01G1350 | CYP78A3 | *Gh_CYP78A3_349* | - | 3,525 | 377 | 42.783 | 9.5 | 8.695 | 0.057 | 1,134 | 1,134 | 43.7 | 2 | 567 | 2,391.00 | E.R |
| Gh_A06G0128 | CYP701A3 | *Gh_CYP701A3_350* | + | 3,056 | 502 | 57.729 | 10.5 | 8.093 | -0.337 | 1,509 | 1,509 | 44.4 | 7 | 215.6 | 257.8 | E.R |
| Gh_D06G0106 | CYP701A3 | *Gh_CYP701A3_351* | + | 2,939 | 504 | 57.824 | 9.5 | 8.083 | -0.31 | 1,515 | 1,515 | 45 | 7 | 216.4 | 237.3 | E.R |
| Gh_D05G1901 | CYP701A3 | *Gh_CYP701A3_352* | + | 17,613 | 495 | 56.944 | 7.5 | 8.059 | -0.245 | 1,488 | 1,488 | 42.7 | 9 | 165.3 | 2,015.60 | E.R |
| Gh_A05G1711 | CYP701A3 | *Gh_CYP701A3_353* | + | 11,543 | 514 | 59.206 | 14 | 8.902 | -0.159 | 1,545 | 1,545 | 41.2 | 9 | 171.7 | 1,249.80 | E.R |
| Gh_A12G2395 | CYP71A26 | *Gh_CYP71A26_354* | + | 1,897 | 104 | 11.977 | -3 | 4.856 | -0.251 | 315 | 315 | 41 | 3 | 105 | 791 | nucl |
| Gh_D10G1845 | CYP73A11 | *Gh_CYP73A11_355* | + | 2,247 | 505 | 58.311 | 15 | 9.66 | -0.223 | 1,518 | 1,518 | 46.9 | 3 | 506 | 364.5 | plas |
| Gh_A10G1590 | CYP73A11 | *Gh_CYP73A11_356* | + | 2,248 | 505 | 58.252 | 15.5 | 9.661 | -0.222 | 1,518 | 1,518 | 46.7 | 3 | 506 | 365 | E.R |
| Gh_D13G2458 | CYP73A11 | *Gh_CYP73A11_357* | + | 2,126 | 505 | 58.068 | 13 | 9.431 | -0.266 | 1,518 | 1,518 | 48 | 3 | 506 | 304 | E.R |
| Gh_Sca069039G01 | CYP73A13 | *Gh_CYP73A13_358* | - | 387 | 128 | 14.41 | 1.5 | 7.612 | -0.334 | 387 | 387 | 45.5 | 1 | 387 | No intron | nucl |
| Gh_A13G2057 | CYP73A11 | *Gh_CYP73A11_359* | + | 2,161 | 505 | 58.052 | 13 | 9.454 | -0.258 | 1,518 | 1,518 | 48 | 3 | 506 | 321.5 | E.R |
| Gh_D11G2286 | CYP73A100 | *Gh_CYP73A100_360* | + | 945 | 314 | 36.554 | 16.5 | 9.882 | -0.057 | 945 | 945 | 44.2 | 1 | 945 | No intron | nucl |
| Gh_A11G3216 | CYP73A100 | *Gh_CYP73A100_361* | - | 1,080 | 359 | 41.491 | 3.5 | 6.998 | -0.245 | 1,080 | 1,080 | 47.5 | 1 | 1,080.00 | No intron | nucl |
| Gh_D11G2287 | CYP73A100 | *Gh_CYP73A100_362* | + | 630 | 209 | 23.555 | 6 | 8.51 | -0.089 | 630 | 630 | 50.5 | 1 | 630 | No intron | nucl |
| Gh_D01G0941 | CYP734A6 | *Gh_CYP734A6_363* | + | 531 | 176 | 20.281 | 11.5 | 10.335 | -0.269 | 531 | 531 | 46.7 | 1 | 531 | No intron | nucl |
| Gh_A05G0319 | CYP711A1 | *Gh_CYP711A1_364* | - | 2,434 | 539 | 60.669 | 15.5 | 9.355 | -0.169 | 1,620 | 1,620 | 41.4 | 5 | 324 | 203.5 | E.R |
| Gh_D05G0425 | CYP711A1 | *Gh_CYP711A1_365* | - | 2,438 | 539 | 60.617 | 16.5 | 9.434 | -0.158 | 1,620 | 1,620 | 41.6 | 5 | 324 | 204.5 | E.R |
| Gh_A06G1538 | CYP78A7 | *Gh_CYP78A7_366* | - | 3,117 | 559 | 63.396 | 15.5 | 8.61 | -0.116 | 1,680 | 1,680 | 40.2 | 7 | 240 | 239.5 | nucl |
| Gh_D06G1909 | CYP78A7 | *Gh_CYP78A7_367* | - | 3,064 | 559 | 63.387 | 20.5 | 9.26 | -0.116 | 1,680 | 1,680 | 40.1 | 7 | 240 | 230.7 | nucl |
| Gh_D07G0027 | CYP734A1 | *Gh_CYP734A1_368* | - | 2,844 | 518 | 59.648 | 27 | 9.904 | -0.06 | 1,557 | 1,557 | 44.4 | 5 | 311.4 | 321.8 | plas |
| Gh_A07G0021 | CYP734A1 | *Gh_CYP734A1_369* | - | 2,792 | 518 | 59.508 | 25.5 | 9.701 | -0.028 | 1,557 | 1,557 | 44.6 | 5 | 311.4 | 308.8 | plas |
| Gh_D06G0548 | CYP734A1 | *Gh_CYP734A1_370* | - | 2,570 | 515 | 58.689 | 24.5 | 9.955 | 0.015 | 1,548 | 1,548 | 45.3 | 5 | 309.6 | 255.5 | nucl |
| Gh_A06G1918 | CYP734A1 | *Gh_CYP734A1_371* | + | 2,566 | 515 | 58.631 | 24.5 | 9.955 | 0.01 | 1,548 | 1,548 | 45 | 5 | 309.6 | 254.5 | nucl |
| Gh_A08G2123 | CYP734A1 | *Gh_CYP734A1_372* | - | 3,080 | 517 | 59.228 | 18 | 9.552 | -0.067 | 1,554 | 1,554 | 43.2 | 5 | 310.8 | 381.5 | plas |
| Gh_D08G2495 | CYP734A1 | *Gh_CYP734A1_373* | - | 2,956 | 517 | 59.195 | 21 | 9.795 | -0.061 | 1,554 | 1,554 | 44.2 | 5 | 310.8 | 350.5 | E.R |
| Gh_A05G0122 | CYP734A1 | *Gh_CYP734A1_374* | - | 2,834 | 524 | 59.861 | 27 | 10.229 | -0.041 | 1,575 | 1,575 | 44.3 | 5 | 315 | 314.8 | plas |
| Gh_D05G0184 | CYP734A1 | *Gh_CYP734A1_375* | - | 2,832 | 524 | 59.772 | 24 | 10.092 | -0.023 | 1,575 | 1,575 | 44.4 | 5 | 315 | 314.3 | plas |
| Gh_A01G0951 | CYP72A15 | *Gh_CYP72A15_376* | + | 414 | 137 | 15.585 | 7.5 | 9.747 | 0.109 | 414 | 414 | 44.2 | 1 | 414 | No intron | nucl |
| Gh_D05G1863 | CYP72A219 | *Gh_CYP72A219_377* | + | 1,664 | 198 | 23.153 | 7 | 10.003 | -0.404 | 597 | 597 | 40.7 | 4 | 149.3 | 355.7 | mito |
| Gh_D07G1898 | CYP749A22 | *Gh_CYP749A22_378* | - | 2,112 | 519 | 59.463 | 15 | 9.109 | -0.09 | 1,560 | 1,560 | 38.8 | 5 | 312 | 138 | nucl |
| Gh_A13G0783 | CYP749A22 | *Gh_CYP749A22_379* | + | 2,427 | 520 | 59.753 | 10 | 8.299 | -0.077 | 1,563 | 1,563 | 39.3 | 5 | 312.6 | 216 | nucl |
| Gh_D13G0921 | CYP749A22 | *Gh_CYP749A22_380* | + | 2,418 | 519 | 59.431 | 8 | 7.536 | -0.069 | 1,560 | 1,560 | 39.4 | 5 | 312 | 214.5 | E.R |
| Gh_D13G0628 | CYP749A22 | *Gh_CYP749A22_381* | + | 2,187 | 513 | 58.87 | 15.5 | 9.081 | -0.169 | 1,542 | 1,542 | 39.6 | 5 | 308.4 | 161.3 | nucl |
| Gh_D10G1401 | CYP749A22 | *Gh_CYP749A22_382* | + | 1,933 | 520 | 59.249 | 12 | 9.174 | -0.211 | 1,563 | 1,563 | 41.8 | 5 | 312.6 | 92.5 | mito |
| Gh_A02G0224 | CYP749A22 | *Gh_CYP749A22_383* | + | 1,391 | 349 | 39.215 | 0 | 6.52 | -0.057 | 1,050 | 1,050 | 39.3 | 3 | 350 | 170.5 | E.R |
| Gh_D02G2386 | CYP749A22 | *Gh_CYP749A22_384* | + | 2,065 | 525 | 60.123 | 12 | 8.545 | -0.124 | 1,578 | 1,578 | 39.9 | 5 | 315.6 | 121.8 | nucl |
| Gh_D02G0287 | CYP749A22 | *Gh_CYP749A22_385* | + | 74,034 | 525 | 60.101 | 15 | 9.387 | -0.091 | 1,578 | 1,578 | 39.8 | 5 | 315.6 | 18,114.00 | E.R |
| Gh_A02G0221 | CYP749A22 | *Gh_CYP749A22_386* | + | 2,707 | 581 | 65.453 | 22.5 | 9.611 | -0.079 | 1,746 | 1,746 | 40.7 | 5 | 349.2 | 240.3 | nucl |
| Gh_D02G2387 | CYP749A22 | *Gh_CYP749A22_387* | + | 24,634 | 516 | 58.785 | 24.5 | 9.911 | -0.125 | 1,551 | 1,551 | 39.7 | 5 | 310.2 | 5,770.80 | mito |
| Gh_A02G0220 | CYP749A22 | *Gh_CYP749A22_388* | + | 2,253 | 516 | 58.865 | 27.5 | 10.08 | -0.13 | 1,551 | 1,551 | 39.3 | 5 | 310.2 | 175.5 | mito |
| Gh_D02G0289 | CYP749A22 | *Gh_CYP749A22_389* | + | 2,420 | 511 | 58.071 | 9.5 | 7.8 | -0.076 | 1,536 | 1,536 | 40.3 | 5 | 307.2 | 221 | E.R |
| Gh_A02G0222 | CYP749A22 | *Gh_CYP749A22_390* | + | 2,410 | 511 | 58.031 | 11 | 8.35 | -0.082 | 1,536 | 1,536 | 39.8 | 5 | 307.2 | 218.5 | nucl |
| Gh_A02G0197 | CYP749A22 | *Gh_CYP749A22_391* | - | 2,657 | 496 | 56.339 | 19.5 | 9.481 | -0.233 | 1,491 | 1,491 | 39.9 | 6 | 248.5 | 233.2 | E.R |
| Gh_D02G0285 | CYP749A22 | *Gh_CYP749A22_392* | + | 2,569 | 516 | 59.033 | 15 | 9.227 | -0.137 | 1,551 | 1,551 | 39.7 | 5 | 310.2 | 254.5 | nucl |
| Gh_D02G0286 | CYP749A22 | *Gh_CYP749A22_393* | + | 6,910 | 515 | 58.907 | 15 | 9.227 | -0.163 | 1,548 | 1,548 | 38.8 | 5 | 309.6 | 1,340.50 | nucl |
| Gh_A02G0219 | CYP749A22 | *Gh_CYP749A22_394* | + | 78,188 | 515 | 58.922 | 23 | 9.822 | -0.092 | 1,548 | 1,548 | 39.1 | 5 | 309.6 | 19,160.00 | nucl |
| Gh_A02G0225 | CYP749A22 | *Gh_CYP749A22_395* | + | 1,880 | 517 | 59.003 | 13.5 | 8.739 | -0.106 | 1,554 | 1,554 | 40.3 | 5 | 310.8 | 81.5 | E.R |
| Gh_A02G0223 | CYP749A22 | *Gh_CYP749A22_396* | + | 1,802 | 406 | 46.126 | 16.5 | 9.851 | -0.22 | 1,221 | 1,221 | 40.4 | 6 | 203.5 | 116.2 | E.R |
| Gh_D04G1889 | CYP749A22 | *Gh_CYP749A22_397* | - | 3,679 | 513 | 58.357 | 15.5 | 9.476 | -0.152 | 1,542 | 1,542 | 42 | 5 | 308.4 | 534.3 | nucl |
| Gh_Sca005510G01 | CYP749A22 | *Gh_CYP749A22_398* | - | 4,193 | 513 | 58.263 | 13.5 | 9.264 | -0.145 | 1,542 | 1,542 | 41.7 | 5 | 308.4 | 662.8 | nucl |
| Gh_D13G0888 | CYP749A22 | *Gh_CYP749A22_399* | + | 7,699 | 549 | 62.637 | 21 | 9.794 | -0.077 | 1,650 | 1,650 | 40.7 | 6 | 275 | 1,209.80 | nucl |
| Gh_D08G2673 | CYP72A219 | *Gh_CYP749A22_400* | - | 2,663 | 521 | 59.557 | 16.5 | 9.48 | -0.11 | 1,566 | 1,566 | 43.6 | 5 | 313.2 | 274.3 | nucl |
| Gh_A01G0294 | CYP72A15 | *Gh_CYP72A15_401* | + | 1,476 | 392 | 45.765 | 8.5 | 8.983 | -0.302 | 1,179 | 1,179 | 40.2 | 4 | 294.8 | 99 | E.R |
| Gh_D01G2314 | CYP72A15 | *Gh_CYP72A15_402* | + | 3,296 | 380 | 44.405 | 6.5 | 8.429 | -0.321 | 1,143 | 1,143 | 40.3 | 5 | 228.6 | 538.3 | E.R |
| Gh_D01G0331 | CYP72A15 | *Gh_CYP72A15_403* | + | 1,884 | 518 | 60.029 | 14.5 | 9.417 | -0.24 | 1,557 | 1,557 | 43 | 5 | 311.4 | 81.8 | mito |
| Gh_D01G2311 | CYP72A15 | *Gh_CYP72A15_404* | + | 1,757 | 476 | 55.045 | 8 | 8.531 | NA | 1,431 | 1,431 | 41.8 | 5 | 286.2 | 81.5 | mito |
| Gh_D01G2312 | CYP72A15 | *Gh_CYP72A15_405* | + | 1,883 | 518 | 59.803 | 11.5 | 8.761 | -0.189 | 1,557 | 1,557 | 42.3 | 5 | 311.4 | 81.5 | mito |
| Gh_A01G0290 | CYP72A15 | *Gh_CYP72A15_406* | + | 5,372 | 621 | 71.214 | 15 | 8.636 | -0.401 | 1,866 | 1,866 | 44.3 | 8 | 233.3 | 500.9 | plas |
| Gh_A01G0291 | CYP72A219 | *Gh_CYP72A219_407* | + | 1,885 | 518 | 60.073 | 14.5 | 9.168 | -0.268 | 1,557 | 1,557 | 42.4 | 5 | 311.4 | 82 | nucl |
| Gh_A01G0293 | CYP72A219 | *Gh_CYP72A219_408* | + | 1,886 | 518 | 59.802 | 14.5 | 9.223 | -0.215 | 1,557 | 1,557 | 42.8 | 5 | 311.4 | 82.3 | nucl |
| Gh_D01G2313 | CYP72A15 | *Gh_CYP72A15_409* | + | 3,106 | 557 | 64.61 | 15 | 9.042 | -0.212 | 1,674 | 1,674 | 41.9 | 6 | 279 | 286.4 | nucl |
| Gh_D05G0336 | CYP72A15 | *Gh_CYP72A15_410* | + | 1,921 | 501 | 57.248 | 16.5 | 9.326 | -0.179 | 1,506 | 1,506 | 42 | 5 | 301.2 | 103.8 | nucl |
| Gh_A05G3945 | CYP72A219 | *Gh_CYP72A219_411* | + | 1,010 | 289 | 33.574 | 15 | 9.917 | -0.036 | 870 | 870 | 41.1 | 3 | 290 | 70 | nucl |
| Gh_A12G1282 | CYP72A219 | *Gh_CYP72A219_412* | + | 2,436 | 514 | 59.176 | 18.5 | 9.576 | -0.218 | 1,545 | 1,545 | 43 | 5 | 309 | 222.8 | nucl |
| Gh_D12G1404 | CYP72A219 | *Gh_CYP72A219_413* | + | 2,440 | 514 | 59.175 | 17.5 | 9.486 | -0.225 | 1,545 | 1,545 | 42.8 | 5 | 309 | 223.8 | nucl |
| Gh_D12G0504 | CYP735A1 | *Gh_CYP735A1_414* | - | 2,088 | 519 | 59.53 | 31 | 9.977 | -0.143 | 1,560 | 1,560 | 43.5 | 5 | 312 | 132 | cyto |
| Gh_A12G0496 | CYP735A1 | *Gh_CYP735A1_415* | - | 1,986 | 519 | 59.567 | 28 | 9.819 | -0.147 | 1,560 | 1,560 | 44 | 5 | 312 | 106.5 | cyto |
| Gh_D11G1669 | CYP735A1 | *Gh_CYP735A1_416* | + | 3,030 | 522 | 59.893 | 26.5 | 9.629 | -0.136 | 1,569 | 1,569 | 43.9 | 5 | 313.8 | 365.3 | cyto |
| Gh_A11G1507 | CYP735A1 | *Gh_CYP735A1_417* | + | 2,827 | 522 | 59.985 | 24.5 | 9.457 | -0.15 | 1,569 | 1,569 | 43.8 | 5 | 313.8 | 314.5 | E.R |
| Gh_Sca142748G01 | CYP735A1 | *Gh_CYP735A1_418* | - | 279 | 93 | 10.893 | -2 | 5.171 | -0.283 | 279 | 279 | 40.5 | 1 | 279 | No intron | nucl |
| Gh_A09G0403 | CYP714A1 | *Gh_CYP714A1_419* | - | 3,682 | 448 | 51.669 | 16.5 | 9.54 | -0.196 | 1,347 | 1,347 | 43 | 4 | 336.8 | 778.3 | nucl |
| Gh_A09G1621 | CYP714A1 | *Gh_CYP714A1_420* | + | 2,043 | 521 | 59.49 | 26 | 10.058 | -0.153 | 1,566 | 1,566 | 45 | 4 | 391.5 | 159 | nucl |
| Gh_D09G1714 | CYP714A1 | *Gh_CYP714A1_421* | + | 2,048 | 521 | 59.378 | 26.5 | 10.085 | -0.161 | 1,566 | 1,566 | 45.5 | 4 | 391.5 | 160.7 | nucl |
| Gh_D12G2456 | CYP735A2 | *Gh_CYP735A2_422* | - | 4,642 | 519 | 59.042 | 17 | 9.58 | -0.061 | 1,560 | 1,560 | 42.5 | 4 | 390 | 1,027.30 | nucl |
| Gh_A12G2322 | CYP735A2 | *Gh_CYP735A2_423* | - | 4,815 | 519 | 59.001 | 15 | 9.398 | -0.074 | 1,560 | 1,560 | 42.8 | 4 | 390 | 1,085.00 | nucl |
| Gh_D12G0981 | CYP714C2 | *Gh_CYP714C2_424* | - | 1,896 | 522 | 59.064 | 16.5 | 9.177 | -0.052 | 1,569 | 1,569 | 41.8 | 5 | 313.8 | 81.8 | nucl |
| Gh_A12G0895 | CYP714C2 | *Gh_CYP714C2_425* | - | 1,891 | 546 | 61.91 | 11 | 8.085 | -0.038 | 1,641 | 1,641 | 41.2 | 4 | 410.3 | 83.3 | nucl |
| Gh_D05G0951 | CYP714C2 | *Gh_CYP714C2_426* | - | 1,920 | 512 | 58.132 | 11.5 | 9.013 | 0.022 | 1,539 | 1,539 | 44.2 | 5 | 307.8 | 95.3 | E.R |
| Gh_A05G0829 | CYP714A1 | *Gh_CYP714A1_427* | - | 1,920 | 388 | 44.143 | 8.5 | 8.554 | 0.129 | 1,167 | 1,167 | 45.2 | 5 | 233.4 | 188.3 | nucl |
| Gh_D07G1829 | CYP714C2 | *Gh_CYP714C2_428* | + | 1,970 | 516 | 57.87 | 11.5 | 8.832 | 0.017 | 1,551 | 1,551 | 44.5 | 5 | 310.2 | 104.8 | nucl |
| Gh_A07G1631 | CYP714C2 | *Gh_CYP714C2_429* | + | 23,538 | 516 | 58.002 | 17.5 | 9.405 | 0.028 | 1,551 | 1,551 | 44.5 | 5 | 310.2 | 5,496.80 | E.R |
| Gh_A13G0154 | CYP714C2 | *Gh_CYP714C2_430* | + | 1,333 | 141 | 15.941 | 2 | 7.162 | -0.009 | 426 | 426 | 40.1 | 2 | 213 | 907 | nucl |
| Gh_A10G1778 | CYP714B2 | *Gh_CYP714B2_431* | + | 1,864 | 510 | 57.416 | 18 | 9.172 | 0.003 | 1,533 | 1,533 | 42.7 | 5 | 306.6 | 82.8 | cyto |
| Gh_D10G2050 | CYP714B2 | *Gh_CYP714B2_432* | + | 1,864 | 510 | 57.336 | 13 | 8.629 | -0.022 | 1,533 | 1,533 | 42.6 | 5 | 306.6 | 82.8 | cyto |
| Gh_D04G0174 | CYP714C2 | *Gh_CYP714C2_433* | - | 1,936 | 513 | 57.846 | 16.5 | 9.035 | -0.085 | 1,542 | 1,542 | 42.8 | 5 | 308.4 | 98.5 | cyto |
| Gh_A05G3416 | CYP714C2 | *Gh_CYP714C2_434* | + | 1,932 | 513 | 57.612 | 15.5 | 8.907 | -0.075 | 1,542 | 1,542 | 42.7 | 5 | 308.4 | 97.5 | cyto |
| Gh_A13G0155 | CYP714A1 | *Gh_CYP714A1_435* | + | 1,502 | 262 | 29.896 | 2.5 | 7.179 | -0.05 | 789 | 789 | 45 | 3 | 263 | 356.5 | nucl |
| Gh_A05G0525 | CYP714A1 | *Gh_CYP714A1_436* | + | 4,139 | 523 | 58.847 | 21 | 9.548 | -0.068 | 1,572 | 1,572 | 44.3 | 5 | 314.4 | 641.8 | cyto |
| Gh_D05G0646 | CYP714A1 | *Gh_CYP714A1_437* | + | 3,812 | 523 | 58.881 | 20.5 | 9.47 | -0.079 | 1,572 | 1,572 | 44.2 | 5 | 314.4 | 560 | cyto |
| Gh_D04G0101 | CYP714A1 | *Gh_CYP714A1_438* | + | 2,917 | 522 | 58.912 | 14 | 8.957 | -0.112 | 1,569 | 1,569 | 46.4 | 5 | 313.8 | 337 | nucl |
| Gh_A05G3508 | CYP714A1 | *Gh_CYP714A1_439* | - | 2,888 | 520 | 58.653 | 15.5 | 9.004 | -0.104 | 1,563 | 1,563 | 46.4 | 5 | 312.6 | 331.3 | nucl |
| Gh_D07G1869 | CYP97B2 | *Gh_CYP97B2_440* | - | 7,182 | 579 | 64.742 | 0.5 | 6.596 | -0.117 | 1,740 | 1,740 | 42.5 | 14 | 124.3 | 418.6 | nucl |
| Gh_A07G2164 | CYP97B2 | *Gh_CYP97B2_441* | + | 7,118 | 579 | 64.774 | 2 | 6.8 | -0.123 | 1,740 | 1,740 | 42.5 | 14 | 124.3 | 413.7 | nucl |
| Gh_D13G1644 | CYP97A3 | *Gh_CYP97A3_442* | - | 5,436 | 618 | 69.106 | -3.5 | 5.836 | -0.212 | 1,857 | 1,857 | 42.8 | 16 | 116.1 | 238.6 | E.R |
| Gh_A13G1336 | CYP97A3 | *Gh_CYP97A3_443* | - | 5,476 | 616 | 68.857 | -4.5 | 5.658 | -0.18 | 1,851 | 1,851 | 42.6 | 16 | 115.7 | 241.7 | E.R |
| Gh_D11G3263 | CYP97C1 | *Gh_CYP97C1_444* | - | 4,268 | 548 | 61.422 | 0.5 | 6.64 | -0.125 | 1,647 | 1,647 | 42.4 | 9 | 183 | 327.6 | E.R |
| Gh_A11G2878 | CYP97C1 | *Gh_CYP97C1_445* | - | 4,250 | 548 | 61.313 | 4.5 | 7.827 | -0.138 | 1,647 | 1,647 | 42.2 | 9 | 183 | 325.4 | E.R |
| Gh_A07G1914 | CYP94A1 | *Gh_CYP94A1_446* | + | 1,524 | 507 | 57.977 | 9 | 8.447 | -0.196 | 1,524 | 1,524 | 47.4 | 1 | 1,524.00 | No intron | cyto |
| Gh_A07G1915 | CYP94A1 | *Gh_CYP94A1_447* | + | 1,524 | 507 | 57.713 | 11.5 | 9.042 | -0.172 | 1,524 | 1,524 | 47.3 | 1 | 1,524.00 | No intron | cyto |
| Gh_D07G2137 | CYP94A1 | *Gh_CYP94A1_448* | + | 1,524 | 507 | 57.851 | 14 | 9.353 | -0.161 | 1,524 | 1,524 | 47.2 | 1 | 1,524.00 | No intron | cyto |
| Gh_D07G2138 | CYP94A1 | *Gh_CYP94A1_449* | + | 1,524 | 507 | 57.922 | 12 | 9.042 | -0.138 | 1,524 | 1,524 | 47 | 1 | 1,524.00 | No intron | cyto |
| Gh_Sca179447G01 | CYP94A1 | *Gh_CYP94A1_450* | + | 186 | 62 | 7.439 | 3 | 9.788 | -0.584 | 186 | 186 | 53.2 | 1 | 186 | No intron | mito |
| Gh_A07G1867 | CYP94A1 | *Gh_CYP94A1_451* | - | 816 | 271 | 31.23 | -5 | 5.091 | -0.148 | 816 | 816 | 44.4 | 1 | 816 | No intron | nucl |
| Gh_D08G2033 | CYP94A1 | *Gh_CYP94A1_452* | + | 1,521 | 506 | 58.251 | 12.5 | 9.288 | -0.199 | 1,521 | 1,521 | 45.4 | 1 | 1,521.00 | No intron | nucl |
| Gh_A08G2364 | CYP94A1 | *Gh_CYP94A1_453* | + | 1,521 | 506 | 58.232 | 12.5 | 9.288 | -0.195 | 1,521 | 1,521 | 45.4 | 1 | 1,521.00 | No intron | E.R |
| Gh_D11G0392 | CYP94B3 | *Gh_CYP94B3_454* | - | 1,548 | 515 | 57.876 | 4 | 7.426 | 0.001 | 1,548 | 1,548 | 48.8 | 1 | 1,548.00 | No intron | E.R |
| Gh_A11G0336 | CYP94B3 | *Gh_CYP94B3_455* | - | 1,548 | 515 | 58.093 | 8 | 8.154 | -0.047 | 1,548 | 1,548 | 48.7 | 1 | 1,548.00 | No intron | nucl |
| Gh_D03G1088 | CYP94B3 | *Gh_CYP94B3_456* | - | 1,533 | 510 | 58.352 | 10.5 | 8.465 | -0.045 | 1,533 | 1,533 | 45.3 | 1 | 1,533.00 | No intron | mito |
| Gh_A03G0450 | CYP94B3 | *Gh_CYP94B3_457* | + | 1,533 | 510 | 58.292 | 7.5 | 8.052 | -0.025 | 1,533 | 1,533 | 45.1 | 1 | 1,533.00 | No intron | mito |
| Gh_A05G2953 | CYP94B3 | *Gh_CYP94B3_458* | + | 1,527 | 508 | 57.66 | 8.5 | 8.153 | 0.016 | 1,527 | 1,527 | 43.7 | 1 | 1,527.00 | No intron | nucl |
| Gh_D04G0742 | CYP94B3 | *Gh_CYP94B3_459* | + | 1,527 | 508 | 57.699 | 7 | 7.847 | 0.003 | 1,527 | 1,527 | 43.7 | 1 | 1,527.00 | No intron | nucl |
| Gh_A09G2505 | CYP94B3 | *Gh_CYP94B3_460* | - | 393 | 130 | 14.885 | 5.5 | 9.404 | -0.173 | 393 | 393 | 46.6 | 1 | 393 | No intron | nucl |
| Gh_A09G2506 | CYP94B3 | *Gh_CYP94B3_461* | - | 969 | 322 | 36.597 | 12 | 9.36 | -0.206 | 969 | 969 | 42.6 | 1 | 969 | No intron | nucl |
| Gh_D09G0361 | CYP94B3 | *Gh_CYP94B3_462* | - | 1,533 | 510 | 58.192 | 15.5 | 9.124 | -0.101 | 1,533 | 1,533 | 43.5 | 1 | 1,533.00 | No intron | nucl |
| Gh_A09G0894 | CYP94C1 | *Gh_CYP94C1_463* | + | 1,503 | 500 | 57.233 | 5 | 7.289 | -0.03 | 1,503 | 1,503 | 43.8 | 1 | 1,503.00 | No intron | nucl |
| Gh_D09G0920 | CYP94C1 | *Gh_CYP94C1_464* | + | 1,500 | 499 | 57.048 | 9.5 | 8.068 | -0.052 | 1,500 | 1,500 | 44.3 | 1 | 1,500.00 | No intron | nucl |
| Gh_A08G0043 | CYP94C1 | *Gh_CYP94C1_465* | + | 1,557 | 518 | 58.85 | 13 | 8.584 | -0.038 | 1,557 | 1,557 | 41.1 | 1 | 1,557.00 | No intron | Extr |
| Gh_D08G0085 | CYP94C1 | *Gh_CYP94C1_466* | + | 1,554 | 517 | 58.841 | 12 | 8.53 | -0.024 | 1,554 | 1,554 | 41.6 | 1 | 1,554.00 | No intron | E.R |
| Gh_D05G0830 | CYP94C1 | *Gh_CYP94C1_467* | - | 1,515 | 504 | 57.369 | 15 | 8.873 | -0.034 | 1,515 | 1,515 | 44.8 | 1 | 1,515.00 | No intron | nucl |
| Gh_A05G0684 | CYP94C1 | *Gh_CYP94C1_468* | - | 1,515 | 504 | 57.469 | 14.5 | 8.758 | -0.041 | 1,515 | 1,515 | 44.6 | 1 | 1,515.00 | No intron | nucl |
| Gh_A09G1292 | CYP94C1 | *Gh_CYP94C1_469* | - | 1,368 | 455 | 52.119 | 17.5 | 9.549 | -0.191 | 1,368 | 1,368 | 45.1 | 1 | 1,368.00 | No intron | E.R |
| Gh_D09G1325 | CYP94C1 | *Gh_CYP94C1_470* | + | 1,581 | 526 | 60.473 | 14 | 8.525 | 0.011 | 1,581 | 1,581 | 44.5 | 1 | 1,581.00 | No intron | E.R |
| Gh_A04G0529 | CYP94C1 | *Gh_CYP94C1_471* | + | 1,503 | 500 | 57.151 | 13 | 8.682 | 0.007 | 1,503 | 1,503 | 46.8 | 1 | 1,503.00 | No intron | plas |
| Gh_D04G0987 | CYP94C1 | *Gh_CYP94C1_472* | + | 1,503 | 500 | 57.137 | 12 | 8.575 | 0.017 | 1,503 | 1,503 | 46.8 | 1 | 1,503.00 | No intron | plas |
| Gh_D01G2017 | CYP94C1 | *Gh_CYP94C1_473* | + | 1,470 | 489 | 56.462 | 13 | 8.673 | -0.047 | 1,470 | 1,470 | 37.8 | 1 | 1,470.00 | No intron | plas |
| Gh_A01G1775 | CYP94C1 | *Gh_CYP94C1_474* | + | 669 | 222 | 25.593 | -1 | 6.128 | -0.161 | 669 | 669 | 39.6 | 1 | 669 | No intron | E.R |
| Gh_D10G2014 | CYP94A1 | *Gh_CYP94A1_475* | + | 1,509 | 502 | 57.918 | 9 | 8.15 | -0.223 | 1,509 | 1,509 | 42.3 | 1 | 1,509.00 | No intron | nucl |
| Gh_A10G1744 | CYP94A1 | *Gh_CYP94A1_476* | + | 1,509 | 502 | 57.966 | 11 | 8.452 | -0.197 | 1,509 | 1,509 | 42.7 | 1 | 1,509.00 | No intron | E.R |
| Gh_D08G2748 | CYP94A1 | *Gh_CYP94A1_477* | - | 1,928 | 531 | 60.829 | 14 | 8.882 | -0.185 | 1,596 | 1,596 | 44.9 | 2 | 798 | 332 | plas |
| Gh_A08G0096 | CYP94A1 | *Gh_CYP94A1_478* | - | 1,401 | 466 | 53.423 | 6.5 | 7.612 | -0.127 | 1,401 | 1,401 | 45.4 | 1 | 1,401.00 | No intron | plas |
| Gh_D03G1120 | CYP86A1 | *Gh_CYP86A1_479* | - | 1,536 | 511 | 58.635 | 12.5 | 9.043 | -0.168 | 1,536 | 1,536 | 46.7 | 1 | 1,536.00 | No intron | nucl |
| Gh_A03G0424 | CYP86A1 | *Gh_CYP86A1_480* | + | 1,536 | 511 | 58.839 | 10.5 | 8.977 | -0.209 | 1,536 | 1,536 | 46.6 | 1 | 1,536.00 | No intron | nucl |
| Gh_D05G0879 | CYP86A1 | *Gh_CYP86A1_481* | - | 1,536 | 511 | 58.567 | 15 | 9.499 | -0.145 | 1,536 | 1,536 | 47.1 | 1 | 1,536.00 | No intron | E.R |
| Gh_A05G0748 | CYP86A1 | *Gh_CYP86A1_482* | - | 1,536 | 511 | 58.564 | 15 | 9.499 | -0.145 | 1,536 | 1,536 | 46.9 | 1 | 1,536.00 | No intron | E.R |
| Gh_D07G1013 | CYP86A1 | *Gh_CYP86A1_483* | + | 1,545 | 514 | 58.605 | 20 | 9.788 | -0.174 | 1,545 | 1,545 | 46.7 | 1 | 1,545.00 | No intron | E.R |
| Gh_A07G0935 | CYP86A1 | *Gh_CYP86A1_484* | + | 1,545 | 514 | 58.616 | 21 | 9.905 | -0.181 | 1,545 | 1,545 | 46.7 | 1 | 1,545.00 | No intron | E.R |
| Gh_Sca117395G01 | CYP86A1 | *Gh_CYP86A1_485* | + | 300 | 99 | 11.343 | 7.5 | 10.166 | -0.207 | 300 | 300 | 49 | 1 | 300 | No intron | nucl |
| Gh_D08G1463 | CYP86A8 | *Gh_CYP86A8_486* | - | 1,584 | 527 | 60.038 | 10 | 8.031 | -0.09 | 1,584 | 1,584 | 48.9 | 1 | 1,584.00 | No intron | nucl |
| Gh_A08G1178 | CYP86A8 | *Gh_CYP86A8_487* | - | 1,584 | 527 | 59.978 | 9 | 7.877 | -0.068 | 1,584 | 1,584 | 48.4 | 1 | 1,584.00 | No intron | nucl |
| Gh_A11G0806 | CYP86A8 | *Gh_CYP86A8_488* | - | 1,587 | 528 | 59.87 | 10 | 8.21 | -0.125 | 1,587 | 1,587 | 49.5 | 1 | 1,587.00 | No intron | nucl |
| Gh_D11G0944 | CYP86A8 | *Gh_CYP86A8_489* | - | 1,587 | 528 | 59.792 | 10 | 8.21 | -0.122 | 1,587 | 1,587 | 49.7 | 1 | 1,587.00 | No intron | nucl |
| Gh_D08G1972 | CYP86A22 | *Gh_CYP86A22_490* | - | 1,605 | 534 | 60.937 | 13 | 8.648 | -0.126 | 1,605 | 1,605 | 45.2 | 1 | 1,605.00 | No intron | nucl |
| Gh_A08G2377 | CYP86A22 | *Gh_CYP86A22_491* | + | 1,605 | 534 | 60.998 | 11 | 8.39 | -0.137 | 1,605 | 1,605 | 44.4 | 1 | 1,605.00 | No intron | mito |
| Gh_D12G2271 | CYP86A22 | *Gh_CYP86A22_492* | - | 2,328 | 562 | 64.64 | 16.5 | 9.123 | -0.19 | 1,689 | 1,689 | 47.5 | 2 | 844.5 | 639 | E.R |
| Gh_A12G2100 | CYP86A22 | *Gh_CYP86A22_493* | - | 2,374 | 561 | 64.318 | 15.5 | 8.822 | -0.166 | 1,686 | 1,686 | 47.1 | 2 | 843 | 688 | E.R |
| Gh_D02G1587 | CYP86B1 | *Gh_CYP86B1_494* | + | 1,578 | 525 | 60.434 | 21.5 | 9.613 | -0.071 | 1,578 | 1,578 | 42.6 | 1 | 1,578.00 | No intron | E.R |
| Gh_A03G2129 | CYP86B1 | *Gh_CYP86B1_495* | + | 1,575 | 524 | 60.579 | 21 | 9.665 | -0.06 | 1,575 | 1,575 | 41.8 | 1 | 1,575.00 | No intron | E.R |
| Gh_A13G0518 | CYP86B1 | *Gh_CYP86B1_496* | - | 2,801 | 539 | 61.517 | 8.5 | 8.418 | -0.039 | 1,620 | 1,620 | 46.5 | 2 | 810 | 1,181.00 | plas |
| Gh_D13G0613 | ALAAT1 | *Gh_ALAAT1_497* | + | 2,064 | 265 | 29.626 | 16 | 9.797 | -0.147 | 798 | 798 | 45.9 | 8 | 99.8 | 180.9 | nucl |
| Gh_D11G2165 | CYP71E7 | *Gh_CYP71E7_498* | + | 1,064 | 227 | 25.258 | 8.5 | 9.812 | -0.001 | 684 | 684 | 42.4 | 3 | 228 | 190 | nucl |
| Gh_D13G0612 | CYP86B1 | *Gh_CYP86B1_499* | + | 1,064 | 122 | 14.193 | 3.5 | 8.431 | -0.309 | 369 | 369 | 45 | 2 | 184.5 | 695 | nucl |
| Gh_A04G0930 | CYP86B1 | *Gh_CYP86B1_500* | - | 6,470 | 644 | 74.093 | 41 | 10.02 | -0.33 | 1,935 | 1,935 | 45.4 | 5 | 387 | 1,133.80 | nucl |
| Gh_D04G1447 | CYP86B1 | *Gh_CYP86B1_501* | - | 4,325 | 644 | 74.026 | 42.5 | 10.047 | -0.329 | 1,935 | 1,935 | 45 | 5 | 387 | 597.5 | nucl |
| Gh_D09G0279 | CYP96A15 | *Gh_CYP96A15_502* | - | 1,442 | 431 | 49.444 | 7.5 | 7.748 | -0.26 | 1,296 | 1,296 | 43.3 | 2 | 648 | 146 | nucl |
| Gh_A09G0268 | CYP96A15 | *Gh_CYP96A15_503* | - | 1,511 | 472 | 53.923 | 9.5 | 7.756 | -0.147 | 1,419 | 1,419 | 42.8 | 2 | 709.5 | 92 | nucl |
| Gh_A09G0267 | CYP96A15 | *Gh_CYP96A15_504* | - | 909 | 302 | 34.832 | 11 | 8.92 | -0.365 | 909 | 909 | 40.4 | 1 | 909 | No intron | nucl |
| Gh_A09G0278 | CYP96A15 | *Gh_CYP96A15_505* | - | 1,297 | 301 | 34.623 | 7 | 8.074 | -0.173 | 906 | 906 | 42.9 | 2 | 453 | 391 | nucl |
| Gh_A09G0277 | CYP96A15 | *Gh_CYP96A15_506* | - | 1,068 | 355 | 40.82 | 9.5 | 8.448 | -0.239 | 1,068 | 1,068 | 42.2 | 1 | 1,068.00 | No intron | nucl |
| Gh_D09G0278 | CYP96A15 | *Gh_CYP96A15_507* | - | 1,512 | 503 | 58.234 | 15.5 | 8.714 | -0.19 | 1,512 | 1,512 | 43 | 1 | 1,512.00 | No intron | nucl |
| Gh_D11G2249 | CYP96A15 | *Gh_CYP96A15_508* | - | 1,524 | 507 | 58.92 | 15.5 | 8.57 | -0.177 | 1,524 | 1,524 | 42.1 | 1 | 1,524.00 | No intron | nucl |
| Gh_A11G3201 | CYP96A15 | *Gh_CYP96A15_509* | + | 826 | 261 | 29.745 | 11.5 | 9.786 | -0.42 | 786 | 786 | 42.1 | 2 | 393 | 40 | nucl |
| Gh_A11G1813 | CYP96A15 | *Gh_CYP96A15_510* | + | 1,500 | 499 | 57.503 | 16 | 8.734 | -0.062 | 1,500 | 1,500 | 42.4 | 1 | 1,500.00 | No intron | nucl |
| Gh_D11G3430 | CYP96A15 | *Gh_CYP96A15_511* | + | 1,500 | 499 | 57.636 | 13.5 | 8.404 | -0.098 | 1,500 | 1,500 | 42.9 | 1 | 1,500.00 | No intron | cyto |
| Gh_D10G1008 | CYP96A15 | *Gh_CYP96A15_512* | - | 1,521 | 506 | 58.273 | 13.5 | 8.528 | -0.096 | 1,521 | 1,521 | 43.5 | 1 | 1,521.00 | No intron | cyto |
| Gh_D13G0806 | CYP96A15 | *Gh_CYP96A15_513* | + | 1,497 | 498 | 56.763 | 15.5 | 8.475 | -0.132 | 1,497 | 1,497 | 43.7 | 1 | 1,497.00 | No intron | E.R |
| Gh_A05G3436 | CYP96A15 | *Gh_CYP96A15_514* | + | 1,533 | 510 | 59.122 | 24.5 | 9.806 | -0.212 | 1,533 | 1,533 | 42.7 | 1 | 1,533.00 | No intron | plas |
| Gh_A05G3438 | CYP96A15 | *Gh_CYP96A15_515* | - | 1,536 | 511 | 59.162 | 18 | 9.377 | -0.24 | 1,536 | 1,536 | 42.3 | 1 | 1,536.00 | No intron | cyto |
| Gh_A05G3439 | CYP96A15 | *Gh_CYP96A15_516* | - | 1,536 | 511 | 59.176 | 19 | 9.468 | -0.241 | 1,536 | 1,536 | 42.3 | 1 | 1,536.00 | No intron | cyto |
| Gh_D04G0154 | CYP96A15 | *Gh_CYP96A15_517* | - | 1,536 | 511 | 58.919 | 20.5 | 9.448 | -0.2 | 1,536 | 1,536 | 43.1 | 1 | 1,536.00 | No intron | cyto |
| Gh_D04G0153 | CYP96A15 | *Gh_CYP96A15_518* | + | 1,529 | 474 | 54.597 | 18.5 | 9.2 | -0.185 | 1,425 | 1,425 | 43.2 | 2 | 712.5 | 104 | cyto |
| Gh_A12G0229 | CYP96A15 | *Gh_CYP96A15_519* | - | 1,536 | 511 | 59.193 | 21.5 | 9.495 | -0.224 | 1,536 | 1,536 | 42.9 | 1 | 1,536.00 | No intron | plas |
| Gh_D12G0230 | CYP96A15 | *Gh_CYP96A15_520* | - | 1,536 | 511 | 59.12 | 18 | 9.18 | -0.235 | 1,536 | 1,536 | 43.4 | 1 | 1,536.00 | No intron | plas |
| Gh_D12G0227 | CYP96A15 | *Gh_CYP96A15_521* | - | 1,038 | 345 | 39.58 | 9 | 8.362 | -0.31 | 1,038 | 1,038 | 43.4 | 1 | 1,038.00 | No intron | plas |
| Gh_A12G0227 | CYP96A15 | *Gh_CYP96A15_522* | - | 345 | 114 | 12.845 | 6 | 9.298 | -0.43 | 345 | 345 | 43.2 | 1 | 345 | No intron | nucl |
| Gh_D12G0229 | CYP96A15 | *Gh_CYP96A15_523* | - | 1,210 | 372 | 42.651 | 16.5 | 9.044 | -0.068 | 1,119 | 1,119 | 42.4 | 3 | 373 | 45.5 | cyto |
| Gh_A12G0228 | CYP86A7 | *Gh_CYP86A7_524* | - | 896 | 189 | 21.905 | 10 | 9.671 | -0.344 | 570 | 570 | 44.2 | 2 | 285 | 326 | plas |
| Gh_A12G1506 | CYP704B1 | *Gh_CYP704B1_525* | + | 2,266 | 559 | 64.361 | 11 | 8.491 | -0.137 | 1,680 | 1,680 | 41.1 | 5 | 336 | 146.5 | nucl |
| Gh_D12G2768 | CYP704B1 | *Gh_CYP704B1_526* | + | 2,264 | 534 | 61.356 | 9.5 | 8.314 | -0.13 | 1,605 | 1,605 | 41.2 | 6 | 267.5 | 131.8 | nucl |
| Gh_A11G0751 | CYP704C1 | *Gh_CYP704C1_527* | + | 27,844 | 506 | 58.264 | 10 | 8.57 | -0.236 | 1,521 | 1,521 | 41.7 | 6 | 253.5 | 5,264.60 | nucl |
| Gh_D11G0872 | CYP704C1 | *Gh_CYP704C1_528* | + | 20,008 | 475 | 54.695 | 14 | 9.186 | -0.152 | 1,428 | 1,428 | 42 | 7 | 204 | 3,096.70 | nucl |
| Gh_A08G1144 | CYP704C1 | *Gh_CYP704C1_529* | + | 3,049 | 512 | 59.404 | 5 | 7.337 | -0.149 | 1,539 | 1,539 | 41.8 | 5 | 307.8 | 377.5 | nucl |
| Gh_D08G1427 | CYP704C1 | *Gh_CYP704C1_530* | + | 3,061 | 512 | 59.316 | 6 | 7.583 | -0.114 | 1,539 | 1,539 | 42.4 | 5 | 307.8 | 380.5 | nucl |
| Gh_A11G0750 | CYP704C1 | *Gh_CYP704C1_531* | + | 1,923 | 523 | 60.561 | 8.5 | 8.176 | -0.114 | 1,572 | 1,572 | 39.2 | 5 | 314.4 | 87.8 | E.R |
| Gh_D11G0871 | CYP704C1 | *Gh_CYP704C1_532* | + | 2,039 | 528 | 61.073 | 9.5 | 8.339 | -0.172 | 1,587 | 1,587 | 39.4 | 5 | 317.4 | 113 | E.R |
| Gh_D07G0402 | CYP704C1 | *Gh_CYP704C1_533* | + | 2,326 | 588 | 67.833 | 9.5 | 7.972 | -0.022 | 1,767 | 1,767 | 40.3 | 5 | 353.4 | 139.8 | nucl |
| Gh_A07G0342 | CYP704C1 | *Gh_CYP704C1_534* | + | 6,250 | 590 | 68.217 | 1 | 6.611 | -0.149 | 1,773 | 1,773 | 41.4 | 7 | 253.3 | 746.2 | mito |
| Gh_D06G1495 | CYP710A1 | *Gh_CYP710A1_535* | - | 1,512 | 503 | 57.893 | 14.5 | 9.066 | -0.027 | 1,512 | 1,512 | 47.6 | 1 | 1,512.00 | No intron | E.R |
| Gh_D05G1416 | CYP710A1 | *Gh_CYP710A1_536* | + | 1,509 | 502 | 58.052 | 5.5 | 7.598 | -0.109 | 1,509 | 1,509 | 46.9 | 1 | 1,509.00 | No intron | cyto |
| Gh_D05G1418 | CYP710A1 | *Gh_CYP710A1_537* | + | 630 | 209 | 24.135 | 2.5 | 7.623 | -0.256 | 630 | 630 | 44.1 | 1 | 630 | No intron | nucl |
| Gh_A05G3812 | CYP710A1 | *Gh_CYP710A1_538* | - | 1,509 | 502 | 57.551 | 6.5 | 7.793 | -0.095 | 1,509 | 1,509 | 48.4 | 1 | 1,509.00 | No intron | cyto |
| Gh_A05G1254 | CYP710A1 | *Gh_CYP710A1_539* | + | 1,509 | 502 | 57.753 | 8.5 | 8.147 | -0.103 | 1,509 | 1,509 | 48.3 | 1 | 1,509.00 | No intron | nucl |
| Gh_D05G1417 | CYP710A1 | *Gh_CYP710A1_540* | + | 1,509 | 502 | 57.635 | 7 | 7.706 | -0.063 | 1,509 | 1,509 | 47.5 | 1 | 1,509.00 | No intron | nucl |
| Gh_D05G1419 | CYP710A2 | *Gh_CYP710A2_541* | + | 180 | 59 | 6.727 | 0.5 | 7.521 | -0.058 | 180 | 180 | 53.3 | 1 | 180 | No intron | mito |
| Gh_A11G1429 | CYP74B2 | *Gh_CYP74B2_542* | + | 10,585 | 512 | 56.575 | 2.5 | 7.151 | -0.121 | 1,539 | 1,539 | 48.3 | 2 | 769.5 | 9,046.00 | E.R |
| Gh_D11G1581 | CYP74B2 | *Gh_CYP74B2_543* | + | 8,772 | 512 | 56.477 | 3.5 | 7.529 | -0.121 | 1,539 | 1,539 | 49 | 2 | 769.5 | 7,233.00 | plas |
| Gh_A12G0268 | CYP74A2 | *Gh_CYP74A2_544* | + | 1,464 | 487 | 54.831 | 3 | 7.189 | -0.033 | 1,464 | 1,464 | 42.8 | 1 | 1,464.00 | No intron | nucl |
| Gh_A12G0269 | CYP74A2 | *Gh_CYP74A2_545* | + | 1,269 | 422 | 47.665 | 2.5 | 6.985 | -0.003 | 1,269 | 1,269 | 41.8 | 1 | 1,269.00 | No intron | nucl |
| Gh_D05G2484 | CYP74A | *Gh_CYP74A_546* | + | 1,572 | 523 | 58.765 | 18 | 9.812 | -0.258 | 1,572 | 1,572 | 47 | 1 | 1,572.00 | No intron | E.R |
| Gh_A05G2224 | CYP74A | *Gh_CYP74A_547* | + | 1,572 | 523 | 58.797 | 18 | 9.812 | -0.247 | 1,572 | 1,572 | 46.7 | 1 | 1,572.00 | No intron | E.R |
| Gh_A03G2119 | CYP74A | *Gh_CYP74A_548* | - | 1,533 | 510 | 57.452 | 9.5 | 8.816 | -0.163 | 1,533 | 1,533 | 46.7 | 1 | 1,533.00 | No intron | nucl |
| Gh_D02G0931 | CYP74A | *Gh_CYP74A_549* | - | 1,533 | 510 | 57.582 | 10.5 | 8.958 | -0.21 | 1,533 | 1,533 | 46.4 | 1 | 1,533.00 | No intron | E.R |
| Gh_A06G0111 | CYP74A | *Gh_CYP74A_550* | + | 1,488 | 495 | 55.778 | 4.5 | 7.951 | -0.171 | 1,488 | 1,488 | 42.2 | 1 | 1,488.00 | No intron | plas |
| Gh_D06G0087 | CYP74A | *Gh_CYP74A_551* | + | 1,488 | 495 | 55.829 | -1.5 | 6.215 | -0.181 | 1,488 | 1,488 | 42.3 | 1 | 1,488.00 | No intron | E.R |
| Gh_D06G0089 | CYP74A | *Gh_CYP74A_552* | + | 1,455 | 455 | 51.084 | 3.5 | 7.569 | -0.173 | 1,368 | 1,368 | 42.5 | 2 | 684 | 87 | E.R |
| Gh_A06G0112 | CYP74A | *Gh_CYP74A_553* | + | 3,223 | 511 | 57.638 | 4.5 | 7.822 | -0.141 | 1,536 | 1,536 | 44 | 3 | 512 | 843.5 | E.R |
| Gh_D06G0091 | CYP74A2 | *Gh_CYP74A2_554* | + | 651 | 216 | 24.332 | -3.5 | 5.36 | 0.009 | 651 | 651 | 45.8 | 1 | 651 | No intron | nucl |
| Gh_D06G0090 | CYP74A | *Gh_CYP74A_555* | + | 1,491 | 496 | 56.174 | 1.5 | 6.878 | -0.108 | 1,491 | 1,491 | 42.6 | 1 | 1,491.00 | No intron | E.R |
| Gh_D06G0088 | CYP74A | *Gh_CYP74A_556* | + | 1,491 | 496 | 56.071 | 2.5 | 7.166 | -0.102 | 1,491 | 1,491 | 42.3 | 1 | 1,491.00 | No intron | E.R |
| Gh_A12G1291 | CYP51G1 | *Gh_CYP51G1_557* | + | 2,371 | 486 | 55.268 | 13.5 | 9.216 | -0.158 | 1,461 | 1,461 | 44.7 | 2 | 730.5 | 910 | nucl |
| Gh_D12G1413 | CYP51G1 | *Gh_CYP51G1_558* | + | 2,398 | 486 | 55.191 | 13 | 9.076 | -0.151 | 1,461 | 1,461 | 45 | 2 | 730.5 | 937 | nucl |
| Gh_D02G1792 | CYP51G1 | *Gh_CYP51G1_559* | + | 2,037 | 486 | 55.509 | 10 | 8.64 | -0.187 | 1,461 | 1,461 | 46.1 | 2 | 730.5 | 576 | nucl |
| Gh_A03G1352 | CYP51G1 | *Gh_CYP51G1_560* | + | 2,037 | 486 | 55.638 | 10.5 | 8.84 | -0.195 | 1,461 | 1,461 | 45.9 | 2 | 730.5 | 576 | nucl |
| Gh_D05G1939 | CYP707A4 | *Gh_CYP707A4_561* | - | 2,876 | 471 | 53.616 | 23 | 9.961 | -0.231 | 1,416 | 1,416 | 44.2 | 9 | 157.3 | 182.5 | E.R |
| Gh_A05G1745 | CYP707A4 | *Gh_CYP707A4_562* | - | 4,947 | 476 | 54.281 | 26 | 10.062 | -0.242 | 1,431 | 1,431 | 44.6 | 10 | 143.1 | 390.7 | plas |
| Gh_D02G2083 | CYP707A4 | *Gh_CYP707A4_563* | + | 3,762 | 473 | 54.037 | 22 | 9.94 | -0.208 | 1,422 | 1,422 | 42 | 9 | 158 | 292.5 | cyto |
| Gh_A03G1667 | CYP707A4 | *Gh_CYP707A4_564* | + | 4,149 | 474 | 54.06 | 23 | 9.942 | -0.172 | 1,425 | 1,425 | 41.8 | 9 | 158.3 | 340.5 | plas |
| Gh_D13G0395 | CYP707A4 | *Gh_CYP707A4_565* | - | 2,462 | 392 | 44.837 | 13 | 9.273 | -0.281 | 1,179 | 1,179 | 43.9 | 8 | 147.4 | 183.3 | E.R |
| Gh_A13G0351 | CYP707A4 | *Gh_CYP707A4_566* | - | 2,715 | 434 | 49.697 | 13.5 | 8.94 | -0.273 | 1,305 | 1,305 | 43.4 | 9 | 145 | 176.3 | E.R |
| Gh_D03G1182 | CYP707A2 | *Gh_CYP707A2_567* | + | 2,885 | 500 | 56.973 | 21.5 | 9.661 | -0.176 | 1,503 | 1,503 | 44 | 8 | 187.9 | 197.4 | plas |
| Gh_A07G0908 | CYP707A2 | *Gh_CYP707A2_568* | - | 3,915 | 346 | 39.096 | 20.5 | 10.018 | -0.27 | 1,041 | 1,041 | 43.9 | 7 | 148.7 | 479 | E.R |
| Gh_D07G0979 | CYP707A2 | *Gh_CYP707A2_569* | - | 3,729 | 388 | 44.221 | 17 | 9.833 | -0.32 | 1,167 | 1,167 | 42.8 | 7 | 166.7 | 427 | nucl |
| Gh_D12G2002 | CYP707A1 | *Gh_CYP707A1_570* | - | 1,997 | 463 | 53.017 | 21.5 | 9.824 | -0.135 | 1,392 | 1,392 | 43.4 | 7 | 198.9 | 100.8 | plas |
| Gh_A12G1830 | CYP707A1 | *Gh_CYP707A1_571* | - | 2,008 | 463 | 53.014 | 21.5 | 9.837 | -0.133 | 1,392 | 1,392 | 43.6 | 7 | 198.9 | 102.7 | plas |
| Gh_D08G1639 | CYP707A1 | *Gh_CYP707A1_572* | - | 5,443 | 438 | 49.944 | 18 | 9.778 | -0.11 | 1,317 | 1,317 | 43.6 | 7 | 188.1 | 687.7 | E.R |
| Gh_A08G1344 | CYP707A1 | *Gh_CYP707A1_573* | - | 3,336 | 446 | 50.726 | 15 | 9.532 | -0.227 | 1,341 | 1,341 | 43.4 | 8 | 167.6 | 285 | E.R |
| Gh_A02G0666 | CYP707A3 | *Gh_CYP707A3_574* | + | 10,323 | 752 | 84.408 | 10 | 7.886 | -0.067 | 2,259 | 2,259 | 40.8 | 21 | 107.6 | 403.2 | nucl |
| Gh_D02G0709 | CYP707A3 | *Gh_CYP707A3_575* | - | 2,526 | 477 | 54.187 | 12 | 9.329 | -0.149 | 1,434 | 1,434 | 40.7 | 9 | 159.3 | 136.5 | nucl |
| Gh_D02G0712 | CYP707A3 | *Gh_CYP707A3_576* | + | 2,523 | 477 | 54.111 | 12 | 9.329 | -0.168 | 1,434 | 1,434 | 40.9 | 9 | 159.3 | 136.1 | nucl |
| Gh_D12G0593 | CYP707A3 | *Gh_CYP707A3_577* | + | 2,316 | 489 | 55.513 | 9 | 8.515 | -0.173 | 1,470 | 1,470 | 42.2 | 9 | 163.3 | 105.8 | nucl |
| Gh_A12G0581 | CYP707A3 | *Gh_CYP707A3_578* | + | 2,325 | 491 | 55.692 | 10 | 8.663 | -0.143 | 1,476 | 1,476 | 42.3 | 9 | 164 | 106.1 | nucl |
| Gh_A06G0061 | CYP707A4 | *Gh_CYP707A4_579* | + | 5,778 | 432 | 48.862 | 3.5 | 7.466 | -0.125 | 1,299 | 1,299 | 41.5 | 9 | 144.3 | 559.9 | mito |
| Gh_D06G0038 | CYP707A4 | *Gh_CYP707A4_580* | + | 5,763 | 426 | 48.076 | 0.5 | 6.631 | -0.067 | 1,281 | 1,281 | 41.4 | 9 | 142.3 | 560.3 | mito |
| Gh_A05G3273 | CYP725A2 | *Gh_CYP725A2_581* | + | 1,612 | 484 | 55.151 | 16.5 | 9.112 | -0.104 | 1,455 | 1,455 | 44.1 | 3 | 485 | 78.5 | nucl |
| Gh_D04G0334 | CYP725A2 | *Gh_CYP725A2_582* | - | 1,612 | 484 | 55.219 | 18.5 | 9.487 | -0.108 | 1,455 | 1,455 | 44.1 | 3 | 485 | 78.5 | mito |
| Gh_A10G0050 | CYP716B2 | *Gh_ CYP716B2_583* | + | 2,887 | 486 | 55.312 | 17.5 | 9.764 | -0.172 | 1,461 | 1,461 | 44.9 | 3 | 487 | 713 | E.R |
| Gh_D10G0057 | CYP716B2 | *Gh_ CYP716B2_584* | + | 2,879 | 486 | 55.236 | 12.5 | 9.31 | -0.213 | 1,461 | 1,461 | 44.8 | 3 | 487 | 709 | plas |
| Gh_A13G0992 | CYP716B2 | *Gh_ CYP716B2_585* | + | 1,643 | 487 | 55.051 | 9 | 8.65 | -0.19 | 1,464 | 1,464 | 46.5 | 3 | 488 | 89.5 | plas |
| Gh_D10G2277 | CYP716B2 | *Gh_ CYP716B2_586* | + | 2,653 | 482 | 54.613 | 7 | 8.303 | -0.23 | 1,449 | 1,449 | 46.4 | 3 | 483 | 602 | E.R |
| Gh_D13G1240 | CYP716B2 | *Gh_ CYP716B2_587* | + | 1,625 | 481 | 54.578 | 8 | 8.486 | -0.202 | 1,446 | 1,446 | 46.3 | 3 | 482 | 89.5 | plas |
| Gh_D13G1224 | CYP716B2 | *Gh_ CYP716B2_588* | - | 1,631 | 483 | 54.495 | 10.5 | 8.817 | -0.209 | 1,452 | 1,452 | 46.3 | 3 | 484 | 89.5 | plas |
| Gh_A13G0977 | CYP716B2 | *Gh_ CYP716B2_589* | + | 1,630 | 483 | 54.533 | 7.5 | 8.311 | -0.217 | 1,452 | 1,452 | 46.2 | 3 | 484 | 89 | plas |
| Gh_D08G0180 | CYP716B2 | *Gh_ CYP716B2_590* | + | 4,861 | 480 | 54.705 | 15 | 9.163 | -0.099 | 1,443 | 1,443 | 43.2 | 3 | 481 | 1,709.00 | nucl |
| Gh_A08G0128 | CYP716B2 | *Gh_ CYP716B2_591* | + | 4,621 | 480 | 54.964 | 15 | 9.236 | -0.137 | 1,443 | 1,443 | 42.7 | 3 | 481 | 1,589.00 | nucl |
| Gh_D05G1098 | CYP716B2 | *Gh_ CYP716B2_592* | - | 2,435 | 476 | 54.224 | 10.5 | 8.984 | -0.168 | 1,431 | 1,431 | 46.3 | 3 | 477 | 502 | plas |
| Gh_A05G0987 | CYP716B2 | *Gh_ CYP716B2_593* | - | 2,408 | 476 | 54.146 | 10.5 | 8.998 | -0.158 | 1,431 | 1,431 | 46.5 | 3 | 477 | 488.5 | plas |
| Gh_D06G1932 | CYP725A4 | *Gh_CYP725A4_594* | - | 3,828 | 494 | 55.602 | 12.5 | 9.365 | -0.03 | 1,485 | 1,485 | 42.9 | 4 | 371.3 | 781 | nucl |
| Gh_A06G1578 | CYP725A4 | *Gh_CYP725A4_595* | - | 3,663 | 494 | 55.766 | 13 | 9.487 | -0.05 | 1,485 | 1,485 | 42.6 | 4 | 371.3 | 726 | nucl |
| Gh_D01G0629 | CYP716B1 | *Gh_CYP716B1_596* | - | 1,736 | 474 | 54.341 | 23 | 10.147 | -0.109 | 1,425 | 1,425 | 43.6 | 4 | 356.3 | 103.7 | nucl |
| Gh_A01G0613 | CYP716B1 | *Gh_CYP716B1_597* | - | 1,730 | 474 | 54.194 | 24 | 10.194 | -0.091 | 1,425 | 1,425 | 43.5 | 4 | 356.3 | 101.7 | nucl |
| Gh_D10G0141 | CYP716B1 | *Gh_CYP716B1_598* | - | 17,324 | 2,144 | 245.579 | 191 | 10.716 | -0.144 | 6,435 | 6,435 | 40.9 | 28 | 229.8 | 403.3 | E.R |
| Gh_A10G0135 | CYP716B2 | *Gh_CYP716B2_599* | - | 5,210 | 571 | 64.39 | 19.5 | 9.69 | -0.179 | 1,716 | 1,716 | 42.9 | 5 | 343.2 | 873.5 | nucl |
| Gh_A12G2062 | CYP87A3 | *Gh_CYP87A3_600* | + | 2,304 | 470 | 53.887 | 16.5 | 9.52 | -0.152 | 1,413 | 1,413 | 41.9 | 9 | 157 | 111.4 | nucl |
| Gh_D03G1481 | CYP87A3 | *Gh_CYP87A3_601* | - | 2,181 | 474 | 54.368 | 18.5 | 9.749 | -0.1 | 1,425 | 1,425 | 41.8 | 9 | 158.3 | 94.5 | nucl |
| Gh_A03G2024 | CYP87A3 | *Gh_CYP87A3_602* | - | 2,177 | 411 | 47.431 | 14.5 | 9.634 | -0.133 | 1,236 | 1,236 | 41.8 | 7 | 176.6 | 156.8 | nucl |
| Gh_D03G1482 | CYP87A3 | *Gh_CYP87A3_603* | - | 2,193 | 474 | 54.586 | 14 | 9.421 | -0.15 | 1,425 | 1,425 | 41.1 | 9 | 158.3 | 96 | nucl |
| Gh_A03G2025 | CYP87A3 | *Gh_CYP87A3_604* | - | 44,126 | 471 | 54.058 | 14 | 9.432 | -0.132 | 1,416 | 1,416 | 40.9 | 9 | 157.3 | 5,338.80 | nucl |
| Gh_D12G2485 | CYP87A3 | *Gh_CYP87A3_605* | + | 1,679 | 378 | 43.107 | 5.5 | 7.354 | 0.038 | 1,137 | 1,137 | 38.1 | 6 | 189.5 | 108.4 | nucl |
| Gh_D12G2482 | CYP87A3 | *Gh_CYP87A3_606* | - | 6,420 | 315 | 35.767 | 8 | 8.469 | NA | 948 | 948 | 40.9 | 3 | 316 | 2,736.00 | nucl |
| Gh_A12G2347 | CYP87A3 | *Gh_CYP87A3_607* | - | 7,005 | 456 | 52.073 | 17 | 8.883 | -0.029 | 1,371 | 1,371 | 38.3 | 7 | 195.9 | 939 | nucl |
| Gh_D07G0514 | CYP87A3 | *Gh_CYP87A3_608* | + | 6,836 | 444 | 50.33 | 11 | 9.018 | -0.144 | 1,335 | 1,335 | 42.7 | 8 | 166.9 | 785.9 | nucl |
| Gh_A07G0450 | CYP87A3 | *Gh_CYP87A3_609* | + | 2,311 | 444 | 50.429 | 10 | 8.965 | -0.159 | 1,335 | 1,335 | 42.5 | 8 | 166.9 | 139.4 | nucl |
| Gh_D01G2344 | CYP87A3 | *Gh_CYP87A3_610* | + | 2,550 | 482 | 55.483 | 8.5 | 8.403 | -0.143 | 1,449 | 1,449 | 41.4 | 9 | 161 | 137.6 | nucl |
| Gh_A01G0396 | CYP87A3 | *Gh_CYP87A3_611* | - | 10,093 | 425 | 48.843 | 3.5 | 6.997 | -0.186 | 1,278 | 1,278 | 41.8 | 11 | 116.2 | 881.5 | nucl |
| Gh_D01G2343 | CYP87A3 | *Gh_CYP87A3_612* | + | 10,622 | 480 | 55.119 | 8.5 | 8.197 | -0.178 | 1,443 | 1,443 | 41.4 | 9 | 160.3 | 1,147.40 | nucl |
| Gh_A03G0401 | CYP87A3 | *Gh_CYP87A3_613* | - | 11,394 | 524 | 58.776 | 5.5 | 7.206 | -0.088 | 1,575 | 1,575 | 43.2 | 12 | 131.3 | 892.6 | nucl |
| Gh_D03G1138 | CYP87A3 | *Gh_CYP87A3_614* | + | 4,903 | 483 | 55.531 | 16.5 | 9.442 | -0.138 | 1,452 | 1,452 | 42.8 | 9 | 161.3 | 431.4 | nucl |
| Gh_D13G0870 | CYP85A | *Gh_ CYP85A1_615* | + | 2,299 | 466 | 53.572 | 19.5 | 9.318 | -0.198 | 1,401 | 1,401 | 41.5 | 9 | 155.7 | 112.3 | nucl |
| Gh_A13G0746 | CYP85A1 | *Gh_CYP85A1_616* | + | 1,287 | 256 | 30.034 | 8.5 | 8.681 | -0.379 | 771 | 771 | 40.3 | 7 | 110.1 | 86 | nucl |
| Gh_D07G1822 | CYP85A | *Gh_ CYP85A _617* | - | 2,587 | 494 | 57.098 | 16.5 | 9.521 | -0.18 | 1,485 | 1,485 | 39.3 | 8 | 185.6 | 157.4 | nucl |
| Gh_A07G1623 | CYP85A | *Gh_ CYP85A _618* | - | 2,611 | 466 | 53.783 | 14.5 | 9.381 | -0.272 | 1,401 | 1,401 | 40.3 | 9 | 155.7 | 151.3 | nucl |
| Gh_A07G0725 | CYP85A | *Gh_ CYP85A _619* | - | 2,893 | 448 | 51.391 | 17.5 | 9.73 | -0.181 | 1,347 | 1,347 | 36.7 | 9 | 149.7 | 193.3 | nucl |
| Gh_D07G0783 | CYP85A | *Gh_ CYP85A _620* | - | 4,177 | 482 | 55.504 | 15.5 | 9.145 | -0.282 | 1,449 | 1,449 | 37.3 | 9 | 161 | 341 | nucl |
| Gh_D11G1862 | CYP90B1 | *Gh_CYP90B1_621* | - | 3,173 | 516 | 59.283 | 17 | 9.316 | -0.146 | 1,551 | 1,551 | 41.5 | 7 | 221.6 | 270.3 | nucl |
| Gh_A11G1705 | CYP90B1 | *Gh_CYP90B1_622* | - | 3,325 | 515 | 59.059 | 16.5 | 9.195 | -0.164 | 1,548 | 1,548 | 42 | 7 | 221.1 | 296.2 | nucl |
| Gh_A11G1365 | CYP724B1 | *Gh_CYP724B1_623* | + | 3,310 | 490 | 55.621 | 14.5 | 8.402 | -0.174 | 1,473 | 1,473 | 41.6 | 9 | 163.7 | 229.6 | nucl |
| Gh_D11G1510 | CYP724B1 | *Gh_CYP724B1_624* | + | 3,334 | 493 | 55.911 | 11.5 | 7.971 | -0.177 | 1,482 | 1,482 | 41.4 | 9 | 164.7 | 231.5 | nucl |
| Gh_D04G0555 | CYP724B1 | *Gh_CYP724B1_625* | - | 7,482 | 445 | 50.944 | 10.5 | 8.5 | -0.065 | 1,338 | 1,338 | 39.7 | 10 | 133.8 | 682.7 | mito |
| Gh_A05G3085 | CYP724B1 | *Gh_CYP724B1_626* | + | 2,572 | 438 | 50.125 | 14 | 8.901 | -0.11 | 1,317 | 1,317 | 41.1 | 9 | 146.3 | 156.9 | mito |
| Gh_A10G2109 | CYP90A1 | *Gh_CYP90A1_627* | + | 4,622 | 470 | 53.901 | 18 | 9.823 | -0.184 | 1,413 | 1,413 | 44.7 | 8 | 176.6 | 458.4 | nucl |
| Gh_D10G2490 | CYP90A1 | *Gh_CYP90A1_628* | - | 4,517 | 473 | 54.305 | 20 | 9.905 | -0.185 | 1,422 | 1,422 | 45.2 | 8 | 177.8 | 442.1 | nucl |
| Gh_A06G1446 | CYP90A1 | *Gh_CYP90A1_629* | - | 4,337 | 472 | 54.142 | 17 | 9.77 | -0.179 | 1,419 | 1,419 | 44.3 | 8 | 177.4 | 416.9 | plas |
| Gh_D06G1792 | CYP90A1 | *Gh_CYP90A1_630* | + | 4,314 | 472 | 54.103 | 17.5 | 9.77 | -0.161 | 1,419 | 1,419 | 45.1 | 8 | 177.4 | 413.6 | plas |
| Gh_D05G1281 | CYP90D1 | *Gh_CYP90D1_631* | - | 3,435 | 501 | 57.694 | 12.5 | 9.039 | 0.039 | 1,506 | 1,506 | 40 | 8 | 188.3 | 275.6 | nucl |
| Gh_A05G3816 | CYP90D1 | *Gh_CYP90D1_632* | + | 9,113 | 451 | 52.086 | 7.5 | 8.545 | -0.211 | 1,356 | 1,356 | 41.1 | 10 | 135.6 | 861.9 | mito |
| Gh_A02G1611 | CYP90D1 | *Gh_* CYP90D1*_633* | + | 6,350 | 514 | 58.413 | 10.5 | 8.693 | -0.133 | 1,545 | 1,545 | 40.5 | 9 | 171.7 | 600.6 | nucl |
| Gh_D03G0111 | CYP90D1 | *Gh_* CYP90D1*_634* | - | 6,701 | 516 | 58.451 | 10.5 | 8.806 | -0.14 | 1,551 | 1,551 | 41.5 | 9 | 172.3 | 643.8 | nucl |
| Gh_A11G1658 | CYP90D1 | *Gh_* CYP90D1*_635* | - | 3,752 | 491 | 56.214 | 14.5 | 9.306 | -0.208 | 1,476 | 1,476 | 42.3 | 9 | 164 | 284.5 | cyto |
| Gh_D11G1818 | CYP90D1 | *Gh_* CYP90D1*_636* | - | 3,697 | 491 | 56.15 | 12.5 | 9.008 | -0.179 | 1,476 | 1,476 | 42.3 | 9 | 164 | 277.6 | cyto |
| Gh_A13G0103 | CYP88A3 | *Gh_* CYP88A3*_637* | - | 3,140 | 463 | 53.61 | 8 | 8.112 | -0.146 | 1,392 | 1,392 | 43.1 | 7 | 198.9 | 291.3 | cyto |
| Gh_D13G0119 | CYP88A3 | *Gh_* CYP88A3*_638* | - | 3,054 | 367 | 42.5 | 3.5 | 7.003 | -0.267 | 1,104 | 1,104 | 42.9 | 6 | 184 | 390 | E.R |
| Gh_A06G1386 | CYP88A3 | *Gh_* CYP88A3*_639* | - | 3,053 | 444 | 51.422 | 15.5 | 9.503 | -0.323 | 1,335 | 1,335 | 41.8 | 7 | 190.7 | 286.3 | E.R |
| Gh_D06G1730 | CYP88A3 | *Gh_* CYP88A3*_640* | - | 3,460 | 523 | 60.375 | 12.5 | 9.161 | -0.189 | 1,572 | 1,572 | 43.4 | 7 | 224.6 | 314.7 | nucl |
| Gh_D08G1725 | CYP88A3 | *Gh_* CYP88A3*_641* | - | 89,711 | 430 | 49.843 | 18.5 | 9.816 | -0.166 | 1,293 | 1,293 | 41.5 | 6 | 215.5 | 17,683.60 | E.R |
| Gh_A08G1431 | CYP88A4 | *Gh_* CYP88A4*_642* | - | 21,376 | 487 | 55.851 | 13.5 | 9.171 | -0.125 | 1,464 | 1,464 | 42.1 | 8 | 183 | 2,844.60 | E.R |
| Gh_Sca015724G01 | CYP88D6 | *Gh_CYP88D6_643* | - | 1,710 | 310 | 35.531 | 5.5 | 8.417 | -0.216 | 933 | 933 | 42.6 | 7 | 133.3 | 129.5 | nucl |
| Gh_D09G1890 | CYP88D6 | *Gh_CYP88D6_644* | + | 3,927 | 464 | 52.936 | 21.5 | 9.677 | -0.215 | 1,395 | 1,395 | 42.3 | 7 | 199.3 | 422 | plas |
| Gh_Sca030829G01 | CYP88A3 | *Gh_* CYP88A3*_645* | - | 332 | 71 | 7.739 | 1.5 | 8.211 | 0.179 | 216 | 216 | 41.2 | 2 | 108 | 116 | chlo |
| Gh_A09G2349 | CYP88D6 | *Gh_CYP88D6_646* | + | 2,964 | 416 | 47.74 | 22.5 | 9.908 | -0.29 | 1,251 | 1,251 | 41.2 | 7 | 178.7 | 285.5 | plas |
| Gh_A08G1430 | CYP88D6 | *Gh_CYP88D6_647* | - | 4,606 | 490 | 56.346 | 22.5 | 9.803 | -0.196 | 1,473 | 1,473 | 41.7 | 8 | 184.1 | 447.6 | mito |
| Gh_Sca005221G01 | CYP88D6 | *Gh_CYP88D6_648* | + | 2,572 | 486 | 55.508 | 21 | 9.666 | -0.188 | 1,461 | 1,461 | 41.5 | 8 | 182.6 | 158.7 | mito |
| Gh_Sca015778G01 | CYP89A2 | *Gh_CYP89A2_649* | - | 1,130 | 235 | 27.586 | 6 | 8.621 | -0.354 | 708 | 708 | 41.2 | 3 | 236 | 211 | nucl |
| Gh_D13G0938 | CYP89A2 | *Gh_CYP89A2_650* | + | 1,554 | 517 | 59.482 | 20.5 | 10.007 | -0.138 | 1,554 | 1,554 | 43.9 | 1 | 1,554.00 | No intron | nucl |
| Gh_D03G0537 | CYP89A2 | *Gh_CYP89A2_651* | + | 1,539 | 512 | 59.279 | 17.5 | 9.704 | -0.182 | 1,539 | 1,539 | 43.6 | 1 | 1,539.00 | No intron | plas |
| Gh_A02G1117 | CYP89A2 | *Gh_CYP89A2_652* | + | 1,539 | 512 | 59.258 | 17.5 | 9.727 | -0.169 | 1,539 | 1,539 | 43.2 | 1 | 1,539.00 | No intron | plas |
| Gh_D08G2695 | CYP89A2 | *Gh_CYP89A2_653* | + | 1,545 | 514 | 59.16 | 15.5 | 9.499 | -0.219 | 1,545 | 1,545 | 43.9 | 1 | 1,545.00 | No intron | E.R |
| Gh_A08G2322 | CYP89A9 | *Gh_CYP89A9_654* | + | 1,545 | 514 | 59.205 | 17 | 9.697 | -0.202 | 1,545 | 1,545 | 43.8 | 1 | 1,545.00 | No intron | nucl |
| Gh_A11G2695 | CYP89A2 | *Gh_CYP89A2_655* | + | 1,530 | 509 | 58.887 | 16 | 9.599 | -0.176 | 1,530 | 1,530 | 42.7 | 1 | 1,530.00 | No intron | E.R |
| Gh_D11G3053 | CYP89A2 | *Gh_CYP89A2_656* | + | 1,647 | 548 | 63.119 | 25.5 | 9.931 | -0.275 | 1,647 | 1,647 | 42.6 | 1 | 1,647.00 | No intron | nucl |
| Gh_A11G2696 | CYP89A2 | *Gh_CYP89A2_657* | + | 1,539 | 512 | 58.809 | 14 | 9.625 | -0.148 | 1,539 | 1,539 | 42.4 | 1 | 1,539.00 | No intron | E.R |
| Gh_D11G3054 | CYP89A2 | *Gh_CYP89A2_658* | + | 1,539 | 512 | 58.832 | 14 | 9.602 | -0.143 | 1,539 | 1,539 | 42.6 | 1 | 1,539.00 | No intron | E.R |
| Gh_A11G2693 | CYP89A2 | *Gh_CYP89A2_659* | + | 1,527 | 508 | 58.554 | 10.5 | 8.449 | -0.07 | 1,527 | 1,527 | 42.5 | 1 | 1,527.00 | No intron | nucl |
| Gh_D11G3052 | CYP89A2 | *Gh_CYP89A2_660* | + | 1,542 | 513 | 58.687 | 9 | 8.318 | -0.062 | 1,542 | 1,542 | 43.2 | 1 | 1,542.00 | No intron | nucl |
| Gh_A10G0140 | CYP77A3 | *Gh_CYP77A3_661* | - | 1,530 | 509 | 57.78 | 9.5 | 8.395 | -0.046 | 1,530 | 1,530 | 47.4 | 1 | 1,530.00 | No intron | cyto |
| Gh_D10G0144 | CYP77A3 | *Gh_CYP77A3_662* | - | 1,521 | 506 | 57.394 | 9.5 | 8.396 | -0.017 | 1,521 | 1,521 | 47.4 | 1 | 1,521.00 | No intron | cyto |
| Gh_D06G2203 | CYP77A3 | *Gh_CYP77A3_663* | + | 1,521 | 506 | 57.344 | 12.5 | 8.753 | -0.086 | 1,521 | 1,521 | 46.9 | 1 | 1,521.00 | No intron | nucl |
| Gh_A06G1776 | CYP77A3 | *Gh_CYP77A3_664* | - | 717 | 238 | 27.037 | 14.5 | 10.375 | 0.098 | 717 | 717 | 45.5 | 1 | 717 | No intron | nucl |
| Gh_A06G1775 | CYP77A2 | *Gh_CYP77A2_665* | - | 714 | 237 | 26.431 | -7.5 | 4.876 | -0.086 | 714 | 714 | 48.3 | 1 | 714 | No intron | nucl |
| Gh_A06G1290 | CYP77A3 | *Gh_CYP77A3_666* | + | 1,518 | 505 | 57.029 | 12 | 8.668 | -0.021 | 1,518 | 1,518 | 48.9 | 1 | 1,518.00 | No intron | mito |
| Gh_D06G1613 | CYP77A3 | *Gh_CYP77A3_667* | + | 1,518 | 505 | 56.938 | 11.5 | 8.667 | -0.006 | 1,518 | 1,518 | 49.1 | 1 | 1,518.00 | No intron | mito |
| Gh_A10G1767 | CYP77A3 | *Gh_CYP77A3_668* | - | 1,518 | 505 | 57.001 | 8.5 | 8.366 | -0.154 | 1,518 | 1,518 | 47.4 | 1 | 1,518.00 | No intron | E.R |
| Gh_D10G2041 | CYP77A3 | *Gh_CYP77A3_669* | - | 1,518 | 505 | 57.088 | 8.5 | 8.366 | -0.165 | 1,518 | 1,518 | 47.4 | 1 | 1,518.00 | No intron | E.R |
| Gh_Sca041349G01 | CYP77A3 | *Gh_CYP77A3_670* | - | 744 | 248 | 28.468 | 9 | 9.584 | -0.224 | 744 | 744 | 45.6 | 1 | 744 | No intron | nucl |
| Gh_A12G2559 | CYP77A3 | *Gh_CYP77A3_671* | - | 1,560 | 519 | 59.165 | 21 | 9.848 | -0.117 | 1,560 | 1,560 | 41.6 | 1 | 1,560.00 | No intron | cyto |
| Gh_D12G0871 | CYP77A3 | *Gh_CYP77A3_672* | + | 1,560 | 519 | 59.224 | 19.5 | 9.798 | -0.146 | 1,560 | 1,560 | 42.2 | 1 | 1,560.00 | No intron | cyto |
