# Supplementary material for: Knockdown of Cytochrome P450 Genes Gh_D07G1197 and Gh_A13G2057 on Chromosomes D07 and A13 Reveals Their Putative Role in Enhancing Drought and Salt Stress Tolerance in Gossypium hirsutum
Source: Genes (Basel). 2019 Mar 18;10(3):226. doi: 10.3390/genes10030226 (PMC6471685; doi:10.3390/genes10030226)
Supplement: Supplementary file 1 [file genes-10-00226-s001.zip › Supplementary files/Table S3.docx]

Supplementary Table S3: Details of the diploid cotton, *G. arboreum* cytochrome P450 proteins, their physiochemical properties and gene annotation as analysed through phylogenetic tree

| **Gene ID** | **Gene Name** | **annotation** | **Chromosome** | **Start** | **End** | **Strand** | **Length (bp)** | **Protein Length (aa)** | **Molecular Weight (kDa)** | **Charge** | **Isoelectric Point** | **Grand Average of Hydropathy** | **Transcript Length (bp)** | **CDS Length (bp)** | **CDS GC Content (%)** | **Exon Number** | **Mean Exon Length (bp)** | **Mean Intron Length (bp)** | **Wolfsport subcellular localization** |
| --- | --- | --- | --- | --- | --- | --- | --- | --- | --- | --- | --- | --- | --- | --- | --- | --- | --- | --- | --- |
| Ga05G0726 | CYP75A1 | Ga_CYP75A1_1 | Chr05 | 6,286,341 | 6,288,077 | - | 1,737 | 527 | 59.612 | 15 | 9.412 | -0.087 | 1,584 | 1,584 | 47.2 | 3 | 528 | 76.5 | nucl |
| Ga12G0754 | CYP76B6 | Ga_CYP76B6_2 | Chr12 | 7,093,355 | 7,096,214 | + | 2,860 | 488 | 55.861 | 10 | 8.221 | -0.205 | 1,467 | 1,467 | 46.3 | 2 | 733.5 | 1,393.00 | plas |
| Ga12G0755 | CYP76B6 | Ga_CYP76B6_3 | Chr12 | 7,096,542 | 7,098,112 | + | 1,571 | 499 | 56.712 | 11.5 | 8.855 | -0.113 | 1,500 | 1,500 | 44 | 2 | 750 | 71 | nucl |
| Ga09G2170 | CYP76B6 | Ga_CYP76B6_4 | Chr09 | 79,410,288 | 79,419,239 | - | 8,952 | 500 | 56.386 | 4.5 | 7.561 | -0.059 | 1,503 | 1,503 | 41.3 | 2 | 751.5 | 7,449.00 | nucl |
| Ga09G2174 | CYP76B6 | Ga_CYP76B6_5 | Chr09 | 79,463,383 | 79,477,238 | - | 13,856 | 473 | 53.155 | 5 | 7.554 | -0.044 | 1,422 | 1,422 | 42.1 | 5 | 284.4 | 3,108.50 | E.R. |
| Ga08G2078 | CYP76B6 | Ga_CYP76B6_6 | Chr08 | 118,530,315 | 118,531,881 | + | 1,567 | 453 | 51.896 | 10 | 8.09 | -0.171 | 1,362 | 1,362 | 42.1 | 3 | 454 | 102.5 | nucl |
| Ga08G2076 | CYP76B6 | Ga_CYP76B6_7 | Chr08 | 118,494,474 | 118,496,052 | + | 1,579 | 490 | 55.818 | 6.5 | 7.439 | -0.056 | 1,473 | 1,473 | 41.1 | 3 | 491 | 53 | nucl |
| Ga08G2073 | CYP76B6 | Ga_CYP76B6_8 | Chr08 | 118,445,720 | 118,446,948 | + | 1,229 | 380 | 43.369 | -3 | 5.915 | -0.177 | 1,143 | 1,143 | 39.1 | 2 | 571.5 | 86 | nucl |
| Ga08G2075 | CYP76B6 | Ga_CYP76B6_9 | Chr08 | 118,469,368 | 118,481,952 | + | 12,585 | 497 | 56.585 | 6 | 7.666 | -0.067 | 1,494 | 1,494 | 41.1 | 2 | 747 | 11,091.00 | cyto |
| Ga11G0096 | CYP76A2 | Ga_CYP76A2_10 | Chr11 | 656,997 | 658,970 | + | 1,974 | 513 | 58.344 | 20.5 | 9.546 | -0.184 | 1,542 | 1,542 | 45.5 | 2 | 771 | 432 | cyto |
| Ga11G0098 | CYP76B10 | Ga_CYP76B10_11 | Chr11 | 691,173 | 693,090 | + | 1,918 | 434 | 49.269 | 12.5 | 9.017 | -0.078 | 1,305 | 1,305 | 44.1 | 5 | 261 | 153.3 | nucl |
| Ga09G1557 | CYP76A2 | Ga_CYP76A2_12 | Chr09 | 72,739,959 | 72,741,143 | + | 1,185 | 299 | 33.919 | -2.5 | 5.755 | -0.175 | 900 | 900 | 45.2 | 4 | 225 | 95 | nucl |
| Ga09G1627 | CYP76A2 | Ga_CYP76A2_13 | Chr09 | 73,710,207 | 73,710,575 | - | 369 | 122 | 13.621 | 11 | 10.281 | 0.18 | 369 | 369 | 40.4 | 1 | 369 | No intron | extr |
| Ga04G0935 | CYP76A2 | Ga_CYP76A2_14 | Chr04 | 29,844,439 | 29,846,090 | + | 1,652 | 485 | 55.14 | 12.5 | 9.362 | -0.277 | 1,458 | 1,458 | 45.1 | 3 | 486 | 97 | E.R. |
| Ga04G0936 | CYP76A2 | Ga_CYP76A2_15 | Chr04 | 29,891,479 | 29,893,090 | + | 1,612 | 512 | 58.325 | 13.5 | 9.119 | -0.141 | 1,539 | 1,539 | 44.8 | 2 | 769.5 | 73 | E.R. |
| Ga08G2029 | CYP76B6 | Ga_CYP76B6_16 | Chr08 | 117,456,163 | 117,457,791 | - | 1,629 | 493 | 55.869 | 16.5 | 9.493 | -0.088 | 1,482 | 1,482 | 43.7 | 3 | 494 | 73.5 | plas |
| Ga01G2651 | CYP75B1 | Ga_CYP75B1_17 | Chr01 | 110,708,076 | 110,709,786 | + | 1,711 | 536 | 60.119 | 4 | 7.524 | -0.122 | 1,611 | 1,611 | 43.8 | 2 | 805.5 | 100 | nucl |
| Ga01G2647 | CYP736A12 | Ga_ CYP736A12_18 | Chr01 | 110,654,671 | 110,657,650 | + | 2,980 | 532 | 60.203 | 14 | 9.21 | -0.083 | 1,599 | 1,599 | 42.2 | 2 | 799.5 | 1,381.00 | nucl |
| Ga01G2646 | CYP75A2 | Ga_CYP75A2_19 | Chr01 | 110,651,464 | 110,654,563 | + | 3,100 | 538 | 60.599 | 7 | 8.197 | -0.036 | 1,617 | 1,617 | 43.1 | 3 | 539 | 741.5 | nucl |
| Ga01G2648 | CYP75A2 | Ga_CYP75A2_20 | Chr01 | 110,662,006 | 110,663,540 | + | 1,535 | 484 | 54.73 | 4.5 | 7.588 | -0.124 | 1,455 | 1,455 | 43.4 | 2 | 727.5 | 80 | nucl |
| Ga01G2649 | CYP75A2 | Ga_CYP75A2_21 | Chr01 | 110,674,227 | 110,675,889 | - | 1,663 | 527 | 59.707 | 13.5 | 9.592 | -0.129 | 1,584 | 1,584 | 41.9 | 2 | 792 | 79 | nucl |
| Ga01G2650 | CYP76B6 | Ga_CYP76B6_22 | Chr01 | 110,703,100 | 110,703,854 | - | 755 | 249 | 27.764 | 3 | 8.065 | 0.148 | 750 | 750 | 43.6 | 2 | 375 | 5 | nucl |
| Ga12G0582 | CYP93A1 | Ga_CYP93A1_23 | Chr12 | 5,019,052 | 5,021,039 | - | 1,988 | 502 | 56.373 | 5 | 7.704 | -0.046 | 1,509 | 1,509 | 46.4 | 2 | 754.5 | 479 | E.R. |
| Ga12G0137 | CYP93A1 | Ga_CYP93A1_24 | Chr12 | 1,142,832 | 1,145,383 | - | 2,552 | 530 | 60.489 | 14.5 | 9.661 | -0.025 | 1,593 | 1,593 | 44.8 | 2 | 796.5 | 959 | nucl |
| Ga12G0138 | CYP76C3 | Ga_CYP76C3_25 | Chr12 | 1,148,658 | 1,151,413 | - | 2,756 | 531 | 60.378 | 9.5 | 8.945 | -0.046 | 1,596 | 1,596 | 44.9 | 2 | 798 | 1,160.00 | nucl |
| Ga12G0139 | CYP76AH1 | Ga_CYP76AH1_26 | Chr12 | 1,154,949 | 1,158,078 | - | 3,130 | 530 | 60.529 | 14 | 9.323 | -0.011 | 1,593 | 1,593 | 44.2 | 2 | 796.5 | 1,537.00 | nucl |
| Ga10G0401 | CYP93A2 | Ga_CYP93A2_27 | Chr10 | 6,195,174 | 6,197,923 | - | 2,750 | 529 | 59.822 | 15 | 9.625 | -0.062 | 1,590 | 1,590 | 46.5 | 2 | 795 | 1,160.00 | E.R. |
| Ga12G0136 | CYP76AH1 | Ga_CYP76AH1_28 | Chr12 | 1,133,717 | 1,136,889 | - | 3,173 | 531 | 60.172 | 14.5 | 9.538 | -0.028 | 1,596 | 1,596 | 45.4 | 2 | 798 | 1,577.00 | nucl |
| Ga11G4049 | CYP736A12 | Ga_CYP736A12_29 | Chr11 | 123,771,183 | 123,772,855 | + | 1,673 | 457 | 51.549 | 7 | 8.135 | -0.023 | 1,374 | 1,374 | 44.1 | 3 | 458 | 149.5 | nucl |
| Ga11G4050 | CYP76C3 | Ga_CYP76C3_30 | Chr11 | 123,777,553 | 123,793,526 | + | 15,974 | 721 | 82.447 | 10.5 | 8.202 | -0.079 | 2,166 | 2,166 | 43.6 | 7 | 309.4 | 2,301.30 | E.R. |
| Ga05G4278 | CYP76A2 | Ga_CYP76A2_31 | Chr05 | 96,899,391 | 96,900,953 | - | 1,563 | 426 | 47.994 | -1 | 6.325 | -0.039 | 1,281 | 1,281 | 44.7 | 3 | 427 | 141 | E.R. |
| Ga05G4078 | CYP736A12 | Ga_CYP736A12_32 | Chr05 | 93,619,145 | 93,620,792 | + | 1,648 | 444 | 50.081 | 5.5 | 7.548 | -0.023 | 1,335 | 1,335 | 46.4 | 3 | 445 | 156.5 | E.R. |
| Ga05G4080 | CYP736A12 | Ga_CYP736A12_33 | Chr05 | 93,706,632 | 93,708,279 | + | 1,648 | 444 | 49.951 | 6 | 8.062 | -0.021 | 1,335 | 1,335 | 45.8 | 3 | 445 | 156.5 | E.R. |
| Ga12G1793 | CYP703A2 | Ga_CYP703A2_34 | Chr12 | 28,913,982 | 28,915,974 | + | 1,993 | 524 | 59.805 | 10.5 | 7.697 | -0.209 | 1,575 | 1,575 | 47.6 | 2 | 787.5 | 418 | E.R. |
| Ga05G2980 | CYP79A2 | Ga_CYP79A2_35 | Chr05 | 32,161,913 | 32,163,671 | - | 1,759 | 532 | 60.05 | 9.5 | 8.256 | -0.161 | 1,599 | 1,599 | 44.5 | 3 | 533 | 80 | nucl |
| Ga10G0923 | CYP79A2 | Ga_CYP79A2_36 | Chr10 | 20,126,039 | 20,127,912 | + | 1,874 | 555 | 62.515 | 12.5 | 8.718 | -0.159 | 1,668 | 1,668 | 47 | 2 | 834 | 206 | E.R. |
| Ga05G1159 | CYP79D4 | Ga_CYP79D4_37 | Chr05 | 10,125,806 | 10,128,052 | + | 2,247 | 550 | 61.381 | 5.5 | 7.327 | -0.02 | 1,653 | 1,653 | 49.1 | 2 | 826.5 | 594 | plas |
| Ga07G2226 | CYP79D4 | Ga_CYP79D4_38 | Chr07 | 89,865,403 | 89,867,249 | + | 1,847 | 488 | 55.261 | 10.5 | 8.257 | -0.158 | 1,467 | 1,467 | 44.6 | 2 | 733.5 | 380 | nucl |
| Ga07G2230 | CYP79D4 | Ga_CYP79D4_39 | Chr07 | 90,358,525 | 90,360,331 | + | 1,807 | 527 | 59.733 | 11 | 8.128 | -0.153 | 1,584 | 1,584 | 44.1 | 3 | 528 | 111.5 | mito |
| Ga07G2228 | CYP79D4 | Ga_CYP79D4_40 | Chr07 | 89,966,490 | 89,968,307 | + | 1,818 | 537 | 60.498 | -2.5 | 6.241 | -0.219 | 1,614 | 1,614 | 44.2 | 3 | 538 | 102 | mito |
| Ga14G1688 | CYP79D4 | Ga_CYP79D4_41 | tig00015726 | 175,548 | 177,364 | + | 1,817 | 545 | 61.696 | 7 | 7.257 | -0.206 | 1,638 | 1,638 | 44.1 | 2 | 819 | 179 | nucl |
| Ga14G2330 | CYP73A100 | Ga_CYP73A100_42 | tig00019866 | 270,972 | 272,051 | + | 1,080 | 359 | 41.506 | 6.5 | 7.721 | -0.272 | 1,080 | 1,080 | 47.1 | 1 | 1,080.00 | No intron | nucl |
| Ga10G0968 | CYP73A11 | Ga_CYP73A11_43 | Chr10 | 21,301,307 | 21,303,566 | + | 2,260 | 483 | 55.766 | 11.5 | 9.145 | -0.216 | 1,452 | 1,452 | 46.2 | 4 | 363 | 269.3 | E.R. |
| Ga13G2841 | CYP73A11 | Ga_CYP73A11_44 | Chr13 | 123,822,672 | 123,824,838 | + | 2,167 | 505 | 58.066 | 13 | 9.454 | -0.258 | 1,518 | 1,518 | 48.1 | 3 | 506 | 324.5 | E.R. |
| Ga05G2108 | KO | Ga_KO_45 | Chr05 | 19,462,573 | 19,474,159 | + | 11,587 | 480 | 55.106 | 7 | 7.84 | -0.207 | 1,443 | 1,443 | 42.9 | 11 | 131.2 | 1,014.40 | E.R. |
| Ga06G0168 | KO | Ga_KO_46 | Chr06 | 1,427,459 | 1,430,483 | + | 3,025 | 502 | 57.759 | 10 | 8.269 | -0.319 | 1,509 | 1,509 | 44.5 | 7 | 215.6 | 252.7 | E.R. |
| Ga02G0537 | CYP89A2 | Ga_CYP89A2_47 | Chr02 | 10,170,584 | 10,171,502 | + | 919 | 250 | 28.746 | 13.5 | 9.759 | 0.091 | 753 | 753 | 42.8 | 2 | 376.5 | 166 | chlo |
| Ga02G0538 | CYP89A2 | Ga_CYP89A2_48 | Chr02 | 10,177,836 | 10,180,343 | + | 2,508 | 414 | 48.122 | 21 | 9.84 | -0.316 | 1,245 | 1,245 | 42.5 | 5 | 249 | 315.8 | mito |
| Ga13G1229 | CYP89A2 | Ga_CYP89A2_49 | Chr13 | 68,758,174 | 68,759,727 | + | 1,554 | 517 | 59.556 | 20.5 | 10.007 | -0.165 | 1,554 | 1,554 | 44 | 1 | 1,554.00 | No intron | nucl |
| Ga02G0707 | CYP89A2 | Ga_CYP89A2_50 | Chr02 | 28,688,928 | 28,690,466 | - | 1,539 | 512 | 59.196 | 16.5 | 9.649 | -0.164 | 1,539 | 1,539 | 43.3 | 1 | 1,539.00 | No intron | plas |
| Ga08G2596 | CYP89A9 | Ga_CYP89A9_51 | Chr08 | 126,158,036 | 126,159,580 | + | 1,545 | 514 | 59.125 | 14.5 | 9.372 | -0.201 | 1,545 | 1,545 | 43.9 | 1 | 1,545.00 | No intron | nucl |
| Ga11G0367 | CYP89A2 | Ga_CYP89A2_52 | Chr11 | 4,753,057 | 4,754,583 | - | 1,527 | 508 | 58.509 | 9.5 | 8.318 | -0.075 | 1,527 | 1,527 | 42.4 | 1 | 1,527.00 | No intron | nucl |
| Ga11G0365 | CYP89A2 | Ga_CYP89A2_53 | Chr11 | 4,740,246 | 4,741,784 | - | 1,539 | 512 | 58.709 | 14 | 9.648 | -0.159 | 1,539 | 1,539 | 42.8 | 1 | 1,539.00 | No intron | E.R. |
| Ga11G0366 | CYP89A2 | Ga_CYP89A2_54 | Chr11 | 4,746,192 | 4,747,721 | - | 1,530 | 509 | 58.82 | 17 | 9.673 | -0.155 | 1,530 | 1,530 | 42.9 | 1 | 1,530.00 | No intron | nucl |
| Ga06G1800 | CYP77A3 | Ga_CYP77A3_55 | Chr06 | 117,985,339 | 117,986,856 | + | 1,518 | 505 | 57.042 | 13 | 8.801 | -0.017 | 1,518 | 1,518 | 48.9 | 1 | 1,518.00 | No intron | mito |
| Ga06G2371 | CYP77A3 | Ga_CYP77A3_56 | Chr06 | 129,738,254 | 129,739,774 | - | 1,521 | 506 | 57.258 | 11.5 | 8.611 | -0.053 | 1,521 | 1,521 | 47 | 1 | 1,521.00 | No intron | nucl |
| Ga10G2946 | CYP77A3 | Ga_CYP77A3_57 | Chr10 | 128,296,039 | 128,297,568 | + | 1,530 | 509 | 57.836 | 9.5 | 8.396 | -0.042 | 1,530 | 1,530 | 47.2 | 1 | 1,530.00 | No intron | cyto |
| Ga10G0660 | CYP77A3 | Ga_CYP77A3_58 | Chr10 | 12,227,230 | 12,228,746 | + | 1,517 | 495 | 56.069 | 5.5 | 7.768 | -0.176 | 1,488 | 1,488 | 47.4 | 2 | 744 | 29 | E.R. |
| Ga12G2052 | CYP77A3 | Ga_CYP77A3_59 | Chr12 | 45,319,446 | 45,321,005 | - | 1,560 | 519 | 59.091 | 19 | 9.721 | -0.13 | 1,560 | 1,560 | 41.8 | 1 | 1,560.00 | No intron | cyto |
| Ga06G2160 | CYP78A7 | Ga_CYP78A7_60 | Chr06 | 126,443,446 | 126,446,569 | - | 3,124 | 559 | 63.506 | 18 | 8.865 | -0.143 | 1,680 | 1,680 | 40.1 | 7 | 240 | 240.7 | nucl |
| Ga14G1625 | [CYP74A3](https://www.uniprot.org/uniprot/Q6Z6L1) | Ga_CYP74A3_61 | tig00015610 | 323,563 | 325,023 | + | 1,461 | 486 | 54.742 | 3 | 7.189 | -0.042 | 1,461 | 1,461 | 42.8 | 1 | 1,461.00 | No intron | nucl |
| Ga06G0149 | AOS1 | Ga_AOS1_62 | Chr06 | 1,163,099 | 1,164,586 | + | 1,488 | 495 | 55.779 | 4.5 | 7.949 | -0.164 | 1,488 | 1,488 | 42.3 | 1 | 1,488.00 | No intron | plas |
| Ga03G1170 | AOS1 | Ga_AOS1_63 | Chr03 | 55,010,408 | 55,011,940 | + | 1,533 | 510 | 57.413 | 8.5 | 8.662 | -0.18 | 1,533 | 1,533 | 46.8 | 1 | 1,533.00 | No intron | nucl |
| Ga05G2768 | AOS1 | Ga_AOS1_64 | Chr05 | 28,187,053 | 28,188,624 | + | 1,572 | 523 | 58.77 | 18 | 9.812 | -0.242 | 1,572 | 1,572 | 46.6 | 1 | 1,572.00 | No intron | E.R. |
| Ga06G0150 | AOS1 | Ga_AOS1_65 | Chr06 | 1,168,987 | 1,170,477 | + | 1,491 | 496 | 55.838 | 0.5 | 6.637 | -0.131 | 1,491 | 1,491 | 43.3 | 1 | 1,491.00 | No intron | E.R. |
| Ga07G2112 | CYP97B2 | Ga_CYP97B2_66 | Chr07 | 83,948,511 | 83,955,624 | - | 7,114 | 579 | 64.836 | 2.5 | 6.912 | -0.122 | 1,740 | 1,740 | 42.5 | 14 | 124.3 | 413.4 | nucl |
| Ga11G0088 | CYP97C1 | Ga_CYP97C1_67 | Chr11 | 603,257 | 607,505 | + | 4,249 | 548 | 61.4 | 1.5 | 6.874 | -0.134 | 1,647 | 1,647 | 42.4 | 9 | 183 | 325.3 | E.R. |
| Ga13G1942 | CYP97A3 | Ga_CYP97A3_68 | Chr13 | 110,943,153 | 110,948,837 | - | 5,685 | 589 | 66.124 | -8 | 5.18 | -0.197 | 1,770 | 1,770 | 42.8 | 17 | 104.1 | 244.7 | E.R. |
| Ga05G0443 | CYP711A1 | Ga_CYP711A1_69 | Chr05 | 3,926,617 | 3,929,049 | - | 2,433 | 539 | 60.618 | 16.5 | 9.431 | -0.15 | 1,620 | 1,620 | 41.3 | 5 | 324 | 203.3 | E.R. |
| Ga13G1188 | CYP749A22 | Ga_CYP749A22_70 | Chr13 | 63,100,398 | 63,102,814 | + | 2,417 | 519 | 59.58 | 10 | 8.299 | -0.06 | 1,560 | 1,560 | 39.6 | 5 | 312 | 214.3 | nucl |
| Ga13G1023 | CYP749A22 | Ga_CYP749A22_71 | Chr13 | 36,335,909 | 36,347,551 | + | 11,643 | 541 | 61.599 | 24.5 | 10.061 | -0.078 | 1,626 | 1,626 | 39.9 | 7 | 232.3 | 1,669.50 | nucl |
| Ga13G1025 | CYP749A22 | Ga_CYP749A22_72 | Chr13 | 37,745,614 | 37,753,348 | + | 7,735 | 535 | 60.651 | 20.5 | 9.876 | -0.113 | 1,608 | 1,608 | 40.7 | 6 | 268 | 1,225.40 | nucl |
| Ga10G1484 | CYP749A22 | Ga_CYP749A22_73 | Chr10 | 80,411,991 | 80,413,928 | - | 1,938 | 520 | 59.134 | 14 | 9.42 | -0.176 | 1,563 | 1,563 | 41.8 | 5 | 312.6 | 93.8 | mito |
| Ga10G1486 | CYP749A22 | Ga_CYP749A22_74 | Chr10 | 80,454,629 | 80,456,541 | - | 1,913 | 507 | 57.578 | 15 | 9.499 | -0.153 | 1,524 | 1,524 | 42.2 | 5 | 304.8 | 97.3 | E.R. |
| Ga03G0292 | CYP749A22 | Ga_ CYP749A22_75 | Chr03 | 3,128,990 | 3,130,929 | + | 1,940 | 516 | 58.578 | 15.5 | 9.356 | -0.069 | 1,551 | 1,551 | 40.1 | 5 | 310.2 | 97.3 | cyto |
| Ga03G0293 | CYP749A22 | Ga_ CYP749A22_76 | Chr03 | 3,138,230 | 3,163,235 | + | 25,006 | 516 | 58.659 | 16.5 | 9.578 | -0.107 | 1,551 | 1,551 | 40.6 | 5 | 310.2 | 5,863.80 | cyto |
| Ga03G0285 | CYP749A22 | Ga_ CYP749A22_77 | Chr03 | 2,943,375 | 2,990,899 | + | 47,525 | 475 | 53.488 | 15.5 | 9.481 | -0.083 | 1,428 | 1,428 | 39.4 | 7 | 204 | 7,682.80 | nucl |
| Ga03G0286 | CYP749A22 | Ga_ CYP749A22_78 | Chr03 | 2,992,249 | 2,994,177 | + | 1,929 | 512 | 58.348 | 17 | 9.332 | -0.052 | 1,539 | 1,539 | 38.8 | 5 | 307.8 | 97.5 | nucl |
| Ga03G0287 | CYP749A22 | Ga_ CYP749A22_79 | Chr03 | 2,996,587 | 2,998,548 | + | 1,962 | 495 | 56.708 | 18.5 | 9.651 | -0.142 | 1,488 | 1,488 | 39.9 | 6 | 248 | 94.8 | nucl |
| Ga03G0289 | CYP749A22 | Ga_ CYP749A22_80 | Chr03 | 3,016,799 | 3,019,299 | + | 2,501 | 511 | 58.476 | 20.5 | 9.694 | -0.172 | 1,536 | 1,536 | 38.3 | 5 | 307.2 | 241.3 | nucl |
| Ga03G0294 | CYP749A22 | Ga_ CYP749A22_81 | Chr03 | 3,183,939 | 3,196,697 | + | 12,759 | 517 | 59.174 | 13 | 8.671 | -0.148 | 1,554 | 1,554 | 40 | 5 | 310.8 | 2,801.30 | mito |
| Ga03G0295 | CYP749A22 | Ga_ CYP749A22_82 | Chr03 | 3,203,505 | 3,206,883 | + | 3,379 | 495 | 56.95 | 14 | 8.843 | -0.163 | 1,488 | 1,488 | 38.9 | 6 | 248 | 378.2 | E.R. |
| Ga03G0255 | CYP749A22 | Ga_ CYP749A22_83 | Chr03 | 2,646,547 | 2,649,205 | - | 2,659 | 483 | 54.98 | 19.5 | 9.489 | -0.238 | 1,452 | 1,452 | 39.3 | 7 | 207.4 | 201.2 | E.R. |
| Ga03G0288 | CYP749A22 | Ga_ CYP749A22_84 | Chr03 | 3,008,774 | 3,012,509 | + | 3,736 | 513 | 58.469 | 13 | 8.968 | -0.054 | 1,542 | 1,542 | 40.5 | 6 | 257 | 438.8 | nucl |
| Ga03G0290 | CYP749A22 | Ga_ CYP749A22_85 | Chr03 | 3,050,055 | 3,060,760 | + | 10,706 | 512 | 58.432 | 21.5 | 9.793 | -0.11 | 1,539 | 1,539 | 39.8 | 5 | 307.8 | 2,291.80 | nucl |
| Ga03G0291 | CYP749A22 | Ga_ CYP749A22_86 | Chr03 | 3,084,408 | 3,107,019 | + | 22,612 | 509 | 58.021 | 21 | 9.582 | -0.087 | 1,530 | 1,530 | 39.7 | 7 | 218.6 | 3,513.70 | mito |
| Ga13G0732 | CYP749A22 | Ga_CYP749A22_87 | Chr13 | 11,608,682 | 11,610,701 | + | 2,020 | 480 | 54.888 | 12.5 | 8.354 | -0.15 | 1,443 | 1,443 | 40.1 | 6 | 240.5 | 115.4 | nucl |
| Ga13G0726 | CYP749A22 | Ga_CYP749A22_88 | Chr13 | 11,324,663 | 11,327,192 | + | 2,530 | 513 | 58.68 | 15.5 | 8.984 | -0.13 | 1,542 | 1,542 | 39.8 | 5 | 308.4 | 247 | nucl |
| Ga13G0725 | CYP749A22 | Ga_CYP749A22_89 | Chr13 | 11,315,306 | 11,317,755 | + | 2,450 | 513 | 58.76 | 16.5 | 9.202 | -0.133 | 1,542 | 1,542 | 39.3 | 5 | 308.4 | 227 | nucl |
| Ga13G0729 | CYP749A22 | Ga_CYP749A22_90 | Chr13 | 11,392,581 | 11,395,027 | + | 2,447 | 501 | 57.429 | 15.5 | 9.077 | -0.139 | 1,506 | 1,506 | 39.2 | 6 | 251 | 188.2 | nucl |
| Ga13G0355 | CYP736A12 | Ga_CYP736A12_91 | Chr13 | 3,957,633 | 3,959,545 | + | 1,913 | 529 | 60.365 | 11.5 | 8.721 | -0.13 | 1,590 | 1,590 | 40.9 | 5 | 318 | 80.8 | E.R. |
| Ga14G1953 | CYP749A22 | Ga_CYP749A22_92 | tig00016850 | 29,013 | 30,923 | - | 1,911 | 529 | 60.504 | 11 | 8.723 | -0.128 | 1,590 | 1,590 | 40.9 | 5 | 318 | 80.3 | E.R. |
| Ga13G0361 | CYP736A12 | Ga_CYP736A12_93 | Chr13 | 4,104,995 | 4,106,902 | + | 1,908 | 529 | 60.553 | 10.5 | 8.64 | -0.132 | 1,590 | 1,590 | 40.9 | 5 | 318 | 79.5 | E.R. |
| Ga13G0360 | CYP736A12 | Ga_CYP736A12_94 | Chr13 | 4,076,312 | 4,078,220 | + | 1,909 | 529 | 60.455 | 12 | 8.994 | -0.131 | 1,590 | 1,590 | 41.4 | 5 | 318 | 79.8 | E.R. |
| Ga13G0362 | CYP736A12 | Ga_CYP736A12_95 | Chr13 | 4,136,232 | 4,138,101 | + | 1,870 | 519 | 59.293 | 12 | 8.787 | -0.113 | 1,560 | 1,560 | 40.8 | 5 | 312 | 77.5 | E.R. |
| Ga13G0358 | CYP736A12 | Ga_CYP736A12_96 | Chr13 | 4,053,791 | 4,067,143 | + | 13,353 | 519 | 59.054 | 11 | 8.618 | -0.112 | 1,560 | 1,560 | 40.9 | 6 | 260 | 2,358.60 | nucl |
| Ga14G1724 | CYP749A22 | Ga_CYP749A22_97 | tig00015727 | 311,354 | 313,398 | + | 2,045 | 513 | 58.44 | 12.5 | 9.231 | -0.18 | 1,542 | 1,542 | 41.8 | 5 | 308.4 | 125.8 | nucl |
| Ga14G1722 | CYP749A22 | Ga_CYP749A22_98 | tig00015727 | 299,221 | 303,447 | + | 4,227 | 513 | 58.225 | 15 | 9.374 | -0.141 | 1,542 | 1,542 | 41.6 | 5 | 308.4 | 671.3 | nucl |
| Ga14G1723 | CYP749A22 | Ga_CYP749A22_99 | tig00015727 | 305,381 | 309,553 | + | 4,173 | 488 | 55.431 | 11.5 | 8.691 | -0.105 | 1,467 | 1,467 | 41.1 | 5 | 293.4 | 676.5 | nucl |
| Ga12G1480 | CYP72A219 | Ga_CYP72A219_100 | Chr12 | 22,396,627 | 22,399,070 | - | 2,444 | 514 | 59.106 | 19.5 | 9.651 | -0.211 | 1,545 | 1,545 | 43 | 5 | 309 | 224.8 | nucl |
| Ga05G0352 | CYP72A15 | Ga_CYP72A15_101 | Chr05 | 3,187,582 | 3,189,503 | + | 1,922 | 517 | 59.099 | 16 | 9.079 | -0.164 | 1,554 | 1,554 | 42 | 5 | 310.8 | 92 | nucl |
| Ga08G2749 | CYP72A219 | Ga_CYP72A219_102 | Chr08 | 127,649,924 | 127,652,582 | - | 2,659 | 521 | 59.735 | 12 | 8.781 | -0.12 | 1,566 | 1,566 | 43.2 | 5 | 313.2 | 273.3 | nucl |
| Ga01G0432 | CYP72A15 | Ga_CYP72A15_103 | Chr01 | 4,417,690 | 4,419,656 | + | 1,967 | 518 | 60.084 | 14.5 | 9.014 | -0.247 | 1,557 | 1,557 | 42.5 | 5 | 311.4 | 102.5 | E.R. |
| Ga01G0429 | CYP72A15 | Ga_CYP72A15_104 | Chr01 | 4,400,066 | 4,401,955 | + | 1,890 | 483 | 55.764 | 9 | 8.345 | -0.2 | 1,452 | 1,452 | 42.4 | 5 | 290.4 | 109.5 | nucl |
| Ga01G0430 | CYP72A15 | Ga_CYP72A15_105 | Chr01 | 4,410,064 | 4,412,749 | + | 2,686 | 538 | 62.437 | 19 | 9.508 | -0.249 | 1,617 | 1,617 | 41.8 | 6 | 269.5 | 213.8 | nucl |
| Ga01G0427 | CYP72A219 | Ga_CYP72A219_106 | Chr01 | 4,368,236 | 4,371,575 | + | 3,340 | 517 | 60.025 | 5.5 | 7.806 | -0.187 | 1,554 | 1,554 | 40.6 | 6 | 259 | 357.2 | mito |
| Ga01G0428 | CYP72A219 | Ga_CYP72A219_107 | Chr01 | 4,377,576 | 4,379,459 | + | 1,884 | 506 | 58.729 | 14 | 9.283 | -0.229 | 1,521 | 1,521 | 42.1 | 6 | 253.5 | 72.6 | nucl |
| Ga01G0426 | CYP72A15 | Ga_CYP72A15_108 | Chr01 | 4,357,537 | 4,363,391 | + | 5,855 | 628 | 72.24 | 19 | 9.116 | -0.384 | 1,887 | 1,887 | 43.1 | 10 | 188.7 | 440.9 | plas |
| Ga07G0026 | CYP734A1 | Ga_CYP734A1_109 | Chr07 | 261,893 | 264,685 | - | 2,793 | 518 | 59.546 | 25.5 | 9.706 | -0.055 | 1,557 | 1,557 | 44.6 | 5 | 311.4 | 309 | plas |
| Ga06G0599 | CYP734A1 | Ga_CYP734A1_110 | Chr06 | 9,327,158 | 9,329,726 | - | 2,569 | 515 | 58.658 | 24.5 | 9.955 | 0.004 | 1,548 | 1,548 | 45 | 5 | 309.6 | 255.3 | nucl |
| Ga05G0202 | CYP734A1 | Ga_CYP734A1_111 | Chr05 | 1,801,585 | 1,804,397 | - | 2,813 | 524 | 59.859 | 26 | 10.18 | -0.03 | 1,575 | 1,575 | 44.2 | 5 | 315 | 309.5 | cyto |
| Ga08G2815 | CYP734A1 | Ga_CYP734A1_112 | Chr08 | 128,172,033 | 128,175,118 | - | 3,086 | 517 | 59.129 | 19 | 9.555 | -0.03 | 1,554 | 1,554 | 43.1 | 5 | 310.8 | 383 | plas |
| Ga01G1200 | CYP734A6 | Ga_CYP734A6_113 | Chr01 | 23,171,588 | 23,171,965 | + | 378 | 125 | 14.44 | 5 | 9.892 | -0.538 | 378 | 378 | 48.1 | 1 | 378 | No intron | nucl |
| Ga01G1471 | CYP734A1 | Ga_CYP734A1_114 | Chr01 | 47,552,272 | 47,556,342 | - | 4,071 | 493 | 56.82 | 10 | 8.903 | -0.195 | 1,482 | 1,482 | 43.3 | 5 | 296.4 | 647.3 | nucl |
| Ga05G2071 | CYP734A1 | Ga_CYP734A1_115 | Chr05 | 18,968,257 | 18,970,319 | + | 2,063 | 489 | 55.87 | 14.5 | 9.66 | -0.187 | 1,470 | 1,470 | 43.6 | 5 | 294 | 148.3 | nucl |
| Ga05G2072 | CYP734A1 | Ga_CYP734A1_116 | Chr05 | 18,974,086 | 18,976,194 | + | 2,109 | 505 | 58.155 | 16 | 9.526 | -0.229 | 1,518 | 1,518 | 43.5 | 4 | 379.5 | 197 | nucl |
| Ga11G2292 | CYP735A1 | Ga_CYP735A1_117 | Chr11 | 102,063,217 | 102,066,048 | - | 2,832 | 522 | 59.939 | 25.5 | 9.547 | -0.152 | 1,569 | 1,569 | 44 | 5 | 313.8 | 315.8 | E.R. |
| Ga12G2557 | CYP735A1 | Ga_CYP735A1_118 | Chr12 | 95,242,179 | 95,244,166 | + | 1,988 | 519 | 59.565 | 29 | 9.816 | -0.137 | 1,560 | 1,560 | 43.9 | 5 | 312 | 107 | cyto |
| Ga12G1934 | CYP714C2 | Ga_CYP714C2_119 | Chr12 | 32,305,346 | 32,307,235 | + | 1,890 | 508 | 57.542 | 12 | 8.576 | -0.069 | 1,527 | 1,527 | 41.3 | 6 | 254.5 | 72.6 | nucl |
| Ga07G2056 | CYP714C2 | Ga_CYP714C2_120 | Chr07 | 81,035,642 | 81,037,613 | + | 1,972 | 516 | 57.872 | 17.5 | 9.402 | 0.018 | 1,551 | 1,551 | 44.8 | 5 | 310.2 | 105.3 | nucl |
| Ga07G2057 | CYP714C2 | Ga_CYP714C2_121 | Chr07 | 81,071,401 | 81,080,797 | + | 9,397 | 487 | 55.119 | 13.5 | 9.004 | 0.032 | 1,464 | 1,464 | 43.4 | 7 | 209.1 | 1,322.20 | E.R. |
| Ga04G1903 | CYP714C2 | Ga_CYP714C2_122 | Chr04 | 95,253,486 | 95,255,419 | + | 1,934 | 513 | 57.712 | 15.5 | 8.909 | -0.079 | 1,542 | 1,542 | 42.7 | 5 | 308.4 | 98 | cyto |
| Ga13G0190 | CYP714C2 | Ga_CYP714C2_123 | Chr13 | 1,836,755 | 1,841,054 | + | 4,300 | 513 | 57.94 | 18 | 9.443 | 0.012 | 1,542 | 1,542 | 43.1 | 5 | 308.4 | 689.5 | nucl |
| Ga10G0643 | CYP714B2 | Ga_CYP714B2_124 | Chr10 | 11,706,798 | 11,708,662 | + | 1,865 | 510 | 57.326 | 15 | 8.846 | 0.007 | 1,533 | 1,533 | 42.8 | 5 | 306.6 | 83 | cyto |
| Ga05G1029 | CYP714C2 | Ga_CYP714C2_125 | Chr05 | 8,905,818 | 8,907,729 | - | 1,912 | 511 | 58.311 | 7 | 8.119 | 0.056 | 1,536 | 1,536 | 43.7 | 5 | 307.2 | 94 | nucl |
| Ga04G2017 | CYP714A1 | Ga_CYP714A1_126 | Chr04 | 97,010,405 | 97,013,303 | - | 2,899 | 519 | 58.526 | 13.5 | 8.764 | -0.097 | 1,560 | 1,560 | 46.5 | 5 | 312 | 334.8 | nucl |
| Ga05G0674 | CYP714A1 | Ga_CYP714A1_127 | Chr05 | 5,930,570 | 5,934,689 | + | 4,120 | 523 | 58.832 | 21 | 9.548 | -0.067 | 1,572 | 1,572 | 44.1 | 5 | 314.4 | 637 | cyto |
| Ga09G0570 | CYP714A1 | Ga_CYP714A1_128 | Chr09 | 32,288,026 | 32,291,766 | - | 3,741 | 459 | 53.056 | 17.5 | 9.609 | -0.19 | 1,380 | 1,380 | 43 | 4 | 345 | 787 | nucl |
| Ga09G2032 | CYP709B1 | Ga_CYP709B1_129 | Chr09 | 78,227,298 | 78,229,339 | + | 2,042 | 521 | 59.493 | 27 | 10.112 | -0.168 | 1,566 | 1,566 | 44.8 | 4 | 391.5 | 158.7 | nucl |
| Ga12G0220 | CYP735A2 | Ga_CYP735A2_130 | Chr12 | 1,767,393 | 1,772,776 | + | 5,384 | 519 | 59.168 | 15.5 | 9.398 | -0.064 | 1,560 | 1,560 | 42.6 | 4 | 390 | 1,274.70 | nucl |
| Ga10G0688 | CYP94A1 | Ga_CYP94A1_131 | Chr10 | 12,663,640 | 12,665,145 | - | 1,506 | 501 | 57.822 | 10.5 | 8.451 | -0.199 | 1,506 | 1,506 | 42.6 | 1 | 1,506.00 | No intron | E.R. |
| Ga08G0152 | CYP94A1 | Ga_CYP94A1_132 | Chr08 | 1,136,262 | 1,137,827 | - | 1,566 | 521 | 59.709 | 11.5 | 8.322 | -0.144 | 1,566 | 1,566 | 44.8 | 1 | 1,566.00 | No intron | plas |
| Ga07G2443 | CYP94A1 | Ga_CYP94A1_133 | Chr07 | 93,781,712 | 93,783,235 | + | 1,524 | 507 | 58.158 | 10 | 8.658 | -0.182 | 1,524 | 1,524 | 46.9 | 1 | 1,524.00 | No intron | cyto |
| Ga07G2444 | CYP94A1 | Ga_CYP94A1_134 | Chr07 | 93,794,006 | 93,795,529 | + | 1,524 | 507 | 58.158 | 10 | 8.658 | -0.182 | 1,524 | 1,524 | 46.9 | 1 | 1,524.00 | No intron | cyto |
| Ga07G2445 | CYP94A1 | Ga_CYP94A1_135 | Chr07 | 93,809,959 | 93,811,482 | + | 1,524 | 507 | 58.158 | 10 | 8.658 | -0.182 | 1,524 | 1,524 | 46.9 | 1 | 1,524.00 | No intron | cyto |
| Ga14G0616 | CYP94A1 | Ga_CYP94A1_136 | tig00007236 | 13,296 | 14,819 | - | 1,524 | 507 | 58.158 | 10 | 8.658 | -0.182 | 1,524 | 1,524 | 46.9 | 1 | 1,524.00 | No intron | cyto |
| Ga14G0617 | CYP94A1 | Ga_CYP94A1_137 | tig00007236 | 25,576 | 27,099 | - | 1,524 | 507 | 58.158 | 10 | 8.658 | -0.182 | 1,524 | 1,524 | 46.9 | 1 | 1,524.00 | No intron | cyto |
| Ga08G2220 | CYP94A1 | Ga_CYP94A1_138 | Chr08 | 120,934,235 | 120,935,755 | + | 1,521 | 506 | 58.297 | 10.5 | 8.995 | -0.185 | 1,521 | 1,521 | 45.5 | 1 | 1,521.00 | No intron | E.R. |
| Ga08G0092 | CYP94C1 | Ga_CYP94C1_139 | Chr08 | 669,732 | 671,285 | + | 1,554 | 517 | 58.838 | 14 | 8.763 | -0.032 | 1,554 | 1,554 | 41 | 1 | 1,554.00 | No intron | vacu |
| Ga05G0887 | CYP94C1 | Ga_CYP94C1_140 | Chr05 | 7,700,809 | 7,702,323 | - | 1,515 | 504 | 57.439 | 13 | 8.643 | -0.043 | 1,515 | 1,515 | 44.9 | 1 | 1,515.00 | No intron | nucl |
| Ga09G1115 | CYP94C1 | Ga_CYP94C1_141 | Chr09 | 64,937,998 | 64,939,500 | + | 1,503 | 500 | 57.222 | 6 | 7.499 | -0.042 | 1,503 | 1,503 | 43.9 | 1 | 1,503.00 | No intron | nucl |
| Ga09G1578 | CYP94C1 | Ga_CYP94C1_142 | Chr09 | 73,117,440 | 73,118,978 | - | 1,539 | 512 | 58.991 | 17.5 | 8.899 | -0.057 | 1,539 | 1,539 | 44.4 | 1 | 1,539.00 | No intron | E.R. |
| Ga04G1028 | CYP94C1 | Ga_CYP94C1_143 | Chr04 | 40,924,825 | 40,926,327 | - | 1,503 | 500 | 57.237 | 13 | 8.682 | -0.006 | 1,503 | 1,503 | 46.8 | 1 | 1,503.00 | No intron | plas |
| Ga02G1584 | CYP94C1 | Ga_CYP94C1_144 | Chr02 | 96,747,461 | 96,748,930 | + | 1,470 | 489 | 56.316 | 9 | 8.282 | -0.002 | 1,470 | 1,470 | 37.1 | 1 | 1,470.00 | No intron | plas |
| Ga01G2175 | CYP94B3 | Ga_CYP94B3_145 | Chr01 | 102,375,634 | 102,377,130 | - | 1,497 | 498 | 56.856 | 10.5 | 8.473 | -0.027 | 1,497 | 1,497 | 45.1 | 1 | 1,497.00 | No intron | mito |
| Ga11G3703 | CYP94B3 | Ga_CYP94B3_146 | Chr11 | 120,964,630 | 120,966,177 | + | 1,548 | 515 | 58.123 | 7 | 8.006 | -0.058 | 1,548 | 1,548 | 48.6 | 1 | 1,548.00 | No intron | nucl |
| Ga04G1232 | CYP94B3 | Ga_CYP94B3_147 | Chr04 | 75,326,912 | 75,328,438 | - | 1,527 | 508 | 57.732 | 7.5 | 8.008 | 0.008 | 1,527 | 1,527 | 43.9 | 1 | 1,527.00 | No intron | nucl |
| Ga09G0479 | CYP94B3 | Ga_CYP94B3_148 | Chr09 | 16,882,650 | 16,884,178 | - | 1,529 | 498 | 57.041 | 15 | 8.921 | -0.045 | 1,497 | 1,497 | 43.6 | 2 | 748.5 | 32 | nucl |
| Ga01G2205 | CYP86A1 | Ga_CYP86A1_149 | Chr01 | 103,302,034 | 103,303,569 | + | 1,536 | 511 | 58.858 | 11 | 9.131 | -0.211 | 1,536 | 1,536 | 46.9 | 1 | 1,536.00 | No intron | nucl |
| Ga05G0946 | CYP86A1 | Ga_CYP86A1_150 | Chr05 | 8,281,320 | 8,282,855 | - | 1,536 | 511 | 58.536 | 15 | 9.494 | -0.144 | 1,536 | 1,536 | 47 | 1 | 1,536.00 | No intron | E.R. |
| Ga07G1110 | CYP86A1 | Ga_CYP86A1_151 | Chr07 | 16,500,151 | 16,501,695 | + | 1,545 | 514 | 58.648 | 21 | 9.905 | -0.186 | 1,545 | 1,545 | 46.5 | 1 | 1,545.00 | No intron | E.R. |
| Ga08G1573 | CYP86A8 | Ga_CYP86A8_152 | Chr08 | 104,689,212 | 104,690,795 | - | 1,584 | 527 | 60.041 | 10 | 7.996 | -0.057 | 1,584 | 1,584 | 48.2 | 1 | 1,584.00 | No intron | nucl |
| Ga11G3121 | CYP86A8 | Ga_CYP86A8_153 | Chr11 | 115,952,456 | 115,954,042 | + | 1,587 | 528 | 59.797 | 8 | 7.898 | -0.115 | 1,587 | 1,587 | 49.6 | 1 | 1,587.00 | No intron | nucl |
| Ga08G2156 | CYP86A22 | Ga_CYP86A22_154 | Chr08 | 120,146,207 | 120,147,811 | - | 1,605 | 534 | 60.979 | 12 | 8.521 | -0.144 | 1,605 | 1,605 | 44.7 | 1 | 1,605.00 | No intron | nucl |
| Ga12G0500 | CYP86A22 | Ga_CYP86A22_155 | Chr12 | 4,275,639 | 4,277,222 | + | 1,584 | 527 | 60.174 | 9.5 | 8.108 | -0.121 | 1,584 | 1,584 | 47.1 | 1 | 1,584.00 | No intron | E.R. |
| Ga14G2735 | CYP86B1 | Ga_CYP86B1_156 | tig00024743 | 21,013 | 22,587 | + | 1,575 | 524 | 60.433 | 21.5 | 9.706 | -0.073 | 1,575 | 1,575 | 42 | 1 | 1,575.00 | No intron | E.R. |
| Ga04G0419 | CYP86B1 | Ga_CYP86B1_157 | Chr04 | 5,310,230 | 5,314,517 | + | 4,288 | 644 | 74.077 | 41 | 10.02 | -0.325 | 1,935 | 1,935 | 45.4 | 5 | 387 | 588.3 | nucl |
| Ga13G0708 | CYP86B1 | Ga_CYP86B1_158 | Chr13 | 10,916,923 | 10,919,719 | + | 2,797 | 468 | 53.34 | 5 | 7.795 | -0.046 | 1,407 | 1,407 | 45.7 | 5 | 281.4 | 347.5 | E.R. |
| Ga09G0365 | CYP96A15 | Ga_CYP96A15_159 | Chr09 | 10,183,791 | 10,185,310 | + | 1,520 | 409 | 47.506 | 12.5 | 8.735 | -0.244 | 1,230 | 1,230 | 41.9 | 4 | 307.5 | 96.7 | nucl |
| Ga09G0349 | CYP96A15 | Ga_CYP96A15_160 | Chr09 | 9,437,026 | 9,438,466 | - | 1,441 | 411 | 47.519 | 15 | 8.61 | -0.289 | 1,236 | 1,236 | 41.9 | 5 | 247.2 | 51.3 | nucl |
| Ga09G0350 | CYP96A15 | Ga_CYP96A15_161 | Chr09 | 9,451,620 | 9,453,128 | - | 1,509 | 502 | 57.612 | 15 | 8.521 | -0.163 | 1,509 | 1,509 | 42.8 | 1 | 1,509.00 | No intron | nucl |
| Ga09G0362 | CYP96A15 | Ga_CYP96A15_162 | Chr09 | 9,962,581 | 9,964,091 | - | 1,511 | 435 | 49.865 | 11 | 8.39 | -0.171 | 1,308 | 1,308 | 43.3 | 3 | 436 | 101.5 | nucl |
| Ga09G0363 | CYP96A15 | Ga_CYP96A15_163 | Chr09 | 10,033,286 | 10,034,582 | - | 1,297 | 301 | 34.586 | 6.5 | 8.068 | -0.182 | 906 | 906 | 42.9 | 2 | 453 | 391 | nucl |
| Ga09G0364 | CYP96A15 | Ga_CYP96A15_164 | Chr09 | 10,124,776 | 10,125,327 | - | 552 | 183 | 21.121 | -5.5 | 4.78 | -0.11 | 552 | 552 | 39.1 | 1 | 552 | No intron | nucl |
| Ga11G1559 | CYP96A15 | Ga_CYP96A15_165 | Chr11 | 55,345,139 | 55,346,662 | - | 1,524 | 507 | 58.974 | 13.5 | 8.359 | -0.207 | 1,524 | 1,524 | 41.8 | 1 | 1,524.00 | No intron | nucl |
| Ga11G1591 | CYP96A15 | Ga_CYP96A15_166 | Chr11 | 60,416,743 | 60,418,242 | + | 1,500 | 499 | 57.606 | 17 | 8.842 | -0.075 | 1,500 | 1,500 | 42.5 | 1 | 1,500.00 | No intron | nucl |
| Ga13G0932 | CYP96A15 | Ga_CYP96A15_167 | Chr13 | 25,800,062 | 25,801,557 | + | 1,496 | 486 | 55.374 | 16.5 | 8.85 | -0.123 | 1,461 | 1,461 | 43.7 | 2 | 730.5 | 35 | E.R. |
| Ga04G1926 | CYP96A15 | Ga_CYP96A15_168 | Chr04 | 95,624,645 | 95,626,180 | - | 1,536 | 511 | 59.064 | 17 | 9.269 | -0.233 | 1,536 | 1,536 | 42.4 | 1 | 1,536.00 | No intron | cyto |
| Ga14G0173 | CYP96A15 | Ga_CYP96A15_169 | tig00000498 | 1,024,863 | 1,031,170 | - | 6,308 | 412 | 47.456 | 15 | 8.859 | -0.163 | 1,239 | 1,239 | 43.3 | 3 | 413 | 2,534.50 | plas |
| Ga12G1196 | CYP704B1 | Ga_CYP704B1_170 | Chr12 | 14,821,173 | 14,823,438 | - | 2,266 | 534 | 61.299 | 11.5 | 8.685 | -0.128 | 1,605 | 1,605 | 41.3 | 6 | 267.5 | 132.2 | nucl |
| Ga11G3200 | CYP704C1 | Ga_CYP704C1_171 | Chr11 | 116,585,783 | 116,587,852 | - | 2,070 | 506 | 58.36 | 13 | 9.035 | -0.252 | 1,521 | 1,521 | 41.5 | 6 | 253.5 | 109.8 | nucl |
| Ga11G3201 | CYP704C1 | Ga_CYP704C1_172 | Chr11 | 116,634,308 | 116,636,386 | - | 2,079 | 506 | 58.603 | 12.5 | 8.801 | -0.278 | 1,521 | 1,521 | 41.6 | 6 | 253.5 | 111.6 | nucl |
| Ga07G0483 | CYP704C1 | Ga_CYP704C1_173 | Chr07 | 5,220,494 | 5,222,826 | + | 2,333 | 562 | 65.02 | 8.5 | 7.959 | -0.101 | 1,689 | 1,689 | 41.1 | 6 | 281.5 | 128.8 | nucl |
| Ga08G1533 | CYP704C1 | Ga_CYP704C1_174 | Chr08 | 103,253,760 | 103,256,813 | + | 3,054 | 512 | 59.404 | 5 | 7.337 | -0.148 | 1,539 | 1,539 | 41.7 | 5 | 307.8 | 378.8 | nucl |
| Ga11G3202 | CYP704C1 | Ga_CYP704C1_175 | Chr11 | 116,647,336 | 116,649,263 | - | 1,928 | 523 | 60.542 | 7.5 | 7.993 | -0.111 | 1,572 | 1,572 | 39.2 | 5 | 314.4 | 89 | E.R. |
| Ga05G1584 | CYP710A1 | Ga_CYP710A1_176 | Chr05 | 14,100,564 | 14,102,072 | + | 1,509 | 502 | 57.773 | 8.5 | 8.147 | -0.104 | 1,509 | 1,509 | 48.1 | 1 | 1,509.00 | No intron | nucl |
| Ga06G1662 | CYP710A1 | Ga_CYP710A1_177 | Chr06 | 108,706,551 | 108,708,062 | - | 1,512 | 463 | 53.65 | 11.5 | 8.908 | 0.002 | 1,392 | 1,392 | 46.4 | 3 | 464 | 60 | nucl |
| Ga05G1585 | CYP710A1 | Ga_CYP710A1_178 | Chr05 | 14,137,213 | 14,138,721 | + | 1,509 | 502 | 57.708 | 6.5 | 7.793 | -0.11 | 1,509 | 1,509 | 48.2 | 1 | 1,509.00 | No intron | cyto |
| Ga03G0757 | CYP707A3 | Ga_CYP707A3_179 | Chr03 | 11,968,093 | 11,983,029 | + | 14,937 | 693 | 78.141 | 11 | 8.063 | 0.094 | 2,082 | 2,082 | 40.6 | 22 | 94.6 | 612.1 | nucl |
| Ga06G0089 | CYP707A4 | Ga_CYP707A4_180 | Chr06 | 725,454 | 731,259 | + | 5,806 | 500 | 56.743 | 7.5 | 8.088 | -0.091 | 1,503 | 1,503 | 41.5 | 10 | 150.3 | 478.1 | mito |
| Ga12G2447 | CYP707A3 | Ga_CYP707A3_181 | Chr12 | 92,254,070 | 92,256,393 | - | 2,324 | 488 | 55.309 | 12.5 | 9.074 | -0.143 | 1,467 | 1,467 | 42.4 | 10 | 146.7 | 95.2 | E.R. |
| Ga05G2148 | CYP707A4 | Ga_CYP707A4_182 | Chr05 | 19,870,386 | 19,873,258 | - | 2,873 | 478 | 54.483 | 28 | 10.117 | -0.185 | 1,437 | 1,437 | 44.2 | 9 | 159.7 | 179.5 | plas |
| Ga03G2470 | CYP707A4 | Ga_CYP707A4_183 | Chr03 | 133,326,160 | 133,330,320 | - | 4,161 | 474 | 54.087 | 23 | 9.942 | -0.152 | 1,425 | 1,425 | 41.7 | 9 | 158.3 | 342 | plas |
| Ga13G0381 | CYP707A4 | Ga_CYP707A4_184 | Chr13 | 4,456,505 | 4,459,345 | - | 2,841 | 476 | 54.606 | 18.5 | 9.508 | -0.175 | 1,431 | 1,431 | 42.5 | 9 | 159 | 176.3 | E.R. |
| Ga08G1764 | CYP707A1 | Ga_CYP707A1_185 | Chr08 | 110,350,584 | 110,353,936 | - | 3,353 | 446 | 50.726 | 15 | 9.532 | -0.227 | 1,341 | 1,341 | 43.4 | 8 | 167.6 | 287.4 | cyto |
| Ga12G0790 | CYP707A1 | Ga_CYP707A1_186 | Chr12 | 7,430,893 | 7,432,902 | + | 2,010 | 463 | 53.007 | 20.5 | 9.768 | -0.125 | 1,392 | 1,392 | 43.6 | 7 | 198.9 | 103 | plas |
| Ga01G2302 | CYP707A2 | Ga_CYP707A2_187 | Chr01 | 105,276,800 | 105,279,696 | - | 2,897 | 500 | 56.997 | 20.5 | 9.586 | -0.177 | 1,503 | 1,503 | 43.8 | 8 | 187.9 | 199.1 | plas |
| Ga07G1078 | CYP707A2 | Ga_CYP707A2_188 | Chr07 | 15,661,393 | 15,665,510 | - | 4,118 | 491 | 56.019 | 23.5 | 9.98 | -0.208 | 1,476 | 1,476 | 43.2 | 8 | 184.5 | 377.4 | plas |
| Ga13G1007 | BA13 | Ga_BA13_189 | Chr13 | 30,842,354 | 30,844,634 | + | 2,281 | 466 | 53.418 | 19 | 9.238 | -0.153 | 1,401 | 1,401 | 42.1 | 9 | 155.7 | 110 | nucl |
| Ga07G0950 | BA13 | Ga_BA13_190 | Chr07 | 12,815,606 | 12,818,557 | - | 2,952 | 466 | 53.626 | 19.5 | 9.845 | -0.159 | 1,401 | 1,401 | 36.8 | 9 | 155.7 | 193.9 | nucl |
| Ga07G2048 | BA13 | Ga_BA13_191 | Chr07 | 80,532,132 | 80,534,748 | - | 2,617 | 465 | 53.648 | 14.5 | 9.381 | -0.268 | 1,398 | 1,398 | 40.3 | 9 | 155.3 | 152.4 | nucl |
| Ga11G2042 | CYP90B1 | Ga_CYP90B1_192 | Chr11 | 96,771,604 | 96,776,381 | + | 4,778 | 492 | 56.389 | 21 | 9.671 | -0.205 | 1,479 | 1,479 | 41.6 | 9 | 164.3 | 412.4 | nucl |
| Ga04G1456 | CYP724B1 | Ga_CYP724B1_193 | Chr04 | 85,679,478 | 85,682,159 | + | 2,682 | 475 | 54.205 | 17 | 9.175 | -0.004 | 1,428 | 1,428 | 40.6 | 9 | 158.7 | 156.8 | nucl |
| Ga11G2479 | CYP724B1 | Ga_CYP724B1_194 | Chr11 | 106,097,058 | 106,100,366 | - | 3,309 | 490 | 55.593 | 14.5 | 8.402 | -0.179 | 1,473 | 1,473 | 41.7 | 9 | 163.7 | 229.5 | nucl |
| Ga06G2005 | CYP90A1 | Ga_CYP90A1_195 | Chr06 | 122,974,697 | 122,979,016 | - | 4,320 | 472 | 54.095 | 15 | 9.586 | -0.157 | 1,419 | 1,419 | 44.7 | 8 | 177.4 | 414.4 | plas |
| Ga10G0036 | CYP90A1 | Ga_CYP90A1_196 | Chr10 | 407,055 | 411,656 | + | 4,602 | 470 | 53.908 | 19.5 | 9.905 | -0.168 | 1,413 | 1,413 | 44.7 | 8 | 176.6 | 455.6 | nucl |
| Ga05G1430 | CYP90D1 | Ga_CYP90D1_197 | Chr05 | 12,635,756 | 12,639,228 | - | 3,473 | 499 | 57.498 | 14.5 | 9.286 | -0.155 | 1,500 | 1,500 | 40.7 | 8 | 187.5 | 281.9 | nucl |
| Ga02G0118 | ROT3 | Ga_ROT3_198 | Chr02 | 909,762 | 916,102 | - | 6,341 | 516 | 58.535 | 12.5 | 8.864 | -0.149 | 1,551 | 1,551 | 41 | 9 | 172.3 | 598.8 | nucl |
| Ga11G2094 | ROT3 | Ga_ROT3_199 | Chr11 | 97,780,950 | 97,784,704 | + | 3,755 | 490 | 56.112 | 14.5 | 9.306 | -0.207 | 1,473 | 1,473 | 42.5 | 9 | 163.7 | 285.3 | cyto |
| Ga07G0602 | CYP87A3 | Ga_CYP87A3_200 | Chr07 | 6,628,553 | 6,630,819 | + | 2,267 | 444 | 50.303 | 11 | 9.173 | -0.143 | 1,335 | 1,335 | 42.4 | 8 | 166.9 | 133.1 | nucl |
| Ga01G2186 | CYP87A3 | Ga_CYP87A3_201 | Chr01 | 102,638,756 | 102,643,993 | - | 5,238 | 454 | 51.814 | 12 | 8.752 | -0.147 | 1,365 | 1,365 | 42.3 | 10 | 136.5 | 430.3 | nucl |
| Ga01G0582 | CYP87A3 | Ga_CYP87A3_202 | Chr01 | 7,636,968 | 7,640,837 | - | 3,870 | 439 | 50.644 | 7 | 8.378 | -0.104 | 1,320 | 1,320 | 40.9 | 9 | 146.7 | 318.8 | nucl |
| Ga01G0584 | CYP87A3 | Ga_CYP87A3_203 | Chr01 | 7,654,576 | 7,659,063 | - | 4,488 | 462 | 53.161 | 11.5 | 8.798 | -0.173 | 1,389 | 1,389 | 41.6 | 10 | 138.9 | 344.3 | nucl |
| Ga12G0188 | CYP87A3 | Ga_CYP87A3_204 | Chr12 | 1,521,919 | 1,523,421 | - | 1,503 | 178 | 19.991 | 7.5 | 9.77 | 0.113 | 537 | 537 | 38.5 | 6 | 89.5 | 193.2 | nucl |
| Ga12G0190 | CYP87A3 | Ga_CYP87A3_205 | Chr12 | 1,536,683 | 1,543,873 | + | 7,191 | 480 | 55.057 | 20.5 | 9.363 | -0.036 | 1,443 | 1,443 | 38.6 | 8 | 180.4 | 821.1 | nucl |
| Ga12G0535 | CYP87A3 | Ga_CYP87A3_206 | Chr12 | 4,582,794 | 4,587,469 | - | 4,676 | 485 | 55.734 | 16 | 9.111 | -0.031 | 1,458 | 1,458 | 41.4 | 10 | 145.8 | 357.6 | nucl |
| Ga01G2529 | CYP87A3 | Ga_CYP87A3_207 | Chr01 | 109,225,259 | 109,262,491 | + | 37,233 | 486 | 55.823 | 23.5 | 9.926 | -0.12 | 1,461 | 1,461 | 41.5 | 10 | 146.1 | 3,974.70 | mito |
| Ga01G2530 | CYP87A3 | Ga_CYP87A3_208 | Chr01 | 109,269,818 | 109,273,210 | + | 3,393 | 461 | 52.843 | 15 | 9.355 | -0.067 | 1,386 | 1,386 | 41.5 | 9 | 154 | 250.9 | nucl |
| Ga08G1868 | KAO2 | Ga_KAO2_209 | Chr08 | 113,375,975 | 113,379,727 | - | 3,753 | 487 | 55.796 | 12.5 | 9.05 | -0.119 | 1,464 | 1,464 | 42.3 | 8 | 183 | 327 | E.R. |
| Ga08G1866 | CYP88D6 | Ga_CYP88D6_210 | Chr08 | 113,267,380 | 113,271,991 | - | 4,612 | 494 | 56.754 | 23 | 9.75 | -0.176 | 1,485 | 1,485 | 41.8 | 8 | 185.6 | 446.7 | mito |
| Ga08G1867 | CYP88D6 | Ga_CYP88D6_211 | Chr08 | 113,289,885 | 113,292,456 | - | 2,572 | 486 | 55.534 | 21 | 9.666 | -0.179 | 1,461 | 1,461 | 41.6 | 8 | 182.6 | 158.7 | mito |
| Ga09G2223 | CYP88D6 | Ga_CYP88D6_212 | Chr09 | 79,823,726 | 79,826,911 | + | 3,186 | 448 | 51.473 | 15 | 9.146 | -0.22 | 1,347 | 1,347 | 40.7 | 8 | 168.4 | 262.7 | plas |
| Ga09G2226 | CYP88D6 | Ga_CYP88D6_213 | Chr09 | 79,846,006 | 79,849,201 | + | 3,196 | 480 | 55.014 | 20.5 | 9.643 | -0.239 | 1,443 | 1,443 | 41.6 | 8 | 180.4 | 250.4 | plas |
| Ga14G0359 | CYP88D6 | Ga_CYP88D6_214 | tig00004012 | 670 | 3,866 | - | 3,197 | 480 | 55.014 | 20.5 | 9.643 | -0.239 | 1,443 | 1,443 | 41.6 | 8 | 180.4 | 250.6 | plas |
| Ga06G1925 | KAO1 | Ga_KAO1_215 | Chr06 | 120,831,740 | 120,835,269 | - | 3,530 | 494 | 57.093 | 11 | 9.171 | -0.309 | 1,485 | 1,485 | 44.4 | 8 | 185.6 | 292.1 | E.R. |
| Ga13G0130 | CYP88A | Ga_CYP88A_216 | Chr13 | 1,346,871 | 1,350,325 | - | 3,455 | 495 | 57.459 | 14 | 9.226 | -0.262 | 1,488 | 1,488 | 43.5 | 8 | 186 | 281 | cyto |
| Ga10G0909 | CYP725A2 | Ga_CYP725A2_217 | Chr10 | 19,635,345 | 19,636,956 | + | 1,612 | 484 | 55.14 | 14.5 | 8.879 | -0.103 | 1,455 | 1,455 | 44.1 | 3 | 485 | 78.5 | nucl |
| Ga06G2188 | NA | Ga_NA_218 | Chr06 | 126,937,918 | 126,941,581 | - | 3,664 | 494 | 55.747 | 13 | 9.487 | -0.059 | 1,485 | 1,485 | 42.8 | 4 | 371.3 | 726.3 | nucl |
| Ga01G0851 | CYP716B1 | Ga_CYP716B1_219 | Chr01 | 12,776,963 | 12,778,692 | - | 1,730 | 474 | 54.125 | 22 | 10.095 | -0.082 | 1,425 | 1,425 | 43.5 | 4 | 356.3 | 101.7 | nucl |
| Ga10G2951 | ASPM | Ga_ASPM_220 | Chr10 | 128,325,344 | 128,346,742 | + | 21,399 | 2,143 | 245.563 | 193.5 | 10.764 | -0.154 | 6,432 | 6,432 | 40.8 | 29 | 221.8 | 534.5 | E.R. |
| Ga10G3039 | NA | Ga_NA_221 | Chr10 | 128,948,527 | 128,951,385 | - | 2,859 | 486 | 55.344 | 16.5 | 9.709 | -0.182 | 1,461 | 1,461 | 44.7 | 3 | 487 | 699 | plas |
| Ga13G1478 | CYP716A52v2 | Ga_CYP716A52v2_222 | Chr13 | 93,158,357 | 93,159,991 | + | 1,635 | 473 | 53.433 | 9.5 | 8.667 | -0.241 | 1,422 | 1,422 | 45.9 | 4 | 355.5 | 71 | E.R. |
| Ga13G1492 | CYP716A52v2 | Ga_CYP716A52v2_223 | Chr13 | 94,870,473 | 94,872,115 | + | 1,643 | 487 | 55.014 | 10 | 8.912 | -0.203 | 1,464 | 1,464 | 46.4 | 3 | 488 | 89.5 | plas |
| Ga05G1220 | NA | Ga_NA_224 | Chr05 | 10,797,921 | 10,800,343 | - | 2,423 | 476 | 54.177 | 10.5 | 8.998 | -0.17 | 1,431 | 1,431 | 46.5 | 3 | 477 | 496 | plas |
| Ga08G0212 | NA | Ga_NA_225 | Chr08 | 1,750,838 | 1,755,509 | + | 4,672 | 480 | 54.842 | 12.5 | 8.981 | -0.114 | 1,443 | 1,443 | 42.8 | 3 | 481 | 1,614.50 | nucl |
| Ga03G2053 | CYP51G1 | Ga_CYP51G1_226 | Chr03 | 126,928,573 | 126,930,609 | + | 2,037 | 486 | 55.565 | 9.5 | 8.63 | -0.182 | 1,461 | 1,461 | 45.7 | 2 | 730.5 | 576 | nucl |
| Ga12G1444 | CYP51G1 | Ga_CYP51G1_227 | Chr12 | 20,813,468 | 20,815,838 | - | 2,371 | 486 | 55.243 | 13.5 | 9.216 | -0.163 | 1,461 | 1,461 | 44.6 | 2 | 730.5 | 910 | E.R. |
| Ga02G1473 | CYP98A2 | Ga_CYP98A2_228 | Chr02 | 94,674,565 | 94,676,513 | + | 1,949 | 476 | 54.467 | 7 | 7.442 | -0.24 | 1,431 | 1,431 | 46.1 | 5 | 286.2 | 129.5 | nucl |
| Ga01G1795 | CYP78A6 | Ga_CYP78A6_229 | Chr01 | 83,921,866 | 83,924,110 | + | 2,245 | 530 | 59.652 | 16.5 | 9.089 | 0.043 | 1,593 | 1,593 | 48.1 | 2 | 796.5 | 652 | E.R. |
| Ga08G1284 | CYP78A3 | Ga_CYP78A3_230 | Chr08 | 83,741,828 | 83,743,606 | - | 1,779 | 532 | 60.095 | 17.5 | 9.016 | -0.009 | 1,599 | 1,599 | 46.9 | 2 | 799.5 | 180 | nucl |
| Ga12G0395 | CYP78A3 | Ga_CYP78A3_231 | Chr12 | 3,305,380 | 3,307,213 | + | 1,834 | 530 | 59.918 | 11.5 | 8.478 | 0.017 | 1,593 | 1,593 | 47.1 | 2 | 796.5 | 241 | E.R. |
| Ga12G1777 | CYP78A3 | Ga_CYP78A3_232 | Chr12 | 28,465,476 | 28,468,846 | - | 3,371 | 542 | 61.008 | 16 | 9.028 | -0.036 | 1,629 | 1,629 | 44.5 | 3 | 543 | 871 | nucl |
| Ga08G1475 | CYP78A6 | Ga_CYP78A6_233 | Chr08 | 100,295,598 | 100,299,107 | - | 3,510 | 578 | 65.965 | 20.5 | 9.843 | -0.066 | 1,737 | 1,737 | 47 | 3 | 579 | 886.5 | cyto |
| Ga11G3010 | CYP78A3 | Ga_CYP78A3_234 | Chr11 | 114,403,052 | 114,404,885 | - | 1,834 | 528 | 59.182 | 13.5 | 8.555 | 0.001 | 1,587 | 1,587 | 47.4 | 3 | 529 | 123.5 | nucl |
| Ga12G1236 | CYP78A5 | Ga_CYP78A5_235 | Chr12 | 15,389,270 | 15,390,928 | + | 1,659 | 519 | 57.982 | 2.5 | 6.795 | 0.106 | 1,560 | 1,560 | 47.3 | 2 | 780 | 99 | nucl |
| Ga13G1003 | CYP78A5 | Ga_CYP78A5_236 | Chr13 | 30,375,612 | 30,377,252 | + | 1,641 | 518 | 58.089 | 10.5 | 8.177 | 0.05 | 1,557 | 1,557 | 46.8 | 2 | 778.5 | 84 | E.R. |
| Ga11G3452 | CYP78A5 | Ga_CYP78A5_237 | Chr11 | 118,803,161 | 118,803,891 | - | 731 | 241 | 26.801 | 6.5 | 9.562 | 0.111 | 726 | 726 | 46.7 | 2 | 363 | 5 | E.R. |
| Ga03G1445 | CYP78A5 | Ga_CYP78A5_238 | Chr03 | 92,259,051 | 92,260,710 | + | 1,660 | 518 | 58.836 | 8 | 7.501 | 0.028 | 1,557 | 1,557 | 45.2 | 2 | 778.5 | 103 | nucl |
| Ga04G0014 | CYP78A5 | Ga_CYP78A5_239 | Chr04 | 165,113 | 168,308 | - | 3,196 | 514 | 58.025 | 15 | 9.034 | 0.114 | 1,545 | 1,545 | 46.4 | 3 | 515 | 825.5 | mito |
| Ga02G0468 | CYP78A7 | Ga_CYP78A7_240 | Chr02 | 7,644,065 | 7,645,776 | + | 1,712 | 537 | 60.682 | 12.5 | 8.955 | -0.051 | 1,614 | 1,614 | 48.8 | 2 | 807 | 98 | plas |
| Ga06G1291 | CYP78A7 | Ga_CYP78A7_241 | Chr06 | 53,939,488 | 53,941,188 | - | 1,701 | 529 | 58.895 | 13 | 9.066 | 0.049 | 1,590 | 1,590 | 46 | 3 | 530 | 55.5 | nucl |
| Ga07G1002 | CYP82G1 | Ga_CYP82G1_242 | Chr07 | 13,684,767 | 13,688,100 | + | 3,334 | 515 | 58.748 | 9.5 | 7.889 | -0.199 | 1,548 | 1,548 | 45.7 | 2 | 774 | 1,786.00 | nucl |
| Ga07G1004 | CYP82G1 | Ga_CYP82G1_243 | Chr07 | 13,828,185 | 13,830,273 | + | 2,089 | 521 | 59.12 | 17 | 9.062 | -0.216 | 1,566 | 1,566 | 45.7 | 2 | 783 | 523 | nucl |
| Ga07G1426 | CYP82C2 | Ga_CYP82C2_244 | Chr07 | 26,521,789 | 26,522,825 | + | 1,037 | 234 | 25.881 | 22 | 11.302 | 0.309 | 705 | 705 | 46.8 | 2 | 352.5 | 332 | nucl |
| Ga07G1794 | CYP82C4 | Ga_CYP82C4_245 | Chr07 | 47,139,185 | 47,141,095 | - | 1,911 | 530 | 59.62 | 1 | 6.617 | -0.064 | 1,593 | 1,593 | 46 | 2 | 796.5 | 318 | plas |
| Ga07G1427 | CYP82N3 | Ga_CYP82N3_246 | Chr07 | 26,522,861 | 26,523,722 | + | 862 | 168 | 19.094 | -3 | 5.244 | -0.322 | 507 | 507 | 43 | 3 | 169 | 177.5 | cyto |
| Ga07G1795 | CYP82C2 | Ga_CYP82C2_247 | Chr07 | 47,141,544 | 47,141,882 | - | 339 | 112 | 12.601 | 7 | 9.844 | 0.092 | 339 | 339 | 46.9 | 1 | 339 | No intron | nucl |
| Ga05G2103 | CYP82C4 | Ga_CYP82C4_248 | Chr05 | 19,366,339 | 19,368,166 | - | 1,828 | 526 | 59.054 | 8.5 | 8.079 | -0.067 | 1,581 | 1,581 | 46.4 | 2 | 790.5 | 247 | E.R. |
| Ga11G1988 | CYP82C4 | Ga_CYP82C4_249 | Chr11 | 94,547,474 | 94,550,643 | + | 3,170 | 523 | 59.135 | 3.5 | 6.98 | -0.111 | 1,572 | 1,572 | 43.7 | 2 | 786 | 1,598.00 | E.R. |
| Ga11G1989 | CYP82A3 | Ga_CYP82A3_250 | Chr11 | 94,604,784 | 94,606,238 | + | 1,455 | 398 | 45.112 | 13 | 8.349 | 0.034 | 1,197 | 1,197 | 42.3 | 3 | 399 | 129 | E.R. |
| Ga11G1991 | CYP82C2 | Ga_CYP82C2_251 | Chr11 | 94,634,164 | 94,635,888 | + | 1,725 | 520 | 59.081 | 9.5 | 7.757 | -0.118 | 1,563 | 1,563 | 44.6 | 2 | 781.5 | 162 | E.R. |
| Ga11G1990 | CYP82C3 | Ga_CYP82C3_252 | Chr11 | 94,606,266 | 94,607,443 | + | 1,178 | 198 | 22.073 | 7 | 9.298 | -0.242 | 597 | 597 | 49.1 | 3 | 199 | 290.5 | nucl |
| Ga05G2770 | CYP82A3 | Ga_CYP82A3_253 | Chr05 | 28,194,371 | 28,196,317 | - | 1,947 | 526 | 59.79 | 11.5 | 8.531 | -0.207 | 1,581 | 1,581 | 47.2 | 2 | 790.5 | 366 | plas |
| Ga01G1459 | CYP82C4 | Ga_CYP82C4_254 | Chr01 | 47,226,493 | 47,228,135 | - | 1,643 | 508 | 58.116 | 11 | 8.279 | -0.18 | 1,527 | 1,527 | 43 | 3 | 509 | 58 | plas |
| Ga05G2098 | CYP82A4 | Ga_CYP82A4_255 | Chr05 | 19,148,585 | 19,150,042 | - | 1,458 | 383 | 43.359 | 5.5 | 7.371 | -0.318 | 1,152 | 1,152 | 46.9 | 4 | 288 | 102 | plas |
| Ga06G0158 | CYP82A4 | Ga_CYP82A4_256 | Chr06 | 1,297,420 | 1,322,995 | - | 25,576 | 998 | 113.396 | 22 | 8.496 | -0.141 | 2,997 | 2,997 | 42.2 | 6 | 499.5 | 4,515.80 | E.R. |
| Ga06G0160 | CYP82A3 | Ga_CYP82A3_257 | Chr06 | 1,354,402 | 1,356,083 | - | 1,682 | 467 | 52.623 | 12.5 | 9.34 | -0.036 | 1,404 | 1,404 | 41.8 | 6 | 234 | 55.6 | nucl |
| Ga06G0161 | CYP82A4 | Ga_CYP82A4_258 | Chr06 | 1,361,411 | 1,366,260 | - | 4,850 | 518 | 58.331 | 20 | 9.59 | -0.088 | 1,557 | 1,557 | 42 | 4 | 389.3 | 1,097.70 | E.R. |
| Ga06G0159 | CYP82A3 | Ga_CYP82A3_259 | Chr06 | 1,351,585 | 1,352,001 | - | 417 | 138 | 15.322 | 10.5 | 10.036 | 0.044 | 417 | 417 | 42.9 | 1 | 417 | No intron | nucl |
| Ga06G0157 | CYP82A3 | Ga_CYP82A3_260 | Chr06 | 1,284,686 | 1,286,266 | - | 1,581 | 325 | 36.625 | 13 | 9.586 | -0.123 | 978 | 978 | 42.9 | 6 | 163 | 120.6 | plas |
| Ga06G0155 | CYP82A3 | Ga_CYP82A3_261 | Chr06 | 1,221,863 | 1,246,720 | - | 24,858 | 550 | 62.538 | 9.5 | 8.415 | -0.172 | 1,653 | 1,653 | 41.1 | 3 | 551 | 11,602.50 | nucl |
| Ga06G0156 | CYP82A3 | Ga_CYP82A3_262 | Chr06 | 1,253,425 | 1,255,791 | - | 2,367 | 526 | 59.815 | 6.5 | 7.498 | -0.213 | 1,581 | 1,581 | 42 | 2 | 790.5 | 786 | E.R. |
| Ga05G2102 | CYP82A3 | Ga_CYP82A3_263 | Chr05 | 19,363,923 | 19,365,660 | - | 1,738 | 526 | 58.777 | 8.5 | 8.041 | -0.005 | 1,581 | 1,581 | 47.3 | 2 | 790.5 | 157 | E.R. |
| Ga06G0162 | CYP82A3 | Ga_CYP82A3_264 | Chr06 | 1,368,212 | 1,369,866 | - | 1,655 | 510 | 57.516 | 7 | 8.187 | -0.119 | 1,533 | 1,533 | 43.2 | 3 | 511 | 61 | nucl |
| Ga05G2101 | CYP82A3 | Ga_CYP82A3_265 | Chr05 | 19,273,264 | 19,274,937 | + | 1,674 | 510 | 57.044 | 16.5 | 9.484 | -0.132 | 1,533 | 1,533 | 43.6 | 3 | 511 | 70.5 | E.R. |
| Ga05G2099 | CYP82A3 | Ga_CYP82A3_266 | Chr05 | 19,191,825 | 19,193,482 | - | 1,658 | 522 | 58.569 | 14 | 9.344 | -0.208 | 1,569 | 1,569 | 44.4 | 2 | 784.5 | 89 | plas |
| Ga05G2100 | CYP82A3 | Ga_CYP82A3_267 | Chr05 | 19,199,022 | 19,200,680 | - | 1,659 | 522 | 58.438 | 11.5 | 8.969 | -0.204 | 1,569 | 1,569 | 44.6 | 2 | 784.5 | 90 | E.R. |
| Ga05G3412 | CYP82A3 | Ga_CYP82A3_268 | Chr05 | 50,075,857 | 50,077,520 | - | 1,664 | 522 | 58.367 | 10.5 | 8.528 | -0.221 | 1,569 | 1,569 | 44 | 2 | 784.5 | 95 | cyto |
| Ga14G1622 | CYP82C4 | Ga_CYP82C4_269 | tig00015610 | 190,043 | 191,682 | + | 1,640 | 520 | 58.164 | 13.5 | 8.71 | -0.017 | 1,563 | 1,563 | 45.8 | 2 | 781.5 | 77 | nucl |
| Ga04G0124 | CYP82C4 | Ga_CYP82C4_270 | Chr04 | 1,278,334 | 1,279,998 | + | 1,665 | 514 | 57.746 | 14.5 | 8.634 | -0.04 | 1,545 | 1,545 | 45.1 | 3 | 515 | 60 | nucl |
| Ga04G0125 | CYP82C4 | Ga_CYP82C4_271 | Chr04 | 1,281,577 | 1,283,299 | + | 1,723 | 549 | 62.371 | 14.5 | 8.47 | -0.051 | 1,650 | 1,650 | 43.3 | 2 | 825 | 73 | nucl |
| Ga03G0968 | CYP81E7 | Ga_CYP81E7_272 | Chr03 | 22,911,400 | 22,913,017 | - | 1,618 | 508 | 58.151 | 7 | 8.01 | -0.17 | 1,527 | 1,527 | 45.3 | 2 | 763.5 | 91 | plas |
| Ga05G2206 | CYP81E8 | Ga_CYP81E8_273 | Chr05 | 20,480,322 | 20,482,025 | - | 1,704 | 531 | 60.413 | 13.5 | 8.828 | -0.157 | 1,596 | 1,596 | 45.1 | 3 | 532 | 54 | E.R. |
| Ga05G2796 | CYP81E7 | Ga_CYP81E7_274 | Chr05 | 28,682,792 | 28,684,384 | - | 1,593 | 432 | 49.961 | 17 | 9.299 | -0.27 | 1,299 | 1,299 | 44.5 | 4 | 324.8 | 98 | E.R. |
| Ga06G0239 | CYP81E8 | Ga_CYP81E8_275 | Chr06 | 2,135,437 | 2,137,019 | - | 1,583 | 500 | 57.411 | 2.5 | 6.971 | -0.133 | 1,503 | 1,503 | 44.4 | 2 | 751.5 | 80 | nucl |
| Ga12G0140 | CYP71A26 | Ga_CYP71A26_276 | Chr12 | 1,166,456 | 1,168,387 | - | 1,932 | 108 | 12.601 | -2 | 5.16 | -0.267 | 327 | 327 | 41 | 3 | 109 | 802.5 | nucl |
| Ga05G2211 | CYP81D1 | Ga_CYP81D1_277 | Chr05 | 20,527,031 | 20,533,095 | + | 6,065 | 102 | 11.278 | -4 | 4.663 | -0.434 | 309 | 309 | 48.9 | 2 | 154.5 | 5,756.00 | nucl |
| Ga03G0976 | CYP81E1 | Ga_CYP81E1_278 | Chr03 | 25,153,382 | 25,155,545 | + | 2,164 | 507 | 58.313 | 10 | 8.158 | -0.033 | 1,524 | 1,524 | 43.2 | 2 | 762 | 640 | E.R. |
| Ga05G2212 | CYP81E1 | Ga_CYP81E1_279 | Chr05 | 20,533,300 | 20,535,761 | + | 2,462 | 517 | 58.048 | 5.5 | 7.545 | -0.087 | 1,554 | 1,554 | 43.8 | 2 | 777 | 908 | E.R. |
| Ga11G1869 | CYP81D1 | Ga_CYP81D1_280 | Chr11 | 86,480,655 | 86,482,191 | + | 1,537 | 380 | 43.334 | -7 | 5.209 | -0.096 | 1,143 | 1,143 | 43.7 | 6 | 190.5 | 78.8 | nucl |
| Ga11G1872 | CYP81E8 | Ga_CYP81E8_281 | Chr11 | 86,602,060 | 86,603,644 | + | 1,585 | 500 | 56.763 | 6 | 7.412 | -0.186 | 1,503 | 1,503 | 45.6 | 2 | 751.5 | 82 | plas |
| Ga12G2524 | CYP81D1 | Ga_CYP81D1_282 | Chr12 | 93,935,617 | 93,936,971 | + | 1,355 | 424 | 48.005 | 1 | 6.682 | -0.276 | 1,275 | 1,275 | 45.2 | 2 | 637.5 | 80 | nucl |
| Ga07G0916 | CYP81E8 | Ga_CYP81E8_283 | Chr07 | 11,967,841 | 11,969,756 | + | 1,916 | 497 | 56.175 | 20 | 9.597 | -0.057 | 1,494 | 1,494 | 43.8 | 4 | 373.5 | 140.7 | vacu |
| Ga07G0930 | CYP81E1 | Ga_CYP81E1_284 | Chr07 | 12,198,782 | 12,200,439 | + | 1,658 | 340 | 39.54 | 25 | 9.835 | -0.517 | 1,020 | 1,020 | 43.3 | 5 | 204 | 159.5 | E.R. |
| Ga07G0917 | CYP81E1 | Ga_CYP81E1_285 | Chr07 | 12,017,538 | 12,037,322 | + | 19,785 | 319 | 36.911 | 16 | 9.622 | -0.183 | 960 | 960 | 39.2 | 7 | 137.1 | 3,137.50 | nucl |
| Ga07G0931 | CYP81D1 | Ga_CYP81D1_286 | Chr07 | 12,218,442 | 12,220,069 | + | 1,628 | 425 | 48.128 | 11 | 8.366 | -0.407 | 1,278 | 1,278 | 44.9 | 3 | 426 | 175 | nucl |
| Ga10G0216 | CYP81E8 | Ga_CYP81E8_287 | Chr10 | 3,004,377 | 3,005,201 | - | 825 | 264 | 30.191 | 9.5 | 9.354 | -0.161 | 795 | 795 | 43.3 | 2 | 397.5 | 30 | E.R. |
| Ga10G0217 | CYP81E8 | Ga_CYP81E8_288 | Chr10 | 3,016,101 | 3,018,846 | - | 2,746 | 494 | 56.442 | 23.5 | 9.778 | -0.154 | 1,485 | 1,485 | 43.1 | 2 | 742.5 | 1,261.00 | E.R. |
| Ga02G0249 | CYP81E8 | Ga_CYP81E8_289 | Chr02 | 2,276,933 | 2,280,394 | - | 3,462 | 507 | 57.101 | 13.5 | 8.586 | -0.121 | 1,524 | 1,524 | 43.9 | 2 | 762 | 1,938.00 | E.R. |
| Ga12G2223 | CYP81E8 | Ga_CYP81E8_290 | Chr12 | 73,920,502 | 73,922,829 | + | 2,328 | 504 | 57.269 | 6.5 | 7.671 | -0.106 | 1,515 | 1,515 | 44.2 | 2 | 757.5 | 813 | plas |
| Ga06G0818 | CYP75B1 | Ga_CYP75B1_291 | Chr06 | 15,615,881 | 15,618,101 | - | 2,221 | 517 | 59.63 | 11 | 8.902 | -0.296 | 1,554 | 1,554 | 44.9 | 2 | 777 | 667 | mito |
| Ga10G2503 | CYP75B1 | Ga_CYP75B1_292 | Chr10 | 123,783,198 | 123,785,818 | + | 2,621 | 510 | 58.042 | 15.5 | 9.418 | -0.213 | 1,533 | 1,533 | 45.5 | 2 | 766.5 | 1,088.00 | E.R. |
| Ga10G2504 | CYP71A1 | Ga_CYP71A1_293 | Chr10 | 123,793,184 | 123,795,304 | + | 2,121 | 510 | 58.232 | 8 | 8.508 | -0.248 | 1,533 | 1,533 | 47 | 2 | 766.5 | 588 | nucl |
| Ga11G2622 | CYP71A1 | Ga_CYP71A1_294 | Chr11 | 108,244,020 | 108,246,522 | - | 2,503 | 501 | 57.28 | 10 | 8.731 | -0.192 | 1,506 | 1,506 | 45.2 | 3 | 502 | 498.5 | E.R. |
| Ga06G1761 | CYP75A3 | Ga_CYP75A3_295 | Chr06 | 115,284,186 | 115,286,227 | + | 2,042 | 198 | 23.217 | 5.5 | 8.955 | -0.118 | 597 | 597 | 39.4 | 5 | 119.4 | 361.3 | E.R. |
| Ga13G0640 | CYP84A1 | Ga_CYP84A1_296 | Chr13 | 9,495,656 | 9,498,152 | + | 2,497 | 544 | 61.437 | 3.5 | 6.999 | -0.186 | 1,635 | 1,635 | 48.4 | 2 | 817.5 | 862 | plas |
| Ga11G3628 | CYP84A1 | Ga_CYP84A1_297 | Chr11 | 120,364,159 | 120,366,204 | + | 2,046 | 517 | 58.503 | 3 | 7.052 | -0.139 | 1,554 | 1,554 | 47.7 | 2 | 777 | 492 | nucl |
| Ga11G2107 | CYP84A1 | Ga_CYP84A1_298 | Chr11 | 98,229,216 | 98,231,012 | + | 1,797 | 521 | 59.082 | -1 | 6.369 | -0.176 | 1,566 | 1,566 | 50.1 | 2 | 783 | 231 | E.R. |
| Ga10G2004 | CYP84A1 | Ga_CYP84A1_299 | Chr10 | 108,540,020 | 108,541,546 | - | 1,527 | 508 | 58.043 | 9 | 8.449 | -0.199 | 1,527 | 1,527 | 48.4 | 1 | 1,527.00 | No intron | nucl |
| Ga01G0945 | CYP71D11 | Ga_CYP71D11_300 | Chr01 | 14,259,452 | 14,261,084 | - | 1,633 | 502 | 56.984 | 11 | 8.607 | -0.038 | 1,509 | 1,509 | 41.8 | 2 | 754.5 | 124 | nucl |
| Ga13G1547 | CYP71D55 | Ga_CYP71D55_301 | Chr13 | 99,575,449 | 99,576,993 | + | 1,545 | 485 | 55.025 | 9.5 | 8.21 | -0.103 | 1,458 | 1,458 | 42 | 3 | 486 | 43.5 | nucl |
| Ga01G0950 | CYP71D10 | Ga_CYP71D10_302 | Chr01 | 14,506,251 | 14,547,168 | - | 40,918 | 523 | 59.932 | 11.5 | 8.561 | -0.228 | 1,572 | 1,572 | 42.3 | 4 | 393 | 13,115.30 | plas |
| Ga01G0951 | CYP71D55 | Ga_CYP71D55_303 | Chr01 | 14,618,041 | 14,619,714 | - | 1,674 | 494 | 56.657 | 6 | 7.261 | -0.092 | 1,485 | 1,485 | 39.5 | 3 | 495 | 94.5 | nucl |
| Ga01G0943 | CYP71D55 | Ga_CYP71D55_304 | Chr01 | 14,169,934 | 14,171,676 | - | 1,743 | 475 | 54.11 | -1 | 6.4 | -0.113 | 1,428 | 1,428 | 40.4 | 4 | 357 | 105 | nucl |
| Ga01G0949 | CYP71D10 | Ga_CYP71D10_305 | Chr01 | 14,446,221 | 14,447,959 | - | 1,739 | 317 | 36.152 | 3.5 | 7.129 | -0.161 | 954 | 954 | 41.3 | 3 | 318 | 392.5 | E.R. |
| Ga01G0948 | CYP71D10 | Ga_CYP71D10_306 | Chr01 | 14,424,366 | 14,426,178 | - | 1,813 | 530 | 60.323 | 3 | 6.796 | -0.095 | 1,593 | 1,593 | 39.9 | 2 | 796.5 | 220 | nucl |
| Ga01G0939 | CYP71D8 | Ga_CYP71D8_307 | Chr01 | 14,111,555 | 14,113,162 | - | 1,608 | 511 | 57.484 | 7 | 7.847 | -0.118 | 1,536 | 1,536 | 40.8 | 2 | 768 | 72 | nucl |
| Ga03G0052 | CYP71D10 | Ga_CYP71D10_308 | Chr03 | 334,739 | 336,305 | + | 1,567 | 495 | 55.947 | 8 | 7.91 | -0.079 | 1,488 | 1,488 | 41.7 | 2 | 744 | 79 | E.R. |
| Ga01G0941 | CYP71D10 | Ga_CYP71D10_309 | Chr01 | 14,138,785 | 14,140,406 | - | 1,622 | 461 | 52.829 | 18.5 | 9.313 | -0.187 | 1,386 | 1,386 | 40 | 4 | 346.5 | 78.7 | E.R. |
| Ga13G1687 | CYP71D10 | Ga_CYP71D10_310 | Chr13 | 104,596,799 | 104,598,428 | + | 1,630 | 491 | 56.168 | 15.5 | 9.146 | -0.346 | 1,476 | 1,476 | 46.1 | 3 | 492 | 77 | plas |
| Ga13G1672 | CYP71D10 | Ga_CYP71D10_311 | Chr13 | 103,946,940 | 103,948,560 | + | 1,621 | 512 | 57.955 | 13 | 8.797 | -0.053 | 1,539 | 1,539 | 40.9 | 2 | 769.5 | 82 | nucl |
| Ga05G3691 | CYP71D10 | Ga_CYP71D10_312 | Chr05 | 83,335,814 | 83,339,979 | + | 4,166 | 537 | 60.251 | 16 | 9.306 | -0.012 | 1,614 | 1,614 | 41.7 | 4 | 403.5 | 850.7 | plas |
| Ga05G3693 | CYP71D10 | Ga_CYP71D10_313 | Chr05 | 83,371,081 | 83,375,061 | + | 3,981 | 532 | 59.615 | 22 | 9.906 | -0.076 | 1,599 | 1,599 | 43 | 5 | 319.8 | 595.5 | E.R. |
| Ga05G3694 | CYP71D10 | Ga_CYP71D10_314 | Chr05 | 83,410,917 | 83,412,532 | + | 1,616 | 507 | 57.198 | 6 | 7.625 | -0.064 | 1,524 | 1,524 | 41.8 | 2 | 762 | 92 | nucl |
| Ga05G3695 | CYP71D10 | Ga_CYP71D10_315 | Chr05 | 83,481,213 | 83,488,003 | + | 6,791 | 344 | 39.325 | 3.5 | 7.121 | -0.07 | 1,035 | 1,035 | 40.3 | 4 | 258.8 | 1,918.70 | nucl |
| Ga07G2393 | CYP71D10 | Ga_CYP71D10_316 | Chr07 | 93,041,874 | 93,043,479 | + | 1,606 | 493 | 55.577 | 5.5 | 7.403 | -0.047 | 1,482 | 1,482 | 40.6 | 3 | 494 | 62 | mito |
| Ga13G1949 | CYP71D10 | Ga_CYP71D10_317 | Chr13 | 111,140,530 | 111,142,232 | + | 1,703 | 486 | 54.78 | 5 | 7.359 | -0.078 | 1,461 | 1,461 | 40.6 | 3 | 487 | 121 | nucl |
| Ga13G1948 | CYP71D10 | Ga_CYP71D10_318 | Chr13 | 111,105,275 | 111,106,888 | + | 1,614 | 503 | 56.978 | 8 | 8.033 | -0.13 | 1,512 | 1,512 | 40.5 | 2 | 756 | 102 | nucl |
| Ga13G1950 | CYP71D9 | Ga_CYP71D9_319 | Chr13 | 111,175,767 | 111,177,170 | - | 1,404 | 296 | 33.334 | -6 | 5.257 | -0.144 | 891 | 891 | 38.7 | 5 | 178.2 | 128.3 | nucl |
| Ga01G1864 | CYP71A1 | Ga_CYP71A1_320 | Chr01 | 89,172,291 | 89,173,892 | - | 1,602 | 508 | 57.853 | 4.5 | 7.061 | -0.071 | 1,527 | 1,527 | 45.1 | 2 | 763.5 | 75 | E.R. |
| Ga10G0052 | CYP71A1 | Ga_CYP71A1_321 | Chr10 | 559,316 | 561,139 | + | 1,824 | 502 | 57.113 | 5 | 7.02 | -0.114 | 1,509 | 1,509 | 44.5 | 2 | 754.5 | 315 | vacu |
| Ga07G0656 | CYP71A9 | Ga_CYP71A9_322 | Chr07 | 7,350,841 | 7,353,837 | - | 2,997 | 528 | 59.932 | 16.5 | 9.364 | -0.175 | 1,587 | 1,587 | 41.8 | 2 | 793.5 | 1,410.00 | plas |
| Ga07G0658 | CYP71A9 | Ga_CYP71A9_323 | Chr07 | 7,382,073 | 7,395,736 | - | 13,664 | 465 | 52.562 | 11 | 9.019 | -0.177 | 1,398 | 1,398 | 42.5 | 3 | 466 | 6,133.00 | E.R. |
| Ga14G2111 | CYP83B1 | Ga_CYP83B1_324 | tig00018109 | 729,347 | 730,932 | - | 1,586 | 418 | 48.515 | 8 | 7.909 | -0.209 | 1,257 | 1,257 | 40.6 | 5 | 251.4 | 82.3 | mito |
| Ga14G2109 | CYP83B1 | Ga_CYP83B1_325 | tig00018109 | 675,941 | 677,526 | - | 1,586 | 497 | 57.204 | 7 | 7.551 | -0.119 | 1,494 | 1,494 | 39.4 | 2 | 747 | 92 | nucl |
| Ga14G2113 | CYP83B1 | Ga_CYP83B1_326 | tig00018109 | 766,194 | 767,778 | - | 1,585 | 497 | 57.177 | 7.5 | 7.81 | -0.111 | 1,494 | 1,494 | 40 | 2 | 747 | 91 | cyto |
| Ga10G0710 | CYP83B1 | Ga_CYP83B1_327 | Chr10 | 13,061,211 | 13,062,268 | - | 1,058 | 325 | 37.161 | -5 | 5.27 | -0.055 | 978 | 978 | 39.8 | 2 | 489 | 80 | nucl |
| Ga10G0711 | CYP83B1 | Ga_CYP83B1_328 | Chr10 | 13,082,958 | 13,084,541 | - | 1,584 | 487 | 55.299 | 11.5 | 8.409 | 0.078 | 1,464 | 1,464 | 41.2 | 3 | 488 | 60 | nucl |
| Ga10G0709 | CYP83B1 | Ga_CYP83B1_329 | Chr10 | 13,034,818 | 13,036,570 | - | 1,753 | 530 | 61.314 | 6.5 | 7.362 | -0.11 | 1,593 | 1,593 | 38.9 | 3 | 531 | 80 | E.R. |
| Ga10G0714 | CYP83B1 | Ga_CYP83B1_330 | Chr10 | 13,145,852 | 13,165,333 | + | 19,482 | 1,002 | 114.353 | 15 | 8.043 | -0.003 | 3,009 | 3,009 | 40 | 4 | 752.3 | 5,491.00 | plas |
| Ga10G0712 | CYP71B34 | Ga_CYP71B34_331 | Chr10 | 13,104,085 | 13,105,704 | + | 1,620 | 503 | 57.604 | 10.5 | 8.521 | -0.024 | 1,512 | 1,512 | 40.5 | 2 | 756 | 108 | E.R. |
| Ga10G0713 | CYP83B1 | Ga_CYP83B1_332 | Chr10 | 13,131,563 | 13,132,550 | + | 988 | 180 | 20.889 | 1.5 | 6.881 | -0.222 | 543 | 543 | 40 | 3 | 181 | 222.5 | nucl |
| Ga11G1653 | CYP71E7 | Ga_CYP71E7_333 | Chr11 | 68,655,154 | 68,656,678 | + | 1,525 | 423 | 47.364 | 10 | 8.53 | -0.146 | 1,272 | 1,272 | 44.7 | 3 | 424 | 126.5 | nucl |
| Ga11G1654 | CYP71E7 | Ga_CYP71E7_334 | Chr11 | 68,808,698 | 68,810,342 | - | 1,645 | 504 | 57.488 | 3.5 | 7.253 | -0.164 | 1,515 | 1,515 | 44.1 | 3 | 505 | 65 | nucl |
| Ga03G1788 | CYP71B35 | Ga_CYP71B35_335 | Chr03 | 117,243,488 | 117,245,225 | - | 1,738 | 450 | 50.568 | -0.5 | 6.432 | -0.121 | 1,353 | 1,353 | 42.2 | 5 | 270.6 | 96.3 | nucl |
| Ga12G1402 | CYP71B36 | Ga_CYP71B36_336 | Chr12 | 19,493,460 | 19,498,483 | - | 5,024 | 514 | 58.461 | 12 | 8.28 | -0.047 | 1,545 | 1,545 | 46 | 2 | 772.5 | 3,479.00 | nucl |
| Ga12G0917 | CYP71A22 | Ga_CYP71A22_337 | Chr12 | 9,403,177 | 9,406,409 | + | 3,233 | 507 | 57.043 | 14 | 8.884 | -0.123 | 1,524 | 1,524 | 43.2 | 5 | 304.8 | 427.3 | E.R. |
| Ga12G0968 | CYP71A26 | Ga_CYP71A26_338 | Chr12 | 9,985,792 | 9,987,557 | - | 1,766 | 542 | 61.663 | 9.5 | 7.728 | -0.133 | 1,629 | 1,629 | 44.1 | 2 | 814.5 | 137 | plas |
| Ga01G1868 | CYP71A1 | Ga_CYP71A1_339 | Chr01 | 89,218,653 | 89,229,602 | + | 10,950 | 900 | 101.909 | -3.5 | 6.335 | -0.21 | 2,703 | 2,703 | 42.7 | 7 | 386.1 | 1,374.50 | E.R. |
| Ga10G1490 | CYP71A1 | Ga_CYP71A1_340 | Chr10 | 81,422,804 | 81,424,431 | + | 1,628 | 502 | 57.253 | 12.5 | 8.534 | -0.08 | 1,509 | 1,509 | 42.9 | 2 | 754.5 | 119 | plas |
| Ga07G0660 | CYP71A1 | Ga_CYP71A1_341 | Chr07 | 7,428,736 | 7,430,689 | + | 1,954 | 416 | 47.923 | -5 | 5.384 | -0.216 | 1,251 | 1,251 | 38.8 | 3 | 417 | 351.5 | vacu |
| Ga12G0969 | CYP71A1 | Ga_CYP71A1_342 | Chr12 | 10,016,889 | 10,018,604 | + | 1,716 | 539 | 62.048 | 10 | 8.78 | -0.192 | 1,620 | 1,620 | 42.2 | 2 | 810 | 96 | E.R. |
| Ga12G0970 | CYP71A1 | Ga_CYP71A1_343 | Chr12 | 10,025,294 | 10,027,002 | + | 1,709 | 484 | 55.923 | 12 | 9.457 | -0.012 | 1,455 | 1,455 | 41.4 | 5 | 291 | 63.5 | E.R. |
| Ga10G0634 | CYP71A1 | Ga_CYP71A1_344 | Chr10 | 11,298,269 | 11,299,902 | - | 1,634 | 500 | 57.138 | 4 | 7.12 | -0.055 | 1,503 | 1,503 | 41.3 | 3 | 501 | 65.5 | E.R. |
| Ga10G0631 | CYP71A1 | Ga_CYP71A1_345 | Chr10 | 11,059,763 | 11,068,654 | + | 8,892 | 439 | 50.138 | 2 | 6.796 | -0.18 | 1,320 | 1,320 | 41.7 | 4 | 330 | 2,524.00 | E.R. |
| Ga13G2469 | CYP71A1 | Ga_CYP71A1_346 | Chr13 | 119,996,162 | 119,997,782 | + | 1,621 | 510 | 58.297 | 9.5 | 8.036 | -0.009 | 1,533 | 1,533 | 41 | 2 | 766.5 | 88 | E.R. |
| Ga10G0632 | CYP71A1 | Ga_CYP71A1_347 | Chr10 | 11,154,512 | 11,156,125 | - | 1,614 | 507 | 57.721 | 2.5 | 6.897 | -0.008 | 1,524 | 1,524 | 41.4 | 2 | 762 | 90 | E.R. |
| Ga10G0633 | CYP71A1 | Ga_CYP71A1_348 | Chr10 | 11,159,241 | 11,160,854 | - | 1,614 | 507 | 57.94 | 1.5 | 6.739 | -0.058 | 1,524 | 1,524 | 41.4 | 2 | 762 | 90 | E.R. |
| Ga10G0630 | CYP71A9 | Ga_CYP71A9_349 | Chr10 | 11,050,746 | 11,059,620 | - | 8,875 | 405 | 46.347 | 10 | 9.126 | -0.17 | 1,218 | 1,218 | 41 | 6 | 203 | 1,531.40 | E.R. |
| Ga09G2311 | CYP736A12 | Ga_CYP736A12_350 | Chr09 | 80,805,559 | 80,811,638 | - | 6,080 | 499 | 56.747 | 7.5 | 7.594 | -0.126 | 1,500 | 1,500 | 44.7 | 2 | 750 | 4,580.00 | nucl |
| Ga09G0178 | CYP736A12 | Ga_CYP736A12_351 | Chr09 | 3,808,821 | 3,812,031 | - | 3,211 | 480 | 54.754 | 11 | 8.691 | -0.164 | 1,443 | 1,443 | 42.4 | 3 | 481 | 884 | nucl |
| Ga09G0179 | CYP736A12 | Ga_CYP736A12_352 | Chr09 | 3,822,697 | 3,825,988 | - | 3,292 | 468 | 53.341 | 7.5 | 7.501 | -0.092 | 1,407 | 1,407 | 41.9 | 4 | 351.8 | 628.3 | nucl |
| Ga09G0635 | CYP736A12 | Ga_CYP736A12_353 | Chr09 | 43,578,320 | 43,579,906 | + | 1,587 | 493 | 55.849 | 7.5 | 7.965 | -0.09 | 1,482 | 1,482 | 39.5 | 2 | 741 | 105 | nucl |
| Ga05G3150 | CYP736A12 | Ga_CYP736A12_354 | Chr05 | 37,512,170 | 37,513,768 | - | 1,599 | 497 | 55.847 | 6.5 | 7.72 | 0.006 | 1,494 | 1,494 | 41.3 | 2 | 747 | 105 | nucl |
| Ga05G3151 | CYP736A12 | Ga_CYP736A12_355 | Chr05 | 37,605,648 | 37,607,259 | - | 1,612 | 485 | 54.97 | 9.5 | 8.536 | -0.002 | 1,458 | 1,458 | 39.5 | 3 | 486 | 77 | nucl |
| Ga09G0636 | CYP736A12 | Ga_CYP736A12_356 | Chr09 | 44,285,455 | 44,287,053 | + | 1,599 | 497 | 55.752 | 9.5 | 8.357 | 0.014 | 1,494 | 1,494 | 42.4 | 2 | 747 | 105 | nucl |
| Ga09G0797 | CYP736A12 | Ga_CYP736A12_357 | Chr09 | 57,086,785 | 57,087,361 | - | 577 | 158 | 18.101 | 10 | 10.245 | 0.015 | 477 | 477 | 35.8 | 2 | 238.5 | 100 | E.R. |
| Ga09G0177 | CYP736A12 | Ga_CYP736A12_358 | Chr09 | 3,778,134 | 3,779,596 | - | 1,463 | 374 | 42.245 | 3 | 6.892 | -0.111 | 1,125 | 1,125 | 42.4 | 3 | 375 | 169 | nucl |
| Ga01G0684 | CYP736A12 | Ga_CYP736A12_359 | Chr01 | 9,013,804 | 9,015,588 | - | 1,785 | 495 | 55.702 | 11.5 | 8.95 | -0.028 | 1,488 | 1,488 | 46.3 | 3 | 496 | 148.5 | plas |
| Ga01G0685 | CYP736A12 | Ga_CYP736A12_360 | Chr01 | 9,023,201 | 9,025,023 | - | 1,823 | 519 | 58.71 | 12.5 | 8.868 | -0.083 | 1,560 | 1,560 | 45.6 | 2 | 780 | 263 | plas |
| Ga13G0262 | CYP736A12 | Ga_CYP736A12_361 | Chr13 | 2,711,733 | 2,714,380 | - | 2,648 | 489 | 55.841 | 12.5 | 8.361 | -0.103 | 1,470 | 1,470 | 44.1 | 3 | 490 | 589 | plas |
| Ga13G0260 | CYP736A12 | Ga_CYP736A12_362 | Chr13 | 2,675,484 | 2,680,863 | - | 5,380 | 511 | 58.195 | 12 | 8.439 | -0.125 | 1,536 | 1,536 | 44.7 | 3 | 512 | 1,922.00 | plas |
| Ga13G0251 | CYP736A12 | Ga_CYP736A12_363 | Chr13 | 2,532,072 | 2,536,382 | - | 4,311 | 477 | 54.217 | 15.5 | 9.174 | -0.056 | 1,434 | 1,434 | 43.6 | 4 | 358.5 | 959 | plas |
| Ga13G0259 | CYP736A12 | Ga_CYP736A12_364 | Chr13 | 2,649,655 | 2,653,469 | - | 3,815 | 284 | 32.201 | 4.5 | 7.689 | 0.002 | 855 | 855 | 44.1 | 4 | 213.8 | 986.7 | E.R. |
| Ga02G1448 | CYP93A1 | Ga_CYP93A1_365 | Chr02 | 94,342,032 | 94,344,115 | + | 2,084 | 515 | 58.45 | 8.5 | 8.038 | -0.194 | 1,548 | 1,548 | 43.3 | 2 | 774 | 536 | mito |
| Ga09G1433 | CYP93A1 | Ga_CYP93A1_366 | Chr09 | 70,657,864 | 70,659,516 | + | 1,653 | 508 | 57.993 | 14 | 8.916 | -0.167 | 1,527 | 1,527 | 44.4 | 2 | 763.5 | 126 | nucl |
| Ga09G1434 | CYP93A1 | Ga_CYP93A1_367 | Chr09 | 70,659,988 | 70,661,599 | + | 1,612 | 453 | 51.138 | 14 | 9.281 | -0.229 | 1,362 | 1,362 | 43.6 | 3 | 454 | 125 | nucl |
| Ga09G1435 | CYP93A1 | Ga_CYP93A1_368 | Chr09 | 70,702,168 | 70,703,827 | + | 1,660 | 512 | 58.352 | 18.5 | 9.411 | -0.268 | 1,539 | 1,539 | 42.4 | 2 | 769.5 | 121 | E.R. |
| Ga09G2270 | CYP93B1 | Ga_CYP93B1_369 | Chr09 | 80,425,236 | 80,427,053 | + | 1,818 | 486 | 55.117 | 13.5 | 9.112 | -0.063 | 1,461 | 1,461 | 45.9 | 5 | 292.2 | 89.3 | E.R. |
| Ga13G1514 | CYP93B1 | Ga_CYP93B1_370 | Chr13 | 96,662,194 | 96,664,502 | + | 2,309 | 532 | 60.478 | 7.5 | 7.417 | -0.078 | 1,599 | 1,599 | 44.2 | 2 | 799.5 | 710 | E.R. |
| Ga13G1541 | CYP93B1 | Ga_CYP93B1_371 | Chr13 | 99,195,444 | 99,209,404 | - | 13,961 | 474 | 54.449 | 13.5 | 8.885 | -0.103 | 1,425 | 1,425 | 44.1 | 5 | 285 | 3,134.00 | E.R. |
| Ga02G1443 | CYP93A3 | Ga_CYP93A3_372 | Chr02 | 94,259,894 | 94,262,042 | - | 2,149 | 494 | 56.232 | 6 | 7.355 | -0.104 | 1,485 | 1,485 | 41.7 | 3 | 495 | 332 | E.R. |
| Ga02G1444 | CYP93A3 | Ga_CYP93A3_373 | Chr02 | 94,292,260 | 94,295,877 | - | 3,618 | 502 | 56.963 | 5 | 7.172 | -0.087 | 1,509 | 1,509 | 41.3 | 3 | 503 | 1,054.50 | E.R. |
| Ga02G1445 | CYP93A1 | Ga_CYP93A1_374 | Chr02 | 94,318,126 | 94,319,758 | - | 1,633 | 518 | 58.956 | 12.5 | 8.587 | -0.28 | 1,557 | 1,557 | 42.1 | 2 | 778.5 | 76 | nucl |
| Ga09G1437 | CYP93A1 | Ga_CYP93A1_375 | Chr09 | 70,712,385 | 70,715,898 | + | 3,514 | 535 | 60.826 | 7.5 | 7.926 | -0.154 | 1,608 | 1,608 | 42.2 | 3 | 536 | 953 | E.R. |
| Ga09G1438 | CYP93A2 | Ga_CYP93A2_376 | Chr09 | 70,718,841 | 70,719,254 | + | 414 | 97 | 11.022 | 0.5 | 6.775 | -0.812 | 294 | 294 | 48 | 2 | 147 | 120 | nucl |
| Ga12G1008 | CYP75B2 | Ga_CYP75B2_377 | Chr12 | 10,694,310 | 10,698,266 | - | 3,957 | 510 | 56.745 | 14.5 | 9.495 | -0.087 | 1,533 | 1,533 | 51.8 | 3 | 511 | 1,212.00 | plas |
| Ga07G1336 | CYP75A3 | Ga_CYP75A3_378 | Chr07 | 23,772,259 | 23,773,871 | + | 1,613 | 510 | 57.332 | 13.5 | 9.46 | -0.105 | 1,533 | 1,533 | 48.8 | 2 | 766.5 | 80 | plas |
| Ga07G1338 | CYP75A3 | Ga_CYP75A3_379 | Chr07 | 23,801,076 | 23,802,688 | + | 1,613 | 510 | 57.28 | 14.5 | 9.472 | -0.13 | 1,533 | 1,533 | 49.1 | 2 | 766.5 | 80 | plas |
