# Supplementary material for: Knockdown of Cytochrome P450 Genes Gh_D07G1197 and Gh_A13G2057 on Chromosomes D07 and A13 Reveals Their Putative Role in Enhancing Drought and Salt Stress Tolerance in Gossypium hirsutum
Source: Genes (Basel). 2019 Mar 18;10(3):226. doi: 10.3390/genes10030226 (PMC6471685; doi:10.3390/genes10030226)
Supplement: Supplementary file 1 [file genes-10-00226-s001.zip › Supplementary files/Table S4.docx]

Supplementary Table S4: Details of the diploid cotton, *G. raimondii* cytochrome P450 proteins, their physiochemical properties and gene annotation as analysed through phylogenetic tree

| **Gene ID** | **Gene Name** | **Gene annotation** | **Chro** | **Start** | **End** | **Strand** | **Length (bp)** | **Protein Length (aa)** | **Molecular Weight (kDa)** | **Charge** | **pI** | **GRAVY** | **Transcript Length (bp)** | **CDS Length (bp)** | **CDS GC Content (%)** | **Exon Number** | **Mean Exon Length (bp)** | **Mean Intron Length (bp)** | **wolfsport subcellular localization** |
| --- | --- | --- | --- | --- | --- | --- | --- | --- | --- | --- | --- | --- | --- | --- | --- | --- | --- | --- | --- |
| Gorai.006G224300 | CYP736A12 | *Gr_ CYP736A12_1* | Chr06 | 47,617,079 | 47,619,057 | - | 1,979 | 499 | 56.976 | 7 | 7.578 | -0.111 | 1,906 | 1,500 | 44.7 | 2 | 953 | 73 | nucl |
| Gorai.006G224400 | CYP736A12 | *Gr_ CYP736A12_2* | Chr06 | 47,621,606 | 47,623,612 | - | 2,007 | 499 | 56.921 | 8 | 7.829 | -0.153 | 1,678 | 1,500 | 45.4 | 2 | 839 | 73 | nucl |
| Gorai.006G062100 | CYP736A12 | *Gr_ CYP736A12_3* | Chr06 | 22,823,572 | 22,825,321 | - | 1,750 | 493 | 55.647 | 12 | 8.903 | -0.068 | 1,645 | 1,482 | 41.1 | 2 | 822.5 | 105 | nucl |
| Gorai.006G081800 | CYP736A12 | *Gr_ CYP736A12_4* | Chr06 | 31,044,712 | 31,046,253 | - | 1,542 | 438 | 49.753 | 14.5 | 9.142 | 0.153 | 1,317 | 1,317 | 38.4 | 3 | 439 | 112.5 | nucl |
| Gorai.004G095700 | CYP736A12 | *Gr_ CYP736A12_5* | Chr04 | 14,893,403 | 14,895,098 | + | 1,696 | 493 | 55.912 | 13.5 | 9.191 | -0.059 | 1,591 | 1,482 | 40.2 | 2 | 795.5 | 105 | nucl |
| Gorai.006G062000 | CYP736A12 | *Gr_ CYP736A12_6* | Chr06 | 22,676,746 | 22,678,505 | - | 1,760 | 493 | 56.093 | 19 | 9.731 | -0.082 | 1,655 | 1,482 | 40.3 | 2 | 827.5 | 105 | nucl |
| Gorai.006G061800 | CYP736A12 | *Gr_ CYP736A12_7* | Chr06 | 22,596,254 | 22,598,138 | - | 1,885 | 499 | 55.864 | 9.5 | 8.357 | -0.005 | 1,780 | 1,500 | 41.7 | 2 | 890 | 105 | nucl |
| Gorai.009G310200 | CYP736A12 | *Gr_ CYP736A12_8* | Chr09 | 28,747,174 | 28,749,002 | - | 1,829 | 502 | 56.693 | 13.5 | 9.164 | -0.015 | 1,723 | 1,509 | 39.7 | 2 | 861.5 | 106 | nucl |
| Gorai.006G062200 | CYP736A12 | *Gr_ CYP736A12_9* | Chr06 | 22,968,861 | 22,970,716 | - | 1,856 | 513 | 57.996 | 13.5 | 8.56 | -0.058 | 1,761 | 1,542 | 42.7 | 2 | 880.5 | 95 | E.R. |
| Gorai.013G025400 | CYP736A12 | *Gr_ CYP736A12_10* | Chr13 | 1,897,879 | 1,902,498 | - | 4,620 | 507 | 57.695 | 12 | 8.234 | -0.115 | 1,904 | 1,524 | 44.6 | 2 | 952 | 2,716.00 | plas |
| Gorai.013G025300 | CYP736A12 | *Gr_ CYP736A12_11* | Chr13 | 1,886,645 | 1,890,813 | - | 4,169 | 343 | 38.647 | 30 | 10.4 | -0.194 | 1,344 | 1,032 | 46.6 | 3 | 448 | 1,412.50 | plas |
| Gorai.002G072100 | CYP736A12 | *Gr_ CYP736A12_12* | Chr02 | 8,412,489 | 8,413,373 | - | 885 | 78 | 8.939 | -6.5 | 3.976 | -0.246 | 623 | 237 | 42.2 | 2 | 311.5 | 262 | nucl |
| Gorai.002G071900 | CYP736A12 | *Gr_ CYP736A12_13* | Chr02 | 8,364,050 | 8,366,388 | - | 2,339 | 519 | 58.832 | 11 | 8.525 | -0.052 | 2,075 | 1,560 | 45.6 | 2 | 1,037.50 | 264 | plas |
| Gorai.002G072000 | CYP736A12 | *Gr_ CYP736A12_14* | Chr02 | 8,405,998 | 8,408,149 | - | 2,152 | 519 | 58.882 | 8 | 8.129 | -0.04 | 1,884 | 1,560 | 45.3 | 2 | 942 | 268 | plas |
| Gorai.013G032800 | CYP84A1 | *Gr_CYP84A1_15* | Chr13 | 2,485,806 | 2,487,961 | - | 2,156 | 544 | 61.024 | 1.5 | 6.708 | -0.121 | 1,822 | 1,635 | 49.4 | 2 | 911 | 334 | nucl |
| Gorai.007G197700 | CYP84A1 | *Gr_CYP84A1_16* | Chr07 | 19,902,103 | 19,904,493 | - | 2,391 | 515 | 58.373 | -2 | 6.211 | -0.153 | 2,161 | 1,548 | 50.2 | 2 | 1,080.50 | 230 | E.R. |
| Gorai.007G049600 | CYP84A1 | *Gr_CYP84A1_17* | Chr07 | 3,461,832 | 3,464,362 | - | 2,531 | 517 | 58.393 | 3 | 7.052 | -0.136 | 2,031 | 1,554 | 48.3 | 2 | 1,015.50 | 500 | nucl |
| Gorai.011G099800 | CYP84A1 | *Gr_CYP84A1_18* | Chr11 | 11,107,198 | 11,108,724 | - | 1,527 | 508 | 57.918 | 9 | 8.449 | -0.2 | 1,527 | 1,527 | 48.4 | 1 | 1,527.00 | No intron | nucl |
| Gorai.002G097400 | CYP71D8 | *Gr_CYP71D8_19* | Chr02 | 12,300,680 | 12,302,857 | - | 2,178 | 502 | 56.831 | 9 | 8.438 | -0.032 | 2,054 | 1,509 | 42.1 | 2 | 1,027.00 | 124 | nucl |
| Gorai.002G098100 | CYP71D55 | *Gr_CYP71D55_20* | Chr02 | 12,456,157 | 12,458,595 | - | 2,439 | 463 | 52.738 | 1.5 | 6.681 | -0.216 | 1,571 | 1,392 | 42.6 | 4 | 392.8 | 289.3 | nucl |
| Gorai.002G097800 | CYP71D55 | *Gr_CYP71D55_21* | Chr02 | 12,382,000 | 12,383,445 | - | 1,446 | 385 | 43.95 | 0.5 | 6.575 | -0.245 | 1,158 | 1,158 | 41.4 | 5 | 231.6 | 72 | E.R. |
| Gorai.002G097500 | CYP71D55 | *Gr_CYP71D55_22* | Chr02 | 12,324,694 | 12,326,803 | - | 2,110 | 509 | 57.75 | 4 | 6.956 | -0.076 | 1,889 | 1,530 | 41.3 | 2 | 944.5 | 221 | nucl |
| Gorai.002G097700 | CYP71D9 | *Gr_CYP71D9_23* | Chr02 | 12,356,800 | 12,359,960 | - | 3,161 | 250 | 28.597 | 11 | 9.247 | 0.179 | 753 | 753 | 39 | 3 | 251 | 1,204.00 | chlo |
| Gorai.002G097900 | CYP71D10 | *Gr_CYP71D10_24* | Chr02 | 12,408,087 | 12,410,230 | + | 2,144 | 360 | 41.017 | -5.5 | 5.485 | -0.207 | 1,921 | 1,083 | 40.3 | 2 | 960.5 | 223 | nucl |
| Gorai.002G098000 | CYP71AV8 | *Gr_CYP71AV8_25* | Chr02 | 12,424,016 | 12,424,834 | + | 819 | 191 | 21.977 | 13.5 | 10.43 | 0.188 | 819 | 576 | 39.8 | 1 | 819 | No intron | chlo |
| Gorai.002G097300 | CYP71D55 | *Gr_CYP71D55_26* | Chr02 | 12,265,286 | 12,267,258 | - | 1,973 | 508 | 58.015 | 3.5 | 6.826 | -0.142 | 1,753 | 1,527 | 41.3 | 2 | 876.5 | 220 | E.R. |
| Gorai.002G098300 | CYP71D10 | *Gr_CYP71D10_27* | Chr02 | 12,520,605 | 12,521,302 | - | 698 | 153 | 17.715 | 1 | 7.51 | -0.406 | 698 | 462 | 40.5 | 1 | 698 | No intron | nucl |
| Gorai.002G097100 | CYP71D8 | *Gr_CYP71D8_28* | Chr02 | 12,243,374 | 12,245,335 | - | 1,962 | 511 | 57.621 | 10 | 8.452 | -0.124 | 1,890 | 1,536 | 40.8 | 2 | 945 | 72 | nucl |
| Gorai.005G005900 | CYP71D10 | *Gr_CYP71D10_29* | Chr05 | 375,647 | 377,672 | + | 2,026 | 498 | 56.37 | 7 | 7.656 | -0.067 | 1,947 | 1,497 | 41.4 | 2 | 973.5 | 79 | E.R. |
| Gorai.013G180800 | CYP71D10 | *Gr_CYP71D10_30* | Chr13 | 47,527,869 | 47,529,489 | - | 1,621 | 507 | 57.166 | 3.5 | 7 | -0.13 | 1,524 | 1,524 | 39.7 | 2 | 762 | 97 | nucl |
| Gorai.013G180900 | CYP71D10 | *Gr_CYP71D10_31* | Chr13 | 47,552,944 | 47,554,873 | + | 1,930 | 516 | 58.232 | 10.5 | 8.828 | -0.073 | 1,788 | 1,551 | 40.6 | 2 | 894 | 142 | nucl |
| Gorai.001G239400 | CYP71D10 | *Gr_CYP71D10_32* | Chr01 | 47,858,102 | 47,859,291 | + | 1,190 | 369 | 41.579 | -1.5 | 6.194 | -0.261 | 1,110 | 1,110 | 40.6 | 2 | 555 | 80 | nucl |
| Gorai.001G239600 | CYP71D10 | *Gr_CYP71D10_33* | Chr01 | 47,912,580 | 47,914,397 | + | 1,818 | 506 | 57.147 | 8 | 8.129 | -0.081 | 1,738 | 1,521 | 40.8 | 2 | 869 | 80 | mito |
| Gorai.013G154300 | CYP71D10 | *Gr_CYP71D10_34* | Chr13 | 42,516,677 | 42,518,699 | + | 2,023 | 508 | 57.908 | 13 | 8.962 | -0.287 | 1,916 | 1,527 | 46.5 | 2 | 958 | 107 | plas |
| Gorai.013G153200 | CYP71D10 | *Gr_CYP71D10_35* | Chr13 | 42,034,128 | 42,036,063 | + | 1,936 | 529 | 59.798 | 12 | 8.709 | 0.047 | 1,854 | 1,590 | 40.6 | 2 | 927 | 82 | nucl |
| Gorai.013G181700 | CYP71D10 | *Gr_CYP71D10_36* | Chr13 | 47,754,038 | 47,755,995 | - | 1,958 | 500 | 56.433 | 10.5 | 8.477 | -0.042 | 1,843 | 1,503 | 40.6 | 2 | 921.5 | 115 | nucl |
| Gorai.009G358400 | CYP71D10 | *Gr_CYP71D10_37* | Chr09 | 47,056,331 | 47,058,667 | + | 2,337 | 508 | 56.784 | 12.5 | 8.944 | -0.024 | 2,245 | 1,527 | 41.8 | 2 | 1,122.50 | 92 | E.R. |
| Gorai.009G359000 | CYP71D10 | *Gr_CYP71D10_38* | Chr09 | 47,217,558 | 47,219,641 | + | 2,084 | 508 | 56.761 | 10.5 | 8.567 | -0.021 | 1,992 | 1,527 | 42.2 | 2 | 996 | 92 | E.R. |
| Gorai.009G358600 | CYP71D10 | *Gr_CYP71D10_39* | Chr09 | 47,074,337 | 47,076,314 | + | 1,978 | 485 | 54.398 | 8.5 | 8.156 | -0.02 | 1,455 | 1,455 | 42.3 | 3 | 485 | 68.5 | E.R. |
| Gorai.009G358800 | CYP71D9 | *Gr_CYP71D9_40* | Chr09 | 47,108,263 | 47,110,154 | + | 1,892 | 283 | 31.847 | 1 | 6.685 | -0.024 | 1,128 | 852 | 43.5 | 4 | 282 | 254.7 | nucl |
| Gorai.009G359100 | CYP71D10 | *Gr_CYP71D10_41* | Chr09 | 47,235,935 | 47,237,846 | + | 1,912 | 507 | 57.092 | 4.5 | 7.173 | -0.077 | 1,820 | 1,524 | 42.4 | 2 | 910 | 92 | nucl |
| Gorai.008G202500 | CYP71A26 | *Gr_CYP71A26_42* | Chr08 | 48,832,925 | 48,835,032 | + | 2,108 | 542 | 61.658 | 10 | 8.151 | -0.142 | 1,971 | 1,629 | 44.2 | 2 | 985.5 | 137 | E.R. |
| Gorai.008G207500 | CYP71A25 | *Gr_CYP71A25_43* | Chr08 | 49,253,280 | 49,255,134 | - | 1,855 | 421 | 48.657 | 21.5 | 10.03 | -0.093 | 1,266 | 1,266 | 42.1 | 6 | 211 | 117.8 | nucl |
| Gorai.003G107200 | CYP71A1 | *Gr_CYP71A1_44* | Chr03 | 32,923,807 | 32,926,080 | - | 2,274 | 516 | 58.984 | -5.5 | 5.782 | -0.085 | 1,992 | 1,551 | 41.2 | 3 | 664 | 115 | E.R. |
| Gorai.011G157400 | CYP71A1 | *Gr_CYP71A1_45* | Chr11 | 27,589,691 | 27,591,582 | - | 1,892 | 508 | 58.091 | 10 | 8.043 | -0.031 | 1,790 | 1,527 | 43.1 | 2 | 895 | 102 | E.R. |
| Gorai.001G063900 | CYP71A1 | *Gr_CYP71A1_46* | Chr01 | 6,366,067 | 6,368,997 | + | 2,931 | 525 | 60.449 | 5 | 7.521 | -0.091 | 1,949 | 1,578 | 39.1 | 2 | 974.5 | 982 | plas |
| Gorai.008G202400 | CYP71A1 | *Gr_CYP71A1_47* | Chr08 | 48,810,293 | 48,812,362 | - | 2,070 | 539 | 62.216 | 10.5 | 8.931 | -0.173 | 1,970 | 1,620 | 42.2 | 2 | 985 | 100 | E.R. |
| Gorai.011G232300 | CYP71A1 | *Gr_CYP71A1_48* | Chr11 | 54,901,164 | 54,903,091 | + | 1,928 | 409 | 46.646 | 9 | 8.385 | 0.023 | 1,835 | 1,230 | 41.4 | 2 | 917.5 | 93 | E.R. |
| Gorai.011G232500 | CYP71A1 | *Gr_CYP71A1_49* | Chr11 | 54,944,808 | 54,946,763 | + | 1,956 | 509 | 58.474 | 3 | 6.94 | -0.128 | 1,859 | 1,530 | 41.7 | 2 | 929.5 | 97 | E.R. |
| Gorai.011G232600 | CYP71A1 | *Gr_CYP71A1_50* | Chr11 | 55,006,160 | 55,007,943 | + | 1,784 | 507 | 57.874 | 4.5 | 7.327 | -0.062 | 1,700 | 1,524 | 41.4 | 2 | 850 | 84 | E.R. |
| Gorai.011G232700 | CYP71A1 | *Gr_CYP71A1_51* | Chr11 | 55,016,568 | 55,018,380 | + | 1,813 | 507 | 57.667 | 4 | 7.127 | -0.007 | 1,723 | 1,524 | 41.5 | 2 | 861.5 | 90 | E.R. |
| Gorai.011G232900 | CYP71A1 | *Gr_CYP71A1_52* | Chr11 | 55,062,555 | 55,064,765 | + | 2,211 | 415 | 47.348 | 10 | 8.713 | -0.211 | 1,248 | 1,248 | 41.6 | 6 | 208 | 192.6 | E.R. |
| Gorai.011G232800 | CYP71A1 | *Gr_CYP71A1_53* | Chr11 | 55,051,480 | 55,052,885 | - | 1,406 | 436 | 49.987 | -2 | 6.199 | -0.152 | 1,311 | 1,311 | 41.6 | 2 | 655.5 | 95 | E.R. |
| Gorai.013G233300 | CYP71A1 | *Gr_CYP71A1_54* | Chr13 | 55,195,098 | 55,205,127 | + | 10,030 | 512 | 58.701 | 9 | 8.271 | -0.008 | 1,824 | 1,539 | 41.1 | 2 | 912 | 89 | E.R. |
| Gorai.013G233400 | CYP71A1 | *Gr_CYP71A1_55* | Chr13 | 55,203,483 | 55,204,306 | + | 824 | 228 | 25.804 | 8.5 | 9.228 | 0.132 | 824 | 687 | 40.5 | 1 | 824 | No intron | extr |
| Gorai.007G041400 | CYP71A1 | *Gr_CYP71A1_56* | Chr07 | 2,895,495 | 2,896,775 | - | 1,281 | 353 | 40.342 | 1.5 | 6.989 | -0.131 | 1,062 | 1,062 | 42.4 | 4 | 265.5 | 73 | nucl |
| Gorai.001G063600 | CYP71A9 | *Gr_CYP71A9_57* | Chr01 | 6,336,791 | 6,340,120 | - | 3,330 | 527 | 59.728 | 12 | 8.698 | -0.152 | 1,845 | 1,584 | 42.6 | 2 | 922.5 | 1,485.00 | plas |
| Gorai.011G262400 | CYP83B1 | *Gr_CYP83B1_58* | Chr11 | 59,337,596 | 59,339,339 | + | 1,744 | 497 | 57.311 | 7 | 7.762 | -0.058 | 1,653 | 1,494 | 39.3 | 2 | 826.5 | 91 | cyto |
| Gorai.003G024500 | CYP83B1 | *Gr_CYP83B1_59* | Chr03 | 2,121,401 | 2,122,991 | - | 1,591 | 481 | 55.355 | 7.5 | 7.808 | -0.061 | 1,446 | 1,446 | 39.4 | 3 | 482 | 72.5 | nucl |
| Gorai.003G024400 | CYP83B1 | *Gr_CYP83B1_60* | Chr03 | 2,111,397 | 2,112,949 | - | 1,553 | 412 | 47.929 | 6 | 7.618 | -0.256 | 1,337 | 1,239 | 39.9 | 3 | 445.7 | 108 | nucl |
| Gorai.003G023900 | CYP71A1 | *Gr_CYP71A1_61* | Chr03 | 2,032,283 | 2,032,962 | - | 680 | 193 | 22.018 | 0.5 | 7.185 | -0.12 | 680 | 582 | 41.6 | 1 | 680 | No intron | mito |
| Gorai.003G023600 | CYP83B1 | *Gr_CYP83B1__62* | Chr03 | 1,986,746 | 1,987,794 | - | 1,049 | 318 | 36.969 | -6 | 5.155 | -0.269 | 957 | 957 | 39.6 | 2 | 478.5 | 92 | nucl |
| Gorai.009G196300 | CYP71A1 | *Gr_CYP71A1_63* | Chr09 | 15,117,631 | 15,118,176 | + | 546 | 181 | 20.529 | 2.5 | 8.174 | -0.149 | 546 | 546 | 38.1 | 1 | 546 | No intron | nucl |
| Gorai.011G225300 | CYP83B1 | *Gr_CYP83B1_64* | Chr11 | 53,673,378 | 53,675,071 | + | 1,694 | 499 | 57.116 | 6.5 | 7.499 | -0.027 | 1,612 | 1,500 | 41.2 | 2 | 806 | 82 | nucl |
| Gorai.011G225400 | CYP83B1 | *Gr_CYP83B1_65* | Chr11 | 53,680,653 | 53,682,552 | + | 1,900 | 505 | 57.855 | 9.5 | 8.155 | 0.011 | 1,820 | 1,518 | 41.2 | 2 | 910 | 80 | nucl |
| Gorai.011G225500 | CYP83B1 | *Gr_CYP83B1_66* | Chr11 | 53,698,289 | 53,700,195 | + | 1,907 | 510 | 58.915 | 9 | 7.945 | -0.085 | 1,816 | 1,533 | 40 | 2 | 908 | 91 | E.R. |
| Gorai.011G225000 | CYP83B1 | *Gr_CYP83B1_67* | Chr11 | 53,598,219 | 53,600,267 | - | 2,049 | 503 | 57.659 | 3.5 | 7.067 | -0.056 | 1,959 | 1,512 | 39.6 | 2 | 979.5 | 90 | E.R. |
| Gorai.011G224900 | CYP83B1 | *Gr_CYP83B1_68* | Chr11 | 53,572,381 | 53,574,142 | - | 1,762 | 505 | 57.675 | 9.5 | 8.281 | 0.004 | 1,663 | 1,518 | 40.7 | 2 | 831.5 | 99 | cyto |
| Gorai.011G225100 | CYP71B36 | *Gr_CYP71B36_69* | Chr11 | 53,607,407 | 53,608,724 | - | 1,318 | 369 | 42.505 | 3 | 7.405 | -0.003 | 1,110 | 1,110 | 40.6 | 3 | 370 | 104 | nucl |
| Gorai.011G225200 | CYP83B1 | *Gr_CYP83B1_70* | Chr11 | 53,629,347 | 53,631,392 | - | 2,046 | 503 | 57.648 | 10.5 | 8.525 | -0.017 | 1,936 | 1,512 | 41 | 2 | 968 | 110 | E.R. |
| Gorai.007G236600 | CYP71E7 | *Gr_CYP71E7_71* | Chr07 | 31,981,735 | 31,983,257 | + | 1,523 | 377 | 42.776 | -1 | 6.321 | -0.147 | 1,134 | 1,134 | 43.3 | 6 | 189 | 77.8 | E.R. |
| Gorai.007G236700 | CYP71E7 | *Gr_CYP71E7_72* | Chr07 | 32,013,822 | 32,015,265 | + | 1,444 | 384 | 44.002 | 12 | 9.417 | -0.293 | 1,155 | 1,155 | 41.3 | 4 | 288.8 | 96.3 | nucl |
| Gorai.008G161500 | CYP71B36 | *Gr_CYP71B36_73* | Chr08 | 42,859,268 | 42,864,298 | + | 5,031 | 518 | 58.993 | 13 | 8.452 | -0.005 | 2,299 | 1,557 | 45.8 | 2 | 1,149.50 | 2,732.00 | nucl |
| Gorai.005G176000 | CYP71B2 | *Gr_CYP71B2_74* | Chr05 | 51,623,133 | 51,633,327 | + | 10,195 | 428 | 48.63 | 12 | 9.118 | -0.119 | 1,287 | 1,287 | 41.7 | 6 | 214.5 | 1,781.60 | E.R. |
| Gorai.005G176100 | CYP71B36 | *Gr_CYP71B36_75* | Chr05 | 51,656,217 | 51,657,464 | - | 1,248 | 387 | 44.307 | -2.5 | 5.967 | -0.127 | 1,164 | 1,164 | 40 | 2 | 582 | 84 | nucl |
| Gorai.003G107400 | CYP71A1 | *Gr_CYP71A1_76* | Chr03 | 32,957,667 | 32,959,689 | + | 2,023 | 508 | 57.858 | 3 | 6.835 | -0.075 | 1,948 | 1,527 | 45 | 2 | 974 | 75 | E.R. |
| Gorai.011G288200 | CYP71A1 | *Gr_CYP71A1_77* | Chr11 | 61,981,874 | 61,985,074 | + | 3,201 | 504 | 57.598 | 6.5 | 7.287 | -0.123 | 2,139 | 1,515 | 44.7 | 2 | 1,069.50 | 1,062.00 | vacu |
| Gorai.N016100 | CYP71A9 | *Gr_CYP71A9_78* | scaffold_122 | 5,843 | 8,827 | + | 2,985 | 502 | 57.228 | 5.5 | 7.127 | -0.128 | 1,866 | 1,509 | 45.5 | 2 | 933 | 1,119.00 | vacu |
| Gorai.010G088700 | CYP75B1 | *Gr_CYP75B1_79* | Chr10 | 13,565,665 | 13,568,062 | - | 2,398 | 516 | 59.42 | 12.5 | 9.062 | -0.244 | 1,864 | 1,551 | 45.6 | 2 | 932 | 534 | E.R. |
| Gorai.011G060100 | CYP71A1 | *Gr_CYP71A1_80* | Chr11 | 4,865,995 | 4,874,265 | - | 8,271 | 510 | 58.418 | 10.5 | 9.037 | -0.247 | 1,753 | 1,533 | 46.8 | 3 | 584.3 | 3,199.50 | E.R. |
| Gorai.010G175000 | CYP75A3 | *Gr_CYP75A3_81* | Chr10 | 51,052,623 | 51,054,647 | + | 2,025 | 172 | 19.769 | 3 | 8.681 | -0.042 | 519 | 519 | 42.6 | 5 | 103.8 | 376.5 | cyto |
| Gorai.007G151200 | CYP71A1 | *Gr_CYP71A1_82* | Chr07 | 12,962,565 | 12,965,526 | + | 2,962 | 511 | 58.404 | 7.5 | 8.121 | -0.228 | 2,031 | 1,536 | 45.4 | 2 | 1,015.50 | 931 | E.R. |
| Gorai.004G202000 | CYP75B1 | *Gr_CYP75B1_83* | Chr04 | 53,047,467 | 53,049,398 | + | 1,932 | 502 | 56.787 | 16.5 | 9.469 | -0.056 | 1,813 | 1,509 | 43.8 | 2 | 906.5 | 119 | plas |
| Gorai.003G165800 | CYP75B1 | *Gr_CYP75B1_84* | Chr03 | 43,581,094 | 43,583,162 | + | 2,069 | 536 | 60.138 | 3.5 | 7.25 | -0.102 | 1,977 | 1,611 | 43.8 | 2 | 988.5 | 92 | nucl |
| Gorai.003G166100 | CYP75B1 | *Gr_CYP75B1_85* | Chr03 | 43,609,010 | 43,610,970 | + | 1,961 | 475 | 53.145 | 6.5 | 8.117 | -0.033 | 1,687 | 1,428 | 44.4 | 2 | 843.5 | 274 | nucl |
| Gorai.003G165000 | CYP75A2 | *Gr_CYP75A2_86* | Chr03 | 43,474,296 | 43,477,274 | + | 2,979 | 532 | 60.015 | 11 | 8.625 | -0.101 | 1,599 | 1,599 | 42 | 2 | 799.5 | 1,380.00 | nucl |
| Gorai.003G165400 | CYP75A2 | *Gr_CYP75A2_87* | Chr03 | 43,545,631 | 43,548,906 | + | 3,276 | 502 | 56.536 | 6 | 7.623 | -0.137 | 1,886 | 1,509 | 42.3 | 2 | 943 | 1,390.00 | nucl |
| Gorai.003G165100 | CYP75B1 | *Gr_CYP75B1_88* | Chr03 | 43,510,350 | 43,513,300 | + | 2,951 | 439 | 49.482 | 2 | 6.947 | -0.09 | 1,787 | 1,320 | 42.9 | 2 | 893.5 | 1,164.00 | nucl |
| Gorai.003G165500 | CYP75A2 | *Gr_CYP75A2_89* | Chr03 | 43,555,910 | 43,558,697 | + | 2,788 | 532 | 60.061 | 6 | 7.932 | -0.044 | 2,014 | 1,599 | 43.5 | 3 | 671.3 | 387 | nucl |
| Gorai.003G165600 | CYP75A2 | *Gr_CYP75A2_90* | Chr03 | 43,561,922 | 43,564,436 | - | 2,515 | 527 | 59.686 | 8 | 8.68 | -0.087 | 2,435 | 1,584 | 42.3 | 2 | 1,217.50 | 80 | nucl |
| Gorai.003G165300 | CYP81E8 | *Gr_CYP81E8_91* | Chr03 | 43,539,662 | 43,540,971 | - | 1,310 | 319 | 35.78 | -4 | 5.24 | 0.046 | 960 | 960 | 42.7 | 5 | 192 | 87.5 | nucl |
| Gorai.003G165700 | CYP75A2 | *Gr_CYP75A2_92* | Chr03 | 43,573,578 | 43,575,868 | - | 2,291 | 399 | 45.124 | -1 | 6.325 | -0.075 | 1,200 | 1,200 | 42.1 | 3 | 400 | 88.5 | nucl |
| Gorai.008G239200 | CYP93A2 | *Gr_CYP93A2_93* | Chr08 | 52,595,427 | 52,597,939 | + | 2,513 | 502 | 56.173 | 4 | 7.344 | -0.032 | 2,098 | 1,509 | 46.3 | 2 | 1,049.00 | 415 | E.R. |
| Gorai.011G254100 | CYP93A2 | *Gr_CYP93A2_94* | Chr11 | 58,266,484 | 58,269,567 | + | 3,084 | 533 | 60.044 | 13 | 9.311 | -0.035 | 1,815 | 1,602 | 46.9 | 2 | 907.5 | 1,269.00 | E.R. |
| Gorai.008G284600 | CYP93A2 | *Gr_CYP93A2_95* | Chr08 | 56,035,884 | 56,039,116 | + | 3,233 | 524 | 59.37 | 15.5 | 9.685 | -0.03 | 1,755 | 1,575 | 44.9 | 2 | 877.5 | 1,478.00 | nucl |
| Gorai.008G284500 | CYP76C3 | *Gr_CYP76C3_96* | Chr08 | 56,031,566 | 56,033,716 | + | 2,151 | 350 | 40.264 | -5 | 5.316 | -0.141 | 1,197 | 1,053 | 42.4 | 2 | 598.5 | 954 | nucl |
| Gorai.008G284200 | CYP76C3 | *Gr_CYP76C3_97* | Chr08 | 56,010,660 | 56,013,359 | + | 2,700 | 533 | 60.908 | 6.5 | 8.101 | -0.016 | 1,900 | 1,602 | 44.8 | 2 | 950 | 800 | nucl |
| Gorai.008G284300 | CYP76C3 | *Gr_CYP76C3_98* | Chr08 | 56,027,611 | 56,030,184 | + | 2,574 | 531 | 60.401 | 9.5 | 8.973 | -0.014 | 1,727 | 1,596 | 45.2 | 2 | 863.5 | 847 | nucl |
| Gorai.007G008700 | CYP76C1 | *Gr_CYP76C1_99* | Chr07 | 682,277 | 683,968 | - | 1,692 | 533 | 60.278 | 7 | 8.012 | -0.041 | 1,619 | 1,602 | 44.2 | 2 | 809.5 | 73 | nucl |
| Gorai.007G008500 | CYP76B6 | *Gr_CYP76B6_100* | Chr07 | 663,866 | 665,459 | - | 1,594 | 478 | 54.349 | 8 | 8.786 | -0.116 | 1,502 | 1,437 | 42.5 | 2 | 751 | 92 | E.R. |
| Gorai.007G008600 | CYP736A12 | *Gr_ CYP736A12_101* | Chr07 | 671,128 | 672,281 | - | 1,154 | 328 | 37.467 | 5 | 7.764 | 0.05 | 987 | 987 | 43.2 | 3 | 329 | 83.5 | E.R. |
| Gorai.009G424900 | CYP76B6 | *Gr_CYP76B6_102* | Chr09 | 66,651,199 | 66,653,034 | + | 1,836 | 522 | 59.014 | 6 | 7.715 | -0.03 | 1,758 | 1,569 | 44.3 | 2 | 879 | 78 | E.R. |
| Gorai.009G434800 | CYP76A2 | *Gr_CYP76A2_103* | Chr09 | 68,366,672 | 68,368,084 | - | 1,413 | 330 | 37.628 | -5.5 | 5.282 | -0.153 | 1,334 | 993 | 42.6 | 2 | 667 | 79 | nucl |
| Gorai.N008900 | CYP76C1 | *Gr_CYP76C1_104* | scaffold_19 | 89,497 | 91,210 | - | 1,714 | 508 | 57.445 | 6.5 | 7.698 | -0.026 | 1,594 | 1,527 | 44.6 | 3 | 531.3 | 60 | E.R. |
| Gorai.009G424600 | CYP80B3 | *Gr_CYP80B3_105* | Chr09 | 66,531,967 | 66,532,858 | + | 892 | 174 | 19.924 | 4.5 | 7.771 | -0.141 | 525 | 525 | 44.2 | 2 | 262.5 | 367 | E.R. |
| Gorai.009G424500 | CYP76B1 | *Gr_CYP76B1_106* | Chr09 | 66,526,334 | 66,528,736 | + | 2,403 | 363 | 41.237 | 11 | 8.842 | 0.085 | 1,117 | 1,092 | 45.4 | 3 | 372.3 | 643 | E.R. |
| Gorai.009G424800 | CYP76B6 | *Gr_CYP76B6__107* | Chr09 | 66,628,119 | 66,629,979 | + | 1,861 | 522 | 59.192 | 7.5 | 8.076 | -0.049 | 1,782 | 1,569 | 45.4 | 2 | 891 | 79 | E.R. |
| Gorai.009G424700 | CYP76C3 | *Gr_CYP76C3_108* | Chr09 | 66,554,159 | 66,555,103 | + | 945 | 258 | 29.334 | 1 | 6.801 | -0.151 | 866 | 774 | 42.8 | 2 | 433 | 79 | nucl |
| Gorai.N008800 | CYP76B1 | *Gr_CYP76B1_109* | scaffold_19 | 17,407 | 18,398 | - | 992 | 302 | 34.656 | 12 | 10.15 | -0.148 | 992 | 909 | 43.5 | 1 | 992 | No intron | E.R. |
| Gorai.007G371400 | CYP76A2 | *Gr_CYP76A2_110* | Chr07 | 60,432,969 | 60,435,092 | - | 2,124 | 513 | 58.415 | 16 | 8.718 | -0.187 | 1,695 | 1,542 | 44.8 | 2 | 847.5 | 429 | nucl |
| Gorai.009G409000 | CYP76A2 | *Gr_CYP76A2_111* | Chr09 | 61,390,989 | 61,392,886 | - | 1,898 | 512 | 58.395 | 12.5 | 9.103 | -0.138 | 1,822 | 1,539 | 44.4 | 2 | 911 | 76 | E.R. |
| Gorai.008G222500 | CYP76B6 | *Gr_CYP76B6_112* | Chr08 | 50,864,550 | 50,866,120 | - | 1,571 | 499 | 56.629 | 12.5 | 9.038 | -0.137 | 1,500 | 1,500 | 44.2 | 2 | 750 | 71 | E.R. |
| Gorai.008G222600 | CYP76B6 | *Gr_CYP76B6_113* | Chr08 | 50,866,449 | 50,869,198 | - | 2,750 | 501 | 57.474 | 13.5 | 8.553 | -0.185 | 1,506 | 1,506 | 45.5 | 2 | 753 | 1,244.00 | plas |
| Gorai.006G211000 | CYP76B6 | *Gr_CYP76B6_114* | Chr06 | 46,571,139 | 46,573,307 | - | 2,169 | 503 | 56.548 | 12 | 9.123 | -0.097 | 1,829 | 1,512 | 42.8 | 2 | 914.5 | 340 | nucl |
| Gorai.006G211200 | CYP76B10 | *Gr_CYP76B10_115* | Chr06 | 46,582,316 | 46,585,608 | - | 3,293 | 370 | 42.347 | 14.5 | 9.907 | -0.085 | 1,113 | 1,113 | 40.4 | 6 | 185.5 | 436 | nucl |
| Gorai.006G211100 | CYP76B6 | *Gr_CYP76B6_116* | Chr06 | 46,575,262 | 46,577,298 | - | 2,037 | 500 | 56.168 | 10 | 8.66 | -0.067 | 1,503 | 1,503 | 42.4 | 2 | 751.5 | 534 | nucl |
| Gorai.004G205400 | CYP76B6 | *Gr_CYP76B6_117* | Chr04 | 53,569,690 | 53,571,257 | + | 1,568 | 454 | 52.044 | 10.5 | 8.358 | -0.171 | 1,365 | 1,365 | 42.4 | 3 | 455 | 101.5 | nucl |
| Gorai.004G204900 | CYP76B6 | *Gr_CYP76B6_118* | Chr04 | 53,490,999 | 53,492,573 | + | 1,575 | 486 | 55.502 | 11 | 8.358 | -0.121 | 1,461 | 1,461 | 42 | 3 | 487 | 57 | nucl |
| Gorai.004G204700 | CYP76B10 | *Gr_CYP76B10_119* | Chr04 | 53,434,845 | 53,435,684 | + | 840 | 251 | 29.005 | -4 | 5.415 | -0.374 | 756 | 756 | 39 | 2 | 378 | 84 | nucl |
| Gorai.004G204800 | CYP76B6 | *Gr_CYP76B6_120* | Chr04 | 53,483,314 | 53,485,325 | + | 2,012 | 497 | 56.287 | 4 | 7.038 | -0.068 | 1,918 | 1,494 | 41 | 2 | 959 | 94 | cyto |
| Gorai.004G205000 | CYP76B6 | *Gr_CYP76B6_121* | Chr04 | 53,512,555 | 53,514,321 | + | 1,767 | 446 | 51.088 | 12.5 | 8.496 | -0.122 | 1,602 | 1,341 | 42 | 3 | 534 | 82.5 | cyto |
| Gorai.009G071700 | CYP75A1 | *Gr_CYP75A1_122* | Chr09 | 5,088,970 | 5,091,231 | - | 2,262 | 527 | 59.521 | 16.5 | 9.439 | -0.064 | 2,110 | 1,584 | 47.2 | 3 | 703.3 | 76 | nucl |
| Gorai.001G134900 | CYP75A3 | *Gr_CYP75A3_123* | Chr01 | 17,605,306 | 17,607,373 | + | 2,068 | 510 | 57.214 | 13.5 | 9.356 | -0.114 | 1,987 | 1,533 | 49.2 | 2 | 993.5 | 81 | plas |
| Gorai.008G198200 | CYP75B2 | *Gr_CYP75B2_124* | Chr08 | 48,277,406 | 48,281,826 | + | 4,421 | 510 | 56.814 | 14 | 9.495 | -0.091 | 2,001 | 1,533 | 52 | 3 | 667 | 1,210.00 | plas |
| Gorai.008G125800 | CYP703A2 | *Gr_CYP703A2_125* | Chr08 | 36,600,306 | 36,602,428 | - | 2,123 | 537 | 61.431 | 9 | 7.516 | -0.217 | 1,673 | 1,614 | 48.5 | 2 | 836.5 | 450 | nucl |
| Gorai.009G293900 | CYP79A2 | *Gr_CYP79A2_126* | Chr09 | 25,518,040 | 25,519,986 | - | 1,947 | 514 | 57.628 | 15.5 | 9.147 | -0.179 | 1,735 | 1,545 | 44.6 | 4 | 433.8 | 70.7 | nucl |
| Gorai.011G209500 | CYP79A2 | *Gr_CYP79A2_127* | Chr11 | 50,382,075 | 50,384,372 | - | 2,298 | 555 | 62.658 | 9.5 | 8.225 | -0.175 | 2,092 | 1,668 | 46.6 | 2 | 1,046.00 | 206 | E.R. |
| Gorai.009G113900 | CYP79D4 | *Gr_CYP79D4_128* | Chr09 | 8,333,019 | 8,335,732 | + | 2,714 | 550 | 61.691 | 6.5 | 7.536 | -0.029 | 2,119 | 1,653 | 48.8 | 2 | 1,059.50 | 595 | plas |
| Gorai.001G222700 | CYP79D4 | *Gr_CYP79D4_129* | Chr01 | 45,231,431 | 45,233,509 | + | 2,079 | 549 | 62.352 | 5.5 | 7.1 | -0.231 | 1,911 | 1,650 | 43.9 | 2 | 955.5 | 168 | mito |
| Gorai.001G222800 | CYP79D3 | *Gr_CYP79D3_130* | Chr01 | 45,431,226 | 45,432,987 | + | 1,762 | 511 | 57.856 | 3.5 | 6.901 | -0.194 | 1,536 | 1,536 | 44.6 | 3 | 512 | 113 | nucl |
| Gorai.003G047300 | CYP78A7 | *Gr_CYP78A7_131* | Chr03 | 6,403,456 | 6,405,568 | - | 2,113 | 537 | 60.599 | 12 | 8.914 | -0.064 | 2,015 | 1,614 | 49.1 | 2 | 1,007.50 | 98 | plas |
| Gorai.010G130700 | CYP78A7 | *Gr_CYP78A7_132* | Chr10 | 28,304,618 | 28,306,331 | - | 1,714 | 544 | 60.34 | 9.5 | 8.329 | 0.05 | 1,635 | 1,635 | 47.1 | 2 | 817.5 | 79 | nucl |
| Gorai.007G111300 | CYP78A3 | *Gr_CYP78A3_133* | Chr07 | 8,512,466 | 8,515,025 | + | 2,560 | 540 | 60.74 | 17 | 9.103 | 0.001 | 2,355 | 1,623 | 47.7 | 2 | 1,177.50 | 205 | mito |
| Gorai.008G127600 | CYP78A6 | *Gr_CYP78A6_134* | Chr08 | 36,880,573 | 36,882,707 | + | 2,135 | 533 | 60.019 | 13.5 | 8.604 | 0.017 | 2,055 | 1,602 | 45.3 | 2 | 1,027.50 | 80 | nucl |
| Gorai.004G149900 | CYP78A6 | *Gr_CYP78A6_135* | Chr04 | 42,492,093 | 42,495,149 | - | 3,057 | 549 | 62.494 | 15.5 | 9.295 | -0.133 | 2,519 | 1,650 | 48.5 | 3 | 839.7 | 269 | cyto |
| Gorai.008G258700 | CYP78A3 | *Gr_CYP78A3_136* | Chr08 | 53,988,495 | 53,990,766 | - | 2,272 | 530 | 59.617 | 13.5 | 8.697 | 0.016 | 2,030 | 1,593 | 47.2 | 2 | 1,015.00 | 242 | E.R. |
| Gorai.003G077000 | CYP78A6 | *Gr_CYP78A6_137* | Chr03 | 18,739,365 | 18,742,120 | - | 2,756 | 530 | 59.516 | 16 | 9.062 | 0.013 | 2,010 | 1,593 | 48.7 | 2 | 1,005.00 | 746 | E.R. |
| Gorai.004G129700 | CYP78A3 | *Gr_CYP78A3_138* | Chr04 | 34,901,716 | 34,904,005 | + | 2,290 | 533 | 60.076 | 15.5 | 8.772 | -0.034 | 2,110 | 1,602 | 47.5 | 2 | 1,055.00 | 180 | nucl |
| Gorai.005G146000 | CYP78A5 | *Gr_CYP78A5_139* | Chr05 | 39,603,016 | 39,605,133 | + | 2,118 | 518 | 58.663 | 7.5 | 7.482 | 0.019 | 2,017 | 1,557 | 45.7 | 2 | 1,008.50 | 101 | nucl |
| Gorai.013G095000 | CYP78A5 | *Gr_CYP78A5_140* | Chr13 | 15,648,480 | 15,650,443 | + | 1,964 | 518 | 58.272 | 6 | 7.273 | 0.026 | 1,878 | 1,557 | 46.6 | 2 | 939 | 86 | E.R. |
| Gorai.008G176400 | CYP78A5 | *Gr_CYP78A5_141* | Chr08 | 45,278,097 | 45,280,162 | - | 2,066 | 518 | 57.974 | 4.5 | 7.062 | 0.089 | 1,967 | 1,557 | 47.1 | 2 | 983.5 | 99 | nucl |
| Gorai.002G184000 | CYP78A5 | *Gr_CYP78A5_142* | Chr02 | 48,709,672 | 48,712,714 | - | 3,043 | 524 | 59.191 | 20.5 | 9.755 | 0.033 | 1,592 | 1,575 | 45.3 | 2 | 796 | 1,451.00 | E.R. |
| Gorai.012G175600 | CYP78A5 | *Gr_CYP78A5_143* | Chr12 | 34,508,838 | 34,512,503 | + | 3,666 | 548 | 61.972 | 22 | 10.16 | 0.097 | 1,977 | 1,647 | 45.8 | 2 | 988.5 | 1,689.00 | nucl |
| Gorai.002G230900 | CYP98A2 | *Gr_CYP98A2_144* | Chr02 | 59,022,733 | 59,024,713 | + | 1,981 | 508 | 58.062 | 11 | 8.165 | -0.192 | 1,696 | 1,527 | 46.2 | 3 | 565.3 | 142.5 | nucl |
| Gorai.013G000900 | CYP98A2 | *Gr_CYP98A2_145* | Chr13 | 48,309 | 52,500 | + | 4,192 | 510 | 57.914 | 8 | 7.726 | -0.165 | 1,978 | 1,533 | 49.4 | 3 | 659.3 | 1,090.50 | plas |
| Gorai.011G207000 | CYP73A11 | *Gr_CYP73A11_146* | Chr11 | 49,856,270 | 49,859,016 | + | 2,747 | 505 | 58.294 | 15.5 | 9.66 | -0.233 | 1,972 | 1,518 | 47 | 3 | 657.3 | 387.5 | plas |
| Gorai.013G271700 | CYP73A11 | *Gr_CYP73A11_147* | Chr13 | 58,235,813 | 58,238,243 | + | 2,431 | 505 | 58.04 | 13 | 9.431 | -0.27 | 1,829 | 1,518 | 48 | 3 | 609.7 | 301 | E.R. |
| Gorai.009G044000 | CYP711A1 | *Gr_CYP711A1_148* | Chr09 | 3,185,743 | 3,188,379 | - | 2,637 | 539 | 60.504 | 17.5 | 9.511 | -0.128 | 1,820 | 1,620 | 41.9 | 5 | 364 | 204.3 | E.R. |
| Gorai.005G197400 | CYP51G1 | *Gr_CYP51G1_149* | Chr05 | 57,162,298 | 57,165,260 | + | 2,963 | 486 | 55.537 | 11 | 8.851 | -0.189 | 2,081 | 1,461 | 46 | 3 | 693.7 | 441 | nucl |
| Gorai.008G155900 | CYP51G1 | *Gr_CYP51G1_150* | Chr08 | 41,655,053 | 41,658,679 | + | 3,627 | 486 | 55.263 | 12 | 8.914 | -0.158 | 2,265 | 1,461 | 45 | 4 | 566.3 | 454 | nucl |
| Gorai.007G171600 | CYP74B2 | *Gr_CYP74B2_151* | Chr07 | 15,666,530 | 15,676,004 | + | 9,475 | 528 | 58.707 | 9.5 | 8.777 | -0.15 | 1,821 | 1,587 | 48.5 | 3 | 607 | 3,827.00 | plas |
| Gorai.008G044100 | CYP74A2 | *Gr_CYP74A2_152* | Chr08 | 5,920,174 | 5,922,356 | - | 2,183 | 492 | 55.424 | 3.5 | 7.233 | -0.059 | 2,183 | 1,479 | 42.7 | 1 | 2,183.00 | No intron | nucl |
| Gorai.010G013600 | CYP74A | *Gr_CYP74A_153* | Chr10 | 1,010,736 | 1,012,694 | + | 1,959 | 495 | 55.803 | 2.5 | 7.181 | -0.181 | 1,959 | 1,488 | 42.3 | 1 | 1,959.00 | No intron | plas |
| Gorai.005G105800 | CYP74A | *Gr_CYP74A_154* | Chr05 | 18,813,774 | 18,815,885 | - | 2,112 | 510 | 57.582 | 10.5 | 8.958 | -0.21 | 2,009 | 1,533 | 46.5 | 1 | 2,009.00 | No intron | nucl |
| Gorai.009G274700 | CYP74A | *Gr_CYP74A_155* | Chr09 | 23,018,514 | 23,020,894 | + | 2,381 | 523 | 58.708 | 18 | 9.866 | -0.259 | 2,381 | 1,572 | 46.8 | 1 | 2,381.00 | No intron | E.R. |
| Gorai.010G013700 | CYP74A | *Gr_CYP74A_156* | Chr10 | 1,017,459 | 1,019,294 | + | 1,836 | 496 | 56.058 | 4.5 | 7.877 | -0.109 | 1,836 | 1,491 | 42.8 | 1 | 1,836.00 | No intron | E.R. |
| Gorai.010G166000 | CYP710A1 | *Gr_CYP710A1_157* | Chr10 | 47,991,900 | 47,993,411 | - | 1,512 | 503 | 58.296 | 11.5 | 8.709 | -0.021 | 1,512 | 1,512 | 47 | 1 | 1,512.00 | No intron | nucl |
| Gorai.010G166100 | CYP710A1 | *Gr_CYP710A1_158* | Chr10 | 48,046,347 | 48,047,858 | - | 1,512 | 503 | 57.925 | 12.5 | 8.768 | -0.009 | 1,512 | 1,512 | 47.6 | 1 | 1,512.00 | No intron | E.R. |
| Gorai.009G155600 | CYP710A1 | *Gr_CYP710A1_159* | Chr09 | 11,940,658 | 11,942,166 | + | 1,509 | 456 | 52.484 | 7.5 | 8.143 | -0.085 | 1,371 | 1,371 | 47.2 | 4 | 342.8 | 46 | E.R. |
| Gorai.009G155800 | CYP710A1 | *Gr_CYP710A1_160* | Chr09 | 11,954,938 | 11,966,232 | + | 11,295 | 502 | 57.613 | 6.5 | 7.755 | -0.101 | 1,746 | 1,509 | 48.1 | 2 | 873 | 9,549.00 | nucl |
| Gorai.005G079500 | CYP707A3 | *Gr_CYP707A3_161* | Chr05 | 9,208,496 | 9,211,406 | - | 2,911 | 446 | 50.715 | 11 | 9.314 | -0.286 | 1,538 | 1,341 | 40.7 | 10 | 153.8 | 152.6 | nucl |
| Gorai.005G079800 | CYP707A3 | *Gr_CYP707A3_162* | Chr05 | 9,224,167 | 9,226,910 | + | 2,744 | 477 | 54.121 | 13 | 9.461 | -0.165 | 1,638 | 1,434 | 40.9 | 9 | 182 | 138.3 | nucl |
| Gorai.008G065700 | CYP707A3 | *Gr_CYP707A3_163* | Chr08 | 10,560,064 | 10,562,881 | + | 2,818 | 491 | 55.7 | 8 | 8.359 | -0.179 | 1,972 | 1,476 | 42.3 | 9 | 219.1 | 105.8 | nucl |
| Gorai.010G008600 | CYP707A4 | *Gr_CYP707A4_164* | Chr10 | 611,711 | 617,850 | + | 6,140 | 498 | 56.372 | 8.5 | 8.246 | -0.029 | 1,777 | 1,497 | 41.4 | 10 | 177.7 | 484.8 | nucl |
| Gorai.009G211900 | CYP707A4 | *Gr_CYP707A4_165* | Chr09 | 16,399,713 | 16,403,076 | - | 3,364 | 471 | 53.551 | 24.5 | 10.01 | -0.218 | 1,893 | 1,416 | 44.4 | 9 | 210.3 | 183.9 | plas |
| Gorai.005G237800 | CYP707A4 | *Gr_CYP707A4_166* | Chr05 | 61,952,148 | 61,956,240 | + | 4,093 | 474 | 54.264 | 20 | 9.834 | -0.212 | 1,770 | 1,425 | 42.3 | 9 | 196.7 | 290.4 | cyto |
| Gorai.013G044100 | CYP707A4 | *Gr_CYP707A4_167* | Chr13 | 3,822,546 | 3,826,823 | - | 4,278 | 477 | 54.86 | 18 | 9.508 | -0.21 | 1,755 | 1,434 | 42.5 | 9 | 195 | 176.8 | E.R. |
| Gorai.004G177200 | CYP707A1 | *Gr_CYP707A1_168* | Chr04 | 48,354,337 | 48,356,948 | - | 2,612 | 495 | 56.53 | 17.5 | 9.608 | -0.096 | 1,867 | 1,488 | 42.9 | 7 | 266.7 | 124.2 | E.R. |
| Gorai.008G218900 | CYP707A1 | *Gr_CYP707A1_169* | Chr08 | 50,527,220 | 50,529,590 | - | 2,371 | 463 | 52.974 | 21.5 | 9.83 | -0.13 | 1,765 | 1,392 | 43.5 | 7 | 252.1 | 101 | plas |
| Gorai.001G111700 | CYP707A2 | *Gr_CYP707A2_170* | Chr01 | 12,997,815 | 13,002,224 | - | 4,410 | 491 | 56.124 | 24 | 10.01 | -0.235 | 1,751 | 1,476 | 43.4 | 8 | 218.9 | 379.9 | E.R. |
| Gorai.003G129900 | CYP707A2 | *Gr_CYP707A2_171* | Chr03 | 38,368,620 | 38,372,038 | + | 3,419 | 499 | 56.963 | 21 | 9.656 | -0.188 | 2,035 | 1,500 | 43.8 | 8 | 254.4 | 197.7 | plas |
| Gorai.007G203700 | CYP90B1 | *Gr_CYP90B1_172* | Chr07 | 20,904,018 | 20,908,011 | - | 3,994 | 516 | 59.325 | 17 | 9.32 | -0.147 | 2,007 | 1,551 | 41.7 | 7 | 286.7 | 272.8 | nucl |
| Gorai.007G163900 | CYP724B1 | *Gr_CYP724B1_173* | Chr07 | 14,393,275 | 14,396,844 | + | 3,570 | 493 | 55.889 | 11.5 | 7.971 | -0.185 | 1,717 | 1,482 | 41.4 | 9 | 190.8 | 231.6 | nucl |
| Gorai.012G065900 | CYP724B1 | *Gr_CYP724B1_174* | Chr12 | 9,305,651 | 9,308,523 | - | 2,873 | 475 | 54.066 | 19 | 9.467 | -0.016 | 1,600 | 1,428 | 40.8 | 9 | 177.8 | 159.1 | nucl |
| Gorai.010G198500 | CYP90A1 | *Gr_CYP90A1_175* | Chr10 | 55,940,897 | 55,945,621 | - | 4,725 | 472 | 54.02 | 17.5 | 9.77 | -0.149 | 1,874 | 1,419 | 45.2 | 8 | 234.3 | 407.3 | plas |
| Gorai.011G286100 | CYP90A1 | *Gr_CYP90A1_176* | Chr11 | 61,845,824 | 61,850,882 | + | 5,059 | 473 | 54.305 | 20 | 9.905 | -0.185 | 1,948 | 1,422 | 45.2 | 8 | 243.5 | 444.4 | nucl |
| Gorai.009G140600 | CYP90D1 | *Gr_CYP90D1_177* | Chr09 | 10,622,116 | 10,626,271 | - | 4,156 | 487 | 56.023 | 14.5 | 9.424 | -0.077 | 1,647 | 1,464 | 40.6 | 8 | 205.9 | 281.3 | nucl |
| Gorai.003G012100 | CYP90D1 | *Gr_CYP90D1_178* | Chr03 | 773,760 | 781,101 | - | 7,342 | 516 | 58.337 | 9.5 | 8.622 | -0.143 | 2,144 | 1,551 | 41.8 | 9 | 238.2 | 649.8 | nucl |
| Gorai.007G199100 | CYP90D1 | *Gr_CYP90D1_179* | Chr07 | 20,230,350 | 20,234,818 | - | 4,469 | 490 | 56.018 | 11.5 | 8.831 | -0.169 | 2,258 | 1,473 | 42.4 | 9 | 250.9 | 276.4 | cyto |
| Gorai.013G095400 | CYP21A2 | *Gr_CYP21A2 _180* | Chr13 | 15,734,904 | 15,737,518 | + | 2,615 | 466 | 53.562 | 20 | 9.318 | -0.211 | 1,717 | 1,401 | 41.6 | 9 | 190.8 | 112.3 | nucl |
| Gorai.001G090300 | CYP21A2 | *Gr_CYP21A2 _181* | Chr01 | 9,854,839 | 9,858,452 | - | 3,614 | 493 | 56.848 | 18 | 9.689 | -0.157 | 2,089 | 1,482 | 36 | 8 | 261.1 | 208.1 | nucl |
| Gorai.001G208200 | CYP21A2 | *Gr_CYP21A2 _182* | Chr01 | 41,014,295 | 41,017,284 | - | 2,990 | 493 | 56.952 | 16.5 | 9.441 | -0.202 | 1,717 | 1,482 | 39.6 | 8 | 214.6 | 159.6 | nucl |
| Gorai.001G058600 | CYP87A3 | *Gr_CYP87A3_183* | Chr01 | 5,773,510 | 5,778,324 | + | 4,815 | 444 | 50.292 | 11 | 9.018 | -0.137 | 1,640 | 1,335 | 42.4 | 9 | 182.2 | 396.9 | nucl |
| Gorai.003G125500 | CYP87A3 | *Gr_CYP87A3_184* | Chr03 | 37,470,370 | 37,475,826 | + | 5,457 | 472 | 54.043 | 16 | 9.36 | -0.107 | 1,958 | 1,419 | 42.8 | 9 | 217.6 | 437.4 | nucl |
| Gorai.002G062200 | CYP87A3 | *Gr_CYP87A3_185* | Chr02 | 7,341,980 | 7,344,955 | - | 2,976 | 482 | 55.411 | 9.5 | 8.596 | -0.137 | 1,875 | 1,449 | 41.5 | 9 | 208.3 | 137.6 | nucl |
| Gorai.002G062400 | CYP87A3 | *Gr_CYP87A3_186* | Chr02 | 7,370,084 | 7,374,301 | - | 4,218 | 480 | 55.119 | 8.5 | 8.197 | -0.178 | 1,801 | 1,443 | 41.4 | 9 | 200.1 | 302.1 | nucl |
| Gorai.008G279500 | CYP87A3 | *Gr_CYP87A3_187* | Chr08 | 55,666,084 | 55,668,049 | - | 1,966 | 432 | 49.443 | 14 | 9.113 | -0.053 | 1,299 | 1,299 | 40 | 9 | 144.3 | 83.4 | nucl |
| Gorai.008G279700 | CYP87A3 | *Gr_CYP87A3_188* | Chr08 | 55,675,967 | 55,677,645 | + | 1,679 | 340 | 38.366 | 8 | 8.086 | 0.142 | 1,023 | 1,023 | 38.8 | 9 | 113.7 | 82 | nucl |
| Gorai.008G244100 | CYP87A3 | *Gr_CYP87A3_189* | Chr08 | 52,937,507 | 52,939,995 | + | 2,489 | 470 | 53.856 | 13.5 | 9.143 | -0.167 | 1,594 | 1,413 | 42.3 | 9 | 177.1 | 111.9 | nucl |
| Gorai.003G162200 | CYP87A3 | *Gr_CYP87A3_190* | Chr03 | 43,092,329 | 43,094,596 | - | 2,268 | 474 | 54.424 | 20.5 | 9.869 | -0.092 | 1,511 | 1,425 | 42.2 | 9 | 167.9 | 94.6 | nucl |
| Gorai.003G162300 | CYP87A3 | *Gr_CYP87A3_191* | Chr03 | 43,101,605 | 43,104,220 | - | 2,616 | 474 | 54.51 | 13 | 9.305 | -0.146 | 1,840 | 1,425 | 41.3 | 9 | 204.4 | 97 | nucl |
| Gorai.004G187300 | CYP88D6 | *Gr_CYP88D6_192* | Chr04 | 50,249,402 | 50,252,214 | - | 2,813 | 487 | 55.69 | 12.5 | 9.152 | -0.155 | 1,858 | 1,464 | 42.7 | 8 | 232.3 | 136.4 | E.R. |
| Gorai.004G187100 | CYP88D6 | *Gr_CYP88D6_193* | Chr04 | 50,164,019 | 50,166,271 | - | 2,253 | 402 | 46.205 | 12 | 9.353 | -0.19 | 1,209 | 1,209 | 41.9 | 7 | 172.7 | 174 | nucl |
| Gorai.004G187200 | CYP88D6 | *Gr_CYP88D6_194* | Chr04 | 50,193,280 | 50,196,713 | - | 3,434 | 486 | 55.604 | 19 | 9.594 | -0.212 | 1,903 | 1,461 | 41.4 | 8 | 237.9 | 218.7 | nucl |
| Gorai.006G216200 | CYP88D6 | *Gr_CYP88D6_195* | Chr06 | 46,904,990 | 46,909,249 | + | 4,260 | 481 | 54.869 | 17 | 9.362 | -0.247 | 1,638 | 1,446 | 42.2 | 8 | 204.8 | 374.6 | plas |
| Gorai.010G190900 | CYP88A3 | *Gr_CYP88A3_196* | Chr10 | 54,469,534 | 54,474,523 | - | 4,990 | 499 | 57.541 | 11 | 9.168 | -0.249 | 2,860 | 1,500 | 44.2 | 9 | 317.8 | 266.3 | E.R. |
| Gorai.013G013700 | CYP88A3 | *Gr_CYP88A3_197* | Chr13 | 921,295 | 924,312 | - | 3,018 | 368 | 42.796 | 3 | 6.89 | -0.247 | 1,107 | 1,107 | 42.8 | 6 | 184.5 | 382.2 | E.R. |
| Gorai.010G218500 | CYP716A1 | *Gr_CYP716A1_198* | Chr10 | 58,724,700 | 58,728,603 | - | 3,904 | 494 | 55.662 | 11.5 | 9.217 | -0.036 | 1,552 | 1,485 | 43 | 4 | 388 | 784 | nucl |
| Gorai.002G087900 | CYP716B1 | *Gr_CYP716B1_199* | Chr02 | 11,099,296 | 11,101,879 | - | 2,584 | 474 | 54.369 | 24 | 10.19 | -0.11 | 2,273 | 1,425 | 43.5 | 4 | 568.3 | 103.7 | nucl |
| Gorai.011G015500 | CYP716B1 | *Gr_CYP716B1_200* | Chr11 | 1,090,932 | 1,094,092 | - | 3,161 | 474 | 53.975 | 20.5 | 9.726 | -0.093 | 1,611 | 1,425 | 43.8 | 4 | 402.8 | 516.7 | E.R. |
| Gorai.012G041800 | CYP725A2 | *Gr_CYP725A2_201* | Chr12 | 5,231,302 | 5,232,913 | - | 1,612 | 484 | 55.224 | 19.5 | 9.416 | -0.113 | 1,455 | 1,455 | 44.1 | 3 | 485 | 78.5 | E.R. |
| Gorai.011G006100 | CYP716A52v2 | *Gr_CYP716A52v2_202* | Chr11 | 497,239 | 500,430 | + | 3,192 | 486 | 55.224 | 12.5 | 9.31 | -0.221 | 1,749 | 1,461 | 44.8 | 3 | 583 | 721.5 | plas |
| Gorai.013G135000 | CYP716A52v2 | *Gr_CYP716A52v2_203* | Chr13 | 35,941,693 | 35,943,503 | - | 1,811 | 391 | 44.508 | 9.5 | 8.875 | -0.227 | 1,632 | 1,176 | 45.7 | 3 | 544 | 89.5 | E.R. |
| Gorai.013G137300 | CYP716A52v2 | *Gr_CYP716A52v2_204* | Chr13 | 36,716,216 | 36,717,842 | + | 1,627 | 482 | 54.575 | 8 | 8.486 | -0.21 | 1,449 | 1,449 | 46.4 | 3 | 483 | 89 | plas |
| Gorai.004G020800 | CYP716A52v2 | *Gr_CYP716A52v2_205* | Chr04 | 1,558,275 | 1,563,340 | + | 5,066 | 480 | 54.705 | 15 | 9.163 | -0.099 | 1,641 | 1,443 | 43.2 | 3 | 547 | 1,712.50 | nucl |
| Gorai.009G120200 | CYP716A52v2 | *Gr_CYP716A52v2_206* | Chr09 | 8,891,627 | 8,894,187 | - | 2,561 | 476 | 54.224 | 10.5 | 8.984 | -0.168 | 1,599 | 1,431 | 46.3 | 3 | 533 | 481 | plas |
| Gorai.010G215500 | CYP78A7 | *Gr_CYP78A7_207* | Chr10 | 58,282,436 | 58,286,215 | - | 3,780 | 559 | 63.328 | 19.5 | 9.245 | -0.137 | 2,409 | 1,680 | 40.4 | 7 | 344.1 | 228.5 | nucl |
| Gorai.011G227100 | CYP94A1 | *Gr_CYP94A1_208* | Chr11 | 53,981,225 | 53,983,280 | + | 2,056 | 502 | 57.884 | 9 | 8.15 | -0.221 | 2,056 | 1,509 | 42.4 | 1 | 2,056.00 | No intron | nucl |
| Gorai.004G219500 | CYP94A1 | *Gr_CYP94A1_209* | Chr04 | 55,354,940 | 55,356,811 | + | 1,872 | 506 | 58.221 | 12.5 | 9.288 | -0.204 | 1,872 | 1,521 | 45.4 | 1 | 1,872.00 | No intron | E.R. |
| Gorai.013G103800 | CYP94A1 | *Gr_CYP94A1_210* | Chr13 | 20,549,992 | 20,551,603 | - | 1,612 | 507 | 57.901 | 12.5 | 9.173 | -0.159 | 1,612 | 1,524 | 47.1 | 1 | 1,612.00 | No intron | cyto |
| Gorai.006G111000 | CYP94C1 | *Gr_CYP94C1_211* | Chr06 | 35,743,110 | 35,744,916 | + | 1,807 | 499 | 57.052 | 9 | 8.064 | -0.043 | 1,807 | 1,500 | 44.2 | 1 | 1,807.00 | No intron | nucl |
| Gorai.004G009100 | CYP94C1 | *Gr_CYP94C1_212* | Chr04 | 638,075 | 639,784 | + | 1,710 | 512 | 58.453 | 14 | 8.758 | -0.032 | 1,710 | 1,539 | 41.4 | 1 | 1,710.00 | No intron | E.R. |
| Gorai.009G086900 | CYP94C1 | *Gr_CYP94C1_213* | Chr09 | 6,316,774 | 6,318,560 | - | 1,787 | 485 | 55.319 | 14 | 8.757 | -0.174 | 1,458 | 1,458 | 45.7 | 3 | 486 | 164.5 | E.R. |
| Gorai.006G156400 | CYP94C1 | *Gr_CYP94C1__214* | Chr06 | 41,520,622 | 41,522,202 | + | 1,581 | 526 | 60.522 | 14 | 8.525 | 0.018 | 1,581 | 1,581 | 44.4 | 1 | 1,581.00 | No intron | cyto |
| Gorai.002G241100 | CYP94C1 | *Gr_CYP94C1_215* | Chr02 | 60,454,083 | 60,455,817 | + | 1,735 | 489 | 56.392 | 14.5 | 8.784 | -0.04 | 1,735 | 1,470 | 37.8 | 1 | 1,735.00 | No intron | plas |
| Gorai.009G405300 | CYP94C1 | *Gr_CYP94C1_216* | Chr09 | 59,363,884 | 59,366,084 | + | 2,201 | 500 | 57.195 | 11 | 8.467 | 0.01 | 2,201 | 1,503 | 46.9 | 1 | 2,201.00 | No intron | plas |
| Gorai.003G120800 | CYP94B3 | *Gr_CYP94B3_217* | Chr03 | 36,197,433 | 36,198,965 | - | 1,533 | 510 | 58.378 | 10.5 | 8.409 | -0.035 | 1,533 | 1,533 | 45.3 | 1 | 1,533.00 | No intron | mito |
| Gorai.007G042500 | CYP94B3 | *Gr_CYP94B3_218* | Chr07 | 2,953,515 | 2,955,309 | - | 1,795 | 515 | 57.835 | 7 | 8.142 | -0.002 | 1,795 | 1,548 | 48.8 | 1 | 1,795.00 | No intron | E.R. |
| Gorai.006G041600 | CYP94B3 | *Gr_CYP94B3_219* | Chr06 | 11,585,311 | 11,586,843 | - | 1,533 | 510 | 58.21 | 12.5 | 8.763 | -0.097 | 1,533 | 1,533 | 43.2 | 1 | 1,533.00 | No intron | nucl |
| Gorai.012G087200 | CYP94B3 | *Gr_CYP94B3_220* | Chr12 | 14,893,901 | 14,895,427 | + | 1,527 | 508 | 57.74 | 8 | 8.012 | -0.003 | 1,527 | 1,527 | 43.6 | 1 | 1,527.00 | No intron | nucl |
| Gorai.004G014400 | CYP94A1 | *Gr_CYP94A1_221* | Chr04 | 1,022,144 | 1,023,724 | - | 1,581 | 526 | 60.161 | 12 | 8.605 | -0.176 | 1,581 | 1,581 | 44.9 | 1 | 1,581.00 | No intron | plas |
| Gorai.003G123900 | CYP86A1 | *Gr_CYP86A1_222* | Chr03 | 37,010,794 | 37,012,329 | - | 1,536 | 511 | 58.698 | 10.5 | 8.858 | -0.179 | 1,536 | 1,536 | 46.7 | 1 | 1,536.00 | No intron | nucl |
| Gorai.001G115100 | CYP86A1 | *Gr_CYP86A1_223* | Chr01 | 13,512,518 | 13,514,281 | + | 1,764 | 514 | 58.559 | 19 | 9.79 | -0.178 | 1,764 | 1,545 | 46.9 | 1 | 1,764.00 | No intron | E.R. |
| Gorai.009G092900 | CYP86A1 | *Gr_CYP86A1_224* | Chr09 | 6,794,283 | 6,795,818 | - | 1,536 | 511 | 58.626 | 16 | 9.589 | -0.166 | 1,536 | 1,536 | 47.2 | 1 | 1,536.00 | No intron | E.R. |
| Gorai.004G159000 | CYP86A8 | *Gr_CYP86A8_225* | Chr04 | 44,812,797 | 44,814,891 | - | 2,095 | 527 | 60.014 | 9 | 7.877 | -0.086 | 2,095 | 1,584 | 48.7 | 1 | 2,095.00 | No intron | nucl |
| Gorai.007G100300 | CYP86A8 | *Gr_CYP86A8_226* | Chr07 | 7,422,329 | 7,424,516 | - | 2,188 | 528 | 59.808 | 10 | 8.208 | -0.13 | 2,188 | 1,587 | 49.6 | 1 | 2,188.00 | No intron | nucl |
| Gorai.004G213300 | CYP86A22 | *Gr_CYP86A22_227* | Chr04 | 54,612,149 | 54,613,753 | - | 1,605 | 534 | 60.981 | 12 | 8.522 | -0.135 | 1,605 | 1,605 | 45.1 | 1 | 1,605.00 | No intron | nucl |
| Gorai.008G247600 | CYP86A22 | *Gr_CYP86A22_228* | Chr08 | 53,182,932 | 53,185,762 | - | 2,831 | 562 | 64.517 | 18.5 | 9.276 | -0.185 | 2,156 | 1,689 | 47.6 | 2 | 1,078.00 | 675 | E.R. |
| Gorai.005G175500 | CYP86B1 | *Gr_CYP86B1_229* | Chr05 | 51,466,408 | 51,468,191 | + | 1,784 | 525 | 60.361 | 22 | 9.655 | -0.069 | 1,784 | 1,578 | 42.6 | 1 | 1,784.00 | No intron | nucl |
| Gorai.012G133700 | CYP86B1 | *Gr_CYP86B1_230* | Chr12 | 30,560,751 | 30,563,104 | - | 2,354 | 547 | 63.011 | 14 | 8.979 | -0.171 | 2,045 | 1,644 | 45.2 | 2 | 1,022.50 | 309 | nucl |
| Gorai.013G069900 | CYP86B1 | *Gr_CYP86B1_231* | Chr13 | 8,085,846 | 8,088,501 | + | 2,656 | 453 | 51.865 | 6.5 | 8.12 | -0.121 | 1,362 | 1,362 | 45.6 | 6 | 227 | 258.8 | E.R. |
| Gorai.006G032500 | CYP96A15 | *Gr_CYP96A15_232* | Chr06 | 8,378,955 | 8,380,464 | - | 1,510 | 485 | 56.333 | 18.5 | 8.945 | -0.173 | 1,458 | 1,458 | 42 | 2 | 729 | 52 | nucl |
| Gorai.006G032600 | CYP96A15 | *Gr_CYP96A15_233* | Chr06 | 8,421,127 | 8,422,639 | - | 1,513 | 473 | 54.366 | 14.5 | 8.411 | -0.16 | 1,422 | 1,422 | 43.2 | 2 | 711 | 91 | nucl |
| Gorai.007G246400 | CYP96A15 | *Gr_CYP96A15_234* | Chr07 | 37,669,340 | 37,671,429 | + | 2,090 | 507 | 58.982 | 12.5 | 8.247 | -0.173 | 2,090 | 1,524 | 42 | 1 | 2,090.00 | No intron | nucl |
| Gorai.007G244800 | CYP96A15 | *Gr_CYP96A15_235* | Chr07 | 36,484,986 | 36,486,590 | - | 1,605 | 499 | 57.498 | 13.5 | 8.406 | -0.064 | 1,605 | 1,500 | 43.2 | 1 | 1,605.00 | No intron | cyto |
| Gorai.011G113900 | CYP96A15 | *Gr_CYP96A15_236* | Chr11 | 14,602,570 | 14,604,090 | - | 1,521 | 506 | 58.117 | 11 | 8.212 | -0.07 | 1,521 | 1,521 | 43.7 | 1 | 1,521.00 | No intron | cyto |
| Gorai.013G088800 | CYP96A15 | *Gr_CYP96A15_237* | Chr13 | 13,872,543 | 13,874,186 | + | 1,644 | 498 | 56.796 | 17 | 8.748 | -0.115 | 1,644 | 1,497 | 43.7 | 1 | 1,644.00 | No intron | E.R. |
| Gorai.008G026000 | CYP96A15 | *Gr_CYP96A15_238* | Chr08 | 3,047,432 | 3,048,880 | - | 1,449 | 374 | 42.761 | 15 | 8.905 | -0.11 | 1,125 | 1,125 | 42.9 | 3 | 375 | 162 | cyto |
| Gorai.012G021500 | CYP96A15 | *Gr_CYP96A15_239* | Chr12 | 2,641,508 | 2,643,060 | + | 1,553 | 511 | 59.006 | 21.5 | 9.543 | -0.197 | 1,553 | 1,536 | 42.4 | 1 | 1,553.00 | No intron | plas |
| Gorai.012G021600 | CYP96A15 | *Gr_CYP96A15_240* | Chr12 | 2,657,538 | 2,662,658 | + | 5,121 | 511 | 58.951 | 20.5 | 9.448 | -0.203 | 1,954 | 1,536 | 42.8 | 1 | 1,954.00 | No intron | cyto |
| Gorai.012G021700 | CYP96A15 | *Gr_CYP96A15_241* | Chr12 | 2,672,839 | 2,674,401 | - | 1,563 | 511 | 59.059 | 24 | 9.739 | -0.216 | 1,563 | 1,536 | 42.6 | 1 | 1,563.00 | No intron | cyto |
| Gorai.008G025900 | CYP96A15 | *Gr_CYP96A15_242* | Chr08 | 3,014,817 | 3,016,356 | - | 1,540 | 495 | 56.946 | 15 | 8.675 | -0.2 | 1,488 | 1,488 | 43.1 | 2 | 744 | 52 | plas |
| Gorai.008G026100 | CYP96A15 | *Gr_CYP96A15_243* | Chr08 | 3,057,272 | 3,058,804 | - | 1,533 | 510 | 59.041 | 17 | 9.049 | -0.242 | 1,533 | 1,533 | 43.3 | 1 | 1,533.00 | No intron | plas |
| Gorai.008G180000 | CYP704B1 | *Gr_CYP704B1_244* | Chr08 | 45,729,002 | 45,731,923 | + | 2,922 | 534 | 61.412 | 10.5 | 8.436 | -0.127 | 2,263 | 1,605 | 41.2 | 6 | 377.2 | 131.8 | nucl |
| Gorai.007G092600 | CYP704C1 | *Gr_CYP704C1_245* | Chr07 | 6,756,063 | 6,758,568 | + | 2,506 | 506 | 58.559 | 14 | 9.142 | -0.271 | 1,949 | 1,521 | 41.8 | 6 | 324.8 | 111.4 | nucl |
| Gorai.007G092700 | CYP704C1 | *Gr_CYP704C1_246* | Chr07 | 6,772,774 | 6,775,288 | + | 2,515 | 546 | 62.735 | 16 | 9.1 | -0.253 | 1,974 | 1,641 | 42.1 | 6 | 329 | 108.2 | nucl |
| Gorai.001G047200 | CYP704C1 | *Gr_CYP704C1_247* | Chr01 | 4,469,772 | 4,472,279 | + | 2,508 | 588 | 67.786 | 11 | 8.204 | -0.033 | 1,938 | 1,767 | 40.4 | 5 | 387.6 | 139.5 | nucl |
| Gorai.004G155400 | CYP704C1 | *Gr_CYP704C1_248* | Chr04 | 44,013,053 | 44,016,335 | + | 3,283 | 512 | 59.342 | 5.5 | 7.563 | -0.133 | 1,762 | 1,539 | 42.4 | 5 | 352.4 | 380.3 | nucl |
| Gorai.007G092500 | CYP704C1 | *Gr_CYP704C1_249* | Chr07 | 6,750,245 | 6,752,370 | + | 2,126 | 524 | 60.67 | 10 | 8.344 | -0.178 | 1,742 | 1,575 | 39.4 | 5 | 348.4 | 88.8 | E.R. |
| Gorai.001G213800 | CYP97B2 | *Gr_CYP97B2_250* | Chr01 | 42,678,958 | 42,686,557 | - | 7,600 | 579 | 64.858 | 0.5 | 6.597 | -0.125 | 2,201 | 1,740 | 42.4 | 14 | 157.2 | 415.3 | nucl |
| Gorai.007G372200 | CYP97C1 | *Gr_CYP97C1_251* | Chr07 | 60,476,101 | 60,480,911 | - | 4,811 | 548 | 61.392 | 0.5 | 6.64 | -0.122 | 2,191 | 1,647 | 42 | 9 | 243.4 | 327.5 | E.R. |
| Gorai.013G180200 | CYP97A3 | *Gr_CYP97A3_252* | Chr13 | 47,440,822 | 47,446,631 | - | 5,810 | 616 | 68.868 | -4.5 | 5.658 | -0.202 | 2,199 | 1,851 | 42.9 | 16 | 137.4 | 240.7 | E.R. |
| Gorai.001G217300 | CYP749A22 | *Gr_ CYP749A22_253* | Chr01 | 43,503,379 | 43,505,836 | - | 2,458 | 519 | 59.519 | 14 | 8.961 | -0.092 | 1,906 | 1,560 | 39 | 5 | 381.2 | 138 | nucl |
| Gorai.013G101100 | CYP749A22 | *Gr_ CYP749A22_254* | Chr13 | 18,697,172 | 18,699,706 | + | 2,535 | 519 | 59.461 | 8 | 7.536 | -0.074 | 1,711 | 1,560 | 39.5 | 5 | 342.2 | 206 | E.R. |
| Gorai.011G158000 | CYP749A22 | *Gr_ CYP749A22_255* | Chr11 | 27,943,282 | 27,946,014 | + | 2,733 | 520 | 59.248 | 13 | 9.3 | -0.211 | 2,363 | 1,563 | 41.8 | 5 | 472.6 | 92.5 | mito |
| Gorai.005G033500 | CYP749A22 | *Gr_ CYP749A22_256* | Chr05 | 3,134,743 | 3,136,989 | + | 2,247 | 516 | 58.62 | 17.5 | 9.524 | -0.05 | 1,883 | 1,551 | 39.5 | 5 | 376.6 | 91 | nucl |
| Gorai.005G033600 | CYP749A22 | *Gr_ CYP749A22_257* | Chr05 | 3,154,960 | 3,157,014 | + | 2,055 | 500 | 56.933 | 11.5 | 8.791 | -0.07 | 1,503 | 1,503 | 40.2 | 6 | 250.5 | 110.4 | E.R. |
| Gorai.005G032600 | CYP749A22 | *Gr_ CYP749A22_258* | Chr05 | 2,938,564 | 2,941,500 | + | 2,937 | 516 | 59.033 | 15 | 9.227 | -0.137 | 1,924 | 1,551 | 39.8 | 5 | 384.8 | 253.3 | nucl |
| Gorai.005G032800 | CYP749A22 | *Gr_ CYP749A22_259* | Chr05 | 2,951,876 | 2,955,594 | + | 3,719 | 515 | 58.909 | 15 | 9.223 | -0.167 | 2,679 | 1,548 | 38.7 | 5 | 535.8 | 260 | nucl |
| Gorai.005G033400 | CYP749A22 | *Gr_ CYP749A22_260* | Chr05 | 3,127,336 | 3,132,500 | + | 5,165 | 527 | 60.052 | 13 | 8.915 | -0.097 | 1,778 | 1,584 | 40 | 5 | 355.6 | 846.8 | mito |
| Gorai.005G033700 | CYP749A22 | *Gr_ CYP749A22_261* | Chr05 | 3,182,311 | 3,185,657 | + | 3,347 | 511 | 58.039 | 10.5 | 8.068 | -0.056 | 2,204 | 1,536 | 40.3 | 6 | 367.3 | 228.6 | E.R. |
| Gorai.005G032900 | CYP749A22 | *Gr_ CYP749A22_262* | Chr05 | 2,967,500 | 2,970,000 | + | 2,501 | 525 | 60.021 | 12.5 | 8.545 | -0.114 | 2,014 | 1,578 | 39.8 | 5 | 402.8 | 121.8 | nucl |
| Gorai.005G033100 | CYP749A22 | *Gr_ CYP749A22_263* | Chr05 | 2,997,892 | 3,000,538 | + | 2,647 | 501 | 57.352 | 11.5 | 8.551 | -0.131 | 2,001 | 1,506 | 39.8 | 5 | 400.2 | 161.5 | nucl |
| Gorai.005G033200 | CYP749A22 | *Gr_ CYP749A22_264* | Chr05 | 3,012,026 | 3,015,149 | + | 3,124 | 516 | 58.56 | 24.5 | 9.905 | -0.107 | 2,456 | 1,551 | 39.5 | 5 | 491.2 | 167 | mito |
| Gorai.005G033300 | CYP749A22 | *Gr_ CYP749A22_265* | Chr05 | 3,117,325 | 3,120,107 | + | 2,783 | 512 | 58.465 | 23 | 9.896 | -0.122 | 2,060 | 1,539 | 39 | 5 | 412 | 180.8 | E.R. |
| Gorai.013G097000 | CYP749A22 | *Gr_ CYP749A22_266* | Chr13 | 16,794,235 | 16,802,585 | + | 8,351 | 543 | 61.665 | 22.5 | 9.941 | -0.076 | 2,284 | 1,632 | 41 | 6 | 380.7 | 1,213.40 | nucl |
| Gorai.013G071400 | CYP749A22 | *Gr_ CYP749A22_267* | Chr13 | 8,363,972 | 8,366,242 | + | 2,271 | 513 | 58.957 | 15 | 8.941 | -0.154 | 1,730 | 1,542 | 39.5 | 5 | 346 | 135.3 | nucl |
| Gorai.013G041300 | CYP749A22 | *Gr_ CYP749A22_268* | Chr13 | 3,458,916 | 3,461,355 | + | 2,440 | 529 | 60.51 | 13.5 | 9.175 | -0.164 | 1,974 | 1,590 | 41.5 | 6 | 329 | 93.2 | E.R. |
| Gorai.013G041900 | CYP749A22 | *Gr_ CYP749A22_269* | Chr13 | 3,518,798 | 3,520,676 | + | 1,879 | 547 | 62.494 | 13.5 | 8.924 | -0.141 | 1,644 | 1,644 | 41.1 | 4 | 411 | 78.3 | E.R. |
| Gorai.012G185300 | CYP749A22 | *Gr_ CYP749A22_270* | Chr12 | 35,237,473 | 35,241,784 | - | 4,312 | 513 | 58.371 | 14.5 | 9.365 | -0.162 | 1,704 | 1,542 | 41.8 | 5 | 340.8 | 652 | nucl |
| Gorai.004G270700 | CYP72A219 | *Gr_CYP72A219_271* | Chr04 | 60,556,538 | 60,559,538 | - | 3,001 | 521 | 59.608 | 17.5 | 9.581 | -0.113 | 1,910 | 1,566 | 43.5 | 5 | 382 | 272.8 | nucl |
| Gorai.002G043600 | CYP72A15 | *Gr_CYP72A15_272* | Chr02 | 3,599,274 | 3,602,753 | + | 3,480 | 557 | 64.585 | 14.5 | 8.918 | -0.205 | 2,054 | 1,674 | 41.9 | 6 | 342.3 | 285.2 | nucl |
| Gorai.002G043300 | CYP72A219 | *Gr_CYP72A219_273* | Chr02 | 3,568,023 | 3,575,406 | + | 7,384 | 209 | 24.18 | -4 | 5.071 | -0.407 | 627 | 627 | 42.3 | 3 | 209 | 3,378.50 | nucl |
| Gorai.002G043500 | CYP72A63 | *Gr_CYP72A63_274* | Chr02 | 3,593,469 | 3,594,067 | + | 599 | 154 | 17.571 | -4.5 | 4.904 | -0.11 | 513 | 465 | 42.4 | 2 | 256.5 | 86 | nucl |
| Gorai.002G043200 | CYP72A15 | *Gr_CYP72A15_275* | Chr02 | 3,559,636 | 3,576,081 | + | 16,446 | 518 | 59.888 | 13.5 | 9.299 | -0.241 | 2,128 | 1,557 | 42.8 | 5 | 425.6 | 81.5 | mito |
| Gorai.002G043400 | CYP72A15 | *Gr_CYP72A15_276* | Chr02 | 3,586,026 | 3,588,231 | + | 2,206 | 518 | 59.805 | 12.5 | 8.913 | -0.23 | 1,879 | 1,557 | 42.5 | 5 | 375.8 | 81.8 | mito |
| Gorai.009G035000 | CYP72A15 | *Gr_CYP72A15_277* | Chr09 | 2,617,942 | 2,620,104 | + | 2,163 | 517 | 59.119 | 15 | 8.956 | -0.178 | 1,796 | 1,554 | 42 | 5 | 359.2 | 91.8 | nucl |
| Gorai.004G198400 | CYP72A219 | *Gr_CYP72A219_278* | Chr04 | 51,944,788 | 51,945,689 | - | 902 | 237 | 27.279 | 6.5 | 9.627 | -0.349 | 714 | 714 | 39.9 | 3 | 238 | 94 | nucl |
| Gorai.008G154900 | CYP72A219 | *Gr_CYP72A219_279* | Chr08 | 41,386,504 | 41,389,234 | + | 2,731 | 514 | 59.043 | 18.5 | 9.576 | -0.226 | 1,836 | 1,545 | 43.2 | 5 | 367.2 | 223.8 | nucl |
| Gorai.001G002600 | CYP734A1 | *Gr_CYP734A1_280* | Chr01 | 221,412 | 224,591 | - | 3,180 | 518 | 59.718 | 25 | 9.78 | -0.055 | 1,892 | 1,557 | 44.5 | 5 | 378.4 | 322 | plas |
| Gorai.010G065800 | CYP734A1 | *Gr_CYP734A1_281* | Chr10 | 8,441,124 | 8,444,246 | - | 3,123 | 515 | 58.689 | 24.5 | 9.955 | 0.015 | 1,937 | 1,548 | 45.3 | 5 | 387.4 | 257.3 | nucl |
| Gorai.009G020500 | CYP734A1 | *Gr_CYP734A1_282* | Chr09 | 1,562,161 | 1,565,532 | - | 3,372 | 524 | 59.826 | 24.5 | 10.09 | -0.034 | 2,121 | 1,575 | 44.3 | 5 | 424.2 | 312.8 | plas |
| Gorai.004G278200 | CYP734A1 | *Gr_CYP734A1_283* | Chr04 | 61,089,865 | 61,093,531 | - | 3,667 | 517 | 59.202 | 22 | 9.864 | -0.048 | 2,173 | 1,554 | 44 | 5 | 434.6 | 373.5 | E.R. |
| Gorai.002G129200 | CYP734A1 | *Gr_CYP734A1_284* | Chr02 | 20,067,928 | 20,072,197 | + | 4,270 | 454 | 52.463 | 7.5 | 8.124 | -0.288 | 1,795 | 1,365 | 44.4 | 4 | 448.8 | 825 | nucl |
| Gorai.009G203900 | CYP734A1 | *Gr_CYP734A1_285* | Chr09 | 15,798,169 | 15,800,022 | + | 1,854 | 420 | 48.261 | 11.5 | 9.093 | -0.375 | 1,263 | 1,263 | 44.5 | 5 | 252.6 | 147.8 | nucl |
| Gorai.009G204000 | CYP734A1 | *Gr_CYP734A1_286* | Chr09 | 15,803,826 | 15,806,148 | + | 2,323 | 505 | 58.048 | 16 | 9.526 | -0.232 | 1,695 | 1,518 | 43.8 | 4 | 423.8 | 209.3 | nucl |
| Gorai.007G181100 | CYP735A1 | *Gr_CYP735A1_287* | Chr07 | 17,126,977 | 17,130,347 | + | 3,371 | 522 | 59.965 | 24.5 | 9.461 | -0.141 | 1,906 | 1,569 | 43.9 | 5 | 381.2 | 366.3 | cyto |
| Gorai.008G056100 | CYP735A1 | *Gr_CYP735A1_288* | Chr08 | 8,512,938 | 8,515,231 | - | 2,294 | 519 | 59.486 | 31 | 10.03 | -0.148 | 1,866 | 1,560 | 43.7 | 5 | 373.2 | 107 | cyto |
| Gorai.009G066700 | CYP714A1 | *Gr_CYP714A1_289* | Chr09 | 4,751,502 | 4,755,592 | + | 4,091 | 523 | 58.765 | 19.5 | 9.386 | -0.082 | 1,880 | 1,572 | 44.4 | 5 | 376 | 552.8 | cyto |
| Gorai.012G012600 | CYP714A1 | *Gr_CYP714A1_290* | Chr12 | 1,435,764 | 1,439,175 | + | 3,412 | 522 | 58.886 | 14 | 8.877 | -0.108 | 2,002 | 1,569 | 46.3 | 5 | 400.4 | 337 | nucl |
| Gorai.009G101200 | CYP714C2 | *Gr_CYP714C2_291* | Chr09 | 7,293,862 | 7,296,145 | - | 2,284 | 512 | 58.006 | 10.5 | 8.867 | 0.016 | 1,900 | 1,539 | 44.3 | 5 | 380 | 96 | E.R. |
| Gorai.008G110600 | CYP714C2 | *Gr_CYP714C2_292* | Chr08 | 34,170,042 | 34,172,939 | - | 2,898 | 522 | 59.08 | 15.5 | 9.057 | -0.063 | 2,322 | 1,569 | 41.7 | 6 | 387 | 115.2 | nucl |
| Gorai.001G208700 | CYP714C2 | *Gr_CYP714C2_293* | Chr01 | 41,251,056 | 41,253,600 | + | 2,545 | 516 | 57.862 | 11 | 8.711 | 0.009 | 2,118 | 1,551 | 44.7 | 5 | 423.6 | 106.8 | nucl |
| Gorai.011G231100 | CYP714B2 | *Gr_CYP714B2_294* | Chr11 | 54,603,259 | 54,605,218 | + | 1,960 | 510 | 57.408 | 13.5 | 8.626 | -0.011 | 1,624 | 1,533 | 42.7 | 5 | 324.8 | 84 | cyto |
| Gorai.012G023800 | CYP714C2 | *Gr_CYP714C2_295* | Chr12 | 2,938,865 | 2,940,806 | - | 1,942 | 514 | 57.828 | 16.5 | 9.033 | -0.081 | 1,556 | 1,545 | 42.9 | 5 | 311.2 | 96.5 | cyto |
| Gorai.006G198200 | CYP714A1 | *Gr_CYP714A1_296* | Chr06 | 45,547,510 | 45,549,561 | + | 2,052 | 521 | 59.438 | 27.5 | 10.13 | -0.167 | 1,570 | 1,566 | 45.4 | 4 | 392.5 | 160.7 | nucl |
| Gorai.008G276600 | CYP735A1 | *Gr_CYP735A1_297* | Chr08 | 55,428,301 | 55,433,223 | - | 4,923 | 519 | 58.882 | 16.5 | 9.497 | -0.077 | 1,854 | 1,560 | 43 | 4 | 463.5 | 1,023.00 | nucl |
| Gorai.009G207500 | CYP701A3 | *Gr_CYP701A3_298* | Chr09 | 16,099,310 | 16,100,968 | + | 1,659 | 383 | 44.465 | -2 | 6.203 | -0.419 | 1,152 | 1,152 | 41.2 | 8 | 144 | 72.4 | nucl |
| Gorai.010G015900 | CYP701A3 | *Gr_CYP701A3_299* | Chr10 | 1,221,707 | 1,224,905 | + | 3,199 | 502 | 57.654 | 10 | 8.09 | -0.318 | 1,875 | 1,509 | 44.9 | 7 | 267.9 | 220.7 | E.R. |
| Gorai.003G051700 | CYP89A9 | *Gr_CYP89A9_300* | Chr03 | 7,766,587 | 7,771,897 | - | 5,311 | 407 | 47.403 | 22 | 9.977 | -0.192 | 1,224 | 1,224 | 41.6 | 4 | 306 | 1,362.30 | nucl |
| Gorai.013G103500 | CYP89A2 | *Gr_CYP89A2_301* | Chr13 | 20,048,914 | 20,050,417 | + | 1,504 | 484 | 55.917 | 6 | 7.75 | -0.201 | 1,455 | 1,455 | 42.7 | 2 | 727.5 | 49 | nucl |
| Gorai.013G103400 | CYP89A2 | *Gr_CYP89A2_302* | Chr13 | 19,849,143 | 19,851,279 | + | 2,137 | 517 | 59.47 | 20.5 | 10.01 | -0.148 | 2,137 | 1,554 | 44 | 1 | 2,137.00 | No intron | nucl |
| Gorai.004G255300 | CYP89A9 | *Gr_CYP89A9_303* | Chr04 | 59,205,590 | 59,207,149 | + | 1,560 | 514 | 59.123 | 14.5 | 9.372 | -0.19 | 1,560 | 1,545 | 43.6 | 1 | 1,560.00 | No intron | nucl |
| Gorai.003G058600 | CYP89A2 | *Gr_CYP89A2_304* | Chr03 | 10,049,524 | 10,051,506 | + | 1,983 | 512 | 59.254 | 16.5 | 9.62 | -0.169 | 1,983 | 1,539 | 43.4 | 1 | 1,983.00 | No intron | plas |
| Gorai.007G347800 | CYP89A2 | *Gr_CYP89A2_305* | Chr07 | 57,788,882 | 57,790,696 | + | 1,815 | 509 | 58.775 | 15.5 | 9.358 | -0.151 | 1,815 | 1,530 | 42.6 | 1 | 1,815.00 | No intron | nucl |
| Gorai.007G347700 | CYP89A2 | *Gr_CYP89A2_306* | Chr07 | 57,779,914 | 57,781,455 | + | 1,542 | 513 | 58.73 | 10 | 8.563 | -0.074 | 1,542 | 1,542 | 43.1 | 1 | 1,542.00 | No intron | nucl |
| Gorai.007G347900 | CYP89A2 | *Gr_CYP89A2_307* | Chr07 | 57,794,054 | 57,796,315 | + | 2,262 | 512 | 58.79 | 15 | 9.689 | -0.148 | 2,262 | 1,539 | 42.9 | 1 | 2,262.00 | No intron | E.R. |
| Gorai.010G178900 | CYP77A3 | *Gr_CYP77A3_308* | Chr10 | 52,503,432 | 52,505,210 | + | 1,779 | 505 | 57.012 | 11.5 | 8.667 | -0.012 | 1,779 | 1,518 | 49.3 | 1 | 1,779.00 | No intron | mito |
| Gorai.010G252700 | CYP77A3 | *Gr_CYP77A3_309* | Chr10 | 61,867,520 | 61,869,248 | - | 1,729 | 506 | 57.387 | 11.5 | 8.61 | -0.077 | 1,729 | 1,521 | 46.9 | 1 | 1,729.00 | No intron | nucl |
| Gorai.011G016000 | CYP77A3 | *Gr_CYP77A3_310* | Chr11 | 1,120,589 | 1,122,109 | - | 1,521 | 506 | 57.451 | 9.5 | 8.395 | -0.036 | 1,521 | 1,521 | 47.6 | 1 | 1,521.00 | No intron | cyto |
| Gorai.008G098700 | CYP77A3 | *Gr_CYP77A3_311* | Chr08 | 28,098,578 | 28,100,519 | + | 1,942 | 519 | 59.176 | 19.5 | 9.798 | -0.145 | 1,942 | 1,560 | 42.1 | 1 | 1,942.00 | No intron | mito |
| Gorai.011G229800 | CYP77A3 | *Gr_CYP77A3_312* | Chr11 | 54,349,230 | 54,351,422 | - | 2,193 | 505 | 57.074 | 8.5 | 8.366 | -0.165 | 2,193 | 1,518 | 47.2 | 1 | 2,193.00 | No intron | E.R. |
| Gorai.001G188600 | CYP82C4 | *Gr_CYP82C4_313* | Chr01 | 30,397,054 | 30,400,049 | - | 2,996 | 530 | 59.593 | 0.5 | 6.562 | -0.043 | 2,672 | 1,593 | 45.6 | 2 | 1,336.00 | 324 | E.R. |
| Gorai.001G143500 | CYP82C2 | *Gr_CYP82C2_314* | Chr01 | 19,501,603 | 19,504,083 | + | 2,481 | 339 | 39.78 | 28.5 | 10.31 | -0.35 | 1,020 | 1,020 | 43.3 | 5 | 204 | 365.3 | E.R. |
| Gorai.007G156800 | CYP82C2 | *Gr_CYP82C2_315* | Chr07 | 13,543,613 | 13,545,482 | - | 1,870 | 528 | 59.778 | 20.5 | 9.474 | -0.119 | 1,748 | 1,587 | 46.3 | 2 | 874 | 122 | E.R. |
| Gorai.008G044400 | CYP82C4 | *Gr_CYP82C4_316* | Chr08 | 5,946,873 | 5,948,857 | - | 1,985 | 520 | 58.42 | 13 | 8.555 | -0.033 | 1,908 | 1,563 | 45.3 | 2 | 954 | 77 | nucl |
| Gorai.012G164300 | CYP82C4 | *Gr_CYP82C4_317* | Chr12 | 33,551,916 | 33,553,764 | - | 1,849 | 549 | 61.994 | 16 | 8.664 | -0.022 | 1,776 | 1,650 | 43.5 | 2 | 888 | 73 | nucl |
| Gorai.012G165100 | CYP82N4 | *Gr_CYP82N4_318* | Chr12 | 33,592,287 | 33,593,647 | - | 1,361 | 361 | 41.504 | 6 | 7.473 | -0.036 | 1,122 | 1,086 | 42.7 | 3 | 374 | 119.5 | nucl |
| Gorai.012G164400 | CYP82C4 | *Gr_CYP82C4_319* | Chr12 | 33,554,421 | 33,557,891 | - | 3,471 | 513 | 58.054 | 13.5 | 8.858 | -0.076 | 2,013 | 1,542 | 44.6 | 3 | 671 | 729 | nucl |
| Gorai.012G164500 | CYP82H23 | *Gr_CYP82H23 _320* | Chr12 | 33,569,217 | 33,570,140 | + | 924 | 307 | 35.318 | 18.5 | 10.3 | -0.152 | 924 | 924 | 42.2 | 1 | 924 | No intron | nucl |
| Gorai.009G274900 | CYP82A3 | *Gr_CYP82A3_321* | Chr09 | 23,026,578 | 23,028,912 | - | 2,335 | 526 | 59.902 | 9.5 | 8.145 | -0.204 | 2,025 | 1,581 | 47.8 | 2 | 1,012.50 | 310 | plas |
| Gorai.002G130200 | CYP82C4 | *Gr_CYP82C4_322* | Chr02 | 20,304,658 | 20,306,540 | + | 1,883 | 410 | 46.7 | 7.5 | 7.752 | -0.231 | 1,792 | 1,233 | 42.6 | 2 | 896 | 91 | plas |
| Gorai.009G206500 | CYP82A4 | *Gr_CYP82A4_323* | Chr09 | 15,976,553 | 15,978,241 | - | 1,689 | 390 | 44.101 | 5.5 | 7.425 | -0.351 | 1,440 | 1,173 | 46.6 | 4 | 360 | 83 | plas |
| Gorai.010G014200 | CYP82A1 | *Gr_CYP82A1_324* | Chr10 | 1,066,092 | 1,068,194 | - | 2,103 | 428 | 48.65 | 10.5 | 8.557 | -0.138 | 1,693 | 1,287 | 41.6 | 2 | 846.5 | 410 | plas |
| Gorai.010G014300 | CYP82A3 | *Gr_CYP82A3_325* | Chr10 | 1,076,607 | 1,079,103 | - | 2,497 | 526 | 59.695 | 4.5 | 7.003 | -0.217 | 1,780 | 1,581 | 42.6 | 2 | 890 | 717 | nucl |
| Gorai.010G014700 | CYP82A3 | *Gr_CYP82A3_326* | Chr10 | 1,108,091 | 1,111,717 | - | 3,627 | 559 | 63.501 | 12.5 | 8.54 | -0.169 | 1,680 | 1,680 | 42.3 | 5 | 336 | 486.8 | nucl |
| Gorai.010G014500 | CYP82A3 | *Gr_CYP82A3_327* | Chr10 | 1,098,515 | 1,100,287 | - | 1,773 | 524 | 59.73 | 6.5 | 7.716 | -0.153 | 1,661 | 1,575 | 41.4 | 2 | 830.5 | 112 | nucl |
| Gorai.010G014800 | CYP82A3 | *Gr_CYP82A3_328* | Chr10 | 1,113,155 | 1,114,953 | - | 1,799 | 525 | 59.581 | 6 | 7.656 | -0.17 | 1,687 | 1,578 | 41.9 | 2 | 843.5 | 112 | nucl |
| Gorai.010G015300 | CYP82A3 | *Gr_CYP82A3_329* | Chr10 | 1,169,661 | 1,171,434 | - | 1,774 | 374 | 42.282 | 12 | 9.419 | -0.002 | 1,482 | 1,125 | 40.7 | 6 | 247 | 58.4 | vacu |
| Gorai.010G015100 | CYP82A3 | *Gr_CYP82A3_330* | Chr10 | 1,145,423 | 1,154,806 | - | 9,384 | 315 | 36.271 | 12 | 9.547 | 0.028 | 948 | 948 | 37.3 | 6 | 158 | 1,687.20 | nucl |
| Gorai.010G014900 | CYP82A3 | *Gr_CYP82A3_331* | Chr10 | 1,119,799 | 1,121,721 | - | 1,923 | 408 | 45.887 | 10 | 9.137 | -0.157 | 1,769 | 1,227 | 42.7 | 2 | 884.5 | 154 | E.R. |
| Gorai.010G014400 | CYP82A4 | *Gr_CYP82A4_332* | Chr10 | 1,088,255 | 1,090,539 | - | 2,285 | 261 | 29.727 | 18 | 10.5 | -0.023 | 786 | 786 | 42.1 | 2 | 393 | 1,499.00 | E.R. |
| Gorai.010G014600 | CYP82A4 | *Gr_CYP82A4_333* | Chr10 | 1,103,552 | 1,105,532 | - | 1,981 | 525 | 59.902 | 15 | 8.899 | -0.136 | 1,838 | 1,578 | 41.8 | 2 | 919 | 143 | E.R. |
| Gorai.010G015000 | CYP82A3 | *Gr_CYP82A3_334* | Chr10 | 1,133,766 | 1,140,314 | - | 6,549 | 985 | 111.256 | 26 | 9.124 | -0.103 | 3,135 | 2,958 | 42 | 5 | 627 | 853.5 | plas |
| Gorai.010G015200 | CYP82A3 | *Gr_CYP82A3_335* | Chr10 | 1,157,678 | 1,159,586 | - | 1,909 | 532 | 59.932 | 15 | 9.185 | -0.133 | 1,773 | 1,599 | 42.2 | 2 | 886.5 | 136 | E.R. |
| Gorai.009G207000 | CYP82A3 | *Gr_CYP82A3_336* | Chr09 | 16,043,634 | 16,045,885 | - | 2,252 | 526 | 58.759 | 8.5 | 8.301 | -0.032 | 2,097 | 1,581 | 47.7 | 2 | 1,048.50 | 155 | E.R. |
| Gorai.010G015400 | CYP82A3 | *Gr_CYP82A3_337* | Chr10 | 1,175,987 | 1,177,879 | - | 1,893 | 521 | 58.926 | 10 | 8.96 | -0.147 | 1,800 | 1,566 | 43.3 | 2 | 900 | 93 | nucl |
| Gorai.009G206700 | CYP82A3 | *Gr_CYP82A3_338* | Chr09 | 15,989,671 | 15,991,727 | - | 2,057 | 522 | 58.49 | 14 | 9.158 | -0.211 | 1,968 | 1,569 | 45.4 | 2 | 984 | 89 | E.R. |
| Gorai.009G206900 | CYP82A3 | *Gr_CYP82A3_339* | Chr09 | 16,026,638 | 16,028,703 | + | 2,066 | 523 | 58.693 | 15 | 9.267 | -0.187 | 1,979 | 1,572 | 44.3 | 2 | 989.5 | 87 | E.R. |
| Gorai.009G331600 | CYP82A3 | *Gr_CYP82A3_340* | Chr09 | 34,388,631 | 34,390,630 | - | 2,000 | 522 | 58.307 | 13 | 9.132 | -0.217 | 1,908 | 1,569 | 44.8 | 2 | 954 | 92 | cyto |
| Gorai.009G206600 | CYP82A3 | *Gr_CYP82A3_341* | Chr09 | 15,982,718 | 15,984,960 | - | 2,243 | 522 | 58.557 | 13 | 9.235 | -0.218 | 2,154 | 1,569 | 44.9 | 2 | 1,077.00 | 89 | plas |
| Gorai.007G208700 | CYP82C4 | *Gr_CYP82C4_342* | Chr07 | 21,934,039 | 21,936,661 | - | 2,623 | 525 | 59.387 | 8 | 7.564 | -0.106 | 2,473 | 1,578 | 44.8 | 2 | 1,236.50 | 150 | E.R. |
| Gorai.007G208800 | CYP82C4 | *Gr_CYP82C4_343* | Chr07 | 21,964,254 | 21,966,642 | - | 2,389 | 585 | 66.293 | 15.5 | 8.182 | -0.106 | 2,239 | 1,758 | 43.9 | 2 | 1,119.50 | 150 | E.R. |
| Gorai.009G207100 | CYP82C4 | *Gr_CYP82C4_344* | Chr09 | 16,045,967 | 16,048,061 | - | 2,095 | 525 | 59.068 | 11.5 | 8.461 | -0.091 | 1,848 | 1,578 | 46.5 | 2 | 924 | 247 | E.R. |
| Gorai.001G104500 | CYP82G1 | *Gr_CYP82G1_345* | Chr01 | 11,664,349 | 11,666,682 | + | 2,334 | 521 | 59.425 | 17 | 9.027 | -0.233 | 1,794 | 1,566 | 46.2 | 2 | 897 | 540 | nucl |
| Gorai.001G104400 | CYP82G1 | *Gr_CYP82G1_346* | Chr01 | 11,633,700 | 11,636,650 | + | 2,951 | 515 | 58.609 | 10 | 7.895 | -0.191 | 1,961 | 1,548 | 46.6 | 2 | 980.5 | 990 | E.R. |
| Gorai.001G104600 | CYP82G1 | *Gr_CYP82G1_347* | Chr01 | 11,712,694 | 11,715,660 | + | 2,967 | 521 | 59.146 | 8.5 | 7.7 | -0.175 | 1,802 | 1,566 | 45 | 2 | 901 | 1,165.00 | plas |
| Gorai.005G100700 | CYP81E8 | *Gr_CYP81E8_348* | Chr05 | 15,869,399 | 15,872,188 | + | 2,790 | 536 | 61.592 | 13 | 8.46 | -0.036 | 2,228 | 1,611 | 43.5 | 3 | 742.7 | 281 | plas |
| Gorai.010G024400 | CYP81E8 | *Gr_CYP81E8_349* | Chr10 | 1,948,277 | 1,950,146 | - | 1,870 | 500 | 57.19 | 3.5 | 7.189 | -0.119 | 1,790 | 1,503 | 44.6 | 2 | 895 | 80 | nucl |
| Gorai.009G276900 | CYP81E8 | *Gr_CYP81E8_350* | Chr09 | 23,231,439 | 23,233,564 | - | 2,126 | 501 | 57.624 | 17.5 | 9.253 | -0.22 | 2,038 | 1,506 | 46.3 | 2 | 1,019.00 | 88 | E.R. |
| Gorai.005G100400 | CYP81E7 | *Gr_CYP81E7_351* | Chr05 | 15,829,335 | 15,831,534 | - | 2,200 | 508 | 57.927 | 5 | 7.498 | -0.177 | 2,109 | 1,527 | 45.5 | 2 | 1,054.50 | 91 | plas |
| Gorai.009G217000 | CYP81E8 | *Gr_CYP81E8_352* | Chr09 | 16,884,554 | 16,886,457 | + | 1,904 | 542 | 61.786 | 11 | 8.453 | -0.203 | 1,829 | 1,629 | 45.2 | 2 | 914.5 | 75 | E.R. |
| Gorai.009G216500 | CYP81E1 | *Gr_CYP81E1_353* | Chr09 | 16,843,393 | 16,845,959 | - | 2,567 | 518 | 58.268 | 3.5 | 7.089 | -0.109 | 1,669 | 1,557 | 43.7 | 2 | 834.5 | 898 | E.R. |
| Gorai.007G219100 | CYP81E8 | *Gr_CYP81E8_354* | Chr07 | 25,069,900 | 25,071,662 | - | 1,763 | 500 | 56.576 | 3.5 | 6.923 | -0.166 | 1,682 | 1,503 | 45.8 | 2 | 841 | 81 | nucl |
| Gorai.007G219200 | CYP81E8 | *Gr_CYP81E8_355* | Chr07 | 25,116,564 | 25,118,088 | - | 1,525 | 405 | 46.508 | -4 | 5.851 | -0.161 | 1,218 | 1,218 | 41.9 | 5 | 243.6 | 76.8 | nucl |
| Gorai.008G059000 | CYP81D1 | *Gr_CYP81D1_356* | Chr08 | 9,315,143 | 9,316,966 | - | 1,824 | 500 | 56.995 | 12.5 | 8.706 | -0.241 | 1,747 | 1,503 | 44.8 | 2 | 873.5 | 77 | plas |
| Gorai.001G089000 | CYP81E8 | *Gr_CYP81E8__357* | Chr01 | 9,618,655 | 9,620,814 | + | 2,160 | 347 | 39.657 | 10 | 8.732 | -0.212 | 1,044 | 1,044 | 42.3 | 7 | 149.1 | 186 | plas |
| Gorai.001G088900 | CYP81D1 | *Gr_CYP81D1_358* | Chr01 | 9,602,033 | 9,604,342 | + | 2,310 | 501 | 56.593 | 13 | 9.021 | -0.191 | 1,748 | 1,506 | 44.1 | 2 | 874 | 562 | vacu |
| Gorai.001G089100 | CYP81D1 | *Gr_CYP81D1_359* | Chr01 | 9,633,456 | 9,635,234 | + | 1,779 | 326 | 36.705 | -7 | 5.126 | -0.392 | 1,586 | 981 | 43.4 | 2 | 793 | 193 | nucl |
| Gorai.003G027500 | CYP81E8 | *Gr_CYP81E8__360* | Chr03 | 2,488,478 | 2,491,972 | - | 3,495 | 507 | 57.051 | 12 | 8.373 | -0.119 | 1,545 | 1,524 | 44.7 | 2 | 772.5 | 1,950.00 | E.R. |
| Gorai.008G079200 | CYP81E8 | *Gr_CYP81E8__361* | Chr08 | 15,247,029 | 15,249,651 | - | 2,623 | 504 | 57.232 | 8.5 | 8.074 | -0.112 | 1,878 | 1,515 | 44.1 | 2 | 939 | 745 | plas |
| Gorai.011G273000 | CYP81E8 | *Gr_CYP81E8_362* | Chr11 | 60,525,101 | 60,527,733 | + | 2,633 | 457 | 52.32 | 22 | 9.738 | -0.134 | 1,371 | 1,371 | 43.3 | 2 | 685.5 | 1,262.00 | E.R. |
| Gorai.011G272900 | CYP81E8 | *Gr_CYP81E8_363* | Chr11 | 60,498,648 | 60,501,058 | + | 2,411 | 460 | 52.978 | 23 | 9.787 | -0.098 | 1,380 | 1,380 | 42.5 | 2 | 690 | 1,031.00 | plas |
| Gorai.011G273100 | CYP81E8 | *Gr_CYP81E8_364* | Chr11 | 60,534,096 | 60,536,482 | + | 2,387 | 457 | 52.53 | 22 | 9.772 | -0.128 | 1,371 | 1,371 | 43.7 | 2 | 685.5 | 1,016.00 | cyto |
| Gorai.002G228800 | CYP93A1 | *Gr_CYP93A1_365* | Chr02 | 58,787,871 | 58,790,217 | + | 2,347 | 514 | 58.391 | 6.5 | 7.75 | -0.153 | 1,788 | 1,545 | 42.8 | 2 | 894 | 559 | nucl |
| Gorai.006G140400 | CYP93A1 | *Gr_CYP93A1_366* | Chr06 | 39,707,706 | 39,709,386 | + | 1,681 | 508 | 58.097 | 15 | 9.041 | -0.187 | 1,555 | 1,527 | 44.3 | 2 | 777.5 | 126 | nucl |
| Gorai.006G140500 | CYP93A1 | *Gr_CYP93A1_367* | Chr06 | 39,709,869 | 39,716,575 | + | 6,707 | 437 | 49.473 | 13.5 | 9.169 | -0.261 | 1,468 | 1,314 | 43 | 3 | 489.3 | 2,619.50 | E.R. |
| Gorai.006G140600 | CYP93A1 | *Gr_CYP93A1_368* | Chr06 | 39,723,903 | 39,725,752 | + | 1,850 | 512 | 58.33 | 14.5 | 9.007 | -0.272 | 1,729 | 1,539 | 42.9 | 2 | 864.5 | 121 | E.R. |
| Gorai.006G217000 | CYP93B1 | *Gr_CYP93B1_369* | Chr06 | 46,985,837 | 46,987,505 | - | 1,669 | 415 | 46.794 | 15.5 | 9.238 | -0.062 | 1,248 | 1,248 | 47.6 | 4 | 312 | 140.3 | cyto |
| Gorai.013G141500 | CYP93B1 | *Gr_CYP93B1_370* | Chr13 | 38,454,969 | 38,457,298 | - | 2,330 | 519 | 58.915 | 7 | 7.443 | -0.076 | 1,614 | 1,560 | 45.3 | 2 | 807 | 716 | E.R. |
| Gorai.002G228600 | CYP93A3 | *Gr_CYP93A3_371* | Chr02 | 58,744,794 | 58,747,084 | - | 2,291 | 505 | 57.676 | 4 | 7.013 | -0.131 | 1,662 | 1,518 | 41.4 | 2 | 831 | 629 | nucl |
| Gorai.006G140700 | CYP93A1 | *Gr_CYP93A1_372* | Chr06 | 39,734,329 | 39,739,921 | + | 5,593 | 517 | 58.841 | 6.5 | 7.796 | -0.198 | 2,250 | 1,554 | 42.7 | 2 | 1,125.00 | 180 | E.R. |
| Gorai.002G228700 | CYP93A1 | *Gr_CYP93A1_373* | Chr02 | 58,751,253 | 58,753,135 | - | 1,883 | 515 | 58.535 | 7.5 | 7.89 | -0.257 | 1,807 | 1,548 | 42.1 | 2 | 903.5 | 76 | nucl |
