# Supplementary material for: Knockdown of Cytochrome P450 Genes Gh_D07G1197 and Gh_A13G2057 on Chromosomes D07 and A13 Reveals Their Putative Role in Enhancing Drought and Salt Stress Tolerance in Gossypium hirsutum
Source: Genes (Basel). 2019 Mar 18;10(3):226. doi: 10.3390/genes10030226 (PMC6471685; doi:10.3390/genes10030226)
Supplement: Supplementary file 1 [file genes-10-00226-s001.zip › Supplementary files/Table S5.docx]

**Table S5**: Cis-regulatory elements with profound role in abiotic stress responses found within the promoter regions of the various cotton cytochromes (CYP450) genes. The cis-regulatory elements were analyzed in the 1 kb up and down stream regions of translation start site using the PLACE database.

| Cis-Regulatory-elements | Signal Sequence | Function |
| --- | --- | --- |
| ABRELATERD1 | ACGTG | Early responsive to dehydration |
| ACGTABREMOTIFA2OSEM | ACGTGKC | ABA-responsive expression |
| ACGTATERD1 | ACGT | Early responsive to dehydration |
| ASF1MOTIFCAMV | TGACG | Abiotic/biotic stress responsiveness |
| CBFHV | RYCGAC | Dehydration-responsive element (DRE) binding proteins |
| CCAATBOX1 | CCAAT | Heat shock protein |
| DRECRTCOREAT | RCCGAC | Activators that function in drought-, high-salt- and drought stress |
| MYB2CONSENSUSAT | YAACKG | Dehydration-responsive |
| MYBCORE | CNGTTR | Dehydration/water stress |
| MYCATERD1 | CATGTG | Early responsive to dehydration |
| MYCATRD22 | CACATG | Dehydration/Water stress |
| MYCCONSENSUSAT | CANNTG | Dehydration-responsive |
| MYB1AT | WAACCA | Abiotic stress responsiveness |
| ABREATCONSENSUS | YACGTGGC | Stress-responsive abscisic acid signalling/enhance abiotic stress tolerance |
| ABREOSRAB21 | ACGTSSSC | Response to abscisic acid and osmotic stress |
| ARE1 | RGTGACNNNGC | antioxidant response element |
| DRE2COREZMRAB17 | ACCGAC | Drought-responsive element |
| AGMOTIFNTMYB2 | AGATCCAA | induced by various stress such as wounding or elicitor treatment |
| MYBATRD22 | CTAACCA | Dehydration-responsive |
| CRTDREHVCBF2 | GTCGAC | involved in regulation of low-temperature responsive genes |
| DRE1COREZMRAB17 | ACCGAGA | Drought response |
| NAPINMOTIFBN | TACACAT | dehydration-responsive |
| ABREATRD22 | RYACGTGGYR | ABA responsive element/ dehydrative responsiveness |
| ABRECE1HVA22 | TGCCACCGG | ABA responsive element/ dehydrative responsiveness |
| DREDR1ATRD29AB | TACCGACAT | Activators that function in drought-, high-salt- and drought stress |
| ACGTABREMOTIFAOSOSEM | TACGTGTC | ABA-responsive expression |
| ABREZMRAB28 | CCACGTGG | ABA and water-stress responses |
| ABREDISTBBNNAPA | GCCACTTGTC | ABA responsive element/ dehydrative responsiveness |
| ABREAZMRAB28 | GCCACGTGGG | ABA responsive element/ dehydrative responsiveness |

R: G A (puRine); Y: T C (pYrimidine); K: G T (Keto); S: G C (Strong); W: A T (Weak) and N: A GCT (aNy)
