# Supplementary figures and images for: Knockdown of Cytochrome P450 Genes Gh_D07G1197 and Gh_A13G2057 on Chromosomes D07 and A13 Reveals Their Putative Role in Enhancing Drought and Salt Stress Tolerance in Gossypium hirsutum
Source: Genes (Basel). 2019 Mar 18;10(3):226. doi: 10.3390/genes10030226 (PMC6471685; doi:10.3390/genes10030226)

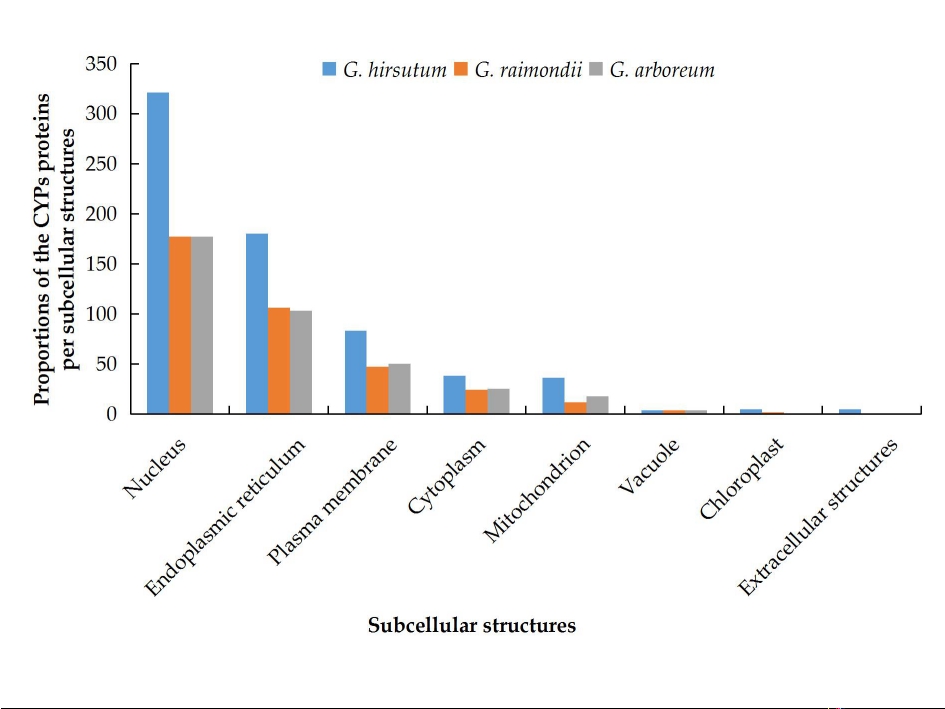

Supplement: Supplementary file 1 [file genes-10-00226-s001.zip › Supplementary files/Figure S1.jpg]

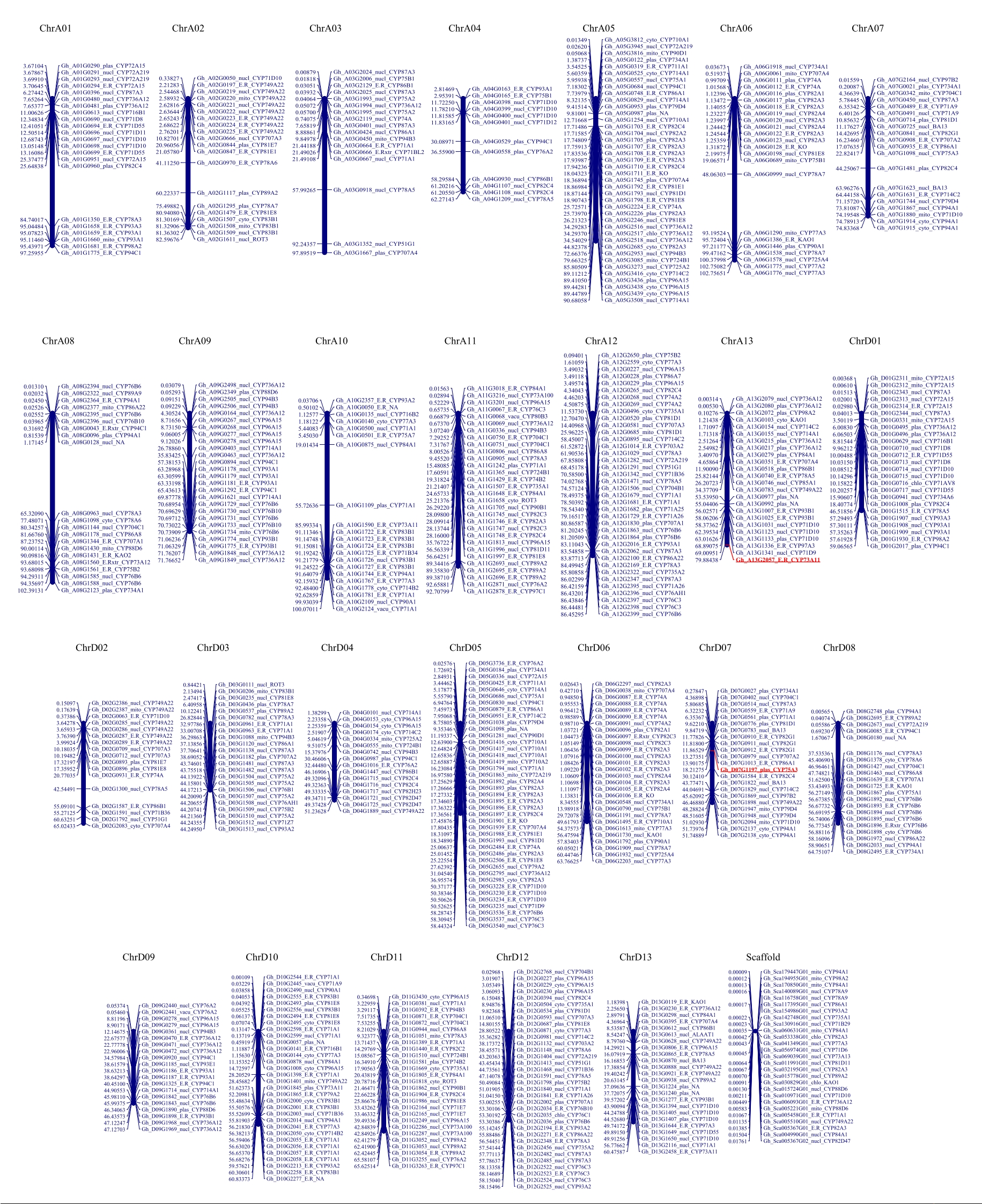

Supplement: Supplementary file 1 [file genes-10-00226-s001.zip › Supplementary files/Figure S2.jpg]

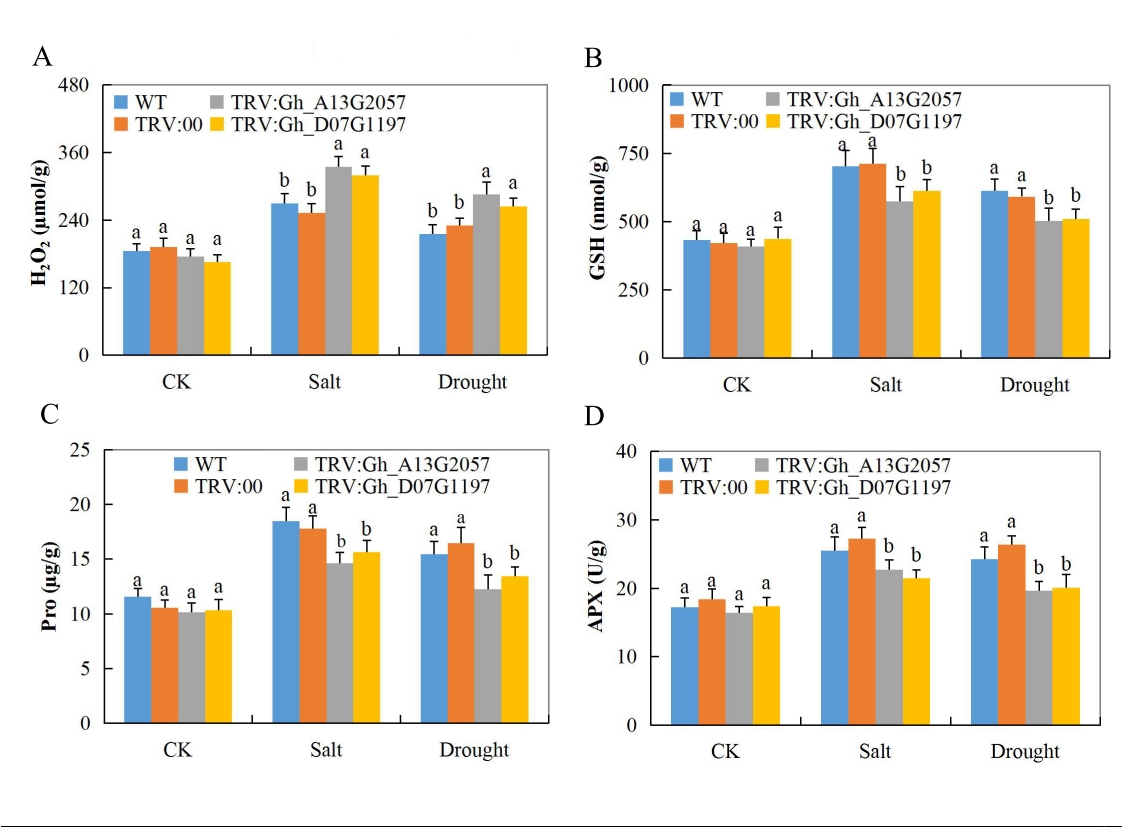

Supplement: Supplementary file 1 [file genes-10-00226-s001.zip › Supplementary files/Figure S3.jpg]

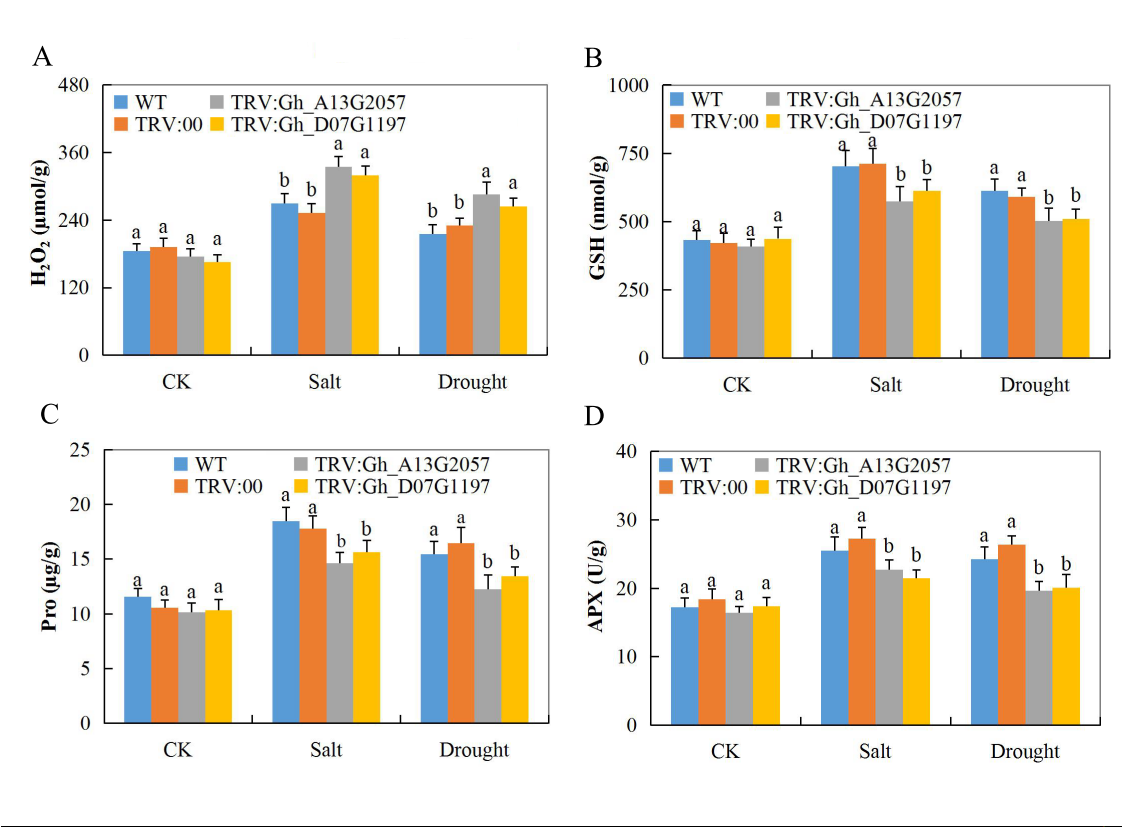

Supplement: Supplementary file 1 [file genes-10-00226-s001.zip › Supplementary files/Figure S3.tif]
